# Analysis of copy number variants by three detection algorithms and their association with body size in horses

**DOI:** 10.1186/1471-2164-14-487

**Published:** 2013-07-18

**Authors:** Julia Metzger, Ute Philipp, Maria Susana Lopes, Artur da Camara Machado, Michela Felicetti, Maurizio Silvestrelli, Ottmar Distl

**Affiliations:** 1 Institute for Animal Breeding and Genetics, University of Veterinary Medicine Hannover, Bünteweg 17p, 30559, Hannover, Germany; 2 Biotechnology Centre of Azores, Associated Laboratory Institute for Biotechnology and Bioengineering, University of Azores, Rua Capitão João D’Ávila, 9700-042 Angra do Heroísmo, Azores, Portugal; 3 Sport Horse Research Centre - Department of Pathology, Diagnostic & Veterinary Clinic - Faculty of Veterinary Medicine, University of Perugia, via S. Costanzo 4, 06126, Perugia, Italy

**Keywords:** Body size, Copy number variation

## Abstract

**Background:**

Copy number variants (CNVs) have been shown to play an important role in genetic diversity of mammals and in the development of many complex phenotypic traits. The aim of this study was to perform a standard comparative evaluation of CNVs in horses using three different CNV detection programs and to identify genomic regions associated with body size in horses.

**Results:**

Analysis was performed using the Illumina Equine SNP50 genotyping beadchip for 854 horses. CNVs were detected by three different algorithms, CNVPartition, PennCNV and QuantiSNP. Comparative analysis revealed 50 CNVs that affected 153 different genes mainly involved in sensory perception, signal transduction and cellular components. Genome-wide association analysis for body size showed highly significant deleted regions on ECA1, ECA8 and ECA9. Homologous regions to the detected CNVs on ECA1 and ECA9 have also been shown to be correlated with human height.

**Conclusions:**

Comparative analysis of CNV detection algorithms was useful to increase the specificity of CNV detection but had certain limitations dependent on the detection tool. GWAS revealed genome-wide associated CNVs for body size in horses.

## Background

The determination of copy number variants (CNVs) has become increasingly important for the evaluation of genomic traits in domestic animals [1]. CNVs have been shown to be a major source for genetic variation especially in complex traits influencing gene expression, phenotypic variation, adaption and the development of diseases [2,3]. Analyses using different detection methods have been performed in diverse species like cows, pigs and horses [4-7]. Recent whole-genome sequencing revealed 282 CNVs in a quarter horse mare and suggested CNVs to be an important resource for future studies of complex diseases and traits in horses [8]. CNV studies for horses were carried out by the array comparative genomic hybridization (CGH) methodology and the CNV detection algorithm PennCNV using whole genome SNP genotyping chips [4,5]. Both techniques have been shown to be a valid method to detect CNVs [3,6,9]. It was proposed that a comparison between these methods might be an important step for understanding the advantages and disadvantages of these platforms [9]. SNP arrays were proposed to be advantageous due to lower prices, lower signal-to-noise ratios and the use of the parameter B-allele frequency which facilitates the interpretation of results [1,6,10]. Furthermore, less samples per experiment were assumed to be required for SNP genotyping compared to CGH analyses [11]. However, the main bias for SNP arrays was considered to be the low SNP coverage of the genome in regions of CNVs due to difficulties of assay development and implementation [11]. In CGH arrays the genome coverage was shown to be highly dependent of the study design. In pig breeds CGH analyses have been proposed to be advantageous due to an enhanced marker density and a uniform distribution of probes [12]. CNV analyses in horses were performed on a CGH array which only targeted exons and therefore did not allow an identification of CNVs in intergenic regions [5]. This was taken into account for the benefit of detecting smaller CNVs in coding exons of annotated genes [5].

CNV calls in CGH as well as SNP arrays have been considered to be highly dependent on the algorithms used for the identification of CNVs [11,13,14]. For SNP arrays, various CNV detection programs have been available. Comparative analyses of CNV detection algorithms suggested that multiple predictions from different detection programs increased the confidence in the data and helped to eliminate false positive results [11]. Nevertheless the accuracy of CNV detection by different algorithms has also been shown to be limited due to false negative results [15,16]. SNP array analyses in horses have not been performed in more than one CNV detection algorithm yet [4]. On the other hand, the analysed horse populations varied from a low number of horses (16) of diverse breeds used in the CGH study, to a higher number of horses (520) of a few breeds (4) in SNP array based PennCNV detections [4,5,17]. Due to the development of the horse population into a highly variable group, evaluations of different breeds have been shown to be important, especially for complex variations which are supposed to be challenging due to genetic heterogeneity and variations in the phenotypic expression [18,19].

Genome-wide association studies (GWAS) for height have been performed for CNVs in human and revealed copy-number variants that were proposed to play a role in the development of short stature [20]. Recent studies in horses identified CNVs in the region of genes mainly involved in sensory perception, signal transduction and metabolism but also in candidates for neuronal homeostasis, coat colour, blood group antigens, keratin formation and height [5]. A quantitative trait locus (QTL) for stature was found on equine chromosome (ECA) 16 at 75 Mb. Size variation in horses has been investigated in various GWAS for single nucleotide polymorphisms (SNPs) using BeadChip data [19,21-23]. On the whole, five loci on ECA3, ECA6, ECA9, ECA11 and ECA28 have been discussed to be probably involved in the determination of withers height. A functional polymorphism on ECA3 was shown to be highly associated with body size and with the relative expression levels of the adjacent gene *ligand dependent nuclear receptor corepressor-like (LCORL)*[23]. It was assumed that *LCORL* might be a main regulator for the determination of body size in horses.

The aim of this study was to perform CNV detection analyses in accordance with current standards using three CNV detection algorithms in a large number of horses of various breeds and to compare these results with current microarray studies. The CNVs detected were further analysed for their association with body size as a model for complex traits.

## Results and discussion

### CNV detection

**Figure 1:**
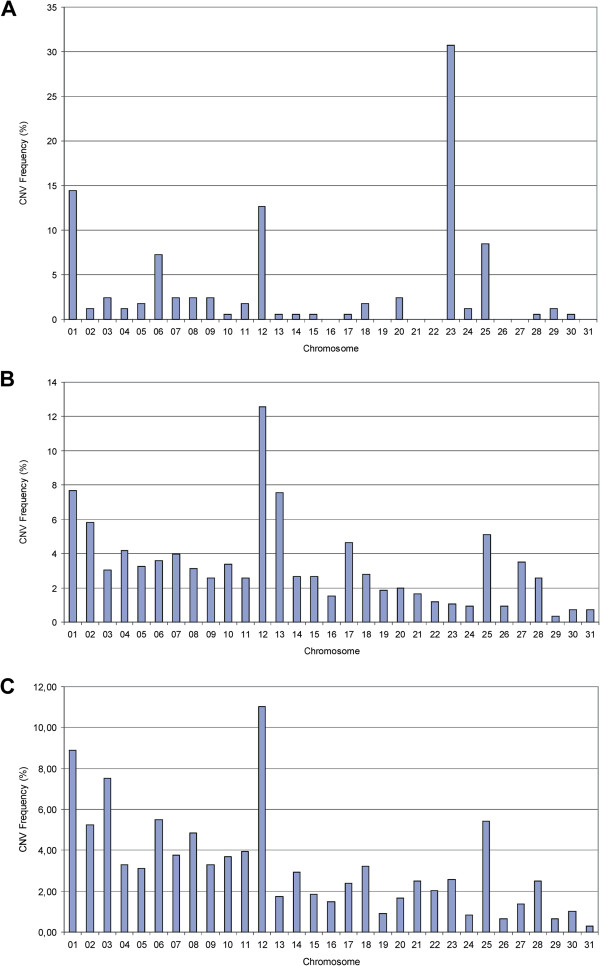
**Chromosomal distribution of CNVs detected by different detection algorithms.** (**A**) Detection results of CNVPartition. (**B**) Detection results of PennCNV. (**C**) Detection results of QuantiSNP.

The detection of CNVs was performed on the data of the Illumina Equine SNP50 beadchip using the algorithms CNVPartition (Illumina), PennCNV [17] and QuantiSNP [24]. Analysis revealed 166, 860 and 1090 CNVs using these programs for the detection (Additional file 1, Additional file 2 and Additional file 3). The mean size for all detected CNVs was 487,562 bp and the median 169,367 bp. Considering the distribution of CNVs over the chromosomes, the detection results of PennCNV revealed the largest number of CNVs on ECA1, ECA12 and ECA13 while the results of QuantiSNP showed an enrichment of CNVs on ECA1, ECA3 and ECA12 (Figure 1). Detection analysis by CNVPartition revealed a high number of detected CNVs on ECA1, ECA12 and ECA23. However, the chromosomes with the highest numbers of CNVs did not necessarily show the highest coverage with CNV regions. We found strong CNV coverage enrichment on ECA23 (CNVPartition), ECA13 (PennCNV), ECA27 and ECA28 (PennCNV and QuantiSNP) and ECA12 (all three programs, Table 1). Across all three detection algorithms, ECA12 was not only significantly enriched by CNVs but also showed the largest number of detected CNVs. An accumulation of CNVs has also been reported in CGH analyses in horses for ECA12, ECA17 and ECA23 and was shown in Illumina Equine SNP50 beadchip based PennCNV analyses for ECA1, ECA2 and ECA17 [4,5]. We assume that the amount of detected CNVs on specific chromosomes is dependent on the detection method and can vary among different populations. Nevertheless, there is much evidence to presume that ECA12, ECA27 and ECA28 are considerably enriched for CNVs.

**Table 1:** Chromosomal enrichment of CNVs detected by three different algorithms

**Chromosome**	**CNVPartition (%)**	**PennCNV (%)**	**QuantiSNP (%)**
1	2.1	4.3	18.5
2	0.6	7.6	31.0
3	0.5	3.6	29.1
4	0.8	4.7	20.7
5	0.5	4.2	18.6
6	1.8	4.2	22.0
7	0.9	6.6	19.4
8	0.5	3.9	15.1
9	1.2	4.7	15.1
10	1.3	7.1	26.3
11	2.5	4.0	25.9
12	8.6	15.7	54.7
13	1.4	91.8	36.3
14	0.3	3.6	19.9
15	0.9	4.7	10.2
16	0	2.1	10.2
17	1.7	7.7	21.3
18	1.5	5.3	14.1
19	0	3.6	3.1
20	2.3	2.7	5.2
21	0	4.4	19.1
22	0	3.0	28.6
23	26.4	2.9	15.0
24	1.0	2.5	4.9
25	3.6	6.2	22.5
26	0	3.0	11.0
27	0	79.1	90.9
28	0.4	70.7	65.4
29	1.4	1.5	8.6
30	3.6	4.3	36.8
31	0	4.1	3.9
Total coverage	1.7	9.0	22.0

The proportion of the chromosome covered by detected CNVs is shown.

### Comparison between three detection programs

**Figure 2:**
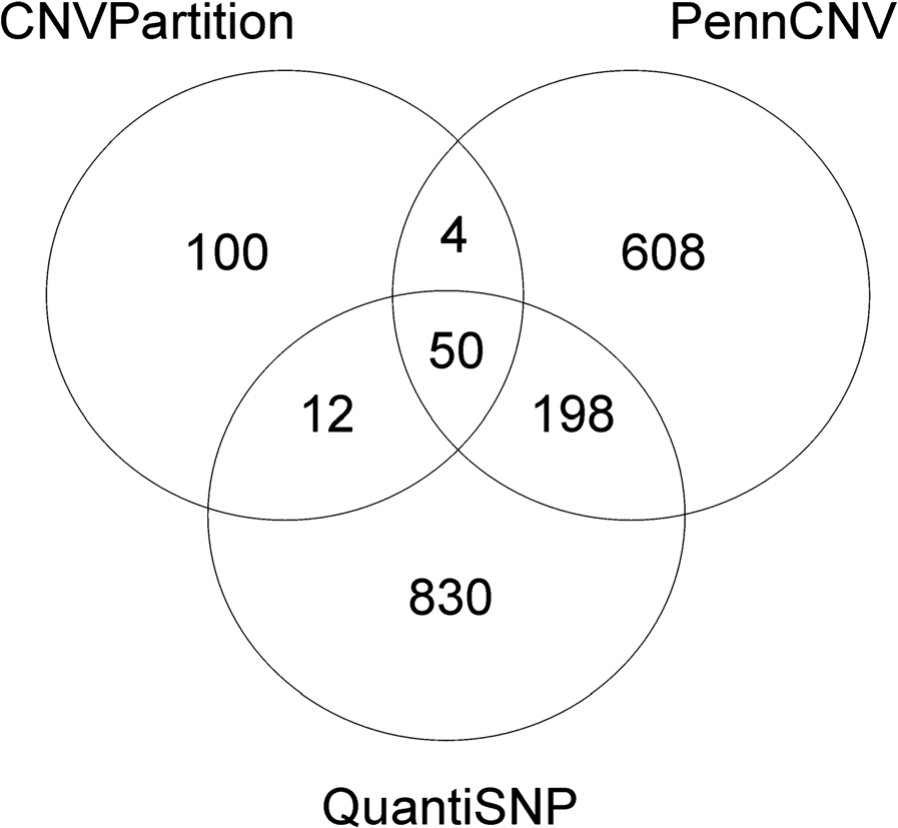
**Overlapping CNVs from the three CNV-detection programs used in analysis.** 50 CNVs could be detected by all three algorithms.

Comparative analysis between the algorithms showed that PennCNV and QuantiSNP detected similar numbers of CNVs identified on each autosome. They displayed a CNV detection overlap of 28.4% and 22.8% (Figure 2). The percentage of CNVs overlapping with CNVPartition, the algorithm with the lowest number of detected CNVs, was 32.5% (PennCNV) and 37.4% (QuantiSNP). In total, 50 CNVs could be detected by all three programs (Additional file 4). The average size of these 50 CNVs detected by all three programs was 388,892 bp and ranged from 516 to 978,353 bp (median size 293,244 bp). The number of losses and gains was computed among those breeds that revealed a CNV in all three algorithms (see Additional file 4). On the whole, five CNVs showed higher copy numbers in some and lower copy numbers in other horses while further 28 CNVs only displayed losses and 17 CNVs only gains in these horses.

Comparisons between our detection results of the 50 CNVs detected in all three programs with recent CNV studies [4,5] showed that these 50 CNV regions were highly similar to those CNV regions in previous studies especially for SNP array analyses (Table 2). Nevertheless, the study by Dupuis *et al.* revealed a considerably higher number of detected CNVs [4]. We propose that this effect was a result of more stringent quality criteria and the combination of three detection algorithms used in our study.

**Table 2:** CNV regions from 50 CNVs of three detection algorithms in comparison with recent studies

**ECA**	**CNV regions detected by CNVPartition, PennCNV, QuantiSNP (717 horses) (bp)**	**CN**	**Samples (n)**	**CNV regions detected by PennCNV (477 horses); Dupuis *et al. *(bp)**	**CN**	**Samples (n)**	**CNV regions detected CGH analysis (16 horses); Doan *et al. *(bp)**	**CN**	**Samples (n)**
1	155487276-155656642	Loss, gain	162, 13	155487276-155656642	Loss	78	155360077–155640397	Loss, gain	3
							155905880–155936772	Loss	1
							155936817–155937275	Gain	2
							155972933–155973160	Gain	1
							156313490–156314132	Loss	1
							156657191–156690829	Loss	1
							156690978–156714131	Gain	6
1	178798269- 179550475	Loss, gain	10, 5	178798269-178815370	Loss	1	─		
				178798269-179550475	Gain	3			
2	106062109-106063373	Gain	20	106062109-106064917	Loss	7	─		
3	65705932- 65951800	Loss, gain	27, 29	65705932-66065643	Gain	50	─		
4	52424614-52612016	Gain	20	52194368-52612016	Gain	1	─		
5	37840041-37916448	Loss	16	37840041-37916448	Loss	5	37878731-37954286	Loss, gain	9, 8
5	88243192-88258862	Gain	57	88243192-88349479	Gain	27	─		
6	26086675-26126581	Gain	42	26102028-26126581	Gain	1	─		
6	72032729-72607543	Loss, gain	136, 11	71956823-72607543	Loss	71	71977669-73390784	Loss, gain	5, 1
7	31406445-31520977	Loss	17	31406445-31529855	Loss	10	─		
7	52610482-52677786	Gain	37	52610482-52677786	Gain	39	52617898-52737112	Loss, gain	5 ,4
7	73083306-73197149	Loss, gain	14, 3	73083306-73197149	Loss	3	73056824-73699458	Loss, gain	14, 14
8	4280605-4621044	Loss, gain	37, 40	4183178-4430473	Loss	2	4381193-4648028	Gain	2
9	31574454-31574969	Loss	4	─	─	─	─		
10	674485-1271225	Loss, gain	1, 6	674485-1141923	Gain	10	─		
11	54645681-54812394	Loss	47	54640169-54812394	Loss	1	─		
12	12524489-14777981	Loss, gain	108, 116	12524489-13488187	Loss	32	12314908-15866355	Loss, gain	12, 13
				13945011-14777981	Loss	114			
18	11660478-12399073	Gain	2	─	─	─	─		
20	32059082-32210308	Loss, gain	24, 3	32059082-32250493	Gain	9	32031829-32033708	Loss	1
24	32416012-32628728	Loss, gain	1, 11	32416012-32628728	Gain	20	─		
25	26318531-27125754	Loss, gain	137, 4	26318531-26942120	Loss	99	26274719-26980393	Loss, gain	2, 2

Overlapping CNV regions from each study are shown in base pairs. Copy number (CN) and number of samples are displayed for all studies.

CGH analyses showed less concordance with our detection events. It was proposed that the results of CGH and SNP arrays were generally not easily comparable [1]. In particular, the CNV-study designs in horses were different, as CGH analyses only targeted exons, while the SNP arrays covered the whole genome. Furthermore, our analysis was performed on a considerably higher number of samples that might allow a more general view on the distribution of CNVs in the horse population. Comparisons between the CNV regions detected by CGH analyses and those identified by the three SNP array detection algorithms individually revealed a relatively high overlap with the detection results of QuantiSNP (25%), less consistency with the PennCNV results (7%) and an extremely low overlap with CNVPartition (3%, Table 3). Despite the relatively low overlap in total, PennCNV showed concordance up to 100% in some chromosomal regions. These comparisons confirm the assumption that the prediction accuracy of CNVPartition seems to be relatively poor due to the high rates of missed events [11]. We suppose that this was the reason for the considerably high number of detected CNVs by individual programs in comparison with the number of overlapping detection events. A closer look at each algorithm showed that PennCNV and QuantiSNP provided a similar distribution of CNVs over the chromosomes and had a considerably higher number of detection events in comparison with CNVPartition. PennCNV and QuantiSNP are both hidden Markov model (HMM) based algorithms that use the log R ratio and B allele frequency independently (QuantiSNP) or in combination (PennCNV) [25]. Comparative analyses of CNV detection methods for SNP arrays confirmed that PennCNV and QuantiSNP had a large overlap of detection events [11]. A study of bladder cancer in human which used a study design similar to ours suggested PennCNV and QuantiSNP to be a more reliable method for the detection of CNVs than CNVPartition [26]. QuantiSNP was even assumed to be the best of these three methods and also outperformed the other algorithms [25].

**Table 3:** Comparison between 754 CNV regions detected by CGH analyses and the SNP array detection results of CNVPartition, PennCNV and QuantiSNP

**ECA**	**Number of CNV regions detected CGH analysis, Doan *et al. *(n)**	**CNV regions detected by CNVPartition (%)**	**CNV regions detected by PennCNV (%)**	**CNV regions detected by QuantiSNP (%)**
1	67	11	11	24
2	31	3	3	35
3	35	0	3	29
4	27	7	11	15
5	44	0	0	11
6	29	7	4	14
7	50	2	4	12
8	24	4	4	38
9	17	0	6	29
10	31	0	0	48
11	39	3	5	28
12	15	7	13	47
13	22	0	55	55
14	32	0	3	34
15	33	0	0	3
16	23	0	0	13
17	22	0	5	14
18	27	0	0	19
19	14	0	0	0
20	47	4	9	17
21	18	0	0	33
22	21	0	0	29
23	17	6	6	24
24	10	0	0	0
25	14	29	29	43
26	4	0	0	0
27	7	0	57	86
28	5	0	100	80
29	6	17	0	17
30	14	0	0	50
31	9	0	0	0
total	754	3	7	25

All CNV regions that show an overlap of at least 50% with the CNV region detected by CGH are displayed for each chromosome. The highest overlap can be seen with QuantiSNP.

### CNV sharing among breeds

After filtering out samples with low call rate and quality features we were able to detect CNVs in 717 horses. The total number of detected (identical and not identical) CNVs was 4013 for these horses of different breeds. Due to the choice of unrelated horses, we supposed this number of CNVs gives a good view on the distribution of CNVs in the horse population with the restriction that different numbers of samples were available per breed. Comparative analysis of all three algorithms revealed 536 CNVs that could be detected in all programs for the same breed. These CNVs were found in Arabian, Hanoverian, Holsteiner, Lusitano, Maremanno, Oldenburg, Thoroughbred, Westphalian (Table 4). With regard to the individuals we could confirm 21 CNVs that were detected in the same horse using all algorithms. Theses CNVs ranged in size from 516 to 862,853 bp and showed an average size of 368,720 bp. Further 29 CNVs showed an overlap among different breeds (Table 5).

**Table 4:** Distribution of detected CNVs among different breeds

**Breed**	**Average height**	**Number of animals**	**Number of detected CNVs**	**Number of CNVs derived from the 50 CNVs of the intersection**	**Number of animals (n)**	**Number of CNVs derived from the inter-section detected in the same horse**	**Number of animals (n)**
Anglo-Arabian	165	1	1	0	0	0	0
Arabian	148	87	713	16	15	1	1
Brandenburger	168	1	1	0	0	0	0
Hanoverian	168	458	2508	442	262	11	10
Holsteiner	168	6	51	2	2	2	2
Lusitano	160	47	263	40	28	0	0
Maremanno	160	44	177	17	13	1	1
Oldenburg	168	11	54	4	4	0	0
Rhenish-German Cold-Blood	165	2	9	0	0	0	0
Rhinelander horse	168	11	49	0	0	0	0
German Riding Pony	148	1	7	0	0	0	0
Selle Francais	165	1	2	0	0	0	0
Thoroughbred	163	13	47	2	2	1	1
Trakehner	168	3	13	0	0	0	0
Westphalian	168	29	112	13	12	2	2
Zweibrücker	168	1	6	0	0	0	0
Total	─	717	4013	536	338	21	17

The total number of CNVs, the number of CNVs derived from the 50 CNVs of all three detection programs (intersection) and the number of animals which showed these CNVs in at least one algorithm is displayed. The last two columns show the more stringent category with the number of CNVs that were detected in the same horse in all three algorithms and with the number of horses under these conditions.

**Table 5:** Number of CNVs derived from the 50 calls of all three detection algorithms that overlap among different breeds

	**Han**	**Old**	**Wes**	**Hol**	**Bran**	**RPon**	**Trak**	**TB-H**	**AV**	**Lus**	**Mar**	**RDK**	**RHD**
**Han**													
**Old**	8												
**Wes**	7	4											
**Hol**	8	1	1										
**Bran**	1	1	0	0									
**RPon**	1	1	1	1	0								
**Trak**	3	1	1	1	0	1							
**TB-H**	4	0	2	0	0	0	1						
**AV**	12	6	4	2	1	1	1	0					
**Lus**	10	5	3	2	1	1	2	1	6				
**Mar**	10	4	3	3	1	1	1	1	5	8			
**RDK**	3	2	2	2	0	1	1	0	1	2	2		
**RHD**	4	3	4	1	0	1	1	1	3	1	2	1	

On the whole 13 breeds shared their CNV with another breed (*Han* Hanoverian, *Lus* Lusitano, *Mar* Maremanno, *Old* Oldenburg, *RDK* Rhenish-German Cold-Blood, *Wes* Westphalian, *Hol* Holsteiner, *TB-H* Thoroughbred, *AV* Arabian, *RHD* Rhinelander horse, *Bran* Brandenburger, *Trak* Trakehner, *RPon* German Riding Pony).

Comparisons between the 50 CNVs derived from all three detection algorithms showed that the largest number of CNVs occurred in the Hanoverians, supposably due to the largest number of samples used in this study. On the whole 18 Hanoverian specific CNVs could be detected. Two of these showed gains and losses in different horses (Additional file 4). In general, only one CNV derived from the comparative analysis of three detection algorithms showed losses and gains among different breeds. This CNV was located on ECA1 at 155.49-155.55 Mb and showed losses in Hanoverian and Lusitano and gains in Holsteiner and Thoroughbred horses.

Furthermore a relatively high number of CNVs shared with the Hanoverian could also be found in Oldenburg, Westphalian and Holsteiner, presumably due to the close relationship among these breeds. A CNV overlap between Arabian, Lusitano and Maremanno horses could be explained by the strong influence of the Arabian and Lusitano bloodlines on the Maremanno breed. In addition, we investigated the distribution of CNVs in two Przewalski horses and detected on the whole 10 CNVs which could not be identified by all three detection algorithms but showed an overlap with different breeds (Additional file 5). We assume that these CNVs might be conserved for a long time and passed on during the domestication of the horse.

Evaluations of the individual horses showed that most horses shared their CNVs with at least another animal according to previous observations [5]. Comparing the average size of CNVs detected in more than one animal (397,708 bp) with the size of CNVs not shared by a second horse (250,773 bp) confirmed the suggestion that CNV sharing among horses is correlated with CNV length as larger CNVs are more likely to be shared [5].

### Analysis of genes within CNV regions

Analysis of genomic regions of 50 CNVs, derived from comparative analysis of CNV detection algorithms, revealed 153 different genes within 45 CNVs. In five CNV regions we were not able to find any functional gene. The major category of genes consisted of olfactory receptor (OR) genes (66.7%), a group of genes which is known to be significantly enriched in CNV regions in human, cattle, pig and rat [6,7,27,28]. It was supposed that the overrepresentation of OR genes is not a result of positive selection but of the frequent appearance of these genes in segmentally duplicated regions [27]. These regions are known to be more susceptible to CNVs while genomic regions with dosage-sensitive genes usually show a low number of CNVs [3,27].

Functional analysis was performed using human orthologs of the horse genes due to the poor annotation of the horse genome. The results of the Database for Annotation, Visualization and Integrated Discovery (DAVID) 6.7 showed an enrichment of 131 genes involved in sensory perception, signal transduction and cellular components (Additional file 6). The highest P-values could be observed for the classifications olfaction (P = 4.20x10^-142^), olfactory receptor (P = 2.40x10^-134^) and sensory perception of smell (P = 6.90x10^-132^). Further analyses with the PANTHER classification system revealed statistically over- and underrepresented biological processes and confirmed the overrepresentation of genes involved in sensory perception (P = 4.53x10^-01^) and signal transduction as well as response to stimuli (Table 6). Processes involved in cell cycle, transcription and metabolism were underrepresented.

**Table 6:** Gene ontology analysis of significantly over- and underrepresented genes

	**Number of reference genes (Homo sapiens)**	**Number of genes in CNV regions (Horse)**	**Expected**	**Raw p-value**	**Bonferroni corrected p-value for multiple testing**
**Biological process-overrepresented genes**					
Response to stress	586	17	3.60	1.34E-07	2.28E-05
G-protein coupled receptor protein signaling pathway	1042	22	6.41	3.99E-07	6.82E-05
Muscle contraction	613	17	3.77	2.50E-07	4.28E-05
Sensory perception	864	13	5.31	2.65E-03	4.53E-01
Phagocytosis	101	6	0.62	4.25E-05	7.28E-03
Macrophage activation	352	8	2.16	1.58E-03	2.71E-01
Response to stimulus	1921	22	11.81	3.19E-03	5.46E-01
Cyclic nucleotide metabolic process	109	6	0.67	6.46E-05	1.10E-02
Nerve-nerve synaptic transmission	122	7	0.75	1.20E-05	2.06E-03
Synaptic transmission	756	12	4.65	2.51E-03	4.30E-01
**Biological process-underrepresented genes**					
Cell cycle	1867	3	11.48	2.47E-03	4.22E-01
Transcription from RNA polymerase II promoter	2343	2	14.41	3.30E-05	5.64E-03
Regulation of transcription from RNA polymerase II promoter	1780	1	10.95	1.37E-04	2.33E-02
Transcription	2353	2	14.47	3.11E-05	5.31E-03
Primary metabolic process	8037	16	49.43	3.67E-11	6.28E-09
Nucleobase, nucleoside, nucleotide and nucleic acid metabolic process	3987	8	24.52	2.75E-05	4.70E-03
Cellular component organization	1496	2	9.20	4.22E-03	7.21E-01
Mesoderm development	1621	2	9.97	2.14E-03	3.67E-01
Proteolysis	1230	1	7.56	3.69E-03	6.30E-01
Metabolic process	8351	17	51.36	1.78E-11	3.04E-09
Protein metabolic process	3295	3	20.26	6.38E-07	1.09E-04

The software PANTHER was used for the evaluation of 45 CNV regions detected by all three algorithms. In five CNV regions we could not detect any genes for evaluation.

Analysis of the molecular functions demonstrated significantly overrepresented processes concerning receptor activities and particularly underrepresented catalytic and hydrolase activities (Additional file 7). The PANTHER protein class analysis showed a comparatively reduced number of nucleic acid binding, transferase and hydrolase proteins. Our results suggest that CNV regions are not randomly distributed among the genes of the horse genome, but they harbour certain genes overrepresented in those regions while other genes involved in specific biological processes are underrepresented. Similar distributions were observed in cattle and in CGH array analyses of 16 horses, showing sensory perception and signal transduction to be the primary processes affected by CNVs [5,7].

Furthermore, an enrichment of genes involved in metabolic processes was also observed in CGH analyses of horses [5]. Our study revealed an impoverishment of CNVs in regions of genes responsible for a metabolic process. This term is defined by PANTHER as chemical reactions and pathways by which living organisms transform chemical substances. A closer look at our analysis of metabolic pathways shows that primary metabolic processes, protein metabolic processes, nucleobase, nucleoside, nucleotide and nucleic acid metabolic processes were underrepresented while the cyclic nucleotide metabolic process was overrepresented. We assume that metabolic process is a very general term and has to be interpreted in context of more specific ontology terms for metabolic processes. Furthermore, we suppose that the high number of horses used in our study in comparison with previous CGH analysis might give a more general view on the distribution of enriched and impoverished genes in CNV regions. Similar to our results, human CNV studies revealed a significant underrepresentation of genes involved in nucleic acid metabolism in deletion regions [29]. Furthermore, enriched gene categories concerning sensory perceptions of smell, chemical stimuli, smell and taste as well as neurophysiological processes, brain development, immune responses and external biotic stimuli could be shown in human [3,30,31]. It was assumed that the underrepresentation of genes occurs due to the strong selective pressure for genes involved in important processes like transcriptional regulation and development [32].

### Association analysis with body size

GWAS for body size was performed for each algorithm on basis of its CNV detection results and for the intersection of all three detection programs. A highly genome-wide significant peak on ECA1 could be shown for the data generated by PennCNV (P = 0.006) and QuantiSNP (P = 0.010). Analysis of the intersection of 50 CNVs revealed a P-value at the threshold of significance (P = 0.057) presumably influenced by CNVPartition which did not reveal any significant association. The associated CNVs were deletions in the region of 156 Mb (Table 7). They were located in the area of the candidate genes *olfactory receptor 4, subfamily K5 (OR4K5), subfamily K2 (OR4K2), subfamily N2 (OR4N2)* and *subfamily M1 (OR4M1)*. GWAS for copy number variation in human revealed the syntenic region of 40.25-40.39 Mb to be significantly associated with stature [20]. It was proposed that individuals with short stature show an excess of lower-frequency deletions [20]. Our analysis revealed the heterozygous deletions on ECA1 to be associated with larger sized horses.

**Table 7:** Genome-wide associated CNVs for body size in horses

**ECA**	**Start**	**End**	**Genome-wide (chromosome-wide) P value PennCNV**	**Genome-wide (chromosome-wide) P value QuantiSNP**	**Genome-wide (chromosome-wide) P value CNVPartition**	**Genome-wide (chromosome-wide) P value intersection of three detection programs**	**Copy number**	**Number of animals**
1	156,012,982	156,870,455	0.006 (0.001)				1	111
1	156,657882	156,870,455		0.010 (0.003)			1	80
1	156,657,882	156,818,876				0.057 (0.018)	1	132
8	4,430,473	4,430,473	0.001 (0.001)			0.081 (0.023)	1	12/61
8	4,579,478	4,621,044		0.0002 (0.0002)			1	33
9	29,889,627	29,892,897		0.006 (0.001)			0, 1	37

The chromosome-wide (EMP1; in brackets) and genome-wide corrected significant P-values (EMP2) of the associated CNV regions detected by PLINK for the different algorithms and the number of animals showing a CNV in this region are given. For the intersection of three detection programs the P-value at the threshold of significance is displayed. CNVPartition did not show any significant association.

A second CNV region, with the highest association for body size (P = 0.0002), was located on ECA8 at 4.43-4.62 Mb. All these heterozygous deletions could be found in larger sized horses and were absent in smaller horse breeds. The peak region harboured the candidate genes *immunoglobulin lambda variable 3–19 (IGLV3-19), variable 3–27 (IGLV3-27)* and *variable 2–11 (IGLV2-11)* which all belong to the immunoglobulin lambda light chain variable gene cluster, which is important for immunoglobulin structure [33].

A third candidate region could be detected by QuantiSNP on ECA9 (P = 0.006) at 29.89 Mb. This CNV showed deletions for 37 larger sized warmblood horses. The neighbouring candidate genes *opioid receptor, kappa 1 (OPRK1) and ATPase, H + transporting, lysosomal, 50/57-KD, V1 subunit H (ATP6V1H)* have been shown to be associated with body conformation in pigs [34]. Vacular-type H + ATPase proton pump is a complex located in the ruffled border plasma membrane of bone-resorbing osteoclasts and is important for bone resorption. Mutations or deletions in V-ATPase subunits encoding genes have been shown to decrease resorptive activity in bones [35-37]. Further candidate genes in the region of the associated CNV are the *v-yes-1 yamaguchi sarcoma viral related oncogene homolog (LYN), the trimethylguanosine synthase, S. cervisae, homolog of (TGS1) and the pleiomorphic adenoma gene 1 (PLAG1)*. Genome scans for sequence variants in human revealed these genes to be in the region of SNPs with the strongest correlation for height in a meta-analysis [38]. The candidate gene *PLAG1* is known to be involved in developmental processes [39,40]. Its influence on bovine stature could be shown by variants modulating the expression of a chromosome domain encompassing *PLAG1*[39]. A targeted disruption of *PLAG1* in mice caused early growth retardation which was maintained throughout adult life [40].

Our results showed that body size in horses is mainly associated with homozygous or heterozygous deleted CNVs on ECA1, ECA8 and ECA9. These regions do not correspond with the region of the potential main regulator *LCORL* or further associated SNPs or CNVs for body size in horses [5,21-23]. We assume that the associated CNVs detected in our analysis might represent additional regulators in the complex process for the determination of height.

The association of the CNV regions on ECA1 and ECA8 could be confirmed by two detection algorithms, while the peak region on ECA9 was exclusively detected by QuantiSNP. The Illumina-based algorithm CNVPartition did not reveal any association for body size although the associated CNVs on ECA1 and ECA8 could be detected by all three programs. We assume that the generally low number of detected CNVs in only few horses were the reason for the missing association. For this reason we propose that CNVPartition did not allow an appropriate association analysis due to the high false negative rates. This correlates well with our analysis for the detection accuracy of each algorithm. The results demonstrate certain limitations that have to be considered for the use of multiple predictions. It was proposed that analyses of different detection algorithms have to be taken with care due to false negative events but are also useful to avoid false positives caused by noisy data and call attention to discrepancies in the data [11,15]. PennCNV and QuantiSNP were shown to be more reliable in detecting CNVs than CNVPartition [26]. Our analysis confirmed this suggestion and showed that not only the number of compared CNV detection events but also the choice of the program is important for an effective analysis.

### qPCR validation

**Figure 3:**
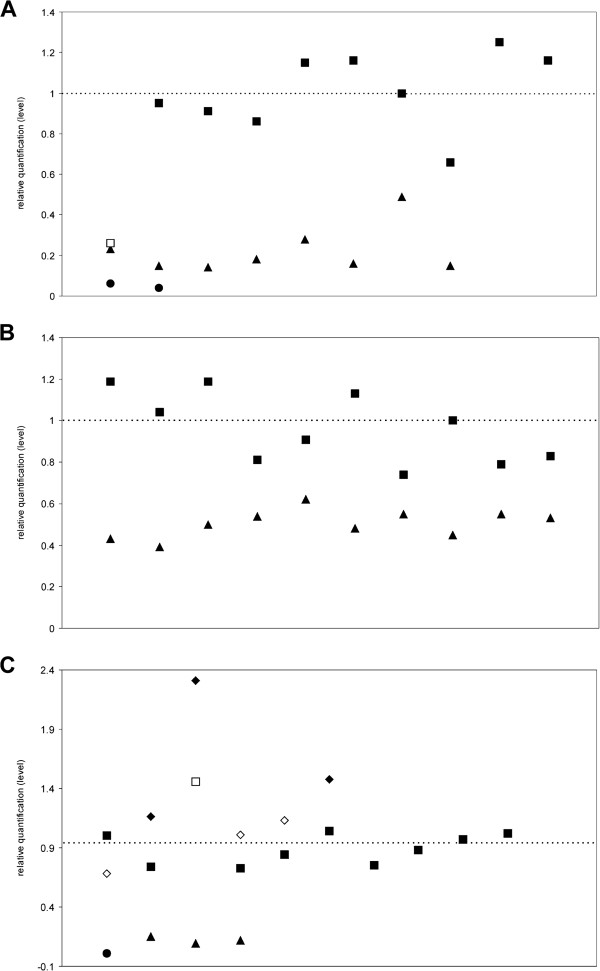
**Verification of detected CNVs by qPCR.** The horizontal line represents the relative quantification level 1. Two copies are shown by squares, one copy by triangles, zero copies by circles and three copies by rhombus. (**A**) The CNV region on ECA1 at 155.63 Mb was validated in nineteen horses (solid symbols). One horse showed a homozygous deletion instead of two copies (unfilled symbol). (**B**) The CNV region on ECA1 at 156.69 Mb was validated in all twenty horses (solid symbols). (**C**) The CNV region on ECA8 at 4.50 Mb was validated in sixteen horses (solid symbols). Four horses showed different copies as detected by SNP chip analysis (unfilled symbols).

For validation of detected CNVs we performed qPCR analysis in two CNVs on ECA1 and one CNV on ECA8 for twenty horses each (Additional file 8). We chose three CNV regions that were detected by all three programs of which two were associated with body size and one was also validated in horses of previous analysis [5]. All three CNV regions could be confirmed by qPCR (Figure 3). The rate for the accurate copy number detection of at least one algorithm was 95% for *Olfr1284 (ENSECAG00000006791)* on ECA1 and 80% for *IGLV3-32 (ENSECAG00000005113)* on ECA8. For the CNV region on ECA1 at the candidate gene *OR4K2 (ENSECAG00000006318)* all horses could be validated. The results show that the analysed CNVs could be validated although few horses did not show the copy number detected by SNP chip analysis. Similar analyses in pigs revealed accurate discovery rates of 71% [6].

Considering the individual programs, the false negative discovery rate of CNVPartition was particularly high, while QuantiSNP showed the lowest false negative discovery rates in all three CNV regions (Additional file 9). We assume that these results underline our comparative analyses for the detection algorithms showing QuantiSNP to be the most reliable and accurate program.

In addition to this validation, we compared CNVs of previous CGH analyses that were validated by qPCR with our detection results. On the whole, four previously verified CNVs could be confirmed in our analysis. One of these CNVs was rare in our population and could only be detected by QuantiSNP and CNVPartition, further three CNVs could be found in a considerably higher number of horses by QuantiSNP and also by PennCNV in contrast to CNVPartition (Additional file 10). This comparison helps to verify our CNV detection results in these regions and confirms the assumption that there are significant differences in the detection abilities of the analysed algorithms. We conclude that a validation of detected CNVs is crucial especially for further analyses of associated regions, and helps to evaluate the accuracy of the detection results by specific programs.

## Conclusions

The aim of our analysis was to investigate three different algorithms for the detection of CNVs in horses and to find associated CNVs for the regulation of body size. Comparative analysis of the detection programs in 717 horses revealed 50 CNVs with an average size of 388,892 bp. Functional analysis of genes located in these CNVs confirmed the high amount of OR genes (66.7%) and showed an overrepresentation of genes involved in sensory perception, signal transduction and cellular components while cell cycle, transcription and metabolic processes were underrepresented.

We conclude that in general the creation of an intersection of three CNV-programs is useful to increase the accuracy of CNV detection and to reduce the number of false positive results. Nevertheless, the comparison between these programs also provides a strong restriction and a higher number of false negative results which is highly dependent on the choice of the detection tool. We recommend taking advantage of the different algorithms used in detection programs and to perform multiple predictions in a first step. Such an analysis will show possible limitations and establish suitable algorithms for further evaluations. In our study the combined use of PennCNV and QuantiSNP was most effective for the accurate detection of CNVs. GWAS for the CNV detection results of these algorithms identified for body size three homozygous or heterozygous deleted CNV regions associated with larger sized horses on ECA1, ECA8 and ECA9. Two of these regions were analysed by qPCR and could be validated. We conclude that a comparative CNV analysis is a useful approach as it reveals the limitations of individual programs and helps to estimate the reliability of the detection results.

## Methods

### Ethic statement

All animal work has been conducted according to the national and international guidelines for animal welfare. The sampling was approved by the Lower Saxony state veterinary office Niedersächsisches Landesamt für Verbraucherschutz und Lebensmittelsicherheit, Oldenburg, Germany (registration numbers 509c-42502-01A60, 02A-138 and 07A-482).

### Sample preparation

EDTA-blood samples of 17 different breeds were collected including 148 Arabian, one Anglo-Arabian, two Brandenburger, 514 Hanoverian, nine Holsteiner, 13 Oldenburg, five Trakehner, 35 Westphalian, one Selle Francais, one German Riding Pony, 47 Lusitano, 48 Maremanno, two Przewalski, 12 Rhinelander horse, two Rhenish-German Cold-Blood, 14 Thoroughbred and one Zweibrücker. The horses were chosen out of different breeding lines to provide unrelated horses as far as possible. For genotyping we isolated genomic DNA using standard methods with RBC (Red Blood Cell) lysis buffer and SE (sodium EDTA) buffer. The DNA concentration of the samples was adjusted to 50 ng/μl using the Nanodrop ND-1000 (Peqlab Biotechnology, Erlangen, Germany) and quality control was performed by gel electrophoresis using 1% agarose gels (peqGold Universal Agarose, Peqlab Biotechnologie, Erlangen, Germany). All 854 animals were genotyped using the Illumina equine SNP50 BeadChip (Illumina, San Diego, USA) including 54,602 SNPs.

### Data analysis

To provide reliable results, quality control was performed in a first step by the choice of DNA samples with high qualities and the determination of a call rate >90% for animals and SNPs. Further criteria for the exclusion of noisy data were chosen individually for each algorithm. Primary visualisation and quality control of the CNV data was performed with the GenomeStudio software (Illumina). Data files containing SNP name, chromosome, position, B-allele frequency and log R ratio were exported as a unique file and separated using the split option in PennCNV package. Analysis of the autosomal regions for CNV was done in three different programs. PennCNV was run according to default criteria (Illumina) [17] using the command line detect_cnv.pl –hmm example.hmm –pfb horse909Tiere.pfb –minsnp 3 –lastchr 31 –test –tabout –coord –conf –listfile list.txt –out penncnv1.txt and probe counts less than three were omitted afterwards. Quality control was performed employing standard exclusions of the Log Ratio (SD (LRR)) >0.3 and the GC-content caused fluctuation of signal intensity (│GCWF│) >0.02. An individual-call mode was used for all samples. Then we used QuantiSNP 2.0, a program based on an Objective Bayes Hidden-Markov Model (HMM) [24], for CNV analysis. The detection was performed by the command: quantisnp2 –outdir horse –logfile 909Pferde –chr [1:31] –chrx 32 –beadstudio-files 909Pferde.txt. After CNV detection the minimum probe count of three, a threshold for the Log Bayes Factor of less than ten was set. The GenomeStudio plug-in cnvPartition 3.1.6 was run by default criteria including a confidence threshold of 35, a minimum homozygous region size of 1,000,000 a minimum probe count of 3 and GcWaveAdjustLRR.

GWAS for the CNV detection results of each individual programme and the intersection of PennCNV, QuantiSNP and cnvPartition was performed using PLINK version 1.07 (http://pngu.mgh.harvard.edu/purcell/plink/) [41] and SAS/Genetics, version 9.3 (2013) in a quantitative trait analysis. A mixed linear model (MLM) was employed to control data for stratification. Size ranges for every breed were estimated and results were averaged as it was shown in our previous analysis for body size (Table 4) [23]. The chromosomal enrichment was also accounted with SAS/Genetics for each algorithm similar to the enrichment analysis in the CGH study [5] by merging overlapping CNVs on basis of the SNP map of 48860 SNPs to CNV regions. The number of SNPs covered by CNV regions was divided by the length of the chromosome. The whole-genome coverage of CNV regions was used as standard after dividing by the total length of the autosomes. Significant chromosomal enrichment occurred when the enrichment of the genome was lower than the enrichment of the chromosome.

### Ontology analysis

Genes located in the CNV regions of the intersection of all three programs were identified using NCBI MapViewer http://ftp.ncbi.nih.gov/genomes/MapView/ Equus_caballus. Due to the insufficient annotation of the horse genome, we determined the human orthologs for these genes by NCBI gene http://www.ncbi.nlm.nih.gov/gene and HomoloGene http://www.ncbi.nlm.nih.gov/homologene to perform ontology analysis. The Database for Annotation, Visualization and Integrated Discovery (DAVID) 6.7 (http://david.abcc.ncifcrf.gov/home.jsp) was used for functional annotation of genes in CNV regions [42,43]. The functional annotation chart was run with a count threshold of 2, an EASE Score threshold of 0.1 and the Bonferoni correction. Results were grouped in functional groups. Further ontology analysis was performed using the PANTHER (Protein ANalysis THrough Evolutionary Relationships, version 8.0) classification system http://www.pantherdb.org/ for the classification of genes by their molecular function, biological process and cellular component with default Bonferoni correction [44].

### qPCR validation

Validation of three different genomic regions harbouring CNVs was performed by quantitative real-time (qRT)-PCR on the ABI7300 sequence detection system (Applied Biosystems by Life Technologies, Darmstadt, Germany). On the whole eighteen warmblood horses and two Arabians were analysed. DNA was isolated from blood samples in the same way as it was done for SNP chip analysis. The DNA concentration of the samples was adjusted to 10 ng/μl using the Nanodrop ND-1000 (Peqlab Biotechnology).

Primer pairs and probes were designed using Primer express 3.0 (Applied Biosystems) and Primer3 (http://frodo.wi.mit.edu/primer3/). *Glyceraldehyde-3-phosphate dehydrogenase* (*GAPDH*) on ECA6 was used as reference gene according to previous CNV analyses in horses [5]. Reactions were assembled in a final volume of 12 μl containing 2.0 μl gDNA, 1.0 μl reverse and 1.0 μl forward primes (10 pmol; Additional file 6), 0.25 μl VIC-labeled TaqMan probe for CNV regions (6 nmol; 100 μM) and FAM-labeled TaqMan probe for *GAPDH*, 1.68 μl nuclease free water and 6 μl Maxima Probe qPCR master mix 2x supplemented by 0.07 μl ROX Solution (50 μM; Fermentas Life Sciences, St. Leon-Rot, Germany). Quantitative real-time (qRT)-PCR was performed for 10 minutes (min) at 95°C followed by 40 cycles at 95°C for 15 seconds and 60°C for 1 min. Analysis was done for every sample two times and the average value was used for further calculations. One sample without copy number variations in the analysed regions was used as reference. For the relative quantification of CNVs we used the 2^-ΔΔCt^ method [45]. The ΔCT values of the test samples were subtracted from the ΔCT of the reference sample and put in the formula 2^-ΔΔCt^.

## Abbreviations

CGH: Comparative genomic hybridization; CNV: Copy number variant; HMM: Hidden Markov model; OR4K5: *Olfactory receptor 4, subfamily K5*; OR4K2: *Olfactory receptor 4,subfamily K2*; OR4N2: *Olfactory receptor 4, subfamily N2*; OR4M1: *Olfactory receptor 4 ,subfamily M1*; IGLV3-19: *Immunoglobulin lambda variable 3–19*; IGLV3-27: *Immunoglobulin lambda variable 3–27*; IGLV2-11: *Immunoglobulin lambda variable 2–11*; LYN: *v-yes-1 yamaguchi sarcoma viral related oncogene homolog*; PCR: Polymerase chain reaction; PLAG1: *Pleomorphic adenoma gene 1*; qPCR: Real-time-quantitative-PCR; TGS1: *The trimethylguanosine synthase, S. cervisae, homolog of.*

## Competing interests

The authors declare that they have no competing interests.

## Authors’ contributions

JM, OD designed the study, carried out the experiments and data analysis, drafted and finalized the manuscript. OD, MSL, ACM, MF and MS provided samples and data for the study and helped to revise the manuscript. UP took part in the design of qPCR analysis. All authors read and approved the final manuscript.

## Supplementary Material

Additional file 1 **CNVs detected by CNVPartition.** The table summarises start and end positions of detected CNVs, their size, copy number, number of samples and genes located in CNV regions. Text in PDF format. Click here for file

Additional file 2 **CNVs detected by PennCNV.** The table summarises start and end positions of detected CNVs, their size, copy number, number of samples and genes located in CNV regions. Text in PDF format. Click here for file

Additional file 3 **CNVs detected by QuantiSNP.** The table summarises start and end positions of detected CNVs, their size, copy number, number of samples and genes located in CNV regions. Text in PDF format. Click here for file

Additional file 4 **Comparative analysis of three CNV detection algorithms.** The table shows 50 CNVs derived from three detection algorithms, their position, size, copy number per sample and breed (HAN: Hanoverian; LUS: Lusitano; MAR: Maremanno; OLD: Oldenburg; RDK: Rhenish-German Cold-Blood; WES: Westphalian; HOL: Holsteiner; TB-H: Thoroughbred; AV: Arabian; RHD: Rhinelander horse; PRZ: Przewalski; BRAN: Brandenburger; TRAK: Trakehner; RPON: German Riding Pony). Text in PDF format. Click here for file

Additional file 5 **Display of CNVs detected in two Przewalski horses by comparative analysis of three algorithms.** The overlap of CNVs with different breeds is shown. Click here for file

Additional file 6 **Functional annotation analysis of enriched genes derived from 45 CNV regions using DAVID 6.7.** In five CNV regions we could not detect any genes for evaluation. Text in DOC format. Click here for file

Additional file 7 **Gene ontology analysis of significantly over- and underrepresented genes involved in molecular functions and protein classes.** The software PANTHER was used for the evaluation of 45 CNV regions detected by all three algorithms. Click here for file

Additional file 8 **Genomic regions analysed for copy number variants (CNVs) by real-time quantitative RT-qPCR.** Primer sequences, their position, product size, annealing temperature and TaqMan probes are shown. Text in DOC format. Click here for file

Additional file 9 **Copy number detection accuracy of three SNP array algorithms.** The deleted (0, 1) or duplicated (3) copy numbers validated by qPCR are compared with the detection results of CNVPartition, PennCNV and QuantiSNP. Reference samples with the copy number 2 are not displayed as they were selected specifically for two copies in all three programs. Click here for file

Additional file 10 **Comparison of CNVs validated by qPCR in CGH analysis (Doan *et al.*) with CNVs detected by CNVPartition, PennCNV and QuantiSNP.** The number of samples detected by QuantiSNP is considerably high. Click here for file
