# Supplementary material for: Analysis of copy number variants by three detection algorithms and their association with body size in horses
Source: BMC Genomics. 2013 Jul 18;14:487. doi: 10.1186/1471-2164-14-487 (PMC3720552; doi:10.1186/1471-2164-14-487)
Supplement: Additional file 1 — CNVs detected by CNVPartition. The table summarises start and end positions of detected CNVs, their size, copy number, number of samples and genes located in CNV regions. Text in PDF format. [file 1471-2164-14-487-S1.pdf]

Chrom	Start	End	Size	Cn	Samples (n)	Genes
01	109374964	109674458	299495	1	1	LOC100060875
01	136563078	136816131	253054	4	1	UNC13C
01	155325635	155652475	326841	1	1	LOC100072099,LOC100058220,LOC100146794,LOC100072083,LOC100072126,LOC100072136,LOC100072146,LOC100072141,LOC100072149,LOC100058263,LOC100147374,LOC100072107,LOC100072089,LOC100072129,LOC100072110,LOC100072120,LOC100072092,LOC100072117,LOC100072079,LOC100072112,LOC100058312
01	155325635	155656642	331008	0	3	LOC100072099,LOC100058220,LOC100146794,LOC100072083,LOC100072126,LOC100072136,LOC100072146,LOC100072141,LOC100072149,LOC100058263,LOC100147374,LOC100072107,LOC100072089,LOC100072129,LOC100072110,LOC100072120,LOC100072092,LOC100072117,LOC100072079,LOC100072112,LOC100058312
01	155487276	155593582	106307	0	1	LOC100072126,LOC100072129,LOC100072120,LOC100072117,LOC100072112,LOC100058312
01	155487276	155652475	165200	0,1	2,8	LOC100072126,LOC100072136,LOC100072146,LOC100072141,LOC100072149,LOC100072129,LOC100072120,LOC100072117,LOC100072112,LOC100058312
01	155487276	155656642	169367	0,1	18,7	LOC100072126,LOC100072136,LOC100072146,LOC100072141,LOC100072149,LOC100072129,LOC100072120,LOC100072117,LOC100072112,LOC100058312
01	155487276	155743552	256277	0	3	LOC100072126,LOC100072136,LOC100072146,LOC100072141,LOC100072149,LOC100072129,LOC100072120,LOC100072117,LOC100072158,LOC100072112,LOC100072163,LOC100058312
01	155795029	156012981	217953	1	1	LOC100072179,LOC100072215,LOC100058354,LOC100072192,LOC100072202,LOC100072184,LOC100072227,LOC100072195,LOC100072221,LOC100072206,LOC100072211,LOC100058393
01	155795029	156657881	862853	0,1	4,5	LOC100072296,LOC100072258,LOC100072414,LOC100072179,LOC100072447,LOC100072426,LOC100072215,LOC100072251,LOC100058354,LOC100072237,LOC100072429,LOC100072368,LOC100072386,LOC100072192,LOC100072351,LOC100072271,LOC100072432,LOC100072275,LOC100072202,LOC100072265,LOC100072279,LOC100072306,LOC100072261,LOC100072184,LOC100072398,LOC100072342,LOC100072288,LOC100072317,LOC100072300,LOC100072380,LOC100072227,LOC100072195,LOC100072365,LOC100072443,LOC100058435,LOC100072402,LOC100072282,LOC100072290,LOC100072423,LOC100072320,LOC100072338,LOC100072221,LOC100072392,LOC100072354,LOC100072404,LOC100146987,LOC100072206,LOC100072211,LOC100072325,LOC100058393,LOC100072375,LOC100072345,LOC100072408,LOC100072254,LOC100072330,LOC100072335,LOC100072245
01	155795652	156425245	629594	1	2	LOC100072296,LOC100072258,LOC100072179,LOC100072215,LOC100072251,LOC100058354,LOC100072237,LOC100072368,LOC100072192,LOC100072351,LOC100072271,LOC100072275,LOC100072202,LOC100072265,LOC100072279,LOC100072306,LOC100072261,LOC100072184,LOC100072342,LOC100072288,LOC100072317,LOC100072300,LOC100072227,LOC100072195,LOC100072365,LOC100072282,LOC100072290,LOC100072320,LOC100072338,LOC100072221,LOC100072354,LOC100146987,LOC100072206,LOC100072211,LOC100072325,LOC100058393,LOC100072345,LOC100072254,LOC100072330,LOC100072335,LOC100072245
01	156012981	156491374	478394	0	1	LOC100072296,LOC100072258,LOC100072251,LOC100072237,LOC100072368,LOC100072386,LOC100072351,LOC100072271,LOC100072275,LOC100072265,LOC100072279,LOC100072306,LOC100072261,LOC100072398,LOC100072342,LOC100072288,LOC100072317,LOC100072300,LOC100072380,LOC100072365,LOC100072282,LOC100072290,LOC100072320,LOC100072338,LOC100072392,LOC100072354,LOC100146987,LOC100072325,LOC100072375,LOC100072345,LOC100072254,LOC100072330,LOC100072335,LOC100072245
01	156125915	156491374	365460	0	4	LOC100072296,LOC100072368,LOC100072386,LOC100072351,LOC100072306,LOC100072398,LOC100072342,LOC100072288,LOC100072317,LOC100072300,LOC100072380,LOC100072365,LOC100072290,LOC100072320,LOC100072338,LOC100072392,LOC100072354,LOC100146987,LOC100072325,LOC100072375,LOC100072345,LOC100072330,LOC100072335
01	156135693	156491374	355682	0	1	LOC100072296,LOC100072368,LOC100072386,LOC100072351,LOC100072306,LOC100072398,LOC100072342,LOC100072288,LOC100072317,LOC100072300,LOC100072380,LOC100072365,LOC100072290,LOC100072320,LOC100072338,LOC100072392,LOC100072354,LOC100146987,LOC100072325,LOC100072375,LOC100072345,LOC100072330,LOC100072335
01	156449080	156657881	208802	0	2	LOC100072414,LOC100072447,LOC100072426,LOC100072429,LOC100072386,LOC100072432,LOC100072398,LOC100072443,LOC100058435,LOC100072402,LOC100072423,LOC100072392,LOC100072404,LOC100072408
01	156449080	156870455	421376	0,1	2,5	LOC100072414,LOC100072447,LOC100072426,LOC100072429,LOC100072386,LOC100072432,LOC100072464,LOC100072493,LOC100072499,LOC100072398,LOC100072469,LOC100629829,LOC100072443,LOC100058435,LOC100072495,LOC100072402,LOC100072423,LOC100072392,LOC100072404,LOC100072457,LOC100072479,LOC100072473,LOC100072408,LOC100072489,LOC100072477,LOC100072452
01	156449080	157078971	629892	0	1	LOC100072414,LOC100072523,LOC100072560,LOC100072447,LOC100072426,LOC100072514,LOC100072520,LOC100072551,LOC100072429,LOC100072386,LOC100072432,LOC100058565,LOC100058482,LOC100072464,LOC100072541,LOC100072493,LOC100072499,LOC100072508,LOC100072398,LOC100072469,LOC100629829,LOC100072555,LOC100072443,LOC100058435,LOC100072495,LOC100072402,LOC100072536,LOC100072423,LOC100072392,LOC100072404,LOC100072530,LOC100072457,LOC100072479,LOC100072527,LOC100072473,LOC100072408,LOC100072489,LOC100072477,LOC100072539,LOC100072546,LOC100058524,LOC100072452
01	156462763	156818876	356114	1	1	LOC100072414,LOC100072447,LOC100072426,LOC100072429,LOC100072386,LOC100072432,LOC100072464,LOC100072398,LOC100072469,LOC100629829,LOC100072443,LOC100058435,LOC100072402,LOC100072423,LOC100072392,LOC100072404,LOC100072457,LOC100072479,LOC100072473,LOC100072408,LOC100072489,LOC100072477,LOC100072452
01	156462763	156870455	407693	1	1	LOC100072414,LOC100072447,LOC100072426,LOC100072429,LOC100072386,LOC100072432,LOC100072464,LOC100072493,LOC100072499,LOC100072398,LOC100072469,LOC100629829,LOC100072443,LOC100058435,LOC100072495,LOC100072402,LOC100072423,LOC100072392,LOC100072404,LOC100072457,LOC100072479,LOC100072473,LOC100072408,LOC100072489,LOC100072477,LOC100072452
01	156529837	156870455	340619	1	1	LOC100072414,LOC100072447,LOC100072426,LOC100072429,LOC100072432,LOC100072464,LOC100072493,LOC100072499,LOC100072469,LOC100629829,LOC100072443,LOC100058435,LOC100072495,LOC100072423,LOC100072457,LOC100072479,LOC100072473,LOC100072489,LOC100072477,LOC100072452
01	156529837	157078971	549135	0	1	LOC100072414,LOC100072523,LOC100072560,LOC100072447,LOC100072426,LOC100072514,LOC100072520,LOC100072551,LOC100072429,LOC100072432,LOC100058565,LOC100058482,LOC100072464,LOC100072541,LOC100072493,LOC100072499,LOC100072508,LOC100072469,LOC100629829,LOC100072555,LOC100072443,LOC100058435,LOC100072495,LOC100072536,LOC100072423,LOC100072530,LOC100072457,LOC100072479,LOC100072527,LOC100072473,LOC100072489,LOC100072477,LOC100072539,LOC100072546,LOC100058524,LOC100072452
01	178337817	178852497	514681	1	1	LOC100062668,LOC100629700
01	178798269	179550475	752207	3	2	LOC100062807,LOC100062668,LOC100062944

Chrom	Start	End	Size	Cn	Samples (n)	Genes
01	178798269	179730039	931771	3	1	LOC100063356,LOC100062807,LOC100062668,LOC100062944
02	101517	559280	457764	1	1	LOC100630718,LOC100066893,LOC100066956,HOOK1,LOC100067247,FGGY
02	106062109	106063373	1265	0	10	
03	41567820	41631210	63391	3	2	
03	41567820	41635136	67317	3	1	
03	45062312	45062839	528	0	1	GRID2
03	65705932	65951800	245869	3	1	LOC100067903,LOC100066472,LOC100629168,LOC100067952,LOC100067974,LOC100067994,LOC100629252,LOC100629210,LOC100066501,LOC100068012
04	52424614	52612016	187403	3	1	LOC100067077
04	96985336	97315650	330315	3	1	LOC100057695,LOC100057617,LOC100057534,LOC100051071,LOC100057334,LOC100057880,LOC100057789,LOC100057734,LOC100057377,LOC100057459,LOC100057655,LOC100147477,LOC100051145,LOC100057834,LOC100057930,LOC100630419,LOC100058023,LOC100057574,LOC100146500,LOC100057252,LOC100057296
05	37840041	37916448	76408	0	8	LOC100053475,LOC100057667
05	88243192	88258862	15671	3	1	
05	88243192	88494901	251710	3	1	
06	25908752	26126581	217830	3,4	1,12	LOC100067436,MIR149,LOC100067268,LOC100147336,LOC100146642
06	26086675	26126581	39907	4	1	LOC100067436,LOC100147336
06	38278097	38421550	143454	3	2	LY49B,LY49C,LY49E,LOC100063178,LY49F
06	71704684	72493903	789220	0	1	LOC100053477,LOC100629654,LOC100053971,LOC100054452,LOC100054400,LOC100630091,LOC100050632,LOC100055222,LOC100055352,LOC100055645,LOC100055600,LOC100055986,LOC100055946,LOC100055778,LOC100146480,LOC100054497,LOC100054585,LOC100050555,LOC100629677,LOC100147639,LOC100053671,LOC100053818,LOC100053922,LOC100054214,LOC100054306,LOC100054063,LOC100054671,LOC100147637,LOC100054771,LOC100055272,LOC100146474,LOC100055559,LOC100055434,LOC100055688,LOC100055866,LOC100055818,LOC100055734,LOC100050712,LOC100055520,LOC100147260,LOC100055477,LOC100055308,LOC100055176,LOC100055082,LOC100055039,LOC100054995,LOC100054952,LOC100054904,LOC100054856,LOC100054816,LOC100146580,LOC100054626,LOC100054539,LOC100054354,LOC100054256,LOC100054118,LOC100053870,LOC100053767,LOC100053623,LOC100053381,LOC100053282
06	71872446	72485833	613388	0	1	LOC100053971,LOC100054452,LOC100054400,LOC100630091,LOC100050632,LOC100055222,LOC100055352,LOC100055645,LOC100055600,LOC100055946,LOC100055778,LOC100146480,LOC100054497,LOC100054585,LOC100050555,LOC100054214,LOC100054306,LOC100054063,LOC100054671,LOC100147637,LOC100054771,LOC100055272,LOC100146474,LOC100055559,LOC100055434,LOC100055688,LOC100055866,LOC100055818,LOC100055734,LOC100050712,LOC100055520,LOC100147260,LOC100055477,LOC100055308,LOC100055176,LOC100055082,LOC100055039,LOC100054995,LOC100054952,LOC100054904,LOC100054856,LOC100054816,LOC100146580,LOC100054626,LOC100054539,LOC100054354,LOC100054256,LOC100054118
06	71872446	72603288	730843	0	5	LOC100053971,LOC100054452,LOC100054400,LOC100630091,LOC100050632,LOC100055222,LOC100055352,LOC100055645,LOC100055600,LOC100055986,LOC100055946,LOC100055778,LOC100146480,LOC100054497,LOC100054585,LOC100050555,LOC100054214,LOC100054306,LOC100054063,LOC100054671,LOC100147637,LOC100054771,LOC100055272,LOC100146474,LOC100055559,LOC100055434,LOC100055688,LOC100056234,LOC100056025,LOC100056186,LOC100630654,LOC100056100,LOC100055866,LOC100055818,LOC100055734,LOC100050712,LOC100055520,LOC100147260,LOC100055477,LOC100055308,LOC100055176,LOC100055082,LOC100055039,LOC100054995,LOC100054952,LOC100054904,LOC100054856,LOC100054816,LOC100146580,LOC100054626,LOC100054539,LOC100054354,LOC100054256,LOC100054118
06	71872446	72607543	735098	0,1	1,2	LOC100053971,LOC100054452,LOC100054400,LOC100630091,LOC100050632,LOC100055222,LOC100055352,LOC100055645,LOC100055600,LOC100055986,LOC100055946,LOC100055778,LOC100146480,LOC100054497,LOC100054585,LOC100050555,LOC100054214,LOC100054306,LOC100054063,LOC100054671,LOC100147637,LOC100054771,LOC100055272,LOC100146474,LOC100055559,LOC100055434,LOC100055688,LOC100056234,LOC100056025,LOC100056186,LOC100630654,LOC100056100,LOC100055866,LOC100055818,LOC100055734,LOC100050712,LOC100055520,LOC100147260,LOC100055477,LOC100055308,LOC100055176,LOC100055082,LOC100055039,LOC100054995,LOC100054952,LOC100054904,LOC100054856,LOC100054816,LOC100146580,LOC100054626,LOC100054539,LOC100054354,LOC100054256,LOC100054118
06	72032729	72485833	453105	1	3	LOC100630091,LOC100050632,LOC100055222,LOC100055352,LOC100055645,LOC100055600,LOC100055946,LOC100055778,LOC100146480,LOC100054585,LOC100050555,LOC100054671,LOC100147637,LOC100054771,LOC100055272,LOC100146474,LOC100055559,LOC100055434,LOC100055688,LOC100055866,LOC100055818,LOC100055734,LOC100050712,LOC100055520,LOC100147260,LOC100055477,LOC100055308,LOC100055176,LOC100055082,LOC100055039,LOC100054995,LOC100054952,LOC100054904,LOC100054856,LOC100054816,LOC100146580,LOC100054626
06	72032729	72493903	461175	0,1	7,11	LOC100630091,LOC100050632,LOC100055222,LOC100055352,LOC100055645,LOC100055600,LOC100055986,LOC100055946,LOC100055778,LOC100146480,LOC100054585,LOC100050555,LOC100054671,LOC100147637,LOC100054771,LOC100055272,LOC100146474,LOC100055559,LOC100055434,LOC100055688,LOC100055866,LOC100055818,LOC100055734,LOC100050712,LOC100055520,LOC100147260,LOC100055477,LOC100055308,LOC100055176,LOC100055082,LOC100055039,LOC100054995,LOC100054952,LOC100054904,LOC100054856,LOC100054816,LOC100146580,LOC100054626
06	72032729	72603288	570560	0,1	1,2	LOC100630091,LOC100050632,LOC100055222,LOC100055352,LOC100055645,LOC100055600,LOC100055986,LOC100055946,LOC100055778,LOC100146480,LOC100054585,LOC100050555,LOC100054671,LOC100147637,LOC100054771,LOC100055272,LOC100146474,LOC100055559,LOC100055434,LOC100055688,LOC100056234,LOC100056025,LOC100056186,LOC100630654,LOC100056100,LOC100055866,LOC100055818,LOC100055734,LOC100050712,LOC100055520,LOC100147260,LOC100055477,LOC100055308,LOC100055176,LOC100055082,LOC100055039,LOC100054995,LOC100054952,LOC100054904,LOC100054856,LOC100054816,LOC100146580,LOC100054626
06	72032729	72607543	574815	1	1	LOC100630091,LOC100050632,LOC100055222,LOC100055352,LOC100055645,LOC100055600,LOC100055986,LOC100055946,LOC100055778,LOC100146480,LOC100054585,LOC100050555,LOC100054671,LOC100147637,LOC100054771,LOC100055272,LOC100146474,LOC100055559,LOC100055434,LOC100055688,LOC100056234,LOC100056025,LOC100056186,LOC100630654,LOC100056100,LOC100055866,LOC100055818,LOC100055734,LOC100050712,LOC100055520,LOC100147260,LOC100055477,LOC100055308,LOC100055176,LOC100055082,LOC100055039,LOC100054995,LOC100054952,LOC100054904,LOC100054856,LOC100054816,LOC100146580,LOC100054626

Chrom	Start	End	Size	Cn	Samples (n)	Genes
06	72032729	72646366	613638	0	1	LOC100630091,LOC100050632,LOC100055222,LOC100055352,LOC100055645,LOC100055600,LOC100055986,LOC100146967,LOC100055946,LOC100056394,LOC100146670,LOC100055778,LOC100146480,LOC100054585,LOC100050555,LOC100054671,LOC100147637,LOC100054771,LOC100055272,LOC100146474,LOC100055559,LOC100055434,LOC100055688,LOC100056234,LOC100056025,LOC100056355,LOC100056186,LOC100630654,LOC100056100,LOC100055866,LOC100055818,LOC100055734,LOC100050712,LOC100055520,LOC100147260,LOC100055477,LOC100055308,LOC100055176,LOC100055082,LOC100055039,LOC100054995,LOC100054952,LOC100054904,LOC100054856,LOC100054816,LOC100146580,LOC100054626
07	31406445	31520977	114533	0,1	1,1	LOC100071915,LOC100071904,LOC100071894,LOC100071910,LOC100630881
07	52610482	52677786	67305	3	1	LOC100146282,LOC100064357,LOC100064296
07	52610482	52739317	128836	3	1	LOC100146282,LOC100064357,LOC100055731,LOC100064420,LOC100064296
07	73083306	73197149	113844	0	1	LOC100146503,LOC100067901,LOC100067972,LOC100067873,LOC100068010,LOC100067992,LOC100067950,LOC100067928,LOC100147186,LOC100146803
08	4280605	4430473	149869	0	1	LOC100062472
08	4430473	4621044	190572	0,1	2,1	LOC100062472
08	4430473	4646812	216340	1	2	LOC100062472
08	4537919	4621044	83126	0	1	LOC100062472
09	29889627	29896240	6614	0	1	
09	31574454	31574969	516	1	1	PXDNL
09	50912923	51314829	401907	0	1	OXR1
09	57755893	57774233	18341	0	4	
10	674485	1271225	596741	3	1	LOC100053533,LOC100053634
11	54645681	54812394	166714	0	3	
11	60428534	61282299	853766	3	1	LOC100147042,MAP2K3,LOC100053429,LOC100053330,LOC100053286,USP22,LOC100051166,MPRIP,LOC100052895,LOC100052790,COPS3,LOC100052734,LOC100052684
11	60428689	61282299	853611	3	1	LOC100147042,MAP2K3,LOC100053429,LOC100053330,LOC100053286,USP22,LOC100051166,MPRIP,LOC100052895,LOC100052790,COPS3,LOC100052734,LOC100052684
12	9578500	10526206	947707	3	1	MAPK8IP1,LOC100056637,SLC35C1,LOC100056544,MIR493A,LOC100056452,LOC100056367,LOC100050141,LOC100056203
12	12524489	13401991	877503	3	1	LOC100146383,LOC100054355,LOC100054307,LOC100054258,LOC100054215,LOC100054169,LOC100054120,LOC100054065,LOC100054021,LOC100050556,LOC100053872,LOC100050476,LOC100053820,LOC100053768,LOC100629822,LOC100053672,LOC100053625,LOC100053568,LOC100053527,LOC100147669,LOC100053479,LOC100053427,LOC100053382,LOC100053328,LOC100053284,LOC100053236,LOC100053185,LOC100053134,LOC100053085,LOC100053045,LOC100052992,LOC100146502,LOC100052946,LOC100146388,LOC100052842,LOC100147383,LOC100052788,LOC100052732,LOC100050399,LOC100052683,LOC100052629,LOC100052515,LOC100052454,LOC100052398,LOC100052341,LOC100052217,LOC100052157,LOC100052096,LOC100052037,LOC100146189,LOC100051977,LOC100051914,LOC100146810,LOC100050328,LOC100051731,LOC100051664,LOC100051592,LOC100051520,LOC100051452,LOC100051381,LOC100050265,LOC100630831
12	12524489	13422256	897768	3	1	LOC100054453,LOC100146383,LOC100054355,LOC100054307,LOC100054258,LOC100054215,LOC100054169,LOC100054120,LOC100054065,LOC100054021,LOC100050556,LOC100053872,LOC100050476,LOC100053820,LOC100053768,LOC100629822,LOC100053672,LOC100053625,LOC100053568,LOC100053527,LOC100147669,LOC100053479,LOC100053427,LOC100053382,LOC100053328,LOC100053284,LOC100053236,LOC100053185,LOC100053134,LOC100053085,LOC100053045,LOC100052992,LOC100146502,LOC100052946,LOC100146388,LOC100052842,LOC100147383,LOC100052788,LOC100052732,LOC100050399,LOC100052683,LOC100052629,LOC100052515,LOC100052454,LOC100052398,LOC100052341,LOC100052217,LOC100052157,LOC100052096,LOC100052037,LOC100146189,LOC100051977,LOC100051914,LOC100146810,LOC100050328,LOC100051731,LOC100051664,LOC100051592,LOC100051520,LOC100051452,LOC100051381,LOC100050265,LOC100630831
12	12829176	13401991	572816	3	1	LOC100146383,LOC100054355,LOC100054307,LOC100054258,LOC100054215,LOC100054169,LOC100054120,LOC100054065,LOC100054021,LOC100050556,LOC100053872,LOC100050476,LOC100053820,LOC100053768,LOC100629822,LOC100053672,LOC100053625,LOC100053568,LOC100053527,LOC100147669,LOC100053479,LOC100053427,LOC100053382,LOC100053328,LOC100053284,LOC100053236,LOC100053185,LOC100053134,LOC100053085,LOC100053045,LOC100052992,LOC100146502,LOC100052946,LOC100146388,LOC100052842,LOC100147383,LOC100052788,LOC100052732,LOC100050399,LOC100052683
12	12829176	13422256	593081	3	2	LOC100054453,LOC100146383,LOC100054355,LOC100054307,LOC100054258,LOC100054215,LOC100054169,LOC100054120,LOC100054065,LOC100054021,LOC100050556,LOC100053872,LOC100050476,LOC100053820,LOC100053768,LOC100629822,LOC100053672,LOC100053625,LOC100053568,LOC100053527,LOC100147669,LOC100053479,LOC100053427,LOC100053382,LOC100053328,LOC100053284,LOC100053236,LOC100053185,LOC100053134,LOC100053085,LOC100053045,LOC100052992,LOC100146502,LOC100052946,LOC100146388,LOC100052842,LOC100147383,LOC100052788,LOC100052732,LOC100050399,LOC100052683
12	13149957	14128309	978353	3	1	LOC100056626,LOC100056578,LOC100056531,LOC100056488,LOC100629158,LOC100050633,LOC100056395,LOC100056356,LOC100056314,LOC100056273,LOC100056235,LOC100056187,LOC100056146,LOC100630788,LOC100629923,LOC100056026,LOC100055988,LOC100146992,LOC100055947,LOC100147569,LOC100146290,LOC100055908,LOC100055867,LOC100055780,LOC100630577,LOC100055646,LOC100055601,LOC100055561,LOC100055521,LOC100055478,LOC100055435,LOC100055397,LOC100055353,LOC100055309,LOC100055273,LOC100055224,LOC100055177,LOC100055129,LOC100055083,LOC100052898,LOC100147187,LOC100054954,LOC100054906,LOC100054858,LOC100054773,LOC100054722,LOC100054673,LOC100054627,LOC100054586,LOC100054541,LOC100054498,LOC100054453,LOC100146383,LOC100054355,LOC100054307,LOC100054258,LOC100054215,LOC100054169,LOC100054120,LOC100054065,LOC100054021,LOC100050556,LOC100053872,LOC100050476,LOC100053820,LOC100053768,LOC100629822
12	13170959	13401991	231033	3	1	LOC100146383,LOC100054355,LOC100054307,LOC100054258,LOC100054215,LOC100054169,LOC100054120,LOC100054065,LOC100054021,LOC100050556,LOC100053872,LOC100050476,LOC100053820

Chrom	Start	End	Size	Cn	Samples (n)	Genes
12	13170959	14124730	953772	3	1	LOC100056626,LOC100056578,LOC100056531,LOC100056488,LOC100629158,LOC100050633,LOC100056395,LOC100056356,LOC100056314,LOC100056273,LOC100056235,LOC100056187,LOC100056146,LOC100630788,LOC100629923,LOC100056026,LOC100055988,LOC100146992,LOC100055947,LOC100147569,LOC100146290,LOC100055908,LOC100055867,LOC100055780,LOC100630577,LOC100055646,LOC100055601,LOC100055561,LOC100055521,LOC100055478,LOC100055435,LOC100055397,LOC100055353,LOC100055309,LOC100055273,LOC100055224,LOC100055177,LOC100055129,LOC100055083,LOC100052898,LOC100147187,LOC100054954,LOC100054906,LOC100054858,LOC100054773,LOC100054722,LOC100054673,LOC100054627,LOC100054586,LOC100054541,LOC100054498,LOC100054453,LOC100146383,LOC100054355,LOC100054307,LOC100054258,LOC100054215,LOC100054169,LOC100054120,LOC100054065,LOC100054021,LOC100050556,LOC100053872,LOC100050476,LOC100053820
12	13364132	14297056	932925	3	1	LOC100056869,LOC100146809,LOC100067978,LOC100067957,LOC100056826,LOC100056793,LOC100056755,LOC100147000,LOC100056708,LOC100056626,LOC100056578,LOC100056531,LOC100056488,LOC100629158,LOC100050633,LOC100056395,LOC100056356,LOC100056314,LOC100056273,LOC100056235,LOC100056187,LOC100056146,LOC100630788,LOC100629923,LOC100056026,LOC100055988,LOC100146992,LOC100055947,LOC100147569,LOC100146290,LOC100055908,LOC100055867,LOC100055780,LOC100630577,LOC100055646,LOC100055601,LOC100055561,LOC100055521,LOC100055478,LOC100055435,LOC100055397,LOC100055353,LOC100055309,LOC100055273,LOC100055224,LOC100055177,LOC100055129,LOC100055083,LOC100052898,LOC100147187,LOC100054954,LOC100054906,LOC100054858,LOC100054773,LOC100054722,LOC100054673,LOC100054627,LOC100054586,LOC100054541,LOC100054498,LOC100054453,LOC100146383,LOC100054355,LOC100054307,LOC100054258
12	13439146	14391372	952227	4	1	LOC100057026,LOC100050714,LOC100056989,LOC100056947,LOC100056906,LOC100056869,LOC100146809,LOC100067978,LOC100067957,LOC100056826,LOC100056793,LOC100056755,LOC100147000,LOC100056708,LOC100056626,LOC100056578,LOC100056531,LOC100056488,LOC100629158,LOC100050633,LOC100056395,LOC100056356,LOC100056314,LOC100056273,LOC100056235,LOC100056187,LOC100056146,LOC100630788,LOC100629923,LOC100056026,LOC100055988,LOC100146992,LOC100055947,LOC100147569,LOC100146290,LOC100055908,LOC100055867,LOC100055780,LOC100630577,LOC100055646,LOC100055601,LOC100055561,LOC100055521,LOC100055478,LOC100055435,LOC100055397,LOC100055353,LOC100055309,LOC100055273,LOC100055224,LOC100055177,LOC100055129,LOC100055083,LOC100052898,LOC100147187,LOC100054954,LOC100054906,LOC100054858,LOC100054773,LOC100054722,LOC100054673,LOC100054627,LOC100054586,LOC100054541
12	13488187	14391372	903186	4	1	LOC100057026,LOC100050714,LOC100056989,LOC100056947,LOC100056906,LOC100056869,LOC100146809,LOC100067978,LOC100067957,LOC100056826,LOC100056793,LOC100056755,LOC100147000,LOC100056708,LOC100056626,LOC100056578,LOC100056531,LOC100056488,LOC100629158,LOC100050633,LOC100056395,LOC100056356,LOC100056314,LOC100056273,LOC100056235,LOC100056187,LOC100056146,LOC100630788,LOC100629923,LOC100056026,LOC100055988,LOC100146992,LOC100055947,LOC100147569,LOC100146290,LOC100055908,LOC100055867,LOC100055780,LOC100630577,LOC100055646,LOC100055601,LOC100055561,LOC100055521,LOC100055478,LOC100055435,LOC100055397,LOC100055353,LOC100055309,LOC100055273,LOC100055224,LOC100055177,LOC100055129,LOC100055083,LOC100052898,LOC100147187,LOC100054954,LOC100054906,LOC100054858,LOC100054773,LOC100054722
12	13573356	14354314	780959	4	1	LOC100056947,LOC100056906,LOC100056869,LOC100146809,LOC100067978,LOC100067957,LOC100056826,LOC100056793,LOC100056755,LOC100147000,LOC100056708,LOC100056626,LOC100056578,LOC100056531,LOC100056488,LOC100629158,LOC100050633,LOC100056395,LOC100056356,LOC100056314,LOC100056273,LOC100056235,LOC100056187,LOC100056146,LOC100630788,LOC100629923,LOC100056026,LOC100055988,LOC100146992,LOC100055947,LOC100147569,LOC100146290,LOC100055908,LOC100055867,LOC100055780,LOC100630577,LOC100055646,LOC100055601,LOC100055561,LOC100055521,LOC100055478,LOC100055435,LOC100055397,LOC100055353,LOC100055309,LOC100055273,LOC100055224,LOC100055177,LOC100055129,LOC100055083,LOC100052898
12	13678953	14391372	712420	3	1	LOC100057026,LOC100050714,LOC100056989,LOC100056947,LOC100056906,LOC100056869,LOC100146809,LOC100067978,LOC100067957,LOC100056826,LOC100056793,LOC100056755,LOC100147000,LOC100056708,LOC100056626,LOC100056578,LOC100056531,LOC100056488,LOC100629158,LOC100050633,LOC100056395,LOC100056356,LOC100056314,LOC100056273,LOC100056235,LOC100056187,LOC100056146,LOC100630788,LOC100629923,LOC100056026,LOC100055988,LOC100146992,LOC100055947,LOC100147569,LOC100146290,LOC100055908,LOC100055867,LOC100055780,LOC100630577,LOC100055646,LOC100055601,LOC100055561,LOC100055521,LOC100055478,LOC100055435,LOC100055397
12	13945011	14128309	183299	4	1	LOC100056626,LOC100056578,LOC100056531,LOC100056488,LOC100629158,LOC100050633,LOC100056395,LOC100056356,LOC100056314,LOC100056273,LOC100056235,LOC100056187,LOC100056146
12	13945011	14391003	445993	3	1	LOC100057026,LOC100050714,LOC100056989,LOC100056947,LOC100056906,LOC100056869,LOC100146809,LOC100067978,LOC100067957,LOC100056826,LOC100056793,LOC100056755,LOC100147000,LOC100056708,LOC100056626,LOC100056578,LOC100056531,LOC100056488,LOC100629158,LOC100050633,LOC100056395,LOC100056356,LOC100056314,LOC100056273,LOC100056235,LOC100056187,LOC100056146
12	13945011	14777981	832971	3	1	LOC100058077,LOC100147282,LOC100058036,LOC100057989,LOC100057945,LOC100057894,LOC100057845,LOC100057804,LOC100057756,LOC100057709,LOC100057670,LOC100057589,LOC100057547,LOC100057513,LOC100057472,LOC100057433,LOC100057393,LOC100057352,LOC100057310,LOC100147286,LOC100146990,LOC100057269,LOC100057228,LOC100057191,LOC100057110,LOC100146600,LOC100057026,LOC100050714,LOC100056989,LOC100056947,LOC100056906,LOC100056869,LOC100146809,LOC100067978,LOC100067957,LOC100056826,LOC100056793,LOC100056755,LOC100147000,LOC100056708,LOC100056626,LOC100056578,LOC100056531,LOC100056488,LOC100629158,LOC100050633,LOC100056395,LOC100056356,LOC100056314,LOC100056273,LOC100056235,LOC100056187,LOC100056146
12	13946509	14128309	181801	0	1	LOC100056626,LOC100056578,LOC100056531,LOC100056488,LOC100629158,LOC100050633,LOC100056395,LOC100056356,LOC100056314,LOC100056273,LOC100056235,LOC100056187,LOC100056146
12	13946509	14391003	444495	3	1	LOC100057026,LOC100050714,LOC100056989,LOC100056947,LOC100056906,LOC100056869,LOC100146809,LOC100067978,LOC100067957,LOC100056826,LOC100056793,LOC100056755,LOC100147000,LOC100056708,LOC100056626,LOC100056578,LOC100056531,LOC100056488,LOC100629158,LOC100050633,LOC100056395,LOC100056356,LOC100056314,LOC100056273,LOC100056235,LOC100056187,LOC100056146
12	13946509	14391372	444864	3	1	LOC100057026,LOC100050714,LOC100056989,LOC100056947,LOC100056906,LOC100056869,LOC100146809,LOC100067978,LOC100067957,LOC100056826,LOC100056793,LOC100056755,LOC100147000,LOC100056708,LOC100056626,LOC100056578,LOC100056531,LOC100056488,LOC100629158,LOC100050633,LOC100056395,LOC100056356,LOC100056314,LOC100056273,LOC100056235,LOC100056187,LOC100056146

Chrom	Start	End	Size	Cn	Samples (n)	Genes
12	14108229	14777981	669753	3	2	LOC100058077,LOC100147282,LOC100058036,LOC100057989,LOC100057945,LOC100057894,LOC100057845,LOC100057804,LOC100057756,LOC100057709,LOC100057670,LOC100057589,LOC100057547,LOC100057513,LOC100057472,LOC100057433,LOC100057393,LOC100057352,LOC100057310,LOC100147286,LOC100146990,LOC100057269,LOC100057228,LOC100057191,LOC100057110,LOC100146600,LOC100057026,LOC100050714,LOC100056989,LOC100056947,LOC100056906,LOC100056869,LOC100146809,LOC100067978,LOC100067957,LOC100056826,LOC100056793,LOC100056755,LOC100147000,LOC100056708,LOC100056626,LOC100056578
12	14108352	14777981	669630	4	1	LOC100058077,LOC100147282,LOC100058036,LOC100057989,LOC100057945,LOC100057894,LOC100057845,LOC100057804,LOC100057756,LOC100057709,LOC100057670,LOC100057589,LOC100057547,LOC100057513,LOC100057472,LOC100057433,LOC100057393,LOC100057352,LOC100057310,LOC100147286,LOC100146990,LOC100057269,LOC100057228,LOC100057191,LOC100057110,LOC100146600,LOC100057026,LOC100050714,LOC100056989,LOC100056947,LOC100056906,LOC100056869,LOC100146809,LOC100067978,LOC100067957,LOC100056826,LOC100056793,LOC100056755,LOC100147000,LOC100056708,LOC100056626,LOC100056578
13	5125834	5861174	735341	0	1	INTS1,LOC100053482,LOC100053430,LOC100053289,LOC100147505,MAD1L1
14	59249572	59463001	213430	3	1	APC
15	56397305	57018352	621048	1	1	LOC100629144
17	79672632	80576852	904221	3	1	LOC100069263,LOC100630815,LOC100069151,LOC100069135,LOC100069163,LOC100067334,LOC100067313,LOC100067285,LOC100630737,LOC100067211,CUL4A,PCID2,LOC100067119,LOC100067094,LOC100067072,MCF2L,ATP11A
18	11533872	12399073	865202	3	1	LOC100052304,LOC100052243,LOC100049893,STEAP3,LOC100629302,DBI,LOC100051693,LOC100051620,LOC100147031
18	11660478	12399073	738596	3	1	LOC100052304,LOC100052243,LOC100049893,STEAP3,LOC100629302,DBI
18	75813682	75879418	65737	3	1	LOC100067956,LOC100067782
20	32059082	32210308	151227	0	5	LOC100059844,LOC100059681
20	32059082	32540917	481836	0	1	LOC100059994,LOC100059844,LOC100059681
20	32127612	32210308	82697	0	12	
20	34019567	34689953	670387	4	1	LOC100062360,LOC100629770,LOC100629238,LOC100061879,MLN,LOC100061821,IP6K3,LOC100146183
23	2699858	2846110	146253	3,4	1,1	FBP2,LOC100063910,LOC100061462
23	3177140	3362366	185227	4	1	
23	3889242	3989324	100083	4	1	
23	4031496	4139129	107634	3	1	LOC100629630,ZCCHC6
23	10629248	10711625	82378	1	1	TLE1
23	11864935	11962495	97561	0	1	
23	12947247	12973967	26721	0	1	
23	13650182	13822679	172498	0	4	LOC100063136
23	14586061	14750197	164137	0	5	LOC100063499
23	16784961	16799292	14332	0	52	
23	17718685	17730679	11995	0	2	RORB
23	18319445	18461870	142426	0	1	LOC100055020
23	19207420	19267349	59930	0	1	
23	19637508	19646702	9195	0	11	
23	21781331	21902570	121240	0	2	LOC100057045,PIP5K1B
23	22561847	22690659	128813	3	1	LOC100057373,DOCK8
23	26109618	26338432	228815	0	2	MIR101-2,RCL1,LOC100059240,LOC100051347,LOC100051417,LOC100629203,LOC100059120
23	26705409	26841790	136382	3	1	LOC100059542,LOC100051703,LOC100051630
23	27264180	27480326	216147	0	2	LOC100059908,RANBP6,LOC100059779
23	28314392	28542968	228577	1	1	
23	29073581	29334199	260619	0,1	1,6	PTPRD
23	30185805	30230205	44401	0,1,3	4,3,1	PTPRD
23	30917994	31045223	127230	0	1	PTPRD
23	32203122	32240543	37422	0,1	22,6	
23	32861094	33138870	277777	1	1	LOC100052009,TYRP1
23	33609793	33807125	197333	3	1	
23	33855357	33940316	84960	0,3	3,1	
23	34802438	34846917	44480	0	1	FREM1,LOC100062259
23	34937323	34959264	21942	0	13	FREM1
23	35814091	35987901	173811	3	5	
23	36453320	36513782	60463	0	1	
23	37253564	37278514	24951	0	1	LOC100063235
23	38033765	38090159	56395	0	1	ADAMTSL1
23	39330423	39420063	89641	0	1	LOC100052427

Chrom	Start	End	Size	Cn	Samples (n)	Genes
23	39812047	39937024	124978	0	2	LOC100064410
23	41781528	41903368	121841	0	1	
23	43018370	43327461	309092	0,1	7,6	
23	45169561	45196242	26682	0	5	LOC100067032
23	46081939	46141402	59464	0	5	
23	47572633	47904587	331955	0,1	9,5	LOC100067610
23	48041799	48527901	486103	0,1	1,4	LOC100067680
23	49243058	49323236	80179	0	2	
23	50883552	50929623	46072	0	8	LOC100063025
23	51761343	51803472	42130	0	10	
23	52306999	52318032	11034	0	4	
23	52460740	52479134	18395	0,1	6,1	
23	52596142	52986449	390308	3	4	LOC100062234,LOC100054996
23	53086471	53239727	153257	3	1	SHC3
23	53980697	54021152	40456	0	1	LOC100060076
23	54759475	54980902	221428	0,1,3	3,3,1	LOC100056145,LOC100146174,LOC100056530,LOC100146796,LOC100147073
23	55148497	55235809	87313	0,3,4	2,18,5	ECM2,LOC100054857,IPPK
24	7167494	7292049	124556	0	1	
24	32416012	32628728	212717	3	1	TTC8,EML5
25	17703603	18248415	544813	0	1	WDR31,LOC100050237,PRPF4,LOC100053266,LOC100050369,FKBP15,LOC100053455,LOC100146201,LOC100034197,LOC100056127,LOC100056214,ZFP37
25	26040043	26942120	902078	1	1	LOC100071244,LOC100071251,LOC100067520,LOC100071258,LOC100071264,LOC100071270,LOC100071275,LOC100071278,LOC100071283,LOC100071287,LOC100071297,LOC100146817,LOC100071311,LOC100071317,LOC100147676,LOC100071322,LOC100071329,LOC100071332,LOC100071338,LOC100071346,LOC100071352,LOC100071358,LOC100071365,LOC100071376,LOC100071382,LOC100071392,LOC100071402,LOC100071407,LOC100071413,LOC100071420,LOC100146823,LOC100071438,LOC100071445,LOC100071448,LOC100071452,LOC100071460,LOC100071469,LOC100071479,LOC100071486,LOC100071488,LOC100071492,LOC100071496,LOC100071502,LOC100071506,LOC100071511,LOC100071518,LOC100071522,LOC100071528,LOC100071531,LOC100071537,LOC100071545,LOC100071560,LOC100071565,LOC100071570,LOC100147012,LOC100071576,LOC100071580,LOC100071584,LOC100071594,LOC100071600
25	26071478	26599374	527897	1	1	LOC100071402,LOC100071407,LOC100071413,LOC100071420,LOC100146823,LOC100071438,LOC100071445,LOC100071448,LOC100071452,LOC100071460,LOC100071469,LOC100071479,LOC100071486,LOC100071488,LOC100071492,LOC100071496,LOC100071502,LOC100071506,LOC100071511,LOC100071518,LOC100071522,LOC100071528,LOC100071531,LOC100071537,LOC100071545,LOC100071560,LOC100071565,LOC100071570,LOC100147012,LOC100071576,LOC100071580,LOC100071584,LOC100071594
25	26318531	26918263	599733	1	2	LOC100071251,LOC100067520,LOC100071258,LOC100071264,LOC100071270,LOC100071275,LOC100071278,LOC100071283,LOC100071287,LOC100071297,LOC100146817,LOC100071311,LOC100071317,LOC100147676,LOC100071322,LOC100071329,LOC100071332,LOC100071338,LOC100071346,LOC100071352,LOC100071358,LOC100071365,LOC100071376,LOC100071382,LOC100071392,LOC100071402,LOC100071407,LOC100071413,LOC100071420,LOC100146823,LOC100071438,LOC100071445,LOC100071448,LOC100071452,LOC100071460,LOC100071469,LOC100071479,LOC100071486,LOC100071488,LOC100071492,LOC100071496,LOC100071502
25	26318531	26942120	623590	1	7	LOC100071244,LOC100071251,LOC100067520,LOC100071258,LOC100071264,LOC100071270,LOC100071275,LOC100071278,LOC100071283,LOC100071287,LOC100071297,LOC100146817,LOC100071311,LOC100071317,LOC100147676,LOC100071322,LOC100071329,LOC100071332,LOC100071338,LOC100071346,LOC100071352,LOC100071358,LOC100071365,LOC100071376,LOC100071382,LOC100071392,LOC100071402,LOC100071407,LOC100071413,LOC100071420,LOC100146823,LOC100071438,LOC100071445,LOC100071448,LOC100071452,LOC100071460,LOC100071469,LOC100071479,LOC100071486,LOC100071488,LOC100071492,LOC100071496,LOC100071502
25	26318605	26942120	623516	1	1	LOC100071244,LOC100071251,LOC100067520,LOC100071258,LOC100071264,LOC100071270,LOC100071275,LOC100071278,LOC100071283,LOC100071287,LOC100071297,LOC100146817,LOC100071311,LOC100071317,LOC100147676,LOC100071322,LOC100071329,LOC100071332,LOC100071338,LOC100071346,LOC100071352,LOC100071358,LOC100071365,LOC100071376,LOC100071382,LOC100071392,LOC100071402,LOC100071407,LOC100071413,LOC100071420,LOC100146823,LOC100071438,LOC100071445,LOC100071448,LOC100071452,LOC100071460,LOC100071469,LOC100071479,LOC100071486,LOC100071488,LOC100071492,LOC100071496,LOC100071502
25	26361000	26918263	557264	0,1	1,5	LOC100071251,LOC100067520,LOC100071258,LOC100071264,LOC100071270,LOC100071275,LOC100071278,LOC100071283,LOC100071287,LOC100071297,LOC100146817,LOC100071311,LOC100071317,LOC100147676,LOC100071322,LOC100071329,LOC100071332,LOC100071338,LOC100071346,LOC100071352,LOC100071358,LOC100071365,LOC100071376,LOC100071382,LOC100071392,LOC100071402,LOC100071407,LOC100071413,LOC100071420,LOC100146823,LOC100071438,LOC100071445,LOC100071448,LOC100071452,LOC100071460,LOC100071469,LOC100071479,LOC100071486
25	26361000	26942120	581121	0,1	14,35	LOC100071244,LOC100071251,LOC100067520,LOC100071258,LOC100071264,LOC100071270,LOC100071275,LOC100071278,LOC100071283,LOC100071287,LOC100071297,LOC100146817,LOC100071311,LOC100071317,LOC100147676,LOC100071322,LOC100071329,LOC100071332,LOC100071338,LOC100071346,LOC100071352,LOC100071358,LOC100071365,LOC100071376,LOC100071382,LOC100071392,LOC100071402,LOC100071407,LOC100071413,LOC100071420,LOC100146823,LOC100071438,LOC100071445,LOC100071448,LOC100071452,LOC100071460,LOC100071469,LOC100071479,LOC100071486
25	26361000	27108765	747766	1	1	RC3H2,LOC100071170,LOC100071180,LOC100067499,LOC100071189,LOC100630709,LOC100071212,LOC100071218,LOC100071227,LOC100071236,LOC100071244,LOC100071251,LOC100067520,LOC100071258,LOC100071264,LOC100071270,LOC100071275,LOC100071278,LOC100071283,LOC100071287,LOC100071297,LOC100146817,LOC100071311,LOC100071317,LOC100147676,LOC100071322,LOC100071329,LOC100071332,LOC100071338,LOC100071346,LOC100071352,LOC100071358,LOC100071365,LOC100071376,LOC100071382,LOC100071392,LOC100071402,LOC100071407,LOC100071413,LOC100071420,LOC100146823,LOC100071438,LOC100071445,LOC100071448,LOC100071452,LOC100071460,LOC100071469,LOC100071479,LOC100071486

Chrom	Start	End	Size	Cn	Samples (n)	Genes
25	26361000	27125754	764755	1	3	RC3H2,LOC100071170,LOC100071180,LOC100067499,LOC100071189,LOC100630709,LOC100071212,LOC100071218,LOC100071227,LOC100071236,LOC100071244,LOC100071251,LOC100067520,LOC100071258,LOC100071264,LOC100071270,LOC100071275,LOC100071278,LOC100071283,LOC100071287,LOC100071297,LOC100146817,LOC100071311,LOC100071317,LOC100147676,LOC100071322,LOC100071329,LOC100071332,LOC100071338,LOC100071346,LOC100071352,LOC100071358,LOC100071365,LOC100071376,LOC100071382,LOC100071392,LOC100071402,LOC100071407,LOC100071413,LOC100071420,LOC100146823,LOC100071438,LOC100071445,LOC100071448,LOC100071452,LOC100071460,LOC100071469,LOC100071479,LOC100071486
25	26361000	27294177	933178	1	1	GPR21,LOC100067385,ZBTB26,ZBTB6,RC3H2,LOC100071170,LOC100071180,LOC100067499,LOC100071189,LOC100630709,LOC100071212,LOC100071218,LOC100071227,LOC100071236,LOC100071244,LOC100071251,LOC100067520,LOC100071258,LOC100071264,LOC100071270,LOC100071275,LOC100071278,LOC100071283,LOC100071287,LOC100071297,LOC100146817,LOC100071311,LOC100071317,LOC100147676,LOC100071322,LOC100071329,LOC100071332,LOC100071338,LOC100071346,LOC100071352,LOC100071358,LOC100071365,LOC100071376,LOC100071382,LOC100071392,LOC100071402,LOC100071407,LOC100071413,LOC100071420,LOC100146823,LOC100071438,LOC100071445,LOC100071448,LOC100071452,LOC100071460,LOC100071469,LOC100071479,LOC100071486
25	26393690	26942120	548431	1	1	LOC100071244,LOC100071251,LOC100067520,LOC100071258,LOC100071264,LOC100071270,LOC100071275,LOC100071278,LOC100071283,LOC100071287,LOC100071297,LOC100146817,LOC100071311,LOC100071317,LOC100147676,LOC100071322,LOC100071329,LOC100071332,LOC100071338,LOC100071346,LOC100071352,LOC100071358,LOC100071365,LOC100071376,LOC100071382,LOC100071392,LOC100071402,LOC100071407,LOC100071413,LOC100071420,LOC100146823,LOC100071438,LOC100071445,LOC100071448,LOC100071452,LOC100071460,LOC100071469
25	26509315	26866414	357100	0	1	LOC100071270,LOC100071275,LOC100071278,LOC100071283,LOC100071287,LOC100071297,LOC100146817,LOC100071311,LOC100071317,LOC100147676,LOC100071322,LOC100071329,LOC100071332,LOC100071338,LOC100071346,LOC100071352,LOC100071358,LOC100071365,LOC100071376,LOC100071382,LOC100071392,LOC100071402,LOC100071407,LOC100071413,LOC100071420,LOC100146823
25	26512888	26866414	353527	0	1	LOC100071270,LOC100071275,LOC100071278,LOC100071283,LOC100071287,LOC100071297,LOC100146817,LOC100071311,LOC100071317,LOC100147676,LOC100071322,LOC100071329,LOC100071332,LOC100071338,LOC100071346,LOC100071352,LOC100071358,LOC100071365,LOC100071376,LOC100071382,LOC100071392,LOC100071402,LOC100071407,LOC100071413,LOC100071420
28	159333	493046	333714	4	2	TBC1D15,LOC100056811,LOC100049910,LOC100049839
29	282613	631698	349086	0	1	LOC100070840,LOC100070867,LOC100070887,LOC100058171,LOC100054174
29	8611891	8844864	232974	0	1	LOC100064524,WAC
30	1889883	2496898	607016	3	1	LOC100060872,LOC100630473
