# Supplementary material for: Analysis of copy number variants by three detection algorithms and their association with body size in horses
Source: BMC Genomics. 2013 Jul 18;14:487. doi: 10.1186/1471-2164-14-487 (PMC3720552; doi:10.1186/1471-2164-14-487)
Supplement: Additional file 2 — CNVs detected by PennCNV. The table summarises start and end positions of detected CNVs, their size, copy number, number of samples and genes located in CNV regions. Text in PDF format. [file 1471-2164-14-487-S2.pdf]

Chrom	Start	End	Size	Cn	Samples (n)	Genes
01	10227674	10281539	53866	3	1	LOC100058262
01	15792557	15820581	28025	0,1	1,2	LOC100067810
01	28700170	28750510	50341	1	1	LOC100070213
01	28700170	28773320	73151	1	1	LOC100070213
01	38378834	38425494	46661	3	1	
01	39587269	39748373	161105	1	1	LIPK,LOC100071690,LOC100071675
01	44009907	44013805	3899	1	1	
01	45241089	45434712	193624	1	1	LOC100072003
01	46662564	46726266	63703	1	1	
01	56852665	56856691	4027	3	1	LOC100072614
01	64282841	64290693	7853	1	1	LOC100064366
01	68778089	68778947	859	1	1	
01	77958896	78030449	71554	1	1	PCNXL2
01	89422205	89439454	17250	1	1	
01	92261671	92266103	4433	1	1	LOC100068475
01	110973212	110977361	4150	1	1	
01	111524212	111565898	41687	1	1	
01	119434134	119503183	69050	3	3	CCDC33,LOC100051799
01	135802169	135938654	136486	1	1	
01	135938654	136025318	86665	1	1	
01	135938654	136053322	114669	1	1	
01	144277678	144340953	63276	3	1	DUOX2,LOC100070557,LOC100070541
01	151343566	151542571	199006	1	1	LOC100057596,LOC100071684
01	154545499	154635880	90382	1	1	LOC100057999,SLC12A6,LOC100057956
01	155487276	155510180	22905	0	3	LOC100058312
01	155487276	155546701	59426	1,3	12,2	LOC100072117,LOC100072112,LOC100058312
01	155487276	155593582	106307	0,1,3	3,3,1	LOC100072126,LOC100072129,LOC100072120,LOC100072117,LOC100072112,LOC100058312
01	155487276	155652475	165200	1	13	LOC100072126,LOC100072136,LOC100072146,LOC100072141,LOC100072149,LOC100072129,LOC100072120,LOC100072117,LOC100072112,LOC100058312
01	155487276	155656642	169367	0,1	11,77	LOC100072126,LOC100072136,LOC100072146,LOC100072141,LOC100072149,LOC100072129,LOC100072120,LOC100072117,LOC100072112,LOC100058312
01	155487276	156012981	525706	1	1	LOC100072179,LOC100072176,LOC100072215,LOC100058354,LOC100072192,LOC100072126,LOC100072136,LOC100072202,LOC100072146,LOC100072141,LOC100072184,LOC100072149,LOC100072227,LOC100072195,LOC100072168,LOC100072221,LOC100072129,LOC100072172,LOC100072206,LOC100072211,LOC100072120,LOC100058393,LOC100072117,LOC100072158,LOC100072112,LOC100072163,LOC100058312
01	155546142	155546701	560	1	2	
01	155795029	156012981	217953	1	1	LOC100072179,LOC100072215,LOC100058354,LOC100072192,LOC100072202,LOC100072184,LOC100072227,LOC100072195,LOC100072221,LOC100072206,LOC100072211,LOC100058393
01	155795029	156657881	862853	1	14	LOC100072296,LOC100072258,LOC100072414,LOC100072179,LOC100072447,LOC100072426,LOC100072215,LOC100072251,LOC100058354,LOC100072237,LOC100072429,LOC100072368,LOC100072386,LOC100072192,LOC100072351,LOC100072271,LOC100072432,LOC100072275,LOC100072202,LOC100072265,LOC100072279,LOC100072306,LOC100072261,LOC100072184,LOC100072398,LOC100072342,LOC100072288,LOC100072317,LOC100072300,LOC100072380,LOC100072227,LOC100072195,LOC100072365,LOC100072443,LOC100058435,LOC100072402,LOC100072282,LOC100072290,LOC100072423,LOC100072320,LOC100072338,LOC100072221,LOC100072392,LOC100072354,LOC100072404,LOC100146987,LOC100072206,LOC100072211,LOC100072325,LOC100058393,LOC100072375,LOC100072345,LOC100072408,LOC100072254,LOC100072330,LOC100072335,LOC100072245
01	155954895	156358692	403798	1	2	LOC100072296,LOC100072258,LOC100072251,LOC100072237,LOC100072271,LOC100072275,LOC100072265,LOC100072279,LOC100072306,LOC100072261,LOC100072342,LOC100072288,LOC100072317,LOC100072300,LOC100072227,LOC100072282,LOC100072290,LOC100072320,LOC100072338,LOC100072221,LOC100146987,LOC100072325,LOC100072345,LOC100072254,LOC100072330,LOC100072335,LOC100072245
01	156002161	156462763	460603	1	1	LOC100072296,LOC100072258,LOC100072251,LOC100072237,LOC100072368,LOC100072386,LOC100072351,LOC100072271,LOC100072275,LOC100072265,LOC100072279,LOC100072306,LOC100072261,LOC100072342,LOC100072288,LOC100072317,LOC100072300,LOC100072380,LOC100072365,LOC100072282,LOC100072290,LOC100072320,LOC100072338,LOC100072354,LOC100146987,LOC100072325,LOC100072375,LOC100072345,LOC100072254,LOC100072330,LOC100072335,LOC100072245
01	156125915	156425245	299331	1	1	LOC100072296,LOC100072368,LOC100072351,LOC100072306,LOC100072342,LOC100072288,LOC100072317,LOC100072300,LOC100072365,LOC100072290,LOC100072320,LOC100072338,LOC100072354,LOC100146987,LOC100072325,LOC100072345,LOC100072330,LOC100072335
01	156125915	156449080	323166	1	2	LOC100072296,LOC100072368,LOC100072351,LOC100072306,LOC100072342,LOC100072288,LOC100072317,LOC100072300,LOC100072380,LOC100072365,LOC100072290,LOC100072320,LOC100072338,LOC100072354,LOC100146987,LOC100072325,LOC100072375,LOC100072345,LOC100072330,LOC100072335

Chrom	Start	End	Size	Cn	Samples (n)	Genes
01	156125915	156462763	336849	1	3	LOC100072296,LOC100072368,LOC100072386,LOC100072351,LOC100072306,LOC100072342,LOC100072288,LOC100072317,LOC100072300,LOC100072380,LOC100072365,LOC100072290,LOC100072320,LOC100072338,LOC100072354,LOC100146987,LOC100072325,LOC100072375,LOC100072345,LOC100072330,LOC100072335
01	156125915	156491374	365460	1	6	LOC100072296,LOC100072368,LOC100072386,LOC100072351,LOC100072306,LOC100072398,LOC100072342,LOC100072288,LOC100072317,LOC100072300,LOC100072380,LOC100072365,LOC100072290,LOC100072320,LOC100072338,LOC100072392,LOC100072354,LOC100146987,LOC100072325,LOC100072375,LOC100072345,LOC100072330,LOC100072335
01	156358067	156425245	67179	1	1	LOC100072368,LOC100072351,LOC100072365,LOC100072354
01	156358067	156449080	91014	1	2	LOC100072368,LOC100072351,LOC100072380,LOC100072365,LOC100072354,LOC100072375
01	156358067	156462763	104697	1	4	LOC100072368,LOC100072386,LOC100072351,LOC100072380,LOC100072365,LOC100072354,LOC100072375
01	156358067	156491374	133308	0,1	4,8	LOC100072368,LOC100072386,LOC100072351,LOC100072398,LOC100072380,LOC100072365,LOC100072392,LOC100072354,LOC100072375
01	156358067	156529837	171771	1	1	LOC100072368,LOC100072386,LOC100072351,LOC100072398,LOC100072380,LOC100072365,LOC100072402,LOC100072392,LOC100072354,LOC100072404,LOC100072375,LOC100072408
01	156358067	156657881	299815	1	2	LOC100072414,LOC100072447,LOC100072426,LOC100072429,LOC100072368,LOC100072386,LOC100072351,LOC100072432,LOC100072398,LOC100072380,LOC100072365,LOC100072443,LOC100058435,LOC100072402,LOC100072423,LOC100072392,LOC100072354,LOC100072404,LOC100072375,LOC100072408
01	156425245	156491374	66130	1	1	LOC100072386,LOC100072398,LOC100072380,LOC100072392,LOC100072375
01	156425245	156657881	232637	1	2	LOC100072414,LOC100072447,LOC100072426,LOC100072429,LOC100072386,LOC100072432,LOC100072398,LOC100072380,LOC100072443,LOC100058435,LOC100072402,LOC100072423,LOC100072392,LOC100072404,LOC100072375,LOC100072408
01	156425245	156870455	445211	1	1	LOC100072414,LOC100072447,LOC100072426,LOC100072429,LOC100072386,LOC100072432,LOC100072464,LOC100072493,LOC100072499,LOC100072398,LOC100072380,LOC100072469,LOC100629829,LOC100072443,LOC100058435,LOC100072495,LOC100072402,LOC100072423,LOC100072392,LOC100072404,LOC100072457,LOC100072479,LOC100072473,LOC100072375,LOC100072408,LOC100072489,LOC100072477,LOC100072452
01	156449080	156633245	184166	0	1	LOC100072414,LOC100072426,LOC100072429,LOC100072386,LOC100072432,LOC100072398,LOC100072443,LOC100072402,LOC100072423,LOC100072392,LOC100072404,LOC100072408
01	156449080	156657881	208802	0	1	LOC100072414,LOC100072447,LOC100072426,LOC100072429,LOC100072386,LOC100072432,LOC100072398,LOC100072443,LOC100058435,LOC100072402,LOC100072423,LOC100072392,LOC100072404,LOC100072408
01	156449080	156870455	421376	1	10	LOC100072414,LOC100072447,LOC100072426,LOC100072429,LOC100072386,LOC100072432,LOC100072464,LOC100072493,LOC100072499,LOC100072398,LOC100072469,LOC100629829,LOC100072443,LOC100058435,LOC100072495,LOC100072402,LOC100072423,LOC100072392,LOC100072404,LOC100072457,LOC100072479,LOC100072473,LOC100072408,LOC100072489,LOC100072477,LOC100072452
01	156462763	156818876	356114	1	1	LOC100072414,LOC100072447,LOC100072426,LOC100072429,LOC100072386,LOC100072432,LOC100072464,LOC100072398,LOC100072469,LOC100629829,LOC100072443,LOC100058435,LOC100072402,LOC100072423,LOC100072392,LOC100072404,LOC100072457,LOC100072479,LOC100072473,LOC100072408,LOC100072489,LOC100072477,LOC100072452
01	156529837	156870455	340619	1	4	LOC100072414,LOC100072447,LOC100072426,LOC100072429,LOC100072432,LOC100072464,LOC100072493,LOC100072499,LOC100072469,LOC100629829,LOC100072443,LOC100058435,LOC100072495,LOC100072423,LOC100072457,LOC100072479,LOC100072473,LOC100072489,LOC100072477,LOC100072452
01	156599954	156657881	57928	1	1	LOC100072447,LOC100072429,LOC100072432,LOC100072443,LOC100058435
01	156599954	156870455	270502	1	2	LOC100072447,LOC100072429,LOC100072432,LOC100072464,LOC100072493,LOC100072499,LOC100072469,LOC100629829,LOC100072443,LOC100058435,LOC100072495,LOC100072457,LOC100072479,LOC100072473,LOC100072489,LOC100072477,LOC100072452
01	156681241	156870455	189215	1	1	LOC100072464,LOC100072493,LOC100072499,LOC100072469,LOC100629829,LOC100072495,LOC100072457,LOC100072479,LOC100072473,LOC100072489,LOC100072477
01	159737598	159749634	12037	1	1	LOC100059022
01	165665636	165970661	305026	1	1	
01	165794587	165970661	176075	1	2	
01	177569314	177705794	136481	1	1	
01	178553782	178573079	19298	1	11	
01	178553782	178815370	261589	1	1	LOC100062668,LOC100629700
01	178553782	178844926	291145	1	1	LOC100062668,LOC100629700
01	178798269	179550475	752207	3	2	LOC100062807,LOC100062668,LOC100062944
01	178918130	179201495	283366	3	1	LOC100062807
01	178918130	179550475	632346	3	1	LOC100062807,LOC100062944
02	8599087	8617076	17990	1	2	FAF1
02	8764738	8799642	34905	1	1	
02	14978295	15080303	102009	3	1	WDR65,LOC100066676,LOC100147050
02	15112913	15284362	171450	1	1	LOC100629289,LOC100066795,LOC100629267,LOC100053497,LOC100066777
02	32764248	32866672	102425	3	1	USP48
02	44732196	44928737	196542	4	1	

Chrom	Start	End	Size	Cn	Samples (n)	Genes
02	48153515	48240185	86671	1	1	LOC100629449,LOC100065860,LOC100629488,LOC100147581
02	61224023	61293831	69809	1	1	
02	64286359	64527025	240667	1	1	
02	64513781	64527025	13245	1	1	
02	64513781	64615153	101373	1	1	
02	65482678	65490796	8119	1	1	CLCN3
02	70212772	70370275	157504	1	1	
02	70212772	70423370	210599	1	1	
02	70367017	70370275	3259	1	1	
02	70367017	70423370	56354	1	3	
02	70367017	70583259	216243	1	1	
02	72938406	72938869	464	3	1	
02	76068326	76116760	48435	3	1	TMEM144
02	76515324	76600049	84726	1	1	
02	77047746	77118167	70422	3	1	GLRB
02	82719505	82814630	95126	1	1	
02	84190834	84247510	56677	3	6	
02	84564860	84565102	243	1	2	
02	85975013	85991799	16787	1	1	TTC29
02	85975013	86065382	90370	3	1	TTC29
02	89616155	89978688	362534	3	1	LOC100063008,IL15,INPP4B
02	92665784	92833858	168075	3	1	
02	93690282	93886796	196515	1	1	
02	93739649	93886796	147148	1	1	
02	96664019	96701792	37774	1,3	2,5	
02	97634986	97689054	54069	1	1	LOC100629764
02	98566988	98861186	294199	3	1	
02	101889589	101930407	40819	3	2	
02	102400180	102426116	25937	1	2	
02	102400180	102484910	84731	1	2	
02	103510664	103613553	102890	3	1	
02	105110931	105238708	127778	3	1	SPATA5
02	105900566	106064917	164352	3	1	LOC100063889
02	106062109	106063373	1265	0,1	5,1	
02	107853583	107924442	70860	3	1	
02	110159273	110161760	2488	3	1	
02	110366126	110551367	185242	3	1	
02	110411873	110669459	257587	1	1	
02	110499252	110551367	52116	1	1	
02	110550935	110669459	118525	1	1	
02	110669459	110703036	33578	3	1	
02	111530939	111554376	23438	1	1	
02	116255680	116795535	539856	3	1	COL25A1
02	119636102	119721490	85389	3	1	
03	3413379	3417297	3919	3	2	BRD7
03	20446074	20490494	44421	1	1	
03	32241051	32376517	135467	3	1	
03	36595110	36614830	19721	1	2	
03	41567820	41635136	67317	3	4	
03	41621380	41635136	13757	3	2	
03	45062312	45089524	27213	1	2	GRID2

Chrom	Start	End	Size	Cn	Samples (n)	Genes
03	49581906	49650451	68546	1	1	PPM1K
03	49641059	49650451	9393	1	1	
03	52066736	52129820	63085	1	1	
03	60843942	60851963	8022	1	1	
03	65705932	65951800	245869	1	1	LOC100067903,LOC100066472,LOC100629168,LOC100067952,LOC100067974,LOC100067994,LOC100629252,LOC100629210,LOC100066501,LOC100068012
03	65932710	65951800	19091	1,3	9,21	LOC100066472,LOC100629252,LOC100066501
03	65932710	66015792	83083	3	1	LOC100066472,LOC100067777,LOC100629252,LOC100066501
03	65932710	66065643	132934	3	1	LOC100066444,LOC100066472,LOC100067777,LOC100629252,LOC100066501
03	70630601	70706642	76042	3	1	
03	70951405	70967718	16314	3	2	
03	72443473	72464566	21094	1	1	
03	74216796	74495490	278695	1	1	
03	74216796	74557812	341017	1	1	
03	75235988	75491946	255959	1	2	
03	75387562	75491946	104385	1	1	
03	91213313	91353260	139948	1	1	
03	95644115	95655868	11754	3	1	
03	107227161	107259452	32292	3	1	
03	114602741	114825361	222621	1	3	TBC1D14,LOC100056560
04	9121425	9500292	378868	1	5	LOC100062122,LOC100061824,LOC100629239,LOC100061654
04	9323693	9500292	176600	1	1	LOC100062122
04	9374294	9500292	125999	1	7	LOC100062122
04	9404330	9500292	95963	1	1	LOC100062122
04	12150224	12290949	140726	1	1	
04	12261477	12290949	29473	1	1	
04	12261477	12316520	55044	1	1	
04	22802309	23011397	209089	1	1	LOC100630459
04	22821965	23011397	189433	1	1	LOC100630459
04	23011397	23158551	147155	1	3	
04	25711112	25782678	71567	1	2	
04	33704681	33747940	43260	1	1	
04	33736922	33747940	11019	1	2	
04	33736922	33917967	181046	1	1	
04	33917967	34041290	123324	1	1	LOC100630646
04	37656221	37658259	2039	1	1	
04	46158925	46362093	203169	1	1	
04	50933745	50935536	1792	1	1	LOC100053170
04	52424614	52612016	187403	3	1	LOC100067077
04	52753250	52832948	79699	1	1	ABCB5,LOC100067191
04	53117054	53148501	31448	1	1	
04	55372175	55524405	152231	1	1	
04	82490516	82490652	137	1	1	GRM8
04	82490516	82508616	18101	1	3	GRM8
04	85453445	85457390	3946	1,3	1,1	
04	94580249	94633304	53056	3	5	LOC100064541,LOC100629938
04	96985336	97011232	25897	1,3	3,74	LOC100057252
04	96985336	97073291	87956	3	1	LOC100057334,LOC100057377,LOC100057252,LOC100057296
04	97007556	97073291	65736	3	1	LOC100057334,LOC100057377,LOC100057296
04	97073291	97192480	119190	3	3	LOC100057695,LOC100057617,LOC100057534,LOC100051071,LOC100057459,LOC100057655,LOC100147477,LOC100051145,LOC100630419,LOC100057574,LOC100146500
04	97123353	97192480	69128	3	4	LOC100057695,LOC100057617,LOC100057655,LOC100147477,LOC100051145,LOC100057574,LOC100146500
04	97565169	97574102	8934	1	1	LOC100058622

Chrom	Start	End	Size	Cn	Samples (n)	Genes
04	99899887	99923181	23295	3	1	CNTNAP2
04	101007622	101028581	20960	1	1	
04	101271134	101326718	55585	1	1	ZNF212,LOC100060969
04	101933836	102186782	252947	3	1	LOC100629360,LOC100146699,LOC100054458,GIMAP8,LOC100063236
05	3543345	3592298	48954	1	1	LOC100056344,LOC100056516
05	26674227	26684633	10407	3	1	
05	26674227	26698540	24314	3	3	
05	27420854	27427012	6159	1	1	KCNH1
05	28782503	28814723	32221	1	1	MIR205
05	37840041	37916448	76408	0	5	LOC100053475,LOC100057667
05	47239959	47259141	19183	1	1	LOC100068789
05	47359920	47399241	39322	3	6	TRNAG-CCC,LOC100059796
05	47398931	47558116	159186	3	1	TRNAN-GUU,LOC100059700,TRNAH-GUG,TRNAN-GUU,TRNAH-GUG,TRNAG-CCC,LOC100059796,TRNAE-UUC,TRNAG-CCC,TRNAE-UUC,LOC100059737,LOC100054164
05	52955124	53121994	166871	1	1	SLC22A15,LOC100059622
05	56110634	56177960	67327	3	2	
05	57754756	57771674	16919	1	1	
05	61640013	61889337	249325	1	1	
05	61877738	61889337	11600	1	2	
05	63904057	63920127	16071	1	4	
05	67881346	67955222	73877	1	1	DPYD
05	67881346	68001725	120380	1	1	DPYD
05	73047724	73094727	47004	1	2	
05	73582972	73620324	37353	3	6	ZNF644
05	74707555	74716431	8877	1	1	LOC100630850
05	77418462	77435333	16872	1	5	CLCA4
05	80420548	80539691	119144	1	1	
05	83401621	83405868	4248	1	3	
05	88243192	88258862	15671	3	27	
05	88243192	88327924	84733	3	3	
05	88243192	88349479	106288	3	1	
05	91661740	91761019	99280	1	1	LRRC7
05	99595549	99631010	35462	3,4	2,6	LOC100070758
06	4565099	4690824	125726	1	1	LOC100050318
06	4645237	4690824	45588	1	2	LOC100050318
06	14212417	14306931	94515	1	1	
06	26086675	26126581	39907	3	3	LOC100067436,LOC100147336
06	26102028	26126581	24554	3	1	LOC100067436
06	26104234	26109288	5055	1	2	
06	26104234	26118925	14692	1	4	LOC100067436
06	26104234	26126581	22348	1,3	1,1	LOC100067436
06	31779824	31877018	97195	3	3	
06	36433011	36436454	3444	1	1	LOC100061827
06	36748414	36794589	46176	1	1	LOC100053615
06	37137122	37433398	296277	1	2	LOC100053808,LOC100053863,LOC100062540,LOC100053911,LOC100062403,LOC100062370
06	37137122	37550849	413728	1	1	LOC100053808,LOC100062618,LOC100053863,LOC100062540,LOC100053963,LOC100053911,LOC100062403,LOC100062370
06	38780683	38906872	126190	1	1	LOC100065343,LOC100065376,LOC100065445,LOC100065421,LOC100146813,LOC100065399
06	71872446	72493903	621458	1	1	LOC100053971,LOC100054452,LOC100054400,LOC100630091,LOC100050632,LOC100055222,LOC100055352,LOC100055645,LOC100055600,LOC100055986,LOC100055946,LOC100055778,LOC100146480,LOC100054497,LOC100054585,LOC100050555,LOC100054214,LOC100054306,LOC100054063,LOC100054671,LOC100147637,LOC100054771,LOC100055272,LOC100146474,LOC100055559,LOC100055434,LOC100055688,LOC100055866,LOC100055818,LOC100055734,LOC100050712,LOC100055520,LOC100147260,LOC100055477,LOC100055308,LOC100055176,LOC100055082,LOC100055039,LOC100054995,LOC100054952,LOC100054904,LOC100054856,LOC100054816,LOC100146580,LOC100054626,LOC100054539,LOC100054354,LOC100054256,LOC100054118

Chrom	Start	End	Size	Cn	Samples (n)	Genes
06	72032729	72101344	68616	0,1	8,9	LOC100630091,LOC100054585,LOC100050555,LOC100054671,LOC100054771,LOC100146580,LOC100054626
06	72032729	72127862	95134	1	2	LOC100630091,LOC100054585,LOC100050555,LOC100054671,LOC100054771,LOC100054904,LOC100054856,LOC100054816,LOC100146580,LOC100054626
06	72032729	72423001	390273	1	1	LOC100630091,LOC100050632,LOC100055222,LOC100055352,LOC100055645,LOC100055600,LOC100055778,LOC100054585,LOC100050555,LOC100054671,LOC100147637,LOC100054771,LOC100055272,LOC100146474,LOC100055559,LOC100055434,LOC100055688,LOC100055734,LOC100050712,LOC100055520,LOC100147260,LOC100055477,LOC100055308,LOC100055176,LOC100055082,LOC100055039,LOC100054995,LOC100054952,LOC100054904,LOC100054856,LOC100054816,LOC100146580,LOC100054626
06	72032729	72485833	453105	1	2	LOC100630091,LOC100050632,LOC100055222,LOC100055352,LOC100055645,LOC100055600,LOC100055946,LOC100055778,LOC100146480,LOC100054585,LOC100050555,LOC100054671,LOC100147637,LOC100054771,LOC100055272,LOC100146474,LOC100055559,LOC100055434,LOC100055688,LOC100055866,LOC100055818,LOC100055734,LOC100050712,LOC100055520,LOC100147260,LOC100055477,LOC100055308,LOC100055176,LOC100055082,LOC100055039,LOC100054995,LOC100054952,LOC100054904,LOC100054856,LOC100054816,LOC100146580,LOC100054626
06	72032729	72493903	461175	1	55	LOC100630091,LOC100050632,LOC100055222,LOC100055352,LOC100055645,LOC100055600,LOC100055986,LOC100055946,LOC100055778,LOC100146480,LOC100054585,LOC100050555,LOC100054671,LOC100147637,LOC100054771,LOC100055272,LOC100146474,LOC100055559,LOC100055434,LOC100055688,LOC100055866,LOC100055818,LOC100055734,LOC100050712,LOC100055520,LOC100147260,LOC100055477,LOC100055308,LOC100055176,LOC100055082,LOC100055039,LOC100054995,LOC100054952,LOC100054904,LOC100054856,LOC100054816,LOC100146580,LOC100054626
06	72032729	72607543	574815	1	2	LOC100630091,LOC100050632,LOC100055222,LOC100055352,LOC100055645,LOC100055600,LOC100055986,LOC100055946,LOC100055778,LOC100146480,LOC100054585,LOC100050555,LOC100054671,LOC100147637,LOC100054771,LOC100055272,LOC100146474,LOC100055559,LOC100055434,LOC100055688,LOC100056234,LOC100056025,LOC100056186,LOC100630654,LOC100056100,LOC100055866,LOC100055818,LOC100055734,LOC100050712,LOC100055520,LOC100147260,LOC100055477,LOC100055308,LOC100055176,LOC100055082,LOC100055039,LOC100054995,LOC100054952,LOC100054904,LOC100054856,LOC100054816,LOC100146580,LOC100054626
06	72032729	72655562	622834	1	1	LOC100630091,LOC100050632,LOC100055222,LOC100055352,LOC100055645,LOC100055600,LOC100055986,LOC100146967,LOC100055946,LOC100056394,LOC100146670,LOC100055778,LOC100146480,LOC100054585,LOC100050555,LOC100054671,LOC100147637,LOC100054771,LOC100055272,LOC100146474,LOC100055559,LOC100055434,LOC100055688,LOC100056234,LOC100056025,LOC100056438,LOC100056355,LOC100056186,LOC100630654,LOC100056100,LOC100055866,LOC100055818,LOC100055734,LOC100050712,LOC100055520,LOC100147260,LOC100055477,LOC100055308,LOC100055176,LOC100055082,LOC100055039,LOC100054995,LOC100054952,LOC100054904,LOC100054856,LOC100054816,LOC100146580,LOC100054626
06	72325156	72485833	160678	0	1	LOC100055645,LOC100055600,LOC100055946,LOC100055778,LOC100146480,LOC100055559,LOC100055688,LOC100055866,LOC100055818,LOC100055734,LOC100050712,LOC100055520,LOC100147260
06	72325156	72493903	168748	0,1	8,21	LOC100055645,LOC100055600,LOC100055986,LOC100055946,LOC100055778,LOC100146480,LOC100055559,LOC100055688,LOC100055866,LOC100055818,LOC100055734,LOC100050712,LOC100055520,LOC100147260
06	72325156	72607543	282388	1	3	LOC100055645,LOC100055600,LOC100055986,LOC100055946,LOC100055778,LOC100146480,LOC100055559,LOC100055688,LOC100056234,LOC100056025,LOC100056186,LOC100630654,LOC100056100,LOC100055866,LOC100055818,LOC100055734,LOC100050712,LOC100055520,LOC100147260
06	72422847	72493903	71057	1	5	LOC100055986,LOC100055946,LOC100146480,LOC100055866,LOC100055818
06	72467998	72493903	25906	1	2	LOC100055986,LOC100055946
06	73072905	73412971	340067	1	1	LOC100057669,LOC100057893,LOC100057944,LOC100058303,LOC100051162,LOC100058635,LOC100058428,LOC100058516,LOC100057754,LOC100057803,LOC100057708,LOC100058076,LOC100057988,LOC100058345,LOC100058592,LOC100051089,LOC100058554,LOC100058471,LOC100058384,LOC100058210,LOC100058122,LOC100058035,LOC100057844,LOC100051018,LOC100146966
06	76052053	76100416	48364	1	1	
06	78531248	78554493	23246	3	2	LOC100629297
06	78531248	78679476	148229	3	2	LOC100629297
07	254594	747210	492617	1	1	ATP8B3,LOC100068855,IRF4,TRNAN-GUU,LOC100068840,LOC100146835,REXO1,TCF3,LOC100147519,MIR1905C,LOC100629298,LOC100060547,LOC100060620,LOC100146126,LOC100068825,TRNAF-GAA,STK11,LOC100069627
07	2250740	2282493	31754	1	1	LOC100146445,LOC100146548,TJP3
07	2250740	2347660	96921	1	1	ATCAY,LOC100061536,LOC100146445,LOC100146548,TJP3
07	4852528	4885046	32519	1	1	INSR,LOC100066300
07	8140391	8160586	20196	1	1	
07	8475870	8584515	108646	1	1	
07	8765950	8871973	106024	1	1	
07	9959138	10363881	404744	1	1	CNTN5
07	25994637	25995528	892	1	1	
07	31406445	31520977	114533	1	6	LOC100071915,LOC100071904,LOC100071894,LOC100071910,LOC100630881
07	31406445	31529855	123411	1	6	LOC100071915,LOC100071904,LOC100071894,LOC100071920,LOC100071910,LOC100630881
07	31414203	31520977	106775	1	3	LOC100071915,LOC100071904,LOC100071910,LOC100630881
07	48219282	48281950	62669	1	1	

Chrom	Start	End	Size	Cn	Samples (n)	Genes
07	48219282	48314947	95666	1	1	
07	50650966	50689513	38548	3	2	
07	52574872	52654017	79146	3	1	LOC100146282,LOC100064235,LOC100064296
07	52574872	52739455	164584	3	1	LOC100146282,LOC100064357,LOC100055731,LOC100064235,LOC100064420,LOC100064296
07	52610482	52654017	43536	3	20	LOC100146282,LOC100064296
07	52610482	52667927	57446	3	2	LOC100146282,LOC100064357,LOC100064296
07	52610482	52677786	67305	3	6	LOC100146282,LOC100064357,LOC100064296
07	52654017	52667927	13911	3	1	LOC100064357
07	52654017	52677786	23770	3	5	LOC100064357
07	52667838	52677786	9949	3	2	
07	54612913	54876128	263216	1	1	MIR492-2
07	56458055	56473649	15595	3	1	NAALAD2
07	56458055	56540470	82416	3	2	LOC100059915,LOC100050258,NAALAD2
07	56458055	56549568	91514	3	1	LOC100059915,LOC100050258,NAALAD2
07	56961092	57006577	45486	3	1	NOX4
07	70677039	71547591	870553	1	1	LOC100052674,LOC100066065,LOC100629820,RNF121,LOC100066133,PDE2A,ARAP1,LOC100146693,LOC100052446,LOC100066161,NUMA1,LOC100066037,LOC100052779,LOC100066106,LOC100052723,LOC100066084,CLPB,MIR139,LOC100052563,FCHSD2
07	73083306	73197149	113844	0,1	1,10	LOC100146503,LOC100067901,LOC100067972,LOC100067873,LOC100068010,LOC100067992,LOC100067950,LOC100067928,LOC100147186,LOC100146803
07	74760403	74859899	99497	1	1	LOC100069580,LOC100069636,LOC100069607,LOC100069556,LOC100069568,LOC100069622,LOC100069592
07	77320673	77402189	81517	1	1	LOC100070786,LOC100070767,LOC100070780,LOC100070801
07	78761527	78813338	51812	1	1	WEE1,ZNF143
07	82561206	82582932	21727	1	1	
08	1291607	1347166	55560	3	1	SUSD2,GGT5,LOC100053375
08	2608511	2628522	20012	3	1	
08	2608511	2712678	104168	3	1	SMARCB1,LOC100056349,MMP11,LOC100056480
08	3565276	3821757	256482	3	1	LOC100630286,LOC100059976,PPM1F,LOC100630192,TOP3B,LOC100630176,LOC100630137,LOC100630122
08	3726675	3821757	95083	3	25	LOC100630286,LOC100059976,LOC100630192,LOC100630176
08	4280605	4430473	149869	1	5	LOC100062472
08	4391896	4430473	38578	1	5	LOC100062472
08	4391896	4559972	168077	1	1	LOC100062472
08	4430473	4618457	187985	1	1	LOC100062472
08	4430473	4621044	190572	1	9	LOC100062472
08	4430473	4646812	216340	1	2	LOC100062472
08	4537919	4559972	22054	1,3	3,3	LOC100062472
08	4537919	4621044	83126	0,1	3,26	LOC100062472
08	4537919	4646812	108894	1	2	LOC100062472
08	16085130	16171233	86104	1	2	NOS1
08	24030822	24188533	157712	3	2	LOC100146720
08	30392818	30454234	61417	1,3	1,1	
08	41644642	41869404	224763	1	1	
08	41810441	41869404	58964	3,4	5,1	
08	51463126	51551764	88639	1	1	
08	54569959	54577344	7386	1	1	NOL4
08	64161502	64316647	155146	1	1	SETBP1
08	65338188	65348845	10658	1	1	
08	72425385	72462720	37336	1,3	1,1	
08	81506440	81550319	43880	1	1	
08	82909949	83024184	114236	1	1	
08	87181631	87279745	98115	1	1	
09	8940014	8961820	21807	1	2	
09	8940014	9090594	150581	1	1	

Chrom	Start	End	Size	Cn	Samples (n)	Genes
09	20053796	20195268	141473	1	1	
09	29889627	29892897	3271	0,1	6,1	
09	31548212	31574969	26758	1	1	PXDNL
09	31574454	31574969	516	1	4	PXDNL
09	33119854	33223660	103807	1	1	SNTG1
09	43791469	43818643	27175	1	1	
09	44946791	45279882	333092	1	1	VPS13B,LOC100060745
09	50387215	50534782	147568	1	1	ZFPM2
09	50519474	50534782	15309	1	1	ZFPM2
09	50803693	50808048	4356	1	7	
09	50803693	51315697	512005	1	1	OXR1
09	50912634	51113455	200822	3	1	
09	56084522	56235306	150785	1	1	
09	57755597	57759678	4082	1,3	1,3	
09	57755597	57774233	18637	1	1	
09	69647250	69651580	4331	1	1	
09	70960794	71004855	44062	3	2	LOC100068406
09	72122855	72307507	184653	3	1	
09	81322899	81402542	79644	3	1	LOC100146865
09	82209483	82435310	225828	1	1	LOC100630449,LOC100146671,LOC100146864,LOC100065550,SCRIB,LOC100146581,LOC100146968,LOC100065757,TSTA3,LOC100629422
10	674485	1141923	467439	3	1	LOC100053533,LOC100053634
10	674485	1271225	596741	3	1	LOC100053533,LOC100053634
10	674485	1332070	657586	3	1	LOC100053533,LOC100053634
10	782630	854532	71903	1	1	
10	3446860	3506099	59240	1	1	
10	4537764	4630934	93171	3	1	PEPD
10	4609986	4630934	20949	3	1	PEPD
10	10907542	10947011	39470	1	1	LOC100146416
10	19222030	19264042	42013	1	1	LOC100051792,LOC100055398
10	26444526	26654044	209519	1	1	EQUCABV1R-PS912,LOC100630829,EQUCABV1R-PS911,LOC100061052,LOC100051465,LOC100060917,EQUCABV1R-PS910,EQUCABV1R907,EQUCABV1R906,LOC100060687
10	30642716	30827421	184706	3	1	LOC100068859
10	30764949	30807450	42502	3	1	
10	30764949	30827421	62473	3	2	
10	32447110	32562724	115615	1	1	
10	37512203	37545357	33155	1	1	
10	40559615	40590249	30635	1	1	
10	41893599	41936853	43255	1	1	
10	45385393	45521495	136103	3	1	
10	46693683	47152670	458988	1	1	LOC100065897
10	48866033	48869952	3920	1,3	4,1	
10	52948258	52949299	1042	1	1	
10	52948258	53015010	66753	1	1	
10	54390376	54399237	8862	3	1	
10	54541826	54658680	116855	1	1	
10	54651405	54686862	35458	3	1	
10	69104691	69110606	5916	3	5	
10	70245511	70369934	124424	1	1	CLVS2
10	73096930	73250750	153821	3	3	LOC100629154
10	77814178	77851298	37121	0	1	ENPP3
11	507104	780294	273191	3	1	LOC100056827,HEXDC,LOC100056149,NARF,TRNAM-CAU,FOXK2,WDR45L,LOC100056579,LOC100056107

Chrom	Start	End	Size	Cn	Samples (n)	Genes
11	551696	780294	228599	3	1	LOC100056827,HEXDC,LOC100056149,NARF,TRNAM-CAU,FOXK2,WDR45L,LOC100056579
11	635764	780294	144531	3	4	LOC100056827,HEXDC,LOC100056149,NARF,TRNAM-CAU,FOXK2
11	754643	780294	25652	3	3	LOC100056827,HEXDC
11	2517091	2521729	4639	3	3	
11	7919169	8092052	172884	3	1	
11	12719610	12722151	2542	3	1	
11	18675278	18692852	17575	3	1	
11	33248110	33269366	21257	3	7	TRIM37
11	33269366	33337791	68426	3	1	LOC100057236,LOC100057203,LOC100057162,MIR301A,MIR454,LOC100629882
11	34936481	34969115	32635	3	1	BCAS3
11	36260837	36330823	69987	1	1	ACACA,AATF
11	38064567	38168907	104341	1	2	
11	54640169	54651986	11818	1	1	
11	54640169	54714505	74337	1	5	
11	54640169	54812394	172226	1	1	
11	54640169	54929094	288926	1	1	
11	54645681	54651986	6306	1	3	
11	54645681	54714505	68825	1	5	
11	54645681	54812394	166714	1	3	
11	58604726	58707648	102923	3	1	ULK2,AKAP10
11	59722467	59737161	14695	1	1	LOC100050716
12	3910273	4038592	128320	1	1	
12	3994147	4038592	44446	1	2	
12	5552984	5637713	84730	1	1	LOC100630247
12	6059876	6109895	50020	1	1	LRRC4C
12	8174285	8177840	3556	1	1	
12	12333201	12524489	191289	3	6	LOC100630831,LOC100051236,LOC100146284,LOC100058772,LOC100058728,PTPRJ
12	12333201	12594142	260942	3	1	LOC100051664,LOC100051592,LOC100051520,LOC100051452,LOC100051381,LOC100050265,LOC100630831,LOC100051236,LOC100146284,LOC100058772,LOC100058728,PTPRJ
12	12346938	12594142	247205	3	1	LOC100051664,LOC100051592,LOC100051520,LOC100051452,LOC100051381,LOC100050265,LOC100630831,LOC100051236,LOC100146284,LOC100058772,LOC100058728
12	12524489	12829176	304688	1	1	LOC100052629,LOC100052515,LOC100052454,LOC100052398,LOC100052341,LOC100052217,LOC100052157,LOC100052096,LOC100052037,LOC100146189,LOC100051977,LOC100051914,LOC100146810,LOC100050328,LOC100051731,LOC100051664,LOC100051592,LOC100051520,LOC100051452,LOC100051381,LOC100050265,LOC100630831
12	12524489	12858531	334043	3	3	LOC100052732,LOC100050399,LOC100052683,LOC100052629,LOC100052515,LOC100052454,LOC100052398,LOC100052341,LOC100052217,LOC100052157,LOC100052096,LOC100052037,LOC100146189,LOC100051977,LOC100051914,LOC100146810,LOC100050328,LOC100051731,LOC100051664,LOC100051592,LOC100051520,LOC100051452,LOC100051381,LOC100050265,LOC100630831
12	12524489	13170959	646471	1,3,4	1,4,2	LOC100053820,LOC100053768,LOC100629822,LOC100053672,LOC100053625,LOC100053568,LOC100053527,LOC100147669,LOC100053479,LOC100053427,LOC100053382,LOC100053328,LOC100053284,LOC100053236,LOC100053185,LOC100053134,LOC100053085,LOC100053045,LOC100052992,LOC100146502,LOC100052946,LOC100146388,LOC100052842,LOC100147383,LOC100052788,LOC100052732,LOC100050399,LOC100052683,LOC100052629,LOC100052515,LOC100052454,LOC100052398,LOC100052341,LOC100052217,LOC100052157,LOC100052096,LOC100052037,LOC100146189,LOC100051977,LOC100051914,LOC100146810,LOC100050328,LOC100051731,LOC100051664,LOC100051592,LOC100051520,LOC100051452,LOC100051381,LOC100050265,LOC100630831
12	12524489	13364132	839644	4	1	LOC100054215,LOC100054169,LOC100054120,LOC100054065,LOC100054021,LOC100050556,LOC100053872,LOC100050476,LOC100053820,LOC100053768,LOC100629822,LOC100053672,LOC100053625,LOC100053568,LOC100053527,LOC100147669,LOC100053479,LOC100053427,LOC100053382,LOC100053328,LOC100053284,LOC100053236,LOC100053185,LOC100053134,LOC100053085,LOC100053045,LOC100052992,LOC100146502,LOC100052946,LOC100146388,LOC100052842,LOC100147383,LOC100052788,LOC100052732,LOC100050399,LOC100052683,LOC100052629,LOC100052515,LOC100052454,LOC100052398,LOC100052341,LOC100052217,LOC100052157,LOC100052096,LOC100052037,LOC100146189,LOC100051977,LOC100051914,LOC100146810,LOC100050328,LOC100051731,LOC100051664,LOC100051592,LOC100051520,LOC100051452,LOC100051381,LOC100050265,LOC100630831

Chrom	Start	End	Size	Cn	Samples (n)	Genes
12	12524489	13401991	877503	3,4	1,1	LOC100146383,LOC100054355,LOC100054307,LOC100054258,LOC100054215,LOC100054169,LOC100054120,LOC100054065,LOC100054021,LOC100050556,LOC100053872,LOC100050476,LOC100053820,LOC100053768,LOC100629822,LOC100053672,LOC100053625,LOC100053568,LOC100053527,LOC100147669,LOC100053479,LOC100053427,LOC100053382,LOC100053328,LOC100053284,LOC100053236,LOC100053185,LOC100053134,LOC100053085,LOC100053045,LOC100052992,LOC100146502,LOC100052946,LOC100146388,LOC100052842,LOC100147383,LOC100052788,LOC100052732,LOC100050399,LOC100052683,LOC100052629,LOC100052515,LOC100052454,LOC100052398,LOC100052341,LOC100052217,LOC100052157,LOC100052096,LOC100052037,LOC100146189,LOC100051977,LOC100051914,LOC100146810,LOC100050328,LOC100051731,LOC100051664,LOC100051592,LOC100051520,LOC100051452,LOC100051381,LOC100050265,LOC100630831
12	12524489	13422256	897768	3	2	LOC100054453,LOC100146383,LOC100054355,LOC100054307,LOC100054258,LOC100054215,LOC100054169,LOC100054120,LOC100054065,LOC100054021,LOC100050556,LOC100053872,LOC100050476,LOC100053820,LOC100053768,LOC100629822,LOC100053672,LOC100053625,LOC100053568,LOC100053527,LOC100147669,LOC100053479,LOC100053427,LOC100053382,LOC100053328,LOC100053284,LOC100053236,LOC100053185,LOC100053134,LOC100053085,LOC100053045,LOC100052992,LOC100146502,LOC100052946,LOC100146388,LOC100052842,LOC100147383,LOC100052788,LOC100052732,LOC100050399,LOC100052683,LOC100052629,LOC100052515,LOC100052454,LOC100052398,LOC100052341,LOC100052217,LOC100052157,LOC100052096,LOC100052037,LOC100146189,LOC100051977,LOC100051914,LOC100146810,LOC100050328,LOC100051731,LOC100051664,LOC100051592,LOC100051520,LOC100051452,LOC100051381,LOC100050265,LOC100630831
12	12524489	13439146	914658	3	1	LOC100054498,LOC100054453,LOC100146383,LOC100054355,LOC100054307,LOC100054258,LOC100054215,LOC100054169,LOC100054120,LOC100054065,LOC100054021,LOC100050556,LOC100053872,LOC100050476,LOC100053820,LOC100053768,LOC100629822,LOC100053672,LOC100053625,LOC100053568,LOC100053527,LOC100147669,LOC100053479,LOC100053427,LOC100053382,LOC100053328,LOC100053284,LOC100053236,LOC100053185,LOC100053134,LOC100053085,LOC100053045,LOC100052992,LOC100146502,LOC100052946,LOC100146388,LOC100052842,LOC100147383,LOC100052788,LOC100052732,LOC100050399,LOC100052683,LOC100052629,LOC100052515,LOC100052454,LOC100052398,LOC100052341,LOC100052217,LOC100052157,LOC100052096,LOC100052037,LOC100146189,LOC100051977,LOC100051914,LOC100146810,LOC100050328,LOC100051731,LOC100051664,LOC100051592,LOC100051520,LOC100051452,LOC100051381,LOC100050265,LOC100630831
12	12524489	13488187	963699	0	2	LOC100054673,LOC100054627,LOC100054586,LOC100054541,LOC100054498,LOC100054453,LOC100146383,LOC100054355,LOC100054307,LOC100054258,LOC100054215,LOC100054169,LOC100054120,LOC100054065,LOC100054021,LOC100050556,LOC100053872,LOC100050476,LOC100053820,LOC100053768,LOC100629822,LOC100053672,LOC100053625,LOC100053568,LOC100053527,LOC100147669,LOC100053479,LOC100053427,LOC100053382,LOC100053328,LOC100053284,LOC100053236,LOC100053185,LOC100053134,LOC100053085,LOC100053045,LOC100052992,LOC100146502,LOC100052946,LOC100146388,LOC100052842,LOC100147383,LOC100052788,LOC100052732,LOC100050399,LOC100052683,LOC100052629,LOC100052515,LOC100052454,LOC100052398,LOC100052341,LOC100052217,LOC100052157,LOC100052096,LOC100052037,LOC100146189,LOC100051977,LOC100051914,LOC100146810,LOC100050328,LOC100051731,LOC100051664,LOC100051592,LOC100051520,LOC100051452,LOC100051381,LOC100050265,LOC100630831
12	12524489	13573356	1048868	3	4	LOC100147187,LOC100054954,LOC100054906,LOC100054858,LOC100054773,LOC100054722,LOC100054673,LOC100054627,LOC100054586,LOC100054541,LOC100054498,LOC100054453,LOC100146383,LOC100054355,LOC100054307,LOC100054258,LOC100054215,LOC100054169,LOC100054120,LOC100054065,LOC100054021,LOC100050556,LOC100053872,LOC100050476,LOC100053820,LOC100053768,LOC100629822,LOC100053672,LOC100053625,LOC100053568,LOC100053527,LOC100147669,LOC100053479,LOC100053427,LOC100053382,LOC100053328,LOC100053284,LOC100053236,LOC100053185,LOC100053134,LOC100053085,LOC100053045,LOC100052992,LOC100146502,LOC100052946,LOC100146388,LOC100052842,LOC100147383,LOC100052788,LOC100052732,LOC100050399,LOC100052683,LOC100052629,LOC100052515,LOC100052454,LOC100052398,LOC100052341,LOC100052217,LOC100052157,LOC100052096,LOC100052037,LOC100146189,LOC100051977,LOC100051914,LOC100146810,LOC100050328,LOC100051731,LOC100051664,LOC100051592,LOC100051520,LOC100051452,LOC100051381,LOC100050265,LOC100630831
12	12524489	13678953	1154465	1	2	LOC100055353,LOC100055309,LOC100055273,LOC100055224,LOC100055177,LOC100055129,LOC100055083,LOC100052898,LOC100147187,LOC100054954,LOC100054906,LOC100054858,LOC100054773,LOC100054722,LOC100054673,LOC100054627,LOC100054586,LOC100054541,LOC100054498,LOC100054453,LOC100146383,LOC100054355,LOC100054307,LOC100054258,LOC100054215,LOC100054169,LOC100054120,LOC100054065,LOC100054021,LOC100050556,LOC100053872,LOC100050476,LOC100053820,LOC100053768,LOC100629822,LOC100053672,LOC100053625,LOC100053568,LOC100053527,LOC100147669,LOC100053479,LOC100053427,LOC100053382,LOC100053328,LOC100053284,LOC100053236,LOC100053185,LOC100053134,LOC100053085,LOC100053045,LOC100052992,LOC100146502,LOC100052946,LOC100146388,LOC100052842,LOC100147383,LOC100052788,LOC100052732,LOC100050399,LOC100052683,LOC100052629,LOC100052515,LOC100052454,LOC100052398,LOC100052341,LOC100052217,LOC100052157,LOC100052096,LOC100052037,LOC100146189,LOC100051977,LOC100051914,LOC100146810,LOC100050328,LOC100051731,LOC100051664,LOC100051592,LOC100051520,LOC100051452,LOC100051381,LOC100050265,LOC100630831
12	12524489	14108352	1583864	3	1	LOC100056531,LOC100056488,LOC100629158,LOC100050633,LOC100056395,LOC100056356,LOC100056314,LOC100056273,LOC100056235,LOC100056187,LOC100056146,LOC100630788,LOC100629923,LOC100056026,LOC100055988,LOC100146992,LOC100055947,LOC100147569,LOC100146290,LOC100055908,LOC100055867,LOC100055780,LOC100630577,LOC100055646,LOC100055601,LOC100055561,LOC100055521,LOC100055478,LOC100055435,LOC100055397,LOC100055353,LOC100055309,LOC100055273,LOC100055224,LOC100055177,LOC100055129,LOC100055083,LOC100052898,LOC100147187,LOC100054954,LOC100054906,LOC100054858,LOC100054773,LOC100054722,LOC100054673,LOC100054627,LOC100054586,LOC100054541,LOC100054498,LOC100054453,LOC100146383,LOC100054355,LOC100054307,LOC100054258,LOC100054215,LOC100054169,LOC100054120,LOC100054065,LOC100054021,LOC100050556,LOC100053872,LOC100050476,LOC100053820,LOC100053768,LOC100629822,LOC100053672,LOC100053625,LOC100053568,LOC100053527,LOC100147669,LOC100053479,LOC100053427,LOC100053382,LOC100053328,LOC100053284,LOC100053236,LOC100053185,LOC100053134,LOC100053085,LOC100053045,LOC100052992,LOC100146502,LOC100052946,LOC100146388,LOC100052842,LOC100147383,LOC100052788,LOC100052732,LOC100050399,LOC100052683,LOC100052629,LOC100052515,LOC100052454,LOC100052398,LOC100052341,LOC100052217,LOC100052157,LOC100052096,LOC100052037,LOC100146189,LOC100051977,LOC100051914,LOC100146810,LOC100050328,LOC100051731,LOC100051664,LOC100051592,LOC100051520,LOC100051452,LOC100051381,LOC100050265,LOC100630831

Chrom	Start	End	Size	Cn	Samples (n)	Genes
12	12524489	14128309	1603821	3	3	LOC100056626,LOC100056578,LOC100056531,LOC100056488,LOC100629158,LOC100050633,LOC100056395,LOC100056356,LOC100056314,LOC100056273,LOC100056235,LOC100056187,LOC100056146,LOC100630788,LOC100629923,LOC100056026,LOC100055988,LOC100146992,LOC100055947,LOC100147569,LOC100146290,LOC100055908,LOC100055867,LOC100055780,LOC100630577,LOC100055646,LOC100055601,LOC100055561,LOC100055521,LOC100055478,LOC100055435,LOC100055397,LOC100055353,LOC100055309,LOC100055273,LOC100055224,LOC100055177,LOC100055129,LOC100055083,LOC100052898,LOC100147187,LOC100054954,LOC100054906,LOC100054858,LOC100054773,LOC100054722,LOC100054673,LOC100054627,LOC100054586,LOC100054541,LOC100054498,LOC100054453,LOC100146383,LOC100054355,LOC100054307,LOC100054258,LOC100054215,LOC100054169,LOC100054120,LOC100054065,LOC100054021,LOC100050556,LOC100053872,LOC100050476,LOC100053820,LOC100053768,LOC100629822,LOC100053672,LOC100053625,LOC100053568,LOC100053527,LOC100147669,LOC100053479,LOC100053427,LOC100053382,LOC100053328,LOC100053284,LOC100053236,LOC100053185,LOC100053134,LOC100053085,LOC100053045,LOC100052992,LOC100146502,LOC100052946,LOC100146388,LOC100052842,LOC100147383,LOC100052788,LOC100052732,LOC100050399,LOC100052683,LOC100052629,LOC100052515,LOC100052454,LOC100052398,LOC100052341,LOC100052217,LOC100052157,LOC100052096,LOC100052037,LOC100146189,LOC100051977,LOC100051914,LOC100146810,LOC100050328,LOC100051731,LOC100051664,LOC100051592,LOC100051520,LOC100051452,LOC100051381,LOC100050265,LOC100630831
12	12524489	14354314	1829826	3,4	4,1	LOC100056947,LOC100056906,LOC100056869,LOC100146809,LOC100067978,LOC100067957,LOC100056826,LOC100056793,LOC100056755,LOC100147000,LOC100056708,LOC100056626,LOC100056578,LOC100056531,LOC100056488,LOC100629158,LOC100050633,LOC100056395,LOC100056356,LOC100056314,LOC100056273,LOC100056235,LOC100056187,LOC100056146,LOC100630788,LOC100629923,LOC100056026,LOC100055988,LOC100146992,LOC100055947,LOC100147569,LOC100146290,LOC100055908,LOC100055867,LOC100055780,LOC100630577,LOC100055646,LOC100055601,LOC100055561,LOC100055521,LOC100055478,LOC100055435,LOC100055397,LOC100055353,LOC100055309,LOC100055273,LOC100055224,LOC100055177,LOC100055129,LOC100055083,LOC100052898,LOC100147187,LOC100054954,LOC100054906,LOC100054858,LOC100054773,LOC100054722,LOC100054673,LOC100054627,LOC100054586,LOC100054541,LOC100054498,LOC100054453,LOC100146383,LOC100054355,LOC100054307,LOC100054258,LOC100054215,LOC100054169,LOC100054120,LOC100054065,LOC100054021,LOC100050556,LOC100053872,LOC100050476,LOC100053820,LOC100053768,LOC100629822,LOC100053672,LOC100053625,LOC100053568,LOC100053527,LOC100147669,LOC100053479,LOC100053427,LOC100053382,LOC100053328,LOC100053284,LOC100053236,LOC100053185,LOC100053134,LOC100053085,LOC100053045,LOC100052992,LOC100146502,LOC100052946,LOC100146388,LOC100052842,LOC100147383,LOC100052788,LOC100052732,LOC100050399,LOC100052683,LOC100052629,LOC100052515,LOC100052454,LOC100052398,LOC100052341,LOC100052217,LOC100052157,LOC100052096,LOC100052037,LOC100146189,LOC100051977,LOC100051914,LOC100146810,LOC100050328,LOC100051731,LOC100051664,LOC100051592,LOC100051520,LOC100051452,LOC100051381,LOC100050265,LOC100630831
12	12524489	14391372	1866884	1,3	39,2	LOC100057026,LOC100050714,LOC100056989,LOC100056947,LOC100056906,LOC100056869,LOC100146809,LOC100067978,LOC100067957,LOC100056826,LOC100056793,LOC100056755,LOC100147000,LOC100056708,LOC100056626,LOC100056578,LOC100056531,LOC100056488,LOC100629158,LOC100050633,LOC100056395,LOC100056356,LOC100056314,LOC100056273,LOC100056235,LOC100056187,LOC100056146,LOC100630788,LOC100629923,LOC100056026,LOC100055988,LOC100146992,LOC100055947,LOC100147569,LOC100146290,LOC100055908,LOC100055867,LOC100055780,LOC100630577,LOC100055646,LOC100055601,LOC100055561,LOC100055521,LOC100055478,LOC100055435,LOC100055397,LOC100055353,LOC100055309,LOC100055273,LOC100055224,LOC100055177,LOC100055129,LOC100055083,LOC100052898,LOC100147187,LOC100054954,LOC100054906,LOC100054858,LOC100054773,LOC100054722,LOC100054673,LOC100054627,LOC100054586,LOC100054541,LOC100054498,LOC100054453,LOC100146383,LOC100054355,LOC100054307,LOC100054258,LOC100054215,LOC100054169,LOC100054120,LOC100054065,LOC100054021,LOC100050556,LOC100053872,LOC100050476,LOC100053820,LOC100053768,LOC100629822,LOC100053672,LOC100053625,LOC100053568,LOC100053527,LOC100147669,LOC100053479,LOC100053427,LOC100053382,LOC100053328,LOC100053284,LOC100053236,LOC100053185,LOC100053134,LOC100053085,LOC100053045,LOC100052992,LOC100146502,LOC100052946,LOC100146388,LOC100052842,LOC100147383,LOC100052788,LOC100052732,LOC100050399,LOC100052683,LOC100052629,LOC100052515,LOC100052454,LOC100052398,LOC100052341,LOC100052217,LOC100052157,LOC100052096,LOC100052037,LOC100146189,LOC100051977,LOC100051914,LOC100146810,LOC100050328,LOC100051731,LOC100051664,LOC100051592,LOC100051520,LOC100051452,LOC100051381,LOC100050265,LOC100630831
12	12524489	14587232	2062744	1	1	LOC100057472,LOC100057433,LOC100057393,LOC100057352,LOC100057310,LOC100147286,LOC100146990,LOC100057269,LOC100057228,LOC100057191,LOC100057110,LOC100146600,LOC100057026,LOC100050714,LOC100056989,LOC100056947,LOC100056906,LOC100056869,LOC100146809,LOC100067978,LOC100067957,LOC100056826,LOC100056793,LOC100056755,LOC100147000,LOC100056708,LOC100056626,LOC100056578,LOC100056531,LOC100056488,LOC100629158,LOC100050633,LOC100056395,LOC100056356,LOC100056314,LOC100056273,LOC100056235,LOC100056187,LOC100056146,LOC100630788,LOC100629923,LOC100056026,LOC100055988,LOC100146992,LOC100055947,LOC100147569,LOC100146290,LOC100055908,LOC100055867,LOC100055780,LOC100630577,LOC100055646,LOC100055601,LOC100055561,LOC100055521,LOC100055478,LOC100055435,LOC100055397,LOC100055353,LOC100055309,LOC100055273,LOC100055224,LOC100055177,LOC100055129,LOC100055083,LOC100052898,LOC100147187,LOC100054954,LOC100054906,LOC100054858,LOC100054773,LOC100054722,LOC100054673,LOC100054627,LOC100054586,LOC100054541,LOC100054498,LOC100054453,LOC100146383,LOC100054355,LOC100054307,LOC100054258,LOC100054215,LOC100054169,LOC100054120,LOC100054065,LOC100054021,LOC100050556,LOC100053872,LOC100050476,LOC100053820,LOC100053768,LOC100629822,LOC100053672,LOC100053625,LOC100053568,LOC100053527,LOC100147669,LOC100053479,LOC100053427,LOC100053382,LOC100053328,LOC100053284,LOC100053236,LOC100053185,LOC100053134,LOC100053085,LOC100053045,LOC100052992,LOC100146502,LOC100052946,LOC100146388,LOC100052842,LOC100147383,LOC100052788,LOC100052732,LOC100050399,LOC100052683,LOC100052629,LOC100052515,LOC100052454,LOC100052398,LOC100052341,LOC100052217,LOC100052157,LOC100052096,LOC100052037,LOC100146189,LOC100051977,LOC100051914,LOC100146810,LOC100050328,LOC100051731,LOC100051664,LOC100051592,LOC100051520,LOC100051452,LOC100051381,LOC100050265, LOC100630831

Chrom	Start	End	Size	Cn	Samples (n)	Genes
12	12524489	14777981	2253493	1,3	2,2	LOC100058077,LOC100147282,LOC100058036,LOC100057989,LOC100057945,LOC100057894,LOC100057845,LOC100057804,LOC100057756,LOC100057709,LOC100057670,LOC100057589,LOC100057547,LOC100057513,LOC100057472,LOC100057433,LOC100057393,LOC100057352,LOC100057310,LOC100147286,LOC100146990,LOC100057269,LOC100057228,LOC100057191,LOC100057110,LOC100146600,LOC100057026,LOC100050714,LOC100056989,LOC100056947,LOC100056906,LOC100056869,LOC100146809,LOC100067978,LOC100067957,LOC100056826,LOC100056793,LOC100056755,LOC100147000,LOC100056708,LOC100056626,LOC100056578,LOC100056531,LOC100056488,LOC100629158,LOC100050633,LOC100056395,LOC100056356,LOC100056314,LOC100056273,LOC100056235,LOC100056187,LOC100056146,LOC100630788,LOC100629923,LOC100056026,LOC100055988,LOC100146992,LOC100055947,LOC100147569,LOC100146290,LOC100055908,LOC100055867,LOC100055780,LOC100630577,LOC100055646,LOC100055601,LOC100055561,LOC100055521,LOC100055478,LOC100055435,LOC100055397,LOC100055353,LOC100055309,LOC100055273,LOC100055224,LOC100055177,LOC100055129,LOC100055083,LOC100052898,LOC100147187,LOC100054954,LOC100054906,LOC100054858,LOC100054773,LOC100054722,LOC100054673,LOC100054627,LOC100054586,LOC100054541,LOC100054498,LOC100054453,LOC100146383,LOC100054355,LOC100054307,LOC100054258,LOC100054215,LOC100054169,LOC100054120,LOC100054065,LOC100054021,LOC100050556,LOC100053872,LOC100050476,LOC100053820,LOC100053768,LOC100629822,LOC100053672,LOC100053625,LOC100053568,LOC100053527,LOC100147669,LOC100053479,LOC100053427,LOC100053382,LOC100053328,LOC100053284,LOC100053236,LOC100053185,LOC100053134,LOC100053085,LOC100053045,LOC100052992,LOC100146502,LOC100052946,LOC100146388,LOC100052842,LOC100147383,LOC100052788,LOC100052732,LOC100050399,LOC100052683,LOC100052629,LOC100052515,LOC100052454,LOC100052398,LOC100052341,LOC100052217,LOC100052157,LOC100052096,LOC100052037,LOC100146189,LOC100051977,LOC100051914,LOC100146810,LOC100050328,LOC100051731,LOC100051664,LOC100051592,LOC100051520,LOC100051452,LOC100051381,LOC100050265,LOC10063083
12	12594142	14391372	1797231	1	2	LOC100057026,LOC100050714,LOC100056989,LOC100056947,LOC100056906,LOC100056869,LOC100146809,LOC100067978,LOC100067957,LOC100056826,LOC100056793,LOC100056755,LOC100147000,LOC100056708,LOC100056626,LOC100056578,LOC100056531,LOC100056488,LOC100629158,LOC100050633,LOC100056395,LOC100056356,LOC100056314,LOC100056273,LOC100056235,LOC100056187,LOC100056146,LOC100630788,LOC100629923,LOC100056026,LOC100055988,LOC100146992,LOC100055947,LOC100147569,LOC100146290,LOC100055908,LOC100055867,LOC100055780,LOC100630577,LOC100055646,LOC100055601,LOC100055561,LOC100055521,LOC100055478,LOC100055435,LOC100055397,LOC100055353,LOC100055309,LOC100055273,LOC100055224,LOC100055177,LOC100055129,LOC100055083,LOC100052898,LOC100147187,LOC100054954,LOC100054906,LOC100054858,LOC100054773,LOC100054722,LOC100054673,LOC100054627,LOC100054586,LOC100054541,LOC100054498,LOC100054453,LOC100146383,LOC100054355,LOC100054307,LOC100054258,LOC100054215,LOC100054169,LOC100054120,LOC100054065,LOC100054021,LOC100050556,LOC100053872,LOC100050476,LOC100053820,LOC100053768,LOC100629822,LOC100053672,LOC100053625,LOC100053568,LOC100053527,LOC100147669,LOC100053479,LOC100053427,LOC100053382,LOC100053328,LOC100053284,LOC100053236,LOC100053185,LOC100053134,LOC100053085,LOC100053045,LOC100052992,LOC100146502,LOC100052946,LOC100146388,LOC100052842,LOC100147383,LOC100052788,LOC100052732,LOC100050399,LOC100052683,LOC100052629,LOC100052515,LOC100052454,LOC100052398,LOC100052341,LOC100052217,LOC100052157,LOC100052096,LOC100052037,LOC100146189,LOC100051977,LOC100051914,LOC100146810,LOC100050328,LOC100051731,LOC100051664
12	12829176	12858531	29356	1,3	14,2	LOC100052732,LOC100050399,LOC100052683
12	12829176	13149957	320782	1,3	3,1	LOC100053672,LOC100053625,LOC100053568,LOC100053527,LOC100147669,LOC100053479,LOC100053427,LOC100053382,LOC100053328,LOC100053284,LOC100053236,LOC100053185,LOC100053134,LOC100053085,LOC100053045,LOC100052992,LOC100146502,LOC100052946,LOC100146388,LOC100052842,LOC100147383,LOC100052788,LOC100052732,LOC100050399,LOC100052683
12	12829176	13170959	341784	1,3	1,1	LOC100053820,LOC100053768,LOC100629822,LOC100053672,LOC100053625,LOC100053568,LOC100053527,LOC100147669,LOC100053479,LOC100053427,LOC100053382,LOC100053328,LOC100053284,LOC100053236,LOC100053185,LOC100053134,LOC100053085,LOC100053045,LOC100052992,LOC100146502,LOC100052946,LOC100146388,LOC100052842,LOC100147383,LOC100052788,LOC100052732,LOC100050399,LOC100052683
12	12829176	13401991	572816	3	4	LOC100146383,LOC100054355,LOC100054307,LOC100054258,LOC100054215,LOC100054169,LOC100054120,LOC100054065,LOC100054021,LOC100050556,LOC100053872,LOC100050476,LOC100053820,LOC100053768,LOC100629822,LOC100053672,LOC100053625,LOC100053568,LOC100053527,LOC100147669,LOC100053479,LOC100053427,LOC100053382,LOC100053328,LOC100053284,LOC100053236,LOC100053185,LOC100053134,LOC100053085,LOC100053045,LOC100052992,LOC100146502,LOC100052946,LOC100146388,LOC100052842,LOC100147383,LOC100052788,LOC100052732,LOC100050399,LOC100052683
12	12829176	13422256	593081	3	1	LOC100054453,LOC100146383,LOC100054355,LOC100054307,LOC100054258,LOC100054215,LOC100054169,LOC100054120,LOC100054065,LOC100054021,LOC100050556,LOC100053872,LOC100050476,LOC100053820,LOC100053768,LOC100629822,LOC100053672,LOC100053625,LOC100053568,LOC100053527,LOC100147669,LOC100053479,LOC100053427,LOC100053382,LOC100053328,LOC100053284,LOC100053236,LOC100053185,LOC100053134,LOC100053085,LOC100053045,LOC100052992,LOC100146502,LOC100052946,LOC100146388,LOC100052842,LOC100147383,LOC100052788,LOC100052732,LOC100050399,LOC100052683
12	12829176	13488187	659012	0	2	LOC100054673,LOC100054627,LOC100054586,LOC100054541,LOC100054498,LOC100054453,LOC100146383,LOC100054355,LOC100054307,LOC100054258,LOC100054215,LOC100054169,LOC100054120,LOC100054065,LOC100054021,LOC100050556,LOC100053872,LOC100050476,LOC100053820,LOC100053768,LOC100629822,LOC100053672,LOC100053625,LOC100053568,LOC100053527,LOC100147669,LOC100053479,LOC100053427,LOC100053382,LOC100053328,LOC100053284,LOC100053236,LOC100053185,LOC100053134,LOC100053085,LOC100053045,LOC100052992,LOC100146502,LOC100052946,LOC100146388,LOC100052842,LOC100147383,LOC100052788,LOC100052732,LOC100050399,LOC100052683
12	12829176	13573356	744181	3	1	LOC100147187,LOC100054954,LOC100054906,LOC100054858,LOC100054773,LOC100054722,LOC100054673,LOC100054627,LOC100054586,LOC100054541,LOC100054498,LOC100054453,LOC100146383,LOC100054355,LOC100054307,LOC100054258,LOC100054215,LOC100054169,LOC100054120,LOC100054065,LOC100054021,LOC100050556,LOC100053872,LOC100050476,LOC100053820,LOC100053768,LOC100629822,LOC100053672,LOC100053625,LOC100053568,LOC100053527,LOC100147669,LOC100053479,LOC100053427,LOC100053382,LOC100053328,LOC100053284,LOC100053236,LOC100053185,LOC100053134,LOC100053085,LOC100053045,LOC100052992,LOC100146502,LOC100052946,LOC100146388,LOC100052842,LOC100147383,LOC100052788,LOC100052732,LOC100050399,LOC100052683

Chrom	Start	End	Size	Cn	Samples (n)	Genes
12	12829176	14354314	1525139	3	1	LOC100056947,LOC100056906,LOC100056869,LOC100146809,LOC100067978,LOC100067957,LOC100056826,LOC100056793,LOC100056755,LOC100147000,LOC100056708,LOC100056626,LOC100056578,LOC100056531,LOC100056488,LOC100629158,LOC100050633,LOC100056395,LOC100056356,LOC100056314,LOC100056273,LOC100056235,LOC100056187,LOC100056146,LOC100630788,LOC100629923,LOC100056026,LOC100055988,LOC100146992,LOC100055947,LOC100147569,LOC100146290,LOC100055908,LOC100055867,LOC100055780,LOC100630577,LOC100055646,LOC100055601,LOC100055561,LOC100055521,LOC100055478,LOC100055435,LOC100055397,LOC100055353,LOC100055309,LOC100055273,LOC100055224,LOC100055177,LOC100055129,LOC100055083,LOC100052898,LOC100147187,LOC100054954,LOC100054906,LOC100054858,LOC100054773,LOC100054722,LOC100054673,LOC100054627,LOC100054586,LOC100054541,LOC100054498,LOC100054453,LOC100146383,LOC100054355,LOC100054307,LOC100054258,LOC100054215,LOC100054169,LOC100054120,LOC100054065,LOC100054021,LOC100050556,LOC100053872,LOC100050476,LOC100053820,LOC100053768,LOC100629822,LOC100053672,LOC100053625,LOC100053568,LOC100053527,LOC100147669,LOC100053479,LOC100053427,LOC100053382,LOC100053328,LOC100053284,LOC100053236,LOC100053185,LOC100053134,LOC100053085,LOC100053045,LOC100052992,LOC100146502,LOC100052946,LOC100146388,LOC100052842,LOC100147383,LOC100052788,LOC100052732,LOC100050399,LOC100052683
12	12829176	14391372	1562197	1,3	7,2	LOC100057026,LOC100050714,LOC100056989,LOC100056947,LOC100056906,LOC100056869,LOC100146809,LOC100067978,LOC100067957,LOC100056826,LOC100056793,LOC100056755,LOC100147000,LOC100056708,LOC100056626,LOC100056578,LOC100056531,LOC100056488,LOC100629158,LOC100050633,LOC100056395,LOC100056356,LOC100056314,LOC100056273,LOC100056235,LOC100056187,LOC100056146,LOC100630788,LOC100629923,LOC100056026,LOC100055988,LOC100146992,LOC100055947,LOC100147569,LOC100146290,LOC100055908,LOC100055867,LOC100055780,LOC100630577,LOC100055646,LOC100055601,LOC100055561,LOC100055521,LOC100055478,LOC100055435,LOC100055397,LOC100055353,LOC100055309,LOC100055273,LOC100055224,LOC100055177,LOC100055129,LOC100055083,LOC100052898,LOC100147187,LOC100054954,LOC100054906,LOC100054858,LOC100054773,LOC100054722,LOC100054673,LOC100054627,LOC100054586,LOC100054541,LOC100054498,LOC100054453,LOC100146383,LOC100054355,LOC100054307,LOC100054258,LOC100054215,LOC100054169,LOC100054120,LOC100054065,LOC100054021,LOC100050556,LOC100053872,LOC100050476,LOC100053820,LOC100053768,LOC100629822,LOC100053672,LOC100053625,LOC100053568,LOC100053527,LOC100147669,LOC100053479,LOC100053427,LOC100053382,LOC100053328,LOC100053284,LOC100053236,LOC100053185,LOC100053134,LOC100053085,LOC100053045,LOC100052992,LOC100146502,LOC100052946,LOC100146388,LOC100052842,LOC100147383,LOC100052788,LOC100052732,LOC100050399,LOC100052683
12	12829176	14777981	1948806	1,3	2,1	LOC100058077,LOC100147282,LOC100058036,LOC100057989,LOC100057945,LOC100057894,LOC100057845,LOC100057804,LOC100057756,LOC100057709,LOC100057670,LOC100057589,LOC100057547,LOC100057513,LOC100057472,LOC100057433,LOC100057393,LOC100057352,LOC100057310,LOC100147286,LOC100146990,LOC100057269,LOC100057228,LOC100057191,LOC100057110,LOC100146600,LOC100057026,LOC100050714,LOC100056989,LOC100056947,LOC100056906,LOC100056869,LOC100146809,LOC100067978,LOC100067957,LOC100056826,LOC100056793,LOC100056755,LOC100147000,LOC100056708,LOC100056626,LOC100056578,LOC100056531,LOC100056488,LOC100629158,LOC100050633,LOC100056395,LOC100056356,LOC100056314,LOC100056273,LOC100056235,LOC100056187,LOC100056146,LOC100630788,LOC100629923,LOC100056026,LOC100055988,LOC100146992,LOC100055947,LOC100147569,LOC100146290,LOC100055908,LOC100055867,LOC100055780,LOC100630577,LOC100055646,LOC100055601,LOC100055561,LOC100055521,LOC100055478,LOC100055435,LOC100055397,LOC100055353,LOC100055309,LOC100055273,LOC100055224,LOC100055177,LOC100055129,LOC100055083,LOC100052898,LOC100147187,LOC100054954,LOC100054906,LOC100054858,LOC100054773,LOC100054722,LOC100054673,LOC100054627,LOC100054586,LOC100054541,LOC100054498,LOC100054453,LOC100146383,LOC100054355,LOC100054307,LOC100054258,LOC100054215,LOC100054169,LOC100054120,LOC100054065,LOC100054021,LOC100050556,LOC100053872,LOC100050476,LOC100053820,LOC100053768,LOC100629822,LOC100053672,LOC100053625,LOC100053568,LOC100053527,LOC100147669,LOC100053479,LOC100053427,LOC100053382,LOC100053328,LOC100053284,LOC100053236,LOC100053185,LOC100053134,LOC100053085,LOC100053045,LOC100052992,LOC100146502,LOC100052946,LOC100146388,LOC100052842,LOC100147383,LOC100052788,LOC100052732,LOC100050399,LOC100052683
12	12841994	13149957	307964	3	1	LOC100053672,LOC100053625,LOC100053568,LOC100053527,LOC100147669,LOC100053479,LOC100053427,LOC100053382,LOC100053328,LOC100053284,LOC100053236,LOC100053185,LOC100053134,LOC100053085,LOC100053045,LOC100052992,LOC100146502,LOC100052946,LOC100146388,LOC100052842,LOC100147383,LOC100052788,LOC100052732,LOC100050399,LOC100052683
12	12841994	13170959	328966	3	1	LOC100053820,LOC100053768,LOC100629822,LOC100053672,LOC100053625,LOC100053568,LOC100053527,LOC100147669,LOC100053479,LOC100053427,LOC100053382,LOC100053328,LOC100053284,LOC100053236,LOC100053185,LOC100053134,LOC100053085,LOC100053045,LOC100052992,LOC100146502,LOC100052946,LOC100146388,LOC100052842,LOC100147383,LOC100052788,LOC100052732,LOC100050399,LOC100052683
12	12841994	13401991	559998	3	2	LOC100146383,LOC100054355,LOC100054307,LOC100054258,LOC100054215,LOC100054169,LOC100054120,LOC100054065,LOC100054021,LOC100050556,LOC100053872,LOC100050476,LOC100053820,LOC100053768,LOC100629822,LOC100053672,LOC100053625,LOC100053568,LOC100053527,LOC100147669,LOC100053479,LOC100053427,LOC100053382,LOC100053328,LOC100053284,LOC100053236,LOC100053185,LOC100053134,LOC100053085,LOC100053045,LOC100052992,LOC100146502,LOC100052946,LOC100146388,LOC100052842,LOC100147383,LOC100052788,LOC100052732,LOC100050399,LOC100052683
12	12841994	13422256	580263	3	1	LOC100054453,LOC100146383,LOC100054355,LOC100054307,LOC100054258,LOC100054215,LOC100054169,LOC100054120,LOC100054065,LOC100054021,LOC100050556,LOC100053872,LOC100050476,LOC100053820,LOC100053768,LOC100629822,LOC100053672,LOC100053625,LOC100053568,LOC100053527,LOC100147669,LOC100053479,LOC100053427,LOC100053382,LOC100053328,LOC100053284,LOC100053236,LOC100053185,LOC100053134,LOC100053085,LOC100053045,LOC100052992,LOC100146502,LOC100052946,LOC100146388,LOC100052842,LOC100147383,LOC100052788,LOC100052732,LOC100050399,LOC100052683

Chrom	Start	End	Size	Cn	Samples (n)	Genes
12	12841994	14391372	1549379	3	1	LOC100057026,LOC100050714,LOC100056989,LOC100056947,LOC100056906,LOC100056869,LOC100146809,LOC100067978,LOC100067957,LOC100056826,LOC100056793,LOC100056755,LOC100147000,LOC100056708,LOC100056626,LOC100056578,LOC100056531,LOC100056488,LOC100629158,LOC100050633,LOC100056395,LOC100056356,LOC100056314,LOC100056273,LOC100056235,LOC100056187,LOC100056146,LOC100630788,LOC100629923,LOC100056026,LOC100055988,LOC100146992,LOC100055947,LOC100147569,LOC100146290,LOC100055908,LOC100055867,LOC100055780,LOC100630577,LOC100055646,LOC100055601,LOC100055561,LOC100055521,LOC100055478,LOC100055435,LOC100055397,LOC100055353,LOC100055309,LOC100055273,LOC100055224,LOC100055177,LOC100055129,LOC100055083,LOC100052898,LOC100147187,LOC100054954,LOC100054906,LOC100054858,LOC100054773,LOC100054722,LOC100054673,LOC100054627,LOC100054586,LOC100054541,LOC100054498,LOC100054453,LOC100146383,LOC100054355,LOC100054307,LOC100054258,LOC100054215,LOC100054169,LOC100054120,LOC100054065,LOC100054021,LOC100050556,LOC100053872,LOC100050476,LOC100053820,LOC100053768,LOC100629822,LOC100053672,LOC100053625,LOC100053568,LOC100053527,LOC100147669,LOC100053479,LOC100053427,LOC100053382,LOC100053328,LOC100053284,LOC100053236,LOC100053185,LOC100053134,LOC100053085,LOC100053045,LOC100052992,LOC100146502,LOC100052946,LOC100146388,LOC100052842,LOC100147383,LOC100052788,LOC100052732,LOC100050399,LOC100052683
12	13149957	13364132	214176	3	2	LOC100054215,LOC100054169,LOC100054120,LOC100054065,LOC100054021,LOC100050556,LOC100053872,LOC100050476,LOC100053820,LOC100053768,LOC100629822
12	13149957	13379725	229769	3	1	LOC100054258,LOC100054215,LOC100054169,LOC100054120,LOC100054065,LOC100054021,LOC100050556,LOC100053872,LOC100050476,LOC100053820,LOC100053768,LOC100629822
12	13149957	13398392	248436	3	3	LOC100054355,LOC100054307,LOC100054258,LOC100054215,LOC100054169,LOC100054120,LOC100054065,LOC100054021,LOC100050556,LOC100053872,LOC100050476,LOC100053820,LOC100053768,LOC100629822
12	13149957	13401991	252035	3	8	LOC100146383,LOC100054355,LOC100054307,LOC100054258,LOC100054215,LOC100054169,LOC100054120,LOC100054065,LOC100054021,LOC100050556,LOC100053872,LOC100050476,LOC100053820,LOC100053768,LOC100629822
12	13149957	13422256	272300	3	1	LOC100054453,LOC100146383,LOC100054355,LOC100054307,LOC100054258,LOC100054215,LOC100054169,LOC100054120,LOC100054065,LOC100054021,LOC100050556,LOC100053872,LOC100050476,LOC100053820,LOC100053768,LOC100629822
12	13149957	13439146	289190	0	1	LOC100054498,LOC100054453,LOC100146383,LOC100054355,LOC100054307,LOC100054258,LOC100054215,LOC100054169,LOC100054120,LOC100054065,LOC100054021,LOC100050556,LOC100053872,LOC100050476,LOC100053820,LOC100053768,LOC100629822
12	13149957	13488187	338231	0,1	4,1	LOC100054673,LOC100054627,LOC100054586,LOC100054541,LOC100054498,LOC100054453,LOC100146383,LOC100054355,LOC100054307,LOC100054258,LOC100054215,LOC100054169,LOC100054120,LOC100054065,LOC100054021,LOC100050556,LOC100053872,LOC100050476,LOC100053820,LOC100053768,LOC100629822
12	13149957	14128309	978353	3	4	LOC100056626,LOC100056578,LOC100056531,LOC100056488,LOC100629158,LOC100050633,LOC100056395,LOC100056356,LOC100056314,LOC100056273,LOC100056235,LOC100056187,LOC100056146,LOC100630788,LOC100629923,LOC100056026,LOC100055988,LOC100146992,LOC100055947,LOC100147569,LOC100146290,LOC100055908,LOC100055867,LOC100055780,LOC100630577,LOC100055646,LOC100055601,LOC100055561,LOC100055521,LOC100055478,LOC100055435,LOC100055397,LOC100055353,LOC100055309,LOC100055273,LOC100055224,LOC100055177,LOC100055129,LOC100055083,LOC100052898,LOC100147187,LOC100054954,LOC100054906,LOC100054858,LOC100054773,LOC100054722,LOC100054673,LOC100054627,LOC100054586,LOC100054541,LOC100054498,LOC100054453,LOC100146383,LOC100054355,LOC100054307,LOC100054258,LOC100054215,LOC100054169,LOC100054120,LOC100054065,LOC100054021,LOC100050556,LOC100053872,LOC100050476,LOC100053820,LOC100053768,LOC100629822
12	13149957	14354314	1204358	3	4	LOC100056947,LOC100056906,LOC100056869,LOC100146809,LOC100067978,LOC100067957,LOC100056826,LOC100056793,LOC100056755,LOC100147000,LOC100056708,LOC100056626,LOC100056578,LOC100056531,LOC100056488,LOC100629158,LOC100050633,LOC100056395,LOC100056356,LOC100056314,LOC100056273,LOC100056235,LOC100056187,LOC100056146,LOC100630788,LOC100629923,LOC100056026,LOC100055988,LOC100146992,LOC100055947,LOC100147569,LOC100146290,LOC100055908,LOC100055867,LOC100055780,LOC100630577,LOC100055646,LOC100055601,LOC100055561,LOC100055521,LOC100055478,LOC100055435,LOC100055397,LOC100055353,LOC100055309,LOC100055273,LOC100055224,LOC100055177,LOC100055129,LOC100055083,LOC100052898,LOC100147187,LOC100054954,LOC100054906,LOC100054858,LOC100054773,LOC100054722,LOC100054673,LOC100054627,LOC100054586,LOC100054541,LOC100054498,LOC100054453,LOC100146383,LOC100054355,LOC100054307,LOC100054258,LOC100054215,LOC100054169,LOC100054120,LOC100054065,LOC100054021,LOC100050556,LOC100053872,LOC100050476,LOC100053820,LOC100053768,LOC100629822
12	13149957	14391372	1241416	1,3,4	1,8,1	LOC100057026,LOC100050714,LOC100056989,LOC100056947,LOC100056906,LOC100056869,LOC100146809,LOC100067978,LOC100067957,LOC100056826,LOC100056793,LOC100056755,LOC100147000,LOC100056708,LOC100056626,LOC100056578,LOC100056531,LOC100056488,LOC100629158,LOC100050633,LOC100056395,LOC100056356,LOC100056314,LOC100056273,LOC100056235,LOC100056187,LOC100056146,LOC100630788,LOC100629923,LOC100056026,LOC100055988,LOC100146992,LOC100055947,LOC100147569,LOC100146290,LOC100055908,LOC100055867,LOC100055780,LOC100630577,LOC100055646,LOC100055601,LOC100055561,LOC100055521,LOC100055478,LOC100055435,LOC100055397,LOC100055353,LOC100055309,LOC100055273,LOC100055224,LOC100055177,LOC100055129,LOC100055083,LOC100052898,LOC100147187,LOC100054954,LOC100054906,LOC100054858,LOC100054773,LOC100054722,LOC100054673,LOC100054627,LOC100054586,LOC100054541,LOC100054498,LOC100054453,LOC100146383,LOC100054355,LOC100054307,LOC100054258,LOC100054215,LOC100054169,LOC100054120,LOC100054065,LOC100054021,LOC100050556,LOC100053872,LOC100050476,LOC100053820,LOC100053768,LOC100629822

Chrom	Start	End	Size	Cn	Samples (n)	Genes
12	13149957	14777981	1628025	3	2	LOC100058077,LOC100147282,LOC100058036,LOC100057989,LOC100057945,LOC100057894,LOC100057845,LOC100057804,LOC100057756,LOC100057709,LOC100057670,LOC100057589,LOC100057547,LOC100057513,LOC100057472,LOC100057433,LOC100057393,LOC100057352,LOC100057310,LOC100147286,LOC100146990,LOC100057269,LOC100057228,LOC100057191,LOC100057110,LOC100146600,LOC100057026,LOC100050714,LOC100056989,LOC100056947,LOC100056906,LOC100056869,LOC100146809,LOC100067978,LOC100067957,LOC100056826,LOC100056793,LOC100056755,LOC100147000,LOC100056708,LOC100056626,LOC100056578,LOC100056531,LOC100056488,LOC100629158,LOC100050633,LOC100056395,LOC100056356,LOC100056314,LOC100056273,LOC100056235,LOC100056187,LOC100056146,LOC100630788,LOC100629923,LOC100056026,LOC100055988,LOC100146992,LOC100055947,LOC100147569,LOC100146290,LOC100055908,LOC100055867,LOC100055780,LOC100630577,LOC100055646,LOC100055601,LOC100055561,LOC100055521,LOC100055478,LOC100055435,LOC100055397,LOC100055353,LOC100055309,LOC100055273,LOC100055224,LOC100055177,LOC100055129,LOC100055083,LOC100052898,LOC100147187,LOC100054954,LOC100054906,LOC100054858,LOC100054773,LOC100054722,LOC100054673,LOC100054627,LOC100054586,LOC100054541,LOC100054498,LOC100054453,LOC100146383,LOC100054355,LOC100054307,LOC100054258,LOC100054215,LOC100054169,LOC100054120,LOC100054065,LOC100054021,LOC100050556,LOC100053872,LOC100050476,LOC100053820,LOC100053768,LOC100629822
12	13170959	13401991	231033	3	2	LOC100146383,LOC100054355,LOC100054307,LOC100054258,LOC100054215,LOC100054169,LOC100054120,LOC100054065,LOC100054021,LOC100050556,LOC100053872,LOC100050476,LOC100053820
12	13170959	14354314	1183356	3	1	LOC100056947,LOC100056906,LOC100056869,LOC100146809,LOC100067978,LOC100067957,LOC100056826,LOC100056793,LOC100056755,LOC100147000,LOC100056708,LOC100056626,LOC100056578,LOC100056531,LOC100056488,LOC100629158,LOC100050633,LOC100056395,LOC100056356,LOC100056314,LOC100056273,LOC100056235,LOC100056187,LOC100056146,LOC100630788,LOC100629923,LOC100056026,LOC100055988,LOC100146992,LOC100055947,LOC100147569,LOC100146290,LOC100055908,LOC100055867,LOC100055780,LOC100630577,LOC100055646,LOC100055601,LOC100055561,LOC100055521,LOC100055478,LOC100055435,LOC100055397,LOC100055353,LOC100055309,LOC100055273,LOC100055224,LOC100055177,LOC100055129,LOC100055083,LOC100052898,LOC100147187,LOC100054954,LOC100054906,LOC100054858,LOC100054773,LOC100054722,LOC100054673,LOC100054627,LOC100054586,LOC100054541,LOC100054498,LOC100054453,LOC100146383,LOC100054355,LOC100054307,LOC100054258,LOC100054215,LOC100054169,LOC100054120,LOC100054065,LOC100054021,LOC100050556,LOC100053872,LOC100050476,LOC100053820
12	13170959	14391372	1220414	3	3	LOC100057026,LOC100050714,LOC100056989,LOC100056947,LOC100056906,LOC100056869,LOC100146809,LOC100067978,LOC100067957,LOC100056826,LOC100056793,LOC100056755,LOC100147000,LOC100056708,LOC100056626,LOC100056578,LOC100056531,LOC100056488,LOC100629158,LOC100050633,LOC100056395,LOC100056356,LOC100056314,LOC100056273,LOC100056235,LOC100056187,LOC100056146,LOC100630788,LOC100629923,LOC100056026,LOC100055988,LOC100146992,LOC100055947,LOC100147569,LOC100146290,LOC100055908,LOC100055867,LOC100055780,LOC100630577,LOC100055646,LOC100055601,LOC100055561,LOC100055521,LOC100055478,LOC100055435,LOC100055397,LOC100055353,LOC100055309,LOC100055273,LOC100055224,LOC100055177,LOC100055129,LOC100055083,LOC100052898,LOC100147187,LOC100054954,LOC100054906,LOC100054858,LOC100054773,LOC100054722,LOC100054673,LOC100054627,LOC100054586,LOC100054541,LOC100054498,LOC100054453,LOC100146383,LOC100054355,LOC100054307,LOC100054258,LOC100054215,LOC100054169,LOC100054120,LOC100054065,LOC100054021,LOC100050556,LOC100053872,LOC100050476,LOC100053820
12	13364132	13398392	34261	3	2	LOC100054355,LOC100054307,LOC100054258
12	13364132	13401991	37860	0,3	1,1	LOC100146383,LOC100054355,LOC100054307,LOC100054258
12	13364132	13422256	58125	0	2	LOC100054453,LOC100146383,LOC100054355,LOC100054307,LOC100054258
12	13364132	13439146	75015	3	1	LOC100054498,LOC100054453,LOC100146383,LOC100054355,LOC100054307,LOC100054258
12	13364132	14128309	764178	3	11	LOC100056626,LOC100056578,LOC100056531,LOC100056488,LOC100629158,LOC100050633,LOC100056395,LOC100056356,LOC100056314,LOC100056273,LOC100056235,LOC100056187,LOC100056146,LOC100630788,LOC100629923,LOC100056026,LOC100055988,LOC100146992,LOC100055947,LOC100147569,LOC100146290,LOC100055908,LOC100055867,LOC100055780,LOC100630577,LOC100055646,LOC100055601,LOC100055561,LOC100055521,LOC100055478,LOC100055435,LOC100055397,LOC100055353,LOC100055309,LOC100055273,LOC100055224,LOC100055177,LOC100055129,LOC100055083,LOC100052898,LOC100147187,LOC100054954,LOC100054906,LOC100054858,LOC100054773,LOC100054722,LOC100054673,LOC100054627,LOC100054586,LOC100054541,LOC100054498,LOC100054453,LOC100146383,LOC100054355,LOC100054307,LOC100054258
12	13364132	14297056	932925	3	1	LOC100056869,LOC100146809,LOC100067978,LOC100067957,LOC100056826,LOC100056793,LOC100056755,LOC100147000,LOC100056708,LOC100056626,LOC100056578,LOC100056531,LOC100056488,LOC100629158,LOC100050633,LOC100056395,LOC100056356,LOC100056314,LOC100056273,LOC100056235,LOC100056187,LOC100056146,LOC100630788,LOC100629923,LOC100056026,LOC100055988,LOC100146992,LOC100055947,LOC100147569,LOC100146290,LOC100055908,LOC100055867,LOC100055780,LOC100630577,LOC100055646,LOC100055601,LOC100055561,LOC100055521,LOC100055478,LOC100055435,LOC100055397,LOC100055353,LOC100055309,LOC100055273,LOC100055224,LOC100055177,LOC100055129,LOC100055083,LOC100052898,LOC100147187,LOC100054954,LOC100054906,LOC100054858,LOC100054773,LOC100054722,LOC100054673,LOC100054627,LOC100054586,LOC100054541,LOC100054498,LOC100054453,LOC100146383,LOC100054355,LOC100054307,LOC100054258
12	13364132	14354314	990183	3	1	LOC100056947,LOC100056906,LOC100056869,LOC100146809,LOC100067978,LOC100067957,LOC100056826,LOC100056793,LOC100056755,LOC100147000,LOC100056708,LOC100056626,LOC100056578,LOC100056531,LOC100056488,LOC100629158,LOC100050633,LOC100056395,LOC100056356,LOC100056314,LOC100056273,LOC100056235,LOC100056187,LOC100056146,LOC100630788,LOC100629923,LOC100056026,LOC100055988,LOC100146992,LOC100055947,LOC100147569,LOC100146290,LOC100055908,LOC100055867,LOC100055780,LOC100630577,LOC100055646,LOC100055601,LOC100055561,LOC100055521,LOC100055478,LOC100055435,LOC100055397,LOC100055353,LOC100055309,LOC100055273,LOC100055224,LOC100055177,LOC100055129,LOC100055083,LOC100052898,LOC100147187,LOC100054954,LOC100054906,LOC100054858,LOC100054773,LOC100054722,LOC100054673,LOC100054627,LOC100054586,LOC100054541,LOC100054498,LOC100054453,LOC100146383,LOC100054355,LOC100054307,LOC100054258

Chrom	Start	End	Size	Cn	Samples (n)	Genes
12	13364132	14391372	1027241	3	5	LOC100057026,LOC100050714,LOC100056989,LOC100056947,LOC100056906,LOC100056869,LOC100146809,LOC100067978,LOC100067957,LOC100056826,LOC100056793,LOC100056755,LOC100147000,LOC100056708,LOC100056626,LOC100056578,LOC100056531,LOC100056488,LOC100629158,LOC100050633,LOC100056395,LOC100056356,LOC100056314,LOC100056273,LOC100056235,LOC100056187,LOC100056146,LOC100630788,LOC100629923,LOC100056026,LOC100055988,LOC100146992,LOC100055947,LOC100147569,LOC100146290,LOC100055908,LOC100055867,LOC100055780,LOC100630577,LOC100055646,LOC100055601,LOC100055561,LOC100055521,LOC100055478,LOC100055435,LOC100055397,LOC100055353,LOC100055309,LOC100055273,LOC100055224,LOC100055177,LOC100055129,LOC100055083,LOC100052898,LOC100147187,LOC100054954,LOC100054906,LOC100054858,LOC100054773,LOC100054722,LOC100054673,LOC100054627,LOC100054586,LOC100054541,LOC100054498,LOC100054453,LOC100146383,LOC100054355,LOC100054307,LOC100054258
12	13364132	14777981	1413850	3	1	LOC100058077,LOC100147282,LOC100058036,LOC100057989,LOC100057945,LOC100057894,LOC100057845,LOC100057804,LOC100057756,LOC100057709,LOC100057670,LOC100057589,LOC100057547,LOC100057513,LOC100057472,LOC100057433,LOC100057393,LOC100057352,LOC100057310,LOC100147286,LOC100146990,LOC100057269,LOC100057228,LOC100057191,LOC100057110,LOC100146600,LOC100057026,LOC100050714,LOC100056989,LOC100056947,LOC100056906,LOC100056869,LOC100146809,LOC100067978,LOC100067957,LOC100056826,LOC100056793,LOC100056755,LOC100147000,LOC100056708,LOC100056626,LOC100056578,LOC100056531,LOC100056488,LOC100629158,LOC100050633,LOC100056395,LOC100056356,LOC100056314,LOC100056273,LOC100056235,LOC100056187,LOC100056146,LOC100630788,LOC100629923,LOC100056026,LOC100055988,LOC100146992,LOC100055947,LOC100147569,LOC100146290,LOC100055908,LOC100055867,LOC100055780,LOC100630577,LOC100055646,LOC100055601,LOC100055561,LOC100055521,LOC100055478,LOC100055435,LOC100055397,LOC100055353,LOC100055309,LOC100055273,LOC100055224,LOC100055177,LOC100055129,LOC100055083,LOC100052898,LOC100147187,LOC100054954,LOC100054906,LOC100054858,LOC100054773,LOC100054722,LOC100054673,LOC100054627,LOC100054586,LOC100054541,LOC100054498,LOC100054453,LOC100146383,LOC100054355,LOC100054307,LOC100054258
12	13379725	13439146	59422	1	1	LOC100054498,LOC100054453,LOC100146383,LOC100054355,LOC100054307
12	13379725	14128309	748585	3	1	LOC100056626,LOC100056578,LOC100056531,LOC100056488,LOC100629158,LOC100050633,LOC100056395,LOC100056356,LOC100056314,LOC100056273,LOC100056235,LOC100056187,LOC100056146,LOC100630788,LOC100629923,LOC100056026,LOC100055988,LOC100146992,LOC100055947,LOC100147569,LOC100146290,LOC100055908,LOC100055867,LOC100055780,LOC100630577,LOC100055646,LOC100055601,LOC100055561,LOC100055521,LOC100055478,LOC100055435,LOC100055397,LOC100055353,LOC100055309,LOC100055273,LOC100055224,LOC100055177,LOC100055129,LOC100055083,LOC100052898,LOC100147187,LOC100054954,LOC100054906,LOC100054858,LOC100054773,LOC100054722,LOC100054673,LOC100054627,LOC100054586,LOC100054541,LOC100054498,LOC100054453,LOC100146383,LOC100054355,LOC100054307
12	13379725	14354314	974590	3	1	LOC100056947,LOC100056906,LOC100056869,LOC100146809,LOC100067978,LOC100067957,LOC100056826,LOC100056793,LOC100056755,LOC100147000,LOC100056708,LOC100056626,LOC100056578,LOC100056531,LOC100056488,LOC100629158,LOC100050633,LOC100056395,LOC100056356,LOC100056314,LOC100056273,LOC100056235,LOC100056187,LOC100056146,LOC100630788,LOC100629923,LOC100056026,LOC100055988,LOC100146992,LOC100055947,LOC100147569,LOC100146290,LOC100055908,LOC100055867,LOC100055780,LOC100630577,LOC100055646,LOC100055601,LOC100055561,LOC100055521,LOC100055478,LOC100055435,LOC100055397,LOC100055353,LOC100055309,LOC100055273,LOC100055224,LOC100055177,LOC100055129,LOC100055083,LOC100052898,LOC100147187,LOC100054954,LOC100054906,LOC100054858,LOC100054773,LOC100054722,LOC100054673,LOC100054627,LOC100054586,LOC100054541,LOC100054498,LOC100054453,LOC100146383,LOC100054355,LOC100054307
12	13379725	14391372	1011648	3	1	LOC100057026,LOC100050714,LOC100056989,LOC100056947,LOC100056906,LOC100056869,LOC100146809,LOC100067978,LOC100067957,LOC100056826,LOC100056793,LOC100056755,LOC100147000,LOC100056708,LOC100056626,LOC100056578,LOC100056531,LOC100056488,LOC100629158,LOC100050633,LOC100056395,LOC100056356,LOC100056314,LOC100056273,LOC100056235,LOC100056187,LOC100056146,LOC100630788,LOC100629923,LOC100056026,LOC100055988,LOC100146992,LOC100055947,LOC100147569,LOC100146290,LOC100055908,LOC100055867,LOC100055780,LOC100630577,LOC100055646,LOC100055601,LOC100055561,LOC100055521,LOC100055478,LOC100055435,LOC100055397,LOC100055353,LOC100055309,LOC100055273,LOC100055224,LOC100055177,LOC100055129,LOC100055083,LOC100052898,LOC100147187,LOC100054954,LOC100054906,LOC100054858,LOC100054773,LOC100054722,LOC100054673,LOC100054627,LOC100054586,LOC100054541,LOC100054498,LOC100054453,LOC100146383,LOC100054355,LOC100054307
12	13379725	14777981	1398257	3	1	LOC100058077,LOC100147282,LOC100058036,LOC100057989,LOC100057945,LOC100057894,LOC100057845,LOC100057804,LOC100057756,LOC100057709,LOC100057670,LOC100057589,LOC100057547,LOC100057513,LOC100057472,LOC100057433,LOC100057393,LOC100057352,LOC100057310,LOC100147286,LOC100146990,LOC100057269,LOC100057228,LOC100057191,LOC100057110,LOC100146600,LOC100057026,LOC100050714,LOC100056989,LOC100056947,LOC100056906,LOC100056869,LOC100146809,LOC100067978,LOC100067957,LOC100056826,LOC100056793,LOC100056755,LOC100147000,LOC100056708,LOC100056626,LOC100056578,LOC100056531,LOC100056488,LOC100629158,LOC100050633,LOC100056395,LOC100056356,LOC100056314,LOC100056273,LOC100056235,LOC100056187,LOC100056146,LOC100630788,LOC100629923,LOC100056026,LOC100055988,LOC100146992,LOC100055947,LOC100147569,LOC100146290,LOC100055908,LOC100055867,LOC100055780,LOC100630577,LOC100055646,LOC100055601,LOC100055561,LOC100055521,LOC100055478,LOC100055435,LOC100055397,LOC100055353,LOC100055309,LOC100055273,LOC100055224,LOC100055177,LOC100055129,LOC100055083,LOC100052898,LOC100147187,LOC100054954,LOC100054906,LOC100054858,LOC100054773,LOC100054722,LOC100054673,LOC100054627,LOC100054586,LOC100054541,LOC100054498,LOC100054453,LOC100146383,LOC100054355,LOC100054307
12	13401991	14391372	989382	1	1	LOC100057026,LOC100050714,LOC100056989,LOC100056947,LOC100056906,LOC100056869,LOC100146809,LOC100067978,LOC100067957,LOC100056826,LOC100056793,LOC100056755,LOC100147000,LOC100056708,LOC100056626,LOC100056578,LOC100056531,LOC100056488,LOC100629158,LOC100050633,LOC100056395,LOC100056356,LOC100056314,LOC100056273,LOC100056235,LOC100056187,LOC100056146,LOC100630788,LOC100629923,LOC100056026,LOC100055988,LOC100146992,LOC100055947,LOC100147569,LOC100146290,LOC100055908,LOC100055867,LOC100055780,LOC100630577,LOC100055646,LOC100055601,LOC100055561,LOC100055521,LOC100055478,LOC100055435,LOC100055397,LOC100055353,LOC100055309,LOC100055273,LOC100055224,LOC100055177,LOC100055129,LOC100055083,LOC100052898,LOC100147187,LOC100054954,LOC100054906,LOC100054858,LOC100054773,LOC100054722,LOC100054673,LOC100054627,LOC100054586,LOC100054541,LOC100054498,LOC100054453,LOC100146383

Chrom	Start	End	Size	Cn	Samples (n)	Genes
12	13422256	14391372	969117	1,3	1,1	LOC100057026,LOC100050714,LOC100056989,LOC100056947,LOC100056906,LOC100056869,LOC100146809,LOC100067978,LOC100067957,LOC100056826,LOC100056793,LOC100056755,LOC100147000,LOC100056708,LOC100056626,LOC100056578,LOC100056531,LOC100056488,LOC100629158,LOC100050633,LOC100056395,LOC100056356,LOC100056314,LOC100056273,LOC100056235,LOC100056187,LOC100056146,LOC100630788,LOC100629923,LOC100056026,LOC100055988,LOC100146992,LOC100055947,LOC100147569,LOC100146290,LOC100055908,LOC100055867,LOC100055780,LOC100630577,LOC100055646,LOC100055601,LOC100055561,LOC100055521,LOC100055478,LOC100055435,LOC100055397,LOC100055353,LOC100055309,LOC100055273,LOC100055224,LOC100055177,LOC100055129,LOC100055083,LOC100052898,LOC100147187,LOC100054954,LOC100054906,LOC100054858,LOC100054773,LOC100054722,LOC100054673,LOC100054627,LOC100054586,LOC100054541,LOC100054498
12	13422256	14777981	1355726	1	1	LOC100058077,LOC100147282,LOC100058036,LOC100057989,LOC100057945,LOC100057894,LOC100057845,LOC100057804,LOC100057756,LOC100057709,LOC100057670,LOC100057589,LOC100057547,LOC100057513,LOC100057472,LOC100057433,LOC100057393,LOC100057352,LOC100057310,LOC100147286,LOC100146990,LOC100057269,LOC100057228,LOC100057191,LOC100057110,LOC100146600,LOC100057026,LOC100050714,LOC100056989,LOC100056947,LOC100056906,LOC100056869,LOC100146809,LOC100067978,LOC100067957,LOC100056826,LOC100056793,LOC100056755,LOC100147000,LOC100056708,LOC100056626,LOC100056578,LOC100056531,LOC100056488,LOC100629158,LOC100050633,LOC100056395,LOC100056356,LOC100056314,LOC100056273,LOC100056235,LOC100056187,LOC100056146,LOC100630788,LOC100629923,LOC100056026,LOC100055988,LOC100146992,LOC100055947,LOC100147569,LOC100146290,LOC100055908,LOC100055867,LOC100055780,LOC100630577,LOC100055646,LOC100055601,LOC100055561,LOC100055521,LOC100055478,LOC100055435,LOC100055397,LOC100055353,LOC100055309,LOC100055273,LOC100055224,LOC100055177,LOC100055129,LOC100055083,LOC100052898,LOC100147187,LOC100054954,LOC100054906,LOC100054858,LOC100054773,LOC100054722,LOC100054673,LOC100054627,LOC100054586,LOC100054541,LOC100054498
12	13439146	13573356	134211	1	1	LOC100147187,LOC100054954,LOC100054906,LOC100054858,LOC100054773,LOC100054722,LOC100054673,LOC100054627,LOC100054586,LOC100054541
12	13439146	14391372	952227	1,3	2,1	LOC100057026,LOC100050714,LOC100056989,LOC100056947,LOC100056906,LOC100056869,LOC100146809,LOC100067978,LOC100067957,LOC100056826,LOC100056793,LOC100056755,LOC100147000,LOC100056708,LOC100056626,LOC100056578,LOC100056531,LOC100056488,LOC100629158,LOC100050633,LOC100056395,LOC100056356,LOC100056314,LOC100056273,LOC100056235,LOC100056187,LOC100056146,LOC100630788,LOC100629923,LOC100056026,LOC100055988,LOC100146992,LOC100055947,LOC100147569,LOC100146290,LOC100055908,LOC100055867,LOC100055780,LOC100630577,LOC100055646,LOC100055601,LOC100055561,LOC100055521,LOC100055478,LOC100055435,LOC100055397,LOC100055353,LOC100055309,LOC100055273,LOC100055224,LOC100055177,LOC100055129,LOC100055083,LOC100052898,LOC100147187,LOC100054954,LOC100054906,LOC100054858,LOC100054773,LOC100054722,LOC100054673,LOC100054627,LOC100054586,LOC100054541
12	13488187	14128309	640123	4	1	LOC100056626,LOC100056578,LOC100056531,LOC100056488,LOC100629158,LOC100050633,LOC100056395,LOC100056356,LOC100056314,LOC100056273,LOC100056235,LOC100056187,LOC100056146,LOC100630788,LOC100629923,LOC100056026,LOC100055988,LOC100146992,LOC100055947,LOC100147569,LOC100146290,LOC100055908,LOC100055867,LOC100055780,LOC100630577,LOC100055646,LOC100055601,LOC100055561,LOC100055521,LOC100055478,LOC100055435,LOC100055397,LOC100055353,LOC100055309,LOC100055273,LOC100055224,LOC100055177,LOC100055129,LOC100055083,LOC100052898,LOC100147187,LOC100054954,LOC100054906,LOC100054858,LOC100054773,LOC100054722
12	13488187	14391372	903186	3	2	LOC100057026,LOC100050714,LOC100056989,LOC100056947,LOC100056906,LOC100056869,LOC100146809,LOC100067978,LOC100067957,LOC100056826,LOC100056793,LOC100056755,LOC100147000,LOC100056708,LOC100056626,LOC100056578,LOC100056531,LOC100056488,LOC100629158,LOC100050633,LOC100056395,LOC100056356,LOC100056314,LOC100056273,LOC100056235,LOC100056187,LOC100056146,LOC100630788,LOC100629923,LOC100056026,LOC100055988,LOC100146992,LOC100055947,LOC100147569,LOC100146290,LOC100055908,LOC100055867,LOC100055780,LOC100630577,LOC100055646,LOC100055601,LOC100055561,LOC100055521,LOC100055478,LOC100055435,LOC100055397,LOC100055353,LOC100055309,LOC100055273,LOC100055224,LOC100055177,LOC100055129,LOC100055083,LOC100052898,LOC100147187,LOC100054954,LOC100054906,LOC100054858,LOC100054773,LOC100054722
12	13488187	14777981	1289795	3	2	LOC100058077,LOC100147282,LOC100058036,LOC100057989,LOC100057945,LOC100057894,LOC100057845,LOC100057804,LOC100057756,LOC100057709,LOC100057670,LOC100057589,LOC100057547,LOC100057513,LOC100057472,LOC100057433,LOC100057393,LOC100057352,LOC100057310,LOC100147286,LOC100146990,LOC100057269,LOC100057228,LOC100057191,LOC100057110,LOC100146600,LOC100057026,LOC100050714,LOC100056989,LOC100056947,LOC100056906,LOC100056869,LOC100146809,LOC100067978,LOC100067957,LOC100056826,LOC100056793,LOC100056755,LOC100147000,LOC100056708,LOC100056626,LOC100056578,LOC100056531,LOC100056488,LOC100629158,LOC100050633,LOC100056395,LOC100056356,LOC100056314,LOC100056273,LOC100056235,LOC100056187,LOC100056146,LOC100630788,LOC100629923,LOC100056026,LOC100055988,LOC100146992,LOC100055947,LOC100147569,LOC100146290,LOC100055908,LOC100055867,LOC100055780,LOC100630577,LOC100055646,LOC100055601,LOC100055561,LOC100055521,LOC100055478,LOC100055435,LOC100055397,LOC100055353,LOC100055309,LOC100055273,LOC100055224,LOC100055177,LOC100055129,LOC100055083,LOC100052898,LOC100147187,LOC100054954,LOC100054906,LOC100054858,LOC100054773,LOC100054722
12	13573356	14354314	780959	3	1	LOC100056947,LOC100056906,LOC100056869,LOC100146809,LOC100067978,LOC100067957,LOC100056826,LOC100056793,LOC100056755,LOC100147000,LOC100056708,LOC100056626,LOC100056578,LOC100056531,LOC100056488,LOC100629158,LOC100050633,LOC100056395,LOC100056356,LOC100056314,LOC100056273,LOC100056235,LOC100056187,LOC100056146,LOC100630788,LOC100629923,LOC100056026,LOC100055988,LOC100146992,LOC100055947,LOC100147569,LOC100146290,LOC100055908,LOC100055867,LOC100055780,LOC100630577,LOC100055646,LOC100055601,LOC100055561,LOC100055521,LOC100055478,LOC100055435,LOC100055397,LOC100055353,LOC100055309,LOC100055273,LOC100055224,LOC100055177,LOC100055129,LOC100055083,LOC100052898
12	13573356	14391372	818017	3	1	LOC100057026,LOC100050714,LOC100056989,LOC100056947,LOC100056906,LOC100056869,LOC100146809,LOC100067978,LOC100067957,LOC100056826,LOC100056793,LOC100056755,LOC100147000,LOC100056708,LOC100056626,LOC100056578,LOC100056531,LOC100056488,LOC100629158,LOC100050633,LOC100056395,LOC100056356,LOC100056314,LOC100056273,LOC100056235,LOC100056187,LOC100056146,LOC100630788,LOC100629923,LOC100056026,LOC100055988,LOC100146992,LOC100055947,LOC100147569,LOC100146290,LOC100055908,LOC100055867,LOC100055780,LOC100630577,LOC100055646,LOC100055601,LOC100055561,LOC100055521,LOC100055478,LOC100055435,LOC100055397,LOC100055353,LOC100055309,LOC100055273,LOC100055224,LOC100055177,LOC100055129,LOC100055083,LOC100052898

Chrom	Start	End	Size	Cn	Samples (n)	Genes
12	13678953	14128309	449357	0	1	LOC100056626,LOC100056578,LOC100056531,LOC100056488,LOC100629158,LOC100050633,LOC100056395,LOC100056356,LOC100056314,LOC100056273,LOC100056235,LOC100056187,LOC100056146,LOC100630788,LOC100629923,LOC100056026,LOC100055988,LOC100146992,LOC100055947,LOC100147569,LOC100146290,LOC100055908,LOC100055867,LOC100055780,LOC100630577,LOC100055646,LOC100055601,LOC100055561,LOC100055521,LOC100055478,LOC100055435,LOC100055397
12	13945011	14108352	163342	1	1	LOC100056531,LOC100056488,LOC100629158,LOC100050633,LOC100056395,LOC100056356,LOC100056314,LOC100056273,LOC100056235,LOC100056187,LOC100056146
12	13945011	14128309	183299	1,3	1,8	LOC100056626,LOC100056578,LOC100056531,LOC100056488,LOC100629158,LOC100050633,LOC100056395,LOC100056356,LOC100056314,LOC100056273,LOC100056235,LOC100056187,LOC100056146
12	13945011	14297056	352046	3	2	LOC100056869,LOC100146809,LOC100067978,LOC100067957,LOC100056826,LOC100056793,LOC100056755,LOC100147000,LOC100056708,LOC100056626,LOC100056578,LOC100056531,LOC100056488,LOC100629158,LOC100050633,LOC100056395,LOC100056356,LOC100056314,LOC100056273,LOC100056235,LOC100056187,LOC100056146
12	13945011	14354314	409304	3	7	LOC100056947,LOC100056906,LOC100056869,LOC100146809,LOC100067978,LOC100067957,LOC100056826,LOC100056793,LOC100056755,LOC100147000,LOC100056708,LOC100056626,LOC100056578,LOC100056531,LOC100056488,LOC100629158,LOC100050633,LOC100056395,LOC100056356,LOC100056314,LOC100056273,LOC100056235,LOC100056187,LOC100056146
12	13945011	14391372	446362	0,3,4	4,10,2	LOC100057026,LOC100050714,LOC100056989,LOC100056947,LOC100056906,LOC100056869,LOC100146809,LOC100067978,LOC100067957,LOC100056826,LOC100056793,LOC100056755,LOC100147000,LOC100056708,LOC100056626,LOC100056578,LOC100056531,LOC100056488,LOC100629158,LOC100050633,LOC100056395,LOC100056356,LOC100056314,LOC100056273,LOC100056235,LOC100056187,LOC100056146
12	13945011	14777981	832971	3,4	5,2	LOC100058077,LOC100147282,LOC100058036,LOC100057989,LOC100057945,LOC100057894,LOC100057845,LOC100057804,LOC100057756,LOC100057709,LOC100057670,LOC100057589,LOC100057547,LOC100057513,LOC100057472,LOC100057433,LOC100057393,LOC100057352,LOC100057310,LOC100147286,LOC100146990,LOC100057269,LOC100057228,LOC100057191,LOC100057110,LOC100146600,LOC100057026,LOC100050714,LOC100056989,LOC100056947,LOC100056906,LOC100056869,LOC100146809,LOC100067978,LOC100067957,LOC100056826,LOC100056793,LOC100056755,LOC100147000,LOC100056708,LOC100056626,LOC100056578,LOC100056531,LOC100056488,LOC100629158,LOC100050633,LOC100056395,LOC100056356,LOC100056314,LOC100056273,LOC100056235,LOC100056187,LOC100056146
12	14108229	14124730	16502	3	2	LOC100056626,LOC100056578
12	14108229	14128309	20081	0,1,3	1,9,5	LOC100056626,LOC100056578
12	14108229	14354314	246086	3	4	LOC100056947,LOC100056906,LOC100056869,LOC100146809,LOC100067978,LOC100067957,LOC100056826,LOC100056793,LOC100056755,LOC100147000,LOC100056708,LOC100056626,LOC100056578
12	14108229	14391372	283144	0,1,3	2,1,3	LOC100057026,LOC100050714,LOC100056989,LOC100056947,LOC100056906,LOC100056869,LOC100146809,LOC100067978,LOC100067957,LOC100056826,LOC100056793,LOC100056755,LOC100147000,LOC100056708,LOC100056626,LOC100056578
12	14108229	14777981	669753	3	2	LOC100058077,LOC100147282,LOC100058036,LOC100057989,LOC100057945,LOC100057894,LOC100057845,LOC100057804,LOC100057756,LOC100057709,LOC100057670,LOC100057589,LOC100057547,LOC100057513,LOC100057472,LOC100057433,LOC100057393,LOC100057352,LOC100057310,LOC100147286,LOC100146990,LOC100057269,LOC100057228,LOC100057191,LOC100057110,LOC100146600,LOC100057026,LOC100050714,LOC100056989,LOC100056947,LOC100056906,LOC100056869,LOC100146809,LOC100067978,LOC100067957,LOC100056826,LOC100056793,LOC100056755,LOC100147000,LOC100056708,LOC100056626,LOC100056578
12	14124730	14297056	172327	1	1	LOC100056869,LOC100146809,LOC100067978,LOC100067957,LOC100056826,LOC100056793,LOC100056755,LOC100147000,LOC100056708
12	14124730	14391372	266643	3	1	LOC100057026,LOC100050714,LOC100056989,LOC100056947,LOC100056906,LOC100056869,LOC100146809,LOC100067978,LOC100067957,LOC100056826,LOC100056793,LOC100056755,LOC100147000,LOC100056708
12	14297056	14391003	93948	3	1	LOC100057026,LOC100050714,LOC100056989,LOC100056947,LOC100056906
12	14297056	14391372	94317	0,3	2,5	LOC100057026,LOC100050714,LOC100056989,LOC100056947,LOC100056906
12	14354314	14391372	37059	0,3	1,11	LOC100057026,LOC100050714,LOC100056989
12	14354314	14777981	423668	3	6	LOC100058077,LOC100147282,LOC100058036,LOC100057989,LOC100057945,LOC100057894,LOC100057845,LOC100057804,LOC100057756,LOC100057709,LOC100057670,LOC100057589,LOC100057547,LOC100057513,LOC100057472,LOC100057433,LOC100057393,LOC100057352,LOC100057310,LOC100147286,LOC100146990,LOC100057269,LOC100057228,LOC100057191,LOC100057110,LOC100146600,LOC100057026,LOC100050714,LOC100056989
12	14391003	14777981	386979	3	1	LOC100058077,LOC100147282,LOC100058036,LOC100057989,LOC100057945,LOC100057894,LOC100057845,LOC100057804,LOC100057756,LOC100057709,LOC100057670,LOC100057589,LOC100057547,LOC100057513,LOC100057472,LOC100057433,LOC100057393,LOC100057352,LOC100057310,LOC100147286,LOC100146990,LOC100057269,LOC100057228,LOC100057191,LOC100057110,LOC100146600,LOC100057026
12	15070581	15968363	897783	3	1	LOC100065858,LOC100065831,LOC100065803,LOC100065755,LOC100065735,LOC100065693,LOC100065674,LOC100065655,LOC100065634,LOC100146350,LOC100065610,LOC100065591,LOC100065549,LOC100146953,LOC100065500,LOC100065474,LOC100062694,LOC100065451,LOC100065423,LOC100065402,LOC100065380,LOC100065349,LOC100065319,LOC100065288,LOC100146469,LOC100065263,LOC100147060,LOC100065230,LOC100065201,LOC100065168,LOC100065136,LOC100065111,LOC100065079,LOC100065048,LOC100065019,LOC100630301,LOC100064956,LOC100064924,LOC100062657,LOC100064900,LOC100064874,LOC100064848,LOC100064820,LOC100064792,LOC100064769,LOC100064716,LOC100064690,LOC100062590,LOC100062626,LOC100064662,LOC100630007,LOC100064597,LOC100064562

Chrom	Start	End	Size	Cn	Samples (n)	Genes
12	15070581	16390335	1319755	3	1	LOC100066417,LOC100066361,LOC100066225,LOC100066190,LOC100066164,LOC100630861,LOC100066108,LOC100066068,LOC100147059,LOC100066040,LOC100062734,LOC100066011,LOC100065987,LOC100146657,LOC100065959,LOC100065934,LOC100147543,LOC100065911,LOC100065887,LOC100065858,LOC100065831,LOC100065803,LOC100065755,LOC100065735,LOC100065693,LOC100065674,LOC100065655,LOC100065634,LOC100146350,LOC100065610,LOC100065591,LOC100065549,LOC100146953,LOC100065500,LOC100065474,LOC100062694,LOC100065451,LOC100065423,LOC100065402,LOC100065380,LOC100065349,LOC100065319,LOC100065288,LOC100146469,LOC100065263,LOC100147060,LOC100065230,LOC100065201,LOC100065168,LOC100065136,LOC100065111,LOC100065079,LOC100065048,LOC100065019,LOC100630301,LOC100064956,LOC100064924,LOC100062657,LOC100064900,LOC100064874,LOC100064848,LOC100064820,LOC100064792,LOC100064769,LOC100064716,LOC100064690,LOC100062590,LOC100062626,LOC100064662,LOC100630007,LOC100064597,LOC100064562
12	15362879	15778263	415385	3	1	LOC100065474,LOC100062694,LOC100065451,LOC100065423,LOC100065402,LOC100065380,LOC100065349,LOC100065319,LOC100065288,LOC100146469,LOC100065263,LOC100147060,LOC100065230,LOC100065201,LOC100065168,LOC100065136,LOC100065111,LOC100065079,LOC100065048,LOC100065019,LOC100630301,LOC100064956,LOC100064924,LOC100062657,LOC100064900,LOC100064874,LOC100064848
12	15954830	16608208	653379	3	1	LOC100066553,LOC100066503,LOC100066447,LOC100066417,LOC100066361,LOC100066225,LOC100066190,LOC100066164,LOC100630861,LOC100066108,LOC100066068,LOC100147059,LOC100066040,LOC100062734,LOC100066011,LOC100065987,LOC100146657,LOC100065959,LOC100065934,LOC100147543,LOC100065911,LOC100065887,LOC100065858
12	16262155	16379687	117533	3	1	LOC100066417,LOC100066361
12	21568982	21582407	13426	3	1	
12	21786703	21817563	30861	3	1	LOC100062672
12	25586800	25752671	165872	3	1	LOC100057319,LOC100051469,LOC100057282,LOC100057238,LOC100057163,LTBP3,SCYL1,LOC100629234
12	25586800	25795367	208568	3	1	LOC100057444,PCNXL3,LOC100057319,LOC100051469,LOC100057282,LOC100057238,LOC100057163,LTBP3,SCYL1,LOC100629234
12	25742763	25795367	52605	1	1	LOC100057444,PCNXL3,LOC100057319,LOC100051469
12	33067635	33087564	19930	1	1	LOC100054956,LOC100051094,RIC8A,LOC100054908
13	211928	741571	529644	3	1	LOC100063055,TRRAP,SMURF1,KPNA7,ARPC1A,ARPC1B,LOC100063675
13	392585	543297	150713	3	1	TRRAP,SMURF1
13	851513	2523529	1672017	3	1	FBXL18,ACTB,LOC100146921,RNF216,LOC100061235,LOC100061318,LOC100061360,LOC100061391,ZNF12,LOC100630235,LOC100061463,LOC100061568,LOC100061601,LOC100061666,DAGLB,LOC100061732,LOC100630095,LOC100061834,LOC100061994,USP42,LOC100629209,LOC100062309,LOC100059133,PMS2,LOC100059180,LOC100062410,LOC100062477,LMTK2,LOC100062732,LOC100146317,LOC100629883,LOC100062881,LOC100062989
13	1691446	2365212	673767	3	1	RNF216,LOC100061235,LOC100061318,LOC100061360,LOC100061391,ZNF12,LOC100630235,LOC100061463,LOC100061568,LOC100061601,LOC100061666,DAGLB,LOC100061732,LOC100630095,LOC100061834
13	2014098	2038953	24856	1	1	LOC100061463
13	2032780	3337465	1304686	3	1	SDK1,FOXK1,LOC100060677,LOC100060713,LOC100060785,WIPI2,LOC100147021,LOC100060904,FBXL18,ACTB,LOC100146921,RNF216,LOC100061235,LOC100061318,LOC100061360,LOC100061391,ZNF12,LOC100630235,LOC100061463
13	2804980	2875776	70797	3	1	LOC100060713,LOC100060785
13	2804980	3003903	198924	3	1	FOXK1,LOC100060677,LOC100060713,LOC100060785
13	4174878	4812708	637831	3	1	LOC100059729,LOC100059762,LOC100147020,LOC100059822,LOC100059919,LOC100058937,AMZ1,LOC100060005,LOC100058981
13	4319066	4330016	10951	3	1	
13	4319066	4948823	629758	3	1	MAD1L1,LOC100059590,LOC100059629,SNX8,LOC100059693,LOC100059729,LOC100059762,LOC100147020,LOC100059822,LOC100059919,LOC100058937,AMZ1,LOC100060005,LOC100058981
13	5426293	5778195	351903	1	1	INTS1,LOC100053482,LOC100053430,LOC100053289
13	6295964	6344120	48157	3	1	PRKAR1B,LOC100054218
13	6340547	7474810	1134264	3	1	CYP3A97,CYP3A96,CYP3A95,CYP3A94,CYP3A89,CYP3A93,LOC100067876,ZNF498,LOC100630140,LOC100067831,LOC100147118,LOC100066997,ZNF394,LOC100067741,LOC100056828,LOC100055086,PRKAR1B
13	7283092	7369733	86642	3	1	CYP3A96
13	8228067	8278055	49989	3	3	LOC100147030
13	8331395	8670458	339064	3	1	LOC100069035,TFR2,MOSPD3,PCOLCE,LOC100067395,LRCH4,LOC100630713,LOC100069009,AGFG2,LOC100067349,LOC100068974,LOC100068962,LOC100068948,MEPCE,ZCWPW1,LOC100068911,STAG3,LOC100146435,LOC100067323,LOC100147030
13	9261143	12202508	2941366	3	1	LOC100062625,LOC100146522,GTF2I,LOC100629315,LOC100062277,LOC100062233,CLIP2,LOC100059980,LOC100062168,MIR590,LOC100062129,LIMK1,LOC100061996,LOC100061961,LOC100059948,LOC100059922,LOC100061925,LOC100061863,LOC100061799,LOC100146721,LOC100061734,MLXIPL,TBL2,LOC100061703,BAZ1B,LOC100061635,LOC100061393,LOC100059823,LOC100061362,LOC100061320,HIP1,LOC100630602,LOC100061208,LOC100061144,POR,LOC100061105,LOC100061080,MDH2,LOC100629706,LOC100059763,LOC100059731,SRCRB4D,LOC100060941,DTX2,LOC100060861,LOC100060825,RASA4,LOC100059695,LRWD1,LOC100059659,LOC100060645,LOC100059630,SH2B2,CUX1,LOC100060444,LOC100060372

Chrom	Start	End	Size	Cn	Samples (n)	Genes
13	9261143	15055288	5794146	3	1	AUTS2,GATSL2,LOC100063070,LOC100062935,LOC100062908,LOC100062883,LOC100062853,LOC100060039,LOC100062625,LOC100146522,GTF2I,LOC100629315,LOC100062277,LOC100062233,CLIP2,LOC100059980,LOC100062168,MIR590,LOC100062129,LIMK1,LOC100061996,LOC100061961,LOC100059948,LOC100059922,LOC100061925,LOC100061863,LOC100061799,LOC100146721,LOC100061734,MLXIPL,TBL2,LOC100061703,BAZ1B,LOC100061635,LOC100061393,LOC100059823,LOC100061362,LOC100061320,HIP1,LOC100630602,LOC100061208,LOC100061144,POR,LOC100061105,LOC100061080,MDH2,LOC100629706,LOC100059763,LOC100059731,SRCRB4D,LOC100060941,DTX2,LOC100060861,LOC100060825,RASA4,LOC100059695,LRWD1,LOC100059659,LOC100060645,LOC100059630,SH2B2,CUX1,LOC100060444,LOC100060372
13	9556299	9798399	242101	3	1	CUX1
13	11673353	11902171	228819	3	1	LOC100062233,CLIP2,LOC100059980,LOC100062168,MIR590,LOC100062129
13	12631220	13157372	526153	3	1	GATSL2,LOC100063070
13	12631220	21471569	8840350	3	1	LOC100066924,LOC100630264,JMJD5,LOC100066837,IL4R,IL21R,LOC100064518,LOC100066772,LOC100064490,XPO6,LOC100066611,LOC100066540,LOC100064430,LOC100066486,LOC100147176,LOC100147468,LOC100066402,ATP2A1,SH2B1,LOC100064341,ATXN2L,EIF3C,LOC100064282,LOC100066345,LOC100066317,LOC100064252,LOC100064194,LOC100064221,LOC100066262,BOLA2,CORO1A,LOC100066204,MAPK3,LOC100146273,LOC100064143,LOC100066147,ALDOA,PPP4C,LOC100066097,LOC100629851,DOC2A,LOC100064084,HIRIP3,TAOK2,LOC100064015,LOC100065972,LOC100065947,SEZ6L2,LOC100065899,MVP,LOC100063953,LOC100065869,LOC100146790,LOC100065842,LOC100065789,LOC100063917,LOC100063884,LOC100065768,LOC100065743,LOC100065719,TBC1D10B,LOC100065683,SEPT1,LOC100629627,LOC100065576,LOC100065556,SEPHS2,ITGAL,LOC100065483,LOC100065389,LOC100065362,PRR14,LOC100063732,SRCAP,LOC100063692,PHKG2,C13H16orf93,RNF40,ZNF629,LOC100063618,LOC100065094,LOC100065061,FBXL19,LOC100063559,SETD1A,LOC100063491,LOC100146591,LOC100063460,ZNF668,ZNF646,PRSS53,LOC100063360,LOC100063321,MYST1,PRSS36,LOC100063286,LOC100063252,LOC100063230,ITGAM,ITGAX,LOC100064753,LOC100063197,LOC100064673,TGFB1I1,LOC100064641,LOC100064608,LOC100630155,SEPT14,ZNF713,LOC100060658,LOC100061188,LOC100061297,CCT6A,LOC100061372,PHKG1,LOC100060727,LOC100629472,LOC100629446,GUSB,LOC100060764,ASL,LOC100060838,LOC100061681,TPST1,LOC100147567,LOC100061936,LOC100060879,LOC100060921,TYW1,LOC100062180,LOC100060956,LOC100060989,AUTS2,GATSL2,LOC100063070
13	12925292	13157372	232081	3	1	
13	13550445	13641080	90636	3	1	
13	13961628	13968758	7131	0,1	1,6	
13	14413556	15098531	684976	3	1	AUTS2
13	15632184	17238578	1606395	3	1	LOC100060879,LOC100060921,TYW1,LOC100062180,LOC100060956
13	16225425	17656980	1431556	3	1	TPST1,LOC100147567,LOC100061936,LOC100060879,LOC100060921,TYW1,LOC100062180,LOC100060956
13	18403940	18600549	196610	3	1	ITGAM,ITGAX,LOC100064753,LOC100063197,LOC100064673,TGFB1I1,LOC100064641,LOC100064608
13	18452358	21471569	3019212	3	1	LOC100066924,LOC100630264,JMJD5,LOC100066837,IL4R,IL21R,LOC100064518,LOC100066772,LOC100064490,XPO6,LOC100066611,LOC100066540,LOC100064430,LOC100066486,LOC100147176,LOC100147468,LOC100066402,ATP2A1,SH2B1,LOC100064341,ATXN2L,EIF3C,LOC100064282,LOC100066345,LOC100066317,LOC100064252,LOC100064194,LOC100064221,LOC100066262,BOLA2,CORO1A,LOC100066204,MAPK3,LOC100146273,LOC100064143,LOC100066147,ALDOA,PPP4C,LOC100066097,LOC100629851,DOC2A,LOC100064084,HIRIP3,TAOK2,LOC100064015,LOC100065972,LOC100065947,SEZ6L2,LOC100065899,MVP,LOC100063953,LOC100065869,LOC100146790,LOC100065842,LOC100065789,LOC100063917,LOC100063884,LOC100065768,LOC100065743,LOC100065719,TBC1D10B,LOC100065683,SEPT1,LOC100629627,LOC100065576,LOC100065556,SEPHS2,ITGAL,LOC100065483,LOC100065389,LOC100065362,PRR14,LOC100063732,SRCAP,LOC100063692,PHKG2,C13H16orf93,RNF40,ZNF629,LOC100063618,LOC100065094,LOC100065061,FBXL19,LOC100063559,SETD1A,LOC100063491,LOC100146591,LOC100063460,ZNF668,ZNF646,PRSS53,LOC100063360,LOC100063321,MYST1,PRSS36,LOC100063286,LOC100063252,LOC100063230,ITGAM,ITGAX,LOC100064753,LOC100063197
13	22035536	22431000	395465	3	1	ZKSCAN2
13	22946895	23301453	354559	3	1	LOC100068407,RBBP6,TNRC6A
13	23069966	23301453	231488	3	1	LOC100068407,RBBP6
13	23069966	24523942	1453977	3	1	HS3ST2,USP48,SCNN1B,LOC100068356,GGA2,EARS2,LOC100069079,PALB2,LOC100068373,LOC100068388,LOC100069133,LOC100069148,LOC100069215,LOC100068407,RBBP6
13	23505245	24759779	1254535	3	1	LOC100068328,LOC100068821,OTOA,HS3ST2,USP48,SCNN1B,LOC100068356,GGA2,EARS2,LOC100069079,PALB2,LOC100068373,LOC100068388,LOC100069133,LOC100069148,LOC100069215
13	23702481	24759779	1057299	3	1	LOC100068328,LOC100068821,OTOA,HS3ST2,USP48,SCNN1B,LOC100068356,GGA2,EARS2,LOC100069079,PALB2,LOC100068373,LOC100068388,LOC100069133,LOC100069148
13	25025789	25173629	147841	1	2	LOC100068270,LOC100068289,LOC100068628,ERI2,ACSM3
13	25333253	30314342	4981090	3	1	RRN3,LOC100053937,LOC100053887,LOC100630548,LOC100053782,LOC100050501,LOC100050421,MYH11,LOC100630456,ABCC1,ABCC6,LOC100053583,XYLT1,LOC100630410,LOC100050211,SMG1,LOC100052906,SYT17,LOC100050077,LOC100052804,TMC5,LOC100050012,CCP110,LOC100052236,LOC100049883,LOC100052174,LOC100052112,GPR139,GP2,LOC100051746,LOC100051683,ACSM5,LOC100067326,LOC100146491,LOC100146786,LOC100059261,LOC100034059,LOC100058079,LOC100630096,DNAH3,LOC100057992,LOC100059065,ANKS4B,LOC100057895,LOC100058987,LOC100058833,LOC100057847,LOC100058758,LOC100629263,VWA3A,SDR42E2,TRNAL-UAG,EEF2K,TRNAL-AAG,POLR3E,CDR2,LOC100057758,LOC100146885
13	26352563	26429577	77015	3	1	DNAH3,LOC100057992,LOC100059065,ANKS4B
13	27266469	27374902	108434	3	1	

Chrom	Start	End	Size	Cn	Samples (n)	Genes
13	27266469	42556495	15290027	3	1	IL9R,LOC100630131,LOC100066312,LOC100066340,LOC100065027,NPRL3,HBZ1,HBA,LOC100147150,LUC7L,LOC100066481,LOC100066511,LOC100146456,LOC100066556,AXIN1,LOC100066606,TMEM8A,LOC100066649,LOC100065146,LOC100066724,LOC100146243,PIGQ,LOC100066853,WFIKKN1,LOC100065210,TRNAG-CCC,LOC100629842,WDR90,RHOT2,RHBDL1,LOC100066977,LOC100066999,LOC100147047,WDR24,LOC100147621,LOC100147618,LOC100067088,LOC100067114,LOC100067135,LOC100065271,LOC100067178,LOC100067203,LOC100065297,LOC100067225,LOC100065327,LOC100067328,LOC100067353,LOC100067398,LOC100067422,LOC100067446,LOC100067492,LOC100067538,RET,LOC100065385,LOC100067554,LOC100065411,LOC100067580,LOC100067623,CCDC154,LOC100067691,PTX4,LOC100067764,LOC100065461,IFT140,LOC100065479,LOC100065505,MAPK8IP3,LOC100146137,LOC100146452,LOC100067860,LOC100065531,LOC100067912,LOC100067938,LOC100067958,LOC100146640,SEPX1,LOC100068030,LOC100065553,LOC100065572,LOC100068047,TBL3,LOC100068060,LOC100065617,LOC100065638,ZNF598,LOC100629171,LOC100146142,LOC100065659,TSC2,LOC100068126,RAB26,TRAF7,CASKIN1,LOC100068216,LOC100068232,MIR1842,LOC100068257,LOC100068273,LOC100065713,LOC100068292,LOC100065738,LOC100068313,LOC100068332,LOC100068378,LOC100068393,LOC100147434,TBC1D24,LOC100065783,LOC100068441,LOC100630736,LOC100068494,LOC100068526,LOC100068549,LOC100630678,LOC100630656,LOC100068644,LOC100630613,LOC100630594,LOC100068690,LOC100068704,LOC100068723,LOC100068741,LOC100068757,LOC100068774,LOC100068790,LOC100629991,LOC100068826,LOC100068842,LOC100146843,PKMYT1,LOC100067620,LOC100067643,LOC100146755,LOC100065863,LOC100068915,LOC100068930,LOC100068942,LOC100065894,ZSCAN10,LOC100068965,LOC100068977,TRNAR-CCG,TRNAR-CCU,TRNAK-CUU,TRNAP-UGG,TRNAP-AGG,TRNAK-CUU,TRNAP-AGG,TRNAK-CUU,TRNAP-UGG,TRNAK-CUU,TRNAP-CGG,TRNAR-CCU,TRNAK-CUU,TRNAK-CUU,TRNAP-UGG,TRNAP-AGG,TRNAK-CUU,TRNAP-AGG,TRNAR-CCU,TRNAK-CUU,LOC100069220,LOC100069235,LOC100069249,ZNF200,LOC100069286,ZNF263,LOC100069369,LOC100069382,LOC100069406,LOC100630058,LOC100069430,ZNF174,LOC100069444,LOC100065966
13	28665470	28686494	21025	3	1	XYLT1
13	28665470	29091166	425697	3	1	XYLT1
13	29574105	30284396	710292	3	1	LOC100053887,LOC100630548,LOC100053782,LOC100050501,LOC100050421,MYH11,LOC100630456,ABCC1,ABCC6,LOC100053583
13	30219094	30284396	65303	1	1	LOC100053887
13	30700736	32378825	1678090	3	1	LOC100055746,LOC100055616,LOC100147264,LOC100050888,ERCC4,MKL2
13	30848866	31063558	214693	3	1	ERCC4,MKL2
13	31854826	31871959	17134	1	1	LOC100147264
13	32182316	32250963	68648	3	1	LOC100055746
13	33005383	33299329	293947	3	1	LOC100051107,TNP2,LOC100056369,LOC100056327,PRM1
13	33005383	38910323	5904941	3	1	ADCY9,LOC100069703,LOC100069711,GLIS2,LOC100629909,LOC100069750,DNAJA3,LOC100069771,LOC100629857,LOC100066114,LOC100069792,LOC100147530,LOC100069818,ANKS3,LOC100066170,LOC100069865,LOC100069881,LOC100147335,GLYR1,UBN1,PPL,SEC14L5,LOC100069983,LOC100069997,LOC100147524,LOC100070021,RBFOX1,LOC100070199,MIR1302E-7,METTL22,ABAT,LOC100057560,LOC100057523,LOC100051399,USP7,LOC100057445,LOC100147369,LOC100051329,LOC100057283,LOC100630263,ATF7IP2,LOC100051255,TEKT5,LOC100056639,LOC100056589,CIITA,LOC100051182,CLEC16A,LOC100051107,TNP2,LOC100056369,LOC100056327,PRM1
13	33730497	34520794	790298	3	1	LOC100051329,LOC100057283,LOC100630263,ATF7IP2,LOC100051255
13	34894181	34963718	69538	3	1	USP7,LOC100057445
13	35814914	35860491	45578	3	1	
13	36239587	37659674	1420088	3	1	RBFOX1
13	37634702	37659674	24973	3	1	
13	38387299	38518390	131092	3	1	DNAJA3,LOC100069771,LOC100629857,LOC100066114,LOC100069792,LOC100147530
13	39491095	42556495	3065401	3	1	IL9R,LOC100630131,LOC100066312,LOC100066340,LOC100065027,NPRL3,HBZ1,HBA,LOC100147150,LUC7L,LOC100066481,LOC100066511,LOC100146456,LOC100066556,AXIN1,LOC100066606,TMEM8A,LOC100066649,LOC100065146,LOC100066724,LOC100146243,PIGQ,LOC100066853,WFIKKN1,LOC100065210,TRNAG-CCC,LOC100629842,WDR90,RHOT2,RHBDL1,LOC100066977,LOC100066999,LOC100147047,WDR24,LOC100147621,LOC100147618,LOC100067088,LOC100067114,LOC100067135,LOC100065271,LOC100067178,LOC100067203,LOC100065297,LOC100067225,LOC100065327,LOC100067328,LOC100067353,LOC100067398,LOC100067422,LOC100067446,LOC100067492,LOC100067538,RET,LOC100065385,LOC100067554,LOC100065411,LOC100067580,LOC100067623,CCDC154,LOC100067691,PTX4,LOC100067764,LOC100065461,IFT140,LOC100065479,LOC100065505,MAPK8IP3,LOC100146137,LOC100146452,LOC100067860,LOC100065531,LOC100067912,LOC100067938,LOC100067958,LOC100146640,SEPX1,LOC100068030,LOC100065553,LOC100065572,LOC100068047,TBL3,LOC100068060,LOC100065617,LOC100065638,ZNF598,LOC100629171,LOC100146142,LOC100065659,TSC2,LOC100068126,RAB26,TRAF7,CASKIN1,LOC100068216,LOC100068232,MIR1842,LOC100068257,LOC100068273,LOC100065713,LOC100068292,LOC100065738,LOC100068313,LOC100068332,LOC100068378,LOC100068393,LOC100147434,TBC1D24,LOC100065783,LOC100068441,LOC100630736,LOC100068494,LOC100068526,LOC100068549,LOC100630678,LOC100630656,LOC100068644,LOC100630613,LOC100630594,LOC100068690,LOC100068704,LOC100068723,LOC100068741,LOC100068757,LOC100068774,LOC100068790,LOC100629991,LOC100068826,LOC100068842,LOC100146843,PKMYT1,LOC100067620,LOC100067643,LOC100146755,LOC100065863,LOC100068915,LOC100068930,LOC100068942,LOC100065894,ZSCAN10,LOC100068965,LOC100068977,TRNAR-CCG,TRNAR-CCU,TRNAK-CUU,TRNAP-UGG,TRNAP-AGG,TRNAK-CUU,TRNAP-AGG,TRNAK-CUU,TRNAP-UGG,TRNAK-CUU,TRNAP-CGG,TRNAR-CCU,TRNAK-CUU,TRNAK-CUU,TRNAP-UGG,TRNAP-AGG,TRNAK-CUU,TRNAP-AGG,TRNAR-CCU,TRNAK-CUU,LOC100069220,LOC100069235,LOC100069249,ZNF200,LOC100069286,ZNF263
13	39776251	39809726	33476	1	1	PKMYT1

Chrom	Start	End	Size	Cn	Samples (n)	Genes
13	39934602	40505245	570644	3	1	LOC100068313,LOC100068332,LOC100068378,LOC100068393,LOC100147434,TBC1D24,LOC100065783,LOC100068441,LOC100630736,LOC100068494,LOC100068526,LOC100068549,LOC100630678,LOC100630656,LOC100068644,LOC100630613,LOC100630594,LOC100068690,LOC100068704,LOC100068723,LOC100068741
13	41502477	41615284	112808	1	1	LOC100067422,LOC100067446,LOC100067492
13	41613568	41943528	329961	3	1	LOC100147618,LOC100067088,LOC100067114,LOC100067135,LOC100065271,LOC100067178,LOC100067203,LOC100065297,LOC100067225,LOC100065327,LOC100067328,LOC100067353,LOC100067398
13	42210951	42247375	36425	1	1	TMEM8A,LOC100066649,LOC100065146
13	42316283	42462353	146071	3	1	HBZ1,HBA,LOC100147150,LUC7L,LOC100066481,LOC100066511,LOC100146456,LOC100066556
14	1296596	1300439	3844	0,3	1,2	LOC100066054
14	1296596	1392437	95842	1	1	LOC100066263,LOC100066238,LOC100066205,TRNAL-AAG,TRNAP-UGG,TRNAT-UGU,LOC100066054
14	8526653	8547630	20978	3	2	LOC100069822,LOC100059037
14	15779185	15836399	57215	1	1	
14	17713486	17826590	113105	1	2	GABRG2
14	18178216	18257717	79502	1	2	GABRA6
14	22810504	22885019	74516	1	1	
14	22810504	22888581	78078	1	1	
14	26211323	26230807	19485	3	1	
14	28687853	28701074	13222	1	1	ABLIM3
14	31707567	31745992	38426	1	1	SH3RF2
14	38340380	38353956	13577	3	2	
14	39890186	39896829	6644	3	2	
14	44955903	44985427	29525	3	1	CHSY3
14	52997893	53276093	278201	3	1	
14	55252399	55256056	3658	3	1	
14	59460837	59463001	2165	3	10	
14	64933907	64933987	81	1	1	
14	64933907	64951664	17758	1	3	
14	73064956	73068761	3806	1	1	
14	79224940	79229181	4242	1	1	TMEM161B
14	85242665	85274390	31726	1	1	LOC100073258,ANKRD34B
14	85989014	86057100	68087	1	1	HOMER1
15	3116498	3120500	4003	1	1	
15	8887106	8890034	2929	1	1	LOC100630166
15	13516385	13628060	111676	3	2	LOC100629216,LOC100063525
15	20981744	21139258	157515	1	1	
15	21073741	21139258	65518	1	2	
15	21073741	21147828	74088	1	1	
15	25242117	25297836	55720	1	1	
15	30066460	30159309	92850	3	1	EXOC6B
15	35232680	35233452	773	1	1	
15	38257100	38267679	10580	1	2	LOC100063987
15	39607619	39700212	92594	1	1	LOC100051994
15	48199824	48278494	78671	1	1	
15	51958226	51973518	15293	3	2	TTC7A
15	55546040	55553663	7624	3	2	
15	56513560	57018352	504793	1	1	LOC100629144
15	57861323	58074504	213182	1	1	SLC8A1
15	57914331	57914904	574	1	1	SLC8A1
15	58977518	58988902	11385	1	1	SOS1
15	64551633	64631484	79852	1	5	LOC100054514,LOC100070614
15	68861626	68953995	92370	3	1	LOC100629358,GTF3C2,EIF2B4,SNX17,ZNF513,LOC100055191,NRBP1
15	77767792	77771242	3451	1	1	

Chrom	Start	End	Size	Cn	Samples (n)	Genes
15	90795121	90843987	48867	3	1	
15	90838706	90843987	5282	3	1	
16	2329497	2334710	5214	3	1	NUP210
16	15132562	15134110	1549	1	1	
16	21802671	21866735	64065	3	1	
16	26921908	26923109	1202	1	1	PTPRG
16	28573346	28579587	6242	1	1	LOC100057165
16	34541548	34552364	10817	1	1	DCP1A
16	35599532	35644290	44759	3	2	LOC100060559,LOC100060527
16	38646249	38748409	102161	1	2	ECATH-3,LOC100063988,LOC100630242
16	38714465	38748409	33945	1	4	
16	47816883	47827828	10946	1	3	GOLGA4
16	78063845	78067333	3489	1	1	
16	80679310	80681397	2088	1	7	
16	86046178	86080644	34467	3	1	LOC100054189
17	21128569	21187559	58991	1	1	TRIM13,LOC100050085,MIR16-2,MIR15A
17	32426470	32635391	208922	3	1	
17	32559665	32654057	94393	3	5	
17	36846001	36852057	6057	3	1	
17	36846001	36905058	59058	1	2	
17	36846001	36967536	121536	1	1	LOC100063921
17	38281077	38472830	191754	1	2	
17	38344298	38472830	128533	1	1	
17	41966673	42030550	63878	1	1	
17	42799674	42807283	7610	1	1	
17	43934442	43951803	17362	1	1	
17	44330520	44330796	277	1	1	
17	48284850	48603600	318751	1	1	LOC100051876,TBC1D4,LOC100051692
17	48562840	48603600	40761	1	1	LOC100051876,TBC1D4
17	49997510	50011096	13587	1	2	LOC100053014
17	50758361	50815805	57445	3	1	
17	52880895	53071134	190240	1	1	
17	52981638	52982220	583	1	2	
17	52981638	53071134	89497	1	2	
17	52981638	53539910	558273	1	1	
17	53536332	53539910	3579	1	2	
17	54202284	54213979	11696	1	1	
17	54626730	54820870	194141	1	1	LOC100050152
17	56394003	56433502	39500	1	2	LOC100056206
17	56596949	56619836	22888	1	1	
17	57317295	57338181	20887	1	1	
17	57317295	57420379	103085	1	3	
17	57337550	57338181	632	1,3	2,1	
17	57337550	57420379	82830	1	4	
17	58048425	58048581	157	1	3	
17	58048425	58094803	46379	1	3	
17	60468135	60625921	157787	1	1	
17	60577739	60625921	48183	1	2	
17	61466128	61571146	105019	1	1	LOC100057915
17	67785183	67810479	25297	3	1	LOC100050664
17	69818185	69931632	113448	1	1	NALCN

Chrom	Start	End	Size	Cn	Samples (n)	Genes
17	73435446	73444774	9329	1	1	
17	74662387	74671901	9515	1	1	
17	74669703	74671901	2199	1	1	
17	75380632	75401514	20883	1	1	LOC100051408
18	11489634	12345200	855567	3	1	LOC100052304,LOC100052243,LOC100049893,STEAP3,LOC100629302,DBI,LOC100051693,LOC100051620,LOC100147031
18	11660478	12399073	738596	3	1	LOC100052304,LOC100052243,LOC100049893,STEAP3,LOC100629302,DBI
18	11660478	12402925	742448	3	1	LOC100052304,LOC100052243,LOC100049893,STEAP3,LOC100629302,DBI
18	19901232	19908419	7188	1	1	LOC100050974
18	24608600	24753371	144772	1	1	
18	24866467	24870435	3969	1	5	
18	25534223	25595772	61550	1	2	
18	35059332	35071905	12574	1	1	
18	36449291	36449543	253	1	2	
18	36449291	36572899	123609	1	1	LOC100058814
18	36449291	36636661	187371	1	1	LOC100058814
18	36449291	36674443	225153	1	1	LOC100058814
18	37589241	37625592	36352	3	2	TRNAG-GCC,TRNAA-CGC
18	38240449	38244258	3810	1	1	
18	46009970	46010221	252	1	2	TTC21B
18	48311209	48338940	27732	1	1	LASS6
18	49467480	49493092	25613	1	1	UBR3
18	60519874	60601238	81365	3	1	NCKAP1
18	61648648	61806107	157460	1	1	ZNF804A
18	64342440	64391698	49259	1	1	
18	73734487	73765080	30594	1	2	
18	75760478	75879559	119082	3	1	LOC100067956,LOC100067782
18	75780975	75864062	83088	3	1	LOC100067956,LOC100067782
18	75813682	75864062	50381	3	13	LOC100067956,LOC100067782
19	1326805	1396027	69223	3	2	
19	1346278	1396027	49750	3	3	
19	1349908	1396027	46120	3	3	
19	5012965	5089359	76395	1	8	
19	5012965	5125435	112471	1	1	
19	15740333	15774140	33808	1	1	
19	16177301	16177592	292	1	2	
19	18782838	18902195	119358	3	1	PEX5L,USP13
19	32598913	32600181	1269	0,1	9,28	DLG1
19	32598913	32621031	22119	1	7	DLG1
19	32598913	32638781	39869	1	1	DLG1
19	44094093	44155141	61049	1	1	LOC100061223,GRAMD1C
19	51921242	51991217	69976	1	1	
19	51921242	52190199	268958	1	1	
19	53868012	53875340	7329	1	1	ZBTB11
19	54585286	54585597	312	1	1	ABI3BP
20	26411124	26498430	87307	3	12	LOC100062341,LOC100062378,LOC100062409,LOC100062441,LOC100062476
20	27984017	28089836	105820	1	2	LOC100058467,LOC100058512,LOC100058550,LOC100058588,LOC100058631,LOC100058670
20	27984017	28206968	222952	1	3	LOC100058118,LOC100058164,LOC100058205,LOC100058247,LOC100058298,LOC100147391,LOC100058338,LOC100058379,LOC100058467,LOC100058512,LOC100058550,LOC100058588,LOC100058631,LOC100058670
20	29191096	29205938	14843	1,3	2,1	LOC100629124
20	31961012	32182629	221618	1	1	LOC100059844,LOC100059681,LOC100059644
20	32059082	32182629	123548	1	2	LOC100059844,LOC100059681

Chrom	Start	End	Size	Cn	Samples (n)	Genes
20	32059082	32210308	151227	0,1	1,4	LOC100059844,LOC100059681
20	32127612	32131071	3460	0,3	1,1	
20	32127612	32182629	55018	1	1	
20	32127612	32210308	82697	0,1	2,9	
20	32131071	32210308	79238	0	4	
20	34355448	34369591	14144	3	1	LOC100061879
20	34355448	34396948	41501	3	1	LOC100061879
20	45342508	45347364	4857	1	3	
20	48306105	48355432	49328	1	2	
20	50391131	50479786	88656	1	1	LOC100056462,LOC100056418,LOC100271875,LOC100629530,LOC100069298
20	52229057	52268858	39802	3	1	LOC100069643
21	2851979	3058622	206644	1	1	LOC100070963,LOC100070946,LOC100070928,LOC100070916,LOC100070899,LOC100069542,LOC100070886,LOC100070878,LOC100146348,LOC100146664
21	4172122	4271646	99525	1	1	LOC100054975
21	15292338	15309477	17140	1	2	
21	15306038	15309477	3440	1	1	
21	18811395	18814925	3531	3	4	
21	20111647	20377024	265378	1	1	ISL1
21	28144819	28335214	190396	1	1	NIPBL,LOC100053501,LOC100053450,NUP155
21	29764312	29785036	20725	1	1	
21	35205768	35230144	24377	1,3	3,2	LOC100069241
21	36213224	36345862	132639	3	1	LOC100054286
21	37492026	37503320	11295	1	1	
21	42207465	42211062	3598	1	1	
21	48275858	48291155	15298	1	1	CTNND2
21	52110779	52114401	3623	1	1	
22	12733165	12749137	15973	1	1	TRNAV-UAC
22	15434589	15483680	49092	1	1	LOC100051558
22	20922073	20926117	4045	3	10	LOC100067431
22	28439582	28451955	12374	3	2	LOC100070061,LOC100070046
22	36384241	36545238	160998	3	3	
22	36435729	36545238	109510	3	8	
22	36965784	36975141	9358	1	1	
22	45748491	45749595	1105	1	1	
22	45748491	45759208	10718	1	1	
22	49007497	49358382	350886	4	1	GMEB2,LOC100060598,LOC100146686,SRMS,PTK6,LOC100630715,EEF1A2,KCNQ2,LOC100147373,YTHDF1
23	231952	264863	32912	1	1	LOC100146373
23	7973293	8450458	477166	3	1	LOC100055310,LOC100055131,LOC100055085,LOC100054860
23	9371048	9485560	114513	1	1	LOC100070085
23	16784961	16799292	14332	1	1	
23	16945569	17031995	86427	3	1	
23	17021853	17100602	78750	1	6	
23	18031161	18042294	11134	0,3	1,2	
23	47584956	47585275	320	1	2	
23	47893418	47984323	90906	1	1	
24	23383820	23401888	18069	1	1	
24	26057366	26087678	30313	3	1	STON2
24	27995825	28016609	20785	1	2	
24	32416012	32508738	92727	3	6	TTC8,EML5
24	32416012	32628728	212717	3	2	TTC8,EML5
24	38370785	38375555	4771	3	21	
24	44638931	44699916	60986	1	1	LOC100146644,EXOC3L4,CDC42BPB

Chrom	Start	End	Size	Cn	Samples (n)	Genes
24	46150523	46186716	36194	3	1	
25	10383396	10385144	1749	3	1	
25	10383396	10424522	41127	3	3	
25	24753354	24753701	348	1	1	LOC100067655
25	24753354	24907656	154303	1	1	LOC100067611,LOC100071754,LOC100067633,LOC100067655
25	26318531	26599435	280905	1	1	LOC100071402,LOC100071407,LOC100071413,LOC100071420,LOC100146823,LOC100071438,LOC100071445,LOC100071448,LOC100071452,LOC100071460,LOC100071469,LOC100071479,LOC100071486,LOC100071488,LOC100071492,LOC100071496,LOC100071502
25	26318531	26918263	599733	1	2	LOC100071251,LOC100067520,LOC100071258,LOC100071264,LOC100071270,LOC100071275,LOC100071278,LOC100071283,LOC100071287,LOC100071297,LOC100146817,LOC100071311,LOC100071317,LOC100147676,LOC100071322,LOC100071329,LOC100071332,LOC100071338,LOC100071346,LOC100071352,LOC100071358,LOC100071365,LOC100071376,LOC100071382,LOC100071392,LOC100071402,LOC100071407,LOC100071413,LOC100071420,LOC100146823,LOC100071438,LOC100071445,LOC100071448,LOC100071452,LOC100071460,LOC100071469,LOC100071479,LOC100071486,LOC100071488,LOC100071492,LOC100071496,LOC100071502
25	26318531	26942120	623590	1	2	LOC100071244,LOC100071251,LOC100067520,LOC100071258,LOC100071264,LOC100071270,LOC100071275,LOC100071278,LOC100071283,LOC100071287,LOC100071297,LOC100146817,LOC100071311,LOC100071317,LOC100147676,LOC100071322,LOC100071329,LOC100071332,LOC100071338,LOC100071346,LOC100071352,LOC100071358,LOC100071365,LOC100071376,LOC100071382,LOC100071392,LOC100071402,LOC100071407,LOC100071413,LOC100071420,LOC100146823,LOC100071438,LOC100071445,LOC100071448,LOC100071452,LOC100071460,LOC100071469,LOC100071479,LOC100071486,LOC100071488,LOC100071492,LOC100071496,LOC100071502
25	26318531	27125754	807224	1	1	RC3H2,LOC100071170,LOC100071180,LOC100067499,LOC100071189,LOC100630709,LOC100071212,LOC100071218,LOC100071227,LOC100071236,LOC100071244,LOC100071251,LOC100067520,LOC100071258,LOC100071264,LOC100071270,LOC100071275,LOC100071278,LOC100071283,LOC100071287,LOC100071297,LOC100146817,LOC100071311,LOC100071317,LOC100147676,LOC100071322,LOC100071329,LOC100071332,LOC100071338,LOC100071346,LOC100071352,LOC100071358,LOC100071365,LOC100071376,LOC100071382,LOC100071392,LOC100071402,LOC100071407,LOC100071413,LOC100071420,LOC100146823,LOC100071438,LOC100071445,LOC100071448,LOC100071452,LOC100071460,LOC100071469,LOC100071479,LOC100071486,LOC100071488,LOC100071492,LOC100071496,LOC100071502
25	26361000	26512888	151889	1	3	LOC100146823,LOC100071438,LOC100071445,LOC100071448,LOC100071452,LOC100071460,LOC100071469,LOC100071479,LOC100071486
25	26361000	26560311	199312	1	3	LOC100071413,LOC100071420,LOC100146823,LOC100071438,LOC100071445,LOC100071448,LOC100071452,LOC100071460,LOC100071469,LOC100071479,LOC100071486
25	26361000	26751364	390365	1	1	LOC100146817,LOC100071311,LOC100071317,LOC100147676,LOC100071322,LOC100071329,LOC100071332,LOC100071338,LOC100071346,LOC100071352,LOC100071358,LOC100071365,LOC100071376,LOC100071382,LOC100071392,LOC100071402,LOC100071407,LOC100071413,LOC100071420,LOC100146823,LOC100071438,LOC100071445,LOC100071448,LOC100071452,LOC100071460,LOC100071469,LOC100071479,LOC100071486
25	26361000	26918263	557264	1	2	LOC100071251,LOC100067520,LOC100071258,LOC100071264,LOC100071270,LOC100071275,LOC100071278,LOC100071283,LOC100071287,LOC100071297,LOC100146817,LOC100071311,LOC100071317,LOC100147676,LOC100071322,LOC100071329,LOC100071332,LOC100071338,LOC100071346,LOC100071352,LOC100071358,LOC100071365,LOC100071376,LOC100071382,LOC100071392,LOC100071402,LOC100071407,LOC100071413,LOC100071420,LOC100146823,LOC100071438,LOC100071445,LOC100071448,LOC100071452,LOC100071460,LOC100071469,LOC100071479,LOC100071486
25	26361000	26942120	581121	0,1	4,12	LOC100071244,LOC100071251,LOC100067520,LOC100071258,LOC100071264,LOC100071270,LOC100071275,LOC100071278,LOC100071283,LOC100071287,LOC100071297,LOC100146817,LOC100071311,LOC100071317,LOC100147676,LOC100071322,LOC100071329,LOC100071332,LOC100071338,LOC100071346,LOC100071352,LOC100071358,LOC100071365,LOC100071376,LOC100071382,LOC100071392,LOC100071402,LOC100071407,LOC100071413,LOC100071420,LOC100146823,LOC100071438,LOC100071445,LOC100071448,LOC100071452,LOC100071460,LOC100071469,LOC100071479,LOC100071486
25	26361000	27125754	764755	1	4	RC3H2,LOC100071170,LOC100071180,LOC100067499,LOC100071189,LOC100630709,LOC100071212,LOC100071218,LOC100071227,LOC100071236,LOC100071244,LOC100071251,LOC100067520,LOC100071258,LOC100071264,LOC100071270,LOC100071275,LOC100071278,LOC100071283,LOC100071287,LOC100071297,LOC100146817,LOC100071311,LOC100071317,LOC100147676,LOC100071322,LOC100071329,LOC100071332,LOC100071338,LOC100071346,LOC100071352,LOC100071358,LOC100071365,LOC100071376,LOC100071382,LOC100071392,LOC100071402,LOC100071407,LOC100071413,LOC100071420,LOC100146823,LOC100071438,LOC100071445,LOC100071448,LOC100071452,LOC100071460,LOC100071469,LOC100071479,LOC100071486
25	26393590	26560311	166722	1	2	LOC100071413,LOC100071420,LOC100146823,LOC100071438,LOC100071445,LOC100071448,LOC100071452,LOC100071460,LOC100071469
25	26393590	26918263	524674	1	1	LOC100071251,LOC100067520,LOC100071258,LOC100071264,LOC100071270,LOC100071275,LOC100071278,LOC100071283,LOC100071287,LOC100071297,LOC100146817,LOC100071311,LOC100071317,LOC100147676,LOC100071322,LOC100071329,LOC100071332,LOC100071338,LOC100071346,LOC100071352,LOC100071358,LOC100071365,LOC100071376,LOC100071382,LOC100071392,LOC100071402,LOC100071407,LOC100071413,LOC100071420,LOC100146823,LOC100071438,LOC100071445,LOC100071448,LOC100071452,LOC100071460,LOC100071469
25	26509315	26599435	90121	0,1	1,1	LOC100071402,LOC100071407,LOC100071413,LOC100071420,LOC100146823
25	26509315	26647861	138547	0,1	1,1	LOC100071352,LOC100071358,LOC100071365,LOC100071376,LOC100071382,LOC100071392,LOC100071402,LOC100071407,LOC100071413,LOC100071420,LOC100146823
25	26509315	26751364	242050	1	1	LOC100146817,LOC100071311,LOC100071317,LOC100147676,LOC100071322,LOC100071329,LOC100071332,LOC100071338,LOC100071346,LOC100071352,LOC100071358,LOC100071365,LOC100071376,LOC100071382,LOC100071392,LOC100071402,LOC100071407,LOC100071413,LOC100071420,LOC100146823
25	26509315	26918263	408949	1	10	LOC100071251,LOC100067520,LOC100071258,LOC100071264,LOC100071270,LOC100071275,LOC100071278,LOC100071283,LOC100071287,LOC100071297,LOC100146817,LOC100071311,LOC100071317,LOC100147676,LOC100071322,LOC100071329,LOC100071332,LOC100071338,LOC100071346,LOC100071352,LOC100071358,LOC100071365,LOC100071376,LOC100071382,LOC100071392,LOC100071402,LOC100071407,LOC100071413,LOC100071420,LOC100146823

Chrom	Start	End	Size	Cn	Samples (n)	Genes
25	26509315	26942120	432806	0,1	1,21	LOC100071244,LOC100071251,LOC100067520,LOC100071258,LOC100071264,LOC100071270,LOC100071275,LOC100071278,LOC100071283,LOC100071287,LOC100071297,LOC100146817,LOC100071311,LOC100071317,LOC100147676,LOC100071322,LOC100071329,LOC100071332,LOC100071338,LOC100071346,LOC100071352,LOC100071358,LOC100071365,LOC100071376,LOC100071382,LOC100071392,LOC100071402,LOC100071407,LOC100071413,LOC100071420,LOC100146823
25	26560311	26599435	39125	0	2	LOC100071402,LOC100071407
25	26560311	26751364	191054	0,1	1,2	LOC100146817,LOC100071311,LOC100071317,LOC100147676,LOC100071322,LOC100071329,LOC100071332,LOC100071338,LOC100071346,LOC100071352,LOC100071358,LOC100071365,LOC100071376,LOC100071382,LOC100071392,LOC100071402,LOC100071407
25	26560311	26866414	306104	1	2	LOC100071270,LOC100071275,LOC100071278,LOC100071283,LOC100071287,LOC100071297,LOC100146817,LOC100071311,LOC100071317,LOC100147676,LOC100071322,LOC100071329,LOC100071332,LOC100071338,LOC100071346,LOC100071352,LOC100071358,LOC100071365,LOC100071376,LOC100071382,LOC100071392,LOC100071402,LOC100071407
25	26560311	26942120	381810	1	4	LOC100071244,LOC100071251,LOC100067520,LOC100071258,LOC100071264,LOC100071270,LOC100071275,LOC100071278,LOC100071283,LOC100071287,LOC100071297,LOC100146817,LOC100071311,LOC100071317,LOC100147676,LOC100071322,LOC100071329,LOC100071332,LOC100071338,LOC100071346,LOC100071352,LOC100071358,LOC100071365,LOC100071376,LOC100071382,LOC100071392,LOC100071402,LOC100071407
25	26560311	27125754	565444	1	1	RC3H2,LOC100071170,LOC100071180,LOC100067499,LOC100071189,LOC100630709,LOC100071212,LOC100071218,LOC100071227,LOC100071236,LOC100071244,LOC100071251,LOC100067520,LOC100071258,LOC100071264,LOC100071270,LOC100071275,LOC100071278,LOC100071283,LOC100071287,LOC100071297,LOC100146817,LOC100071311,LOC100071317,LOC100147676,LOC100071322,LOC100071329,LOC100071332,LOC100071338,LOC100071346,LOC100071352,LOC100071358,LOC100071365,LOC100071376,LOC100071382,LOC100071392,LOC100071402,LOC100071407
25	26599255	26751364	152110	1	1	LOC100146817,LOC100071311,LOC100071317,LOC100147676,LOC100071322,LOC100071329,LOC100071332,LOC100071338,LOC100071346,LOC100071352,LOC100071358,LOC100071365,LOC100071376,LOC100071382,LOC100071392
25	26647861	26751364	103504	1	1	LOC100146817,LOC100071311,LOC100071317,LOC100147676,LOC100071322,LOC100071329,LOC100071332,LOC100071338,LOC100071346
25	26647861	26918263	270403	1	2	LOC100071251,LOC100067520,LOC100071258,LOC100071264,LOC100071270,LOC100071275,LOC100071278,LOC100071283,LOC100071287,LOC100071297,LOC100146817,LOC100071311,LOC100071317,LOC100147676,LOC100071322,LOC100071329,LOC100071332,LOC100071338,LOC100071346
25	26647861	26942120	294260	1	2	LOC100071244,LOC100071251,LOC100067520,LOC100071258,LOC100071264,LOC100071270,LOC100071275,LOC100071278,LOC100071283,LOC100071287,LOC100071297,LOC100146817,LOC100071311,LOC100071317,LOC100147676,LOC100071322,LOC100071329,LOC100071332,LOC100071338,LOC100071346
25	26750874	26751364	491	1,3	1,2	
25	26750874	26866414	115541	1	1	LOC100071270,LOC100071275,LOC100071278,LOC100071283,LOC100071287,LOC100071297
25	26750874	26918263	167390	0,1	1,1	LOC100071251,LOC100067520,LOC100071258,LOC100071264,LOC100071270,LOC100071275,LOC100071278,LOC100071283,LOC100071287,LOC100071297
25	26750874	26942120	191247	1	6	LOC100071244,LOC100071251,LOC100067520,LOC100071258,LOC100071264,LOC100071270,LOC100071275,LOC100071278,LOC100071283,LOC100071287,LOC100071297
25	26866414	26918263	51850	1	7	LOC100071251,LOC100067520,LOC100071258,LOC100071264
25	26866414	26942120	75707	0,1,3	2,3,1	LOC100071244,LOC100071251,LOC100067520,LOC100071258,LOC100071264
25	26891173	26942120	50948	1	2	LOC100071244,LOC100071251,LOC100067520
25	29109453	29120366	10914	3	1	GAPVD1
25	29618659	29621832	3174	3	16	LOC100070756
25	29618659	29684315	65657	3	1	LOC100629330,LOC100070756
25	30085691	30107604	21914	1	1	LOC100070649
25	34218757	34239064	20308	1	1	
25	36451809	36495958	44150	3	1	LOC100069057,LOC100069096
25	36784230	36806253	22024	1	1	LOC100069057
26	1600966	1724169	123204	1	1	EPHA3
26	12021987	12033200	11214	1	1	ROBO2
26	13391944	13474118	82175	1	1	LOC100068747,LIPI
26	21312489	21381972	69484	1	2	
26	28489917	28537733	47817	1	1	
26	39973069	40138871	165803	3	3	LOC100050166,LOC100056049,ITGB2,LOC100630038
26	40183063	40249367	66305	1	1	
26	41794508	41819496	24989	4	1	LOC100054611
27	1073237	1098652	25416	1	1	
27	1073237	2224038	1150802	3	1	CSGALNACT1,SH2D4A,TRNAA-UGC,PSD3
27	3172279	5381712	2209434	3	1	LOC100056890,LOC100056776,LOC100050681,IDO2,LOC100050602,LOC100056469,LOC100055845,LOC100050524,LOC100055757,AGPAT6,LOC100055502,MIR486,ANK1,LOC100630444,MYST3,LOC100054750,AP3M2,PLAT,LOC100630402,LOC100050167
27	5750034	5910085	160052	3	1	ADAM32
27	5910085	5928605	18521	3	3	ADAM32

Chrom	Start	End	Size	Cn	Samples (n)	Genes
27	6539738	6667310	127573	3	1	ADAM2
27	6912701	7982098	1069398	3	1	LOC100146543,LOC100057973,LOC100059780,GPR124,ZFP36L2,RAB11FIP1,LOC100059580,LOC100147322,LOC100057875,ASH2L,STAR,LOC100057784,BAG4,DDHD2,LOC100059204,WHSC1L1,LETM2,FGFR1,TACC1,LOC100057530
27	8257142	14319372	6062231	3	1	LOC100062785,LOC100062721,MBOAT4,LOC100058409,LOC100058367,LOC100058323,LOC100062365,LOC100058282,PPP2CB,TEX15,LOC100058235,PURG,LOC100062021,NRG1,LOC100061264,LOC100061170,LOC100058148,LOC100061066,LOC100060999,LOC100058104,LOC100060811,UNC5D,KCNU1,LOC100058019
27	8937157	8962230	25074	1	1	
27	9184219	9468020	283802	1	1	UNC5D
27	12128462	12220092	91631	1	1	NRG1
27	13011581	13107731	96151	1	3	
27	13198799	13303733	104935	3	1	
27	17404963	17660789	255827	3	1	LOC100049909
27	17540371	17601828	61458	1	1	LOC100049909
27	17601828	17660789	58962	3	6	LOC100049909
27	17779057	20825676	3046620	3	1	MTUS1,PDGFRL,LOC100050240,MTMR7,VPS37A,LOC100050103,LOC100053506,LOC100053360,LOC100050037,MSR1,TUSC3,MIR383,LOC100049909
27	17980506	18067175	86670	1	2	LOC100049909
27	19124767	19196320	71554	1	2	
27	21219902	22182863	962962	3	1	
27	22858481	23127387	268907	3	1	LOC100055580,LOC100630297
27	24568187	25735477	1167291	3	1	ENPP6,IRF2,LOC100630717,CASP3,CCDC111,LOC100058020,ACSL1,LOC100057785,LOC100050837,LOC100050762,LOC100057615,SNX25,LRP2BP,LOC100630630,UFSP2,LOC100057211,LOC100057171,LOC100057090,SORBS2
27	26062882	30665322	4602441	3	1	ODZ1,DCTD,WWC2,LOC100058927,LOC100058890,LOC100058784,LOC100051208,LOC100058620,LOC100051135
27	30131190	30473284	342095	1	1	
27	30665322	30769039	103718	1	2	
27	31283732	35910189	4626458	3	1	CSMD1,ANGPT2,LOC100064063,LOC100063996,XKR5,LOC100629676,LOC100629656,LOC100629644,LOC100629622,LOC100629602,DEFA17,LOC100629584,DEFA26,LOC100629563,DEFA35L,DEFA5,DEFA12,DEFA1,DEFA11,DEFA31L,LOC100629514,BD-1,LOC100629494,LOC100629479,LOC100629456,LOC100629426,LOC100629355,LOC100629408,LOC100629331,LOC100063044,LOC100146734,LOC100062056,LOC100629371,LOC100051492,LOC100061593,WDR17,LOC100051422,LOC100061344,LOC100629176,LOC100060890,NEIL3,LOC100060636
27	35076515	35110927	34413	1	1	
27	35866913	35910189	43277	1	1	CSMD1
27	36494190	39932455	3438266	3	1	LOC100629636,EQUCABV1R-PS932,EQUCABV1R-PS931,FBXO25,LOC100066006,LOC100065976,LOC100065419,LOC100065394,LOC100065372,LOC100065281,MYOM2,CSMD1
27	38122497	38232991	110495	1	1	
28	1087329	1342640	255312	3	1	TRHDE
28	2930576	3510645	580070	3	1	LOC100058105,LOC100058062,KCNC2
28	4603066	5109328	506263	3	1	E2F7,LOC100050307,ZDHHC17
28	6098105	6193273	95169	3	1	
28	6435277	6435555	279	1	1	
28	7381996	9664431	2282436	3	1	LOC100050763,LOC100061266,PPFIA2,ACSS3,LOC100050683,LOC100060703,LOC100050603,PTPRQ,OTOGL,LOC100630781,PPP1R12A
28	10399892	13134454	2734563	3	1	MGAT4C,LOC100630896,LOC100062225,RASSF9,ALX1,LRRIQ1,LOC100146893,LOC100050913
28	10879611	11047216	167606	3	1	
28	13426448	13487430	60983	1	1	
28	17853343	18335085	481743	3	1	EEA1,PLEKHG7,LOC100064227,LOC100629261
28	19163076	23013418	3850343	3	1	LOC100066155,TRNAW-CCA,TRNAD-GUC,MIR135A-2,LOC100629835,NEDD1,LOC100052372,CDK17,ELK3,LOC100065628,TRNAD-GUC,LTA4H,LOC100052193,LOC100065538,CCDC38,LOC100630068,LOC100065441,USP44,METAP2,MIR331,VEZT,FGD6,NR2C1,LOC100065070,LOC100064943,CCDC41,LOC100064706,LOC100064582
28	23426940	25004783	1577844	3	1	ANO4,GAS2L3,NR1H4,SLC17A8,LOC100052713,LOC100066435,ACTR6,UHRF1BP1L,ANKS1B
28	25786535	33955845	8169311	3	1	LOC100054570,LOC100054528,FOXRED2,LOC100069490,MYH9,LOC100069464,LOC100069448,LOC100069374,LOC100069362,LOC100069341,LOC100069335,MB,LOC100054386,MCM5,LOC100069058,TOM1,HMGXB4,LOC100069031,LARGE,TIMP3,SYN3,LOC100068847,BPIL2,LOC100054153,LOC100068816,PRDM4,LOC100054049,BTBD11,CRY1,MTERFD3,LOC100053907,RIC8B,RFX4,POLR3B,LOC100068464,CKAP4,LOC100053752,APLP2,LOC100068154,ALDH1L2,LOC100053656,SLC41A2,CHST11,TXNRD1,NFYB,LOC100067735,LOC100053507,TDG,LOC100630833,GRP94,LOC100067545,STAB2,LOC100146286,PAH,IGF1,LOC100053268,LOC100053215,NUP37,LOC100053117,LOC100067057
28	31181598	31210048	28451	1	1	

Chrom	Start	End	Size	Cn	Samples (n)	Genes
28	34507026	35756074	1249049	3	1	LOC100070178,GTPBP1,LOC100055255,LOC100070131,LOC100070115,LOC100629284,DMC1,DDX17,LOC100070048,LOC100070031,LOC100055155,TMEM184B,PLA2G6,LOC100069993,LOC100069977,PICK1,SOX10,LOC100054935,LOC100069945,MICALL1,EIF3L,LOC100054842,LOC100630844,GCAT,LOC100069872,TRIOBP,LOC100054799,LOC100069841,LOC100630816,SH3BP1,LOC100147078,LOC100069787,LOC100054751,CARD10,LOC100069766,ELFN2,CYTH4,LOC100054701,LOC100069706,LOC100069688
28	36188836	39644623	3455788	3	1	SCUBE1,LOC100071120,LOC100071110,LOC100071100,LOC100056216,PACSIN2,LOC100071070,LOC100071058,LOC100071050,POLDIP3,LOC100071022,LOC100071013,LOC100071003,TCF20,LOC100070990,LOC100146596,LOC100147480,LOC100070962,LOC100056087,LOC100070954,CYP2D50,LOC100146391,LOC100070905,LOC100070895,LOC100147382,LOC100147660,LOC100629651,LOC100070817,LOC100056050,LOC100056008,NAGA,LOC100070785,SEPT3,CENPM,TNFRSF13C,LOC100070715,MIR33A,SREBF2,LOC100055970,MEI1,LOC100629531,LOC100070695,XRCC6,LOC100070677,LOC100055891,LOC100070658,LOC100055847,LOC100055803,LOC100055758,LOC100055713,LOC100055668,ZC3H7B,RANGAP1,CHADL,LOC100070590,EP300,LOC100055581,LOC100629166,XPNPEP3,LOC100055503,LOC100070515,LOC100070500,MCHR1,LOC100070476,LOC100070463,LOC100070427,TNRC6B,LOC100070395,LOC100055418,ENTHD1,LOC100070365,LOC100070342,LOC100070334,LOC100055376,MGAT3,LOC100070300
28	40451212	41254009	802798	3	1	LOC100052016,LOC100051710,NUP50,LOC100629899,PHF21B,LOC100629845,LOC100051354,LOC100051281,LOC100146571
28	40686621	40749346	62726	1	1	
28	42690853	42757848	66996	3	1	TBC1D22A
28	43329623	44244661	915039	3	1	LOC100630223
28	44669398	44813253	143856	3	1	
28	45186697	46054133	867437	3	1	LOC100055523,LOC100055480,CPT1B,LOC100630606,LOC100056829,LOC100055399,LOC100146854,LOC100056712,NCAPH2,LMF2,LOC100056580,LOC100629546,SBF1,PPP6R2,LOC100056442,PLXNB2,LOC100056278,LOC100056239,LOC100146361,LOC100056192,SELO,TRABD,LOC100056108,MOV10L1,LOC100055870,LOC100146652,ZBED4,BRD1
29	477117	631698	154582	3	10	LOC100070840,LOC100070867,LOC100070887
29	19660276	19754993	94718	1	1	
29	22816885	22829932	13048	3	1	
30	2147049	2370660	223612	3	1	
30	7327648	7347808	20161	3	1	LOC100058218
30	16789975	16860626	70652	1	1	
30	17461773	17667911	206139	1	1	
30	23187556	23284405	96850	1	2	
30	28307647	28354841	47195	3	5	PKP1,LOC100063337
31	4829031	4884348	55318	1	1	QKI
31	8225583	8227054	1472	1,3	1,1	MLLT4
31	12046717	12153299	106583	1	1	LOC100061268
31	12602385	12778909	176525	4	1	
31	14723380	14741273	17894	1	1	SYNE1
31	20741186	20797366	56181	1	1	
