# Supplementary material for: Analysis of copy number variants by three detection algorithms and their association with body size in horses
Source: BMC Genomics. 2013 Jul 18;14:487. doi: 10.1186/1471-2164-14-487 (PMC3720552; doi:10.1186/1471-2164-14-487)
Supplement: Additional file 3 — CNVs detected by QuantiSNP. The table summarises start and end positions of detected CNVs, their size, copy number, number of samples and genes located in CNV regions. Text in PDF format. [file 1471-2164-14-487-S3.pdf]

Chrom	Start	End	Size	Cn	Samples (n)	Genes
01	180799	803824	623026	1	1	LOC100066869,LOC100066767,LOC100067022,LOC100066719,LOC100066787,LOC100066579,LOC100066671,LOC100629322,LOC100066391,LOC100066851,LOC100066748,KNDC1,LOC100146619,LOC100067042,LOC100066808,LOC100050642,LOC100066894,LOC100066831,LOC100066555
01	180799	1995023	1814225	3	1	LOC100066869,LOC100146618,LOC100051460,LOC100066767,LOC100067022,LOC100066719,LOC100066787,LOC100066579,LOC100066671,LOC100629322,LOC100066391,LOC100066851,LOC100051673,LOC100051737,LOC100066748,KNDC1,LOC100051318,LOC100146619,LOC100067042,INPP5A,STK32C,JAKMIP3,LOC100066808,LOC100630642,LOC100050642,LOC100066894,LOC100066831,LOC100066555,LOC100050953
01	180799	2708943	2528145	3,4	1,1	LOC100066869,LOC100146618,LOC100051460,LOC100066767,LOC100067022,LOC100066719,LOC100066787,LOC100066579,LOC100066671,LOC100629322,LOC100066391,LOC100066851,LOC100051673,LOC100051737,LOC100066748,KNDC1,LOC100051318,LOC100146619,LOC100067042,TCERG1L,INPP5A,STK32C,JAKMIP3,LOC100066808,LOC100630642,LOC100050642,LOC100066894,LOC100066831,LOC100066555,LOC100050953
01	292214	1995023	1702810	3	1	LOC100146618,LOC100051460,LOC100066767,LOC100066719,LOC100066787,LOC100066579,LOC100066671,LOC100629322,LOC100066391,LOC100051673,LOC100051737,LOC100066748,KNDC1,LOC100051318,LOC100146619,INPP5A,STK32C,JAKMIP3,LOC100066808,LOC100630642,LOC100050642,LOC100066831,LOC100066555,LOC100050953
01	498373	1995023	1496651	3	1	LOC100146618,LOC100051460,LOC100066579,LOC100051673,LOC100051737,KNDC1,LOC100051318,LOC100146619,INPP5A,STK32C,JAKMIP3,LOC100630642,LOC100050642,LOC100066555,LOC100050953
01	846350	2680226	1833877	3	1	LOC100146618,LOC100051460,LOC100051673,LOC100051737,LOC100051318,TCERG1L,INPP5A,STK32C,JAKMIP3,LOC100630642,LOC100050953
01	1005122	1610099	604978	3	1	LOC100146618,LOC100051318,INPP5A,STK32C,LOC100050953
01	15653901	15820581	166681	1	2	LOC100067810,LOC100058872
01	15792557	15820581	28025	1	3	LOC100067810
01	27766435	28912593	1146159	1	1	LOC100070213,LOC100070086,LOC100060342,LOC100070241,LOC100060168,LOC100070266,LOC100060046,LOC100070056,LOC100070286,LOC100060309,LOC100060081,PPRC1,LOC100070099,LOC100060275,LOC100070070,BTRC,MGEA5,POLL,GBF1,NOLC1,KCNIP2,LOC100060384,LZTS2
01	30735779	30753444	17666	1	3	HPSE2
01	44181705	46783126	2601422	1	1	LOC100072003,LOC100072070,PCDH15,LOC100072051
01	49442370	49760307	317938	1	1	ANK3,CCDC6
01	53303287	54337293	1034007	1	1	LOC100072519,LOC100072507
01	53701626	56025202	2323577	1	1	LRRTM3,CTNNA3
01	58756894	59527742	770849	3	1	ADAMTS14,LOC100072751,CDH23,UNC5B,SGPL1,LOC100072768,LOC100072758,LOC100630411
01	58756894	59563056	806163	4	1	ADAMTS14,LOC100072751,CDH23,UNC5B,SGPL1,LOC100072768,LOC100072758,LOC100630411
01	67035016	67562066	527051	4	1	COG2,LOC100065526,LOC100630293
01	71887891	73575439	1687549	1	1	CHRM3,LOC100629229
01	79592692	83324830	3732139	3	1	LOC100062632,LOC100061969,MARCH8,LOC100629629,GNPAT,LOC100051390,LOC100063422,LOC100629985,LOC100063099,ANXA8L1,LOC100062521,CAPN9,LOC100051674,LOC100629963,LOC100061368,FRMPD2,LOC100052105,GDF2,LOC100063000,LOC100063686,LOC100062005,LOC100051860,LOC100051600,OGDHL,LOC100061933,LOC100052165,LOC100063157,TTC13,ERCC6,LOC100061806,LOC100061869,LOC100063878,LOC100060950,ALOX5,GDF10,TRIM67,ARHGAP22,LOC100063727,LOC100051461,LOC100061899,MAPK8,LOC100051738,NCOA4,LOC100060794,LOC100629874,LOC100062210
01	83318031	84342296	1024266	3	1	LOC100052406,BMPR1A,SYT15,LOC100064114,GRID1,LOC100052349,LOC100629593,LOC100064336,WAPAL,LOC100630294,LOC100064139,LOC100052462,MIR346
01	90222341	91266386	1044046	1	1	LOC100067399,ARNT2,LOC100067603,LOC100053052,LOC100067959,LOC100067670,LOC100067745,LOC100067811,LOC100067647
01	93278107	94644957	1366851	3	1	LOC100069456,ABHD2,LOC100069692,FANCI,ACAN,MFGE8,LOC100069086,SEMA4B,LOC100053729,LOC100069672,DET1,MIR1179,MIR7,LOC100069794,LOC100053929,LOC100069760,LOC100069725,LOC100069681,POLG,ZNF710,LOC100629729,ANPEP,WDR93,LOC100053774
01	95947839	96018805	70967	1	1	
01	111349920	111678419	328500	1	1	TRNAE-UUC,LOC100062319
01	111524212	111648330	124119	1	1	
01	116692029	116796279	104251	1	1	LOC100060833
01	117768737	118220212	451476	1	1	LOC100061327,HMG20A,LINGO1
01	118257321	119959000	1701680	4	1	LOC100062176,ULK3,NEIL1,CCDC33,MAN2C1,CSK,LOC100061580,TBC1D21,ARID3B,SIN3A,STOML1,LOC100051100,SNX33,LOXL1,LOC100051391,CSPG4,LOC100061807,LOC100062705,MPI,LOC100061503,LOC100062244,LOC100061743,CLK3,ISLR,LOC100061677,LOC100051985,CYP11A1,LOC100062111,LOC100051799,CYP1A1,LOC100062522,LOC100062043,EDC3,NPTN,LOC100052046,LOC100051030,PML,LOC100062212,LOC100051247,LOC100051534,LOC100062559,LOC100051321
01	135802169	136208689	406521	1	1	
01	136563078	136816131	253054	1	1	UNC13C
01	136563078	136887927	324850	4	1	UNC13C
01	151343566	152621838	1278273	1	1	LOC100057596,LOC100071695,LOC100071713,LOC100071684
01	155323247	155656642	333396	1	1	LOC100072099,LOC100058220,LOC100146794,LOC100072083,LOC100072126,LOC100072136,LOC100072146,LOC100072141,LOC100072149,LOC100058263,LOC100147374,LOC100072107,LOC100072074,LOC100072089,LOC100072129,LOC100072110,LOC100072120,LOC100072092,LOC100072117,LOC100072079,LOC100072112,LOC100058312
01	155487276	155546142	58867	0	1	LOC100072117,LOC100072112,LOC100058312

Chrom	Start	End	Size	Cn	Samples (n)	Genes
01	155487276	155546701	59426	1	10	LOC100072117,LOC100072112,LOC100058312
01	155487276	155593582	106307	0,1,3	1,4,1	LOC100072126,LOC100072129,LOC100072120,LOC100072117,LOC100072112,LOC100058312
01	155487276	155652475	165200	0,1	2,27	LOC100072126,LOC100072136,LOC100072146,LOC100072141,LOC100072149,LOC100072129,LOC100072120,LOC100072117,LOC100072112,LOC100058312
01	155487276	155656642	169367	0,1	25,6	LOC100072126,LOC100072136,LOC100072146,LOC100072141,LOC100072149,LOC100072129,LOC100072120,LOC100072117,LOC100072112,LOC100058312
01	155510180	155656642	146463	1	2	LOC100072126,LOC100072136,LOC100072146,LOC100072141,LOC100072149,LOC100072129,LOC100072120,LOC100072117,LOC100072112
01	155546142	155546701	560	1	1	
01	155546142	155656642	110501	3	1	LOC100072126,LOC100072136,LOC100072146,LOC100072141,LOC100072149,LOC100072129,LOC100072120
01	155759601	156657881	898281	1	1	LOC100072296,LOC100072258,LOC100072414,LOC100072179,LOC100072447,LOC100072426,LOC100072176,LOC100072215,LOC100072251,LOC100058354,LOC100072237,LOC100072429,LOC100072368,LOC100072386,LOC100072192,LOC100072351,LOC100072271,LOC100072432,LOC100072275,LOC100072202,LOC100072265,LOC100072279,LOC100072306,LOC100072261,LOC100072184,LOC100072398,LOC100072342,LOC100072288,LOC100072317,LOC100072300,LOC100072380,LOC100072227,LOC100072195,LOC100072365,LOC100072443,LOC100058435,LOC100072402,LOC100072282,LOC100072290,LOC100072423,LOC100072320,LOC100072338,LOC100072221,LOC100072392,LOC100072354,LOC100072404,LOC100072172,LOC100146987,LOC100072206,LOC100072211,LOC100072325,LOC100058393,LOC100072375,LOC100072345,LOC100072408,LOC100072254,LOC100072330,LOC100072335,LOC100072245
01	155795029	156012981	217953	1	3	LOC100072179,LOC100072215,LOC100058354,LOC100072192,LOC100072202,LOC100072184,LOC100072227,LOC100072195,LOC100072221,LOC100072206,LOC100072211,LOC100058393
01	155795029	156358692	563664	0	1	LOC100072296,LOC100072258,LOC100072179,LOC100072215,LOC100072251,LOC100058354,LOC100072237,LOC100072192,LOC100072271,LOC100072275,LOC100072202,LOC100072265,LOC100072279,LOC100072306,LOC100072261,LOC100072184,LOC100072342,LOC100072288,LOC100072317,LOC100072300,LOC100072227,LOC100072195,LOC100072282,LOC100072290,LOC100072320,LOC100072338,LOC100072221,LOC100146987,LOC100072206,LOC100072211,LOC100072325,LOC100058393,LOC100072345,LOC100072254,LOC100072330,LOC100072335,LOC100072245
01	155795029	156449080	654052	1	1	LOC100072296,LOC100072258,LOC100072179,LOC100072215,LOC100072251,LOC100058354,LOC100072237,LOC100072368,LOC100072192,LOC100072351,LOC100072271,LOC100072275,LOC100072202,LOC100072265,LOC100072279,LOC100072306,LOC100072261,LOC100072184,LOC100072342,LOC100072288,LOC100072317,LOC100072300,LOC100072380,LOC100072227,LOC100072195,LOC100072365,LOC100072282,LOC100072290,LOC100072320,LOC100072338,LOC100072221,LOC100072354,LOC100146987,LOC100072206,LOC100072211,LOC100072325,LOC100058393,LOC100072375,LOC100072345,LOC100072254,LOC100072330,LOC100072335,LOC100072245
01	155795029	156491374	696346	1	1	LOC100072296,LOC100072258,LOC100072179,LOC100072215,LOC100072251,LOC100058354,LOC100072237,LOC100072368,LOC100072386,LOC100072192,LOC100072351,LOC100072271,LOC100072275,LOC100072202,LOC100072265,LOC100072279,LOC100072306,LOC100072261,LOC100072184,LOC100072398,LOC100072342,LOC100072288,LOC100072317,LOC100072300,LOC100072380,LOC100072227,LOC100072195,LOC100072365,LOC100072282,LOC100072290,LOC100072320,LOC100072338,LOC100072221,LOC100072392,LOC100072354,LOC100146987,LOC100072206,LOC100072211,LOC100072325,LOC100058393,LOC100072375,LOC100072345,LOC100072254,LOC100072330,LOC100072335,LOC100072245
01	155795029	156657881	862853	1	14	LOC100072296,LOC100072258,LOC100072414,LOC100072179,LOC100072447,LOC100072426,LOC100072215,LOC100072251,LOC100058354,LOC100072237,LOC100072429,LOC100072368,LOC100072386,LOC100072192,LOC100072351,LOC100072271,LOC100072432,LOC100072275,LOC100072202,LOC100072265,LOC100072279,LOC100072306,LOC100072261,LOC100072184,LOC100072398,LOC100072342,LOC100072288,LOC100072317,LOC100072300,LOC100072380,LOC100072227,LOC100072195,LOC100072365,LOC100072443,LOC100058435,LOC100072402,LOC100072282,LOC100072290,LOC100072423,LOC100072320,LOC100072338,LOC100072221,LOC100072392,LOC100072354,LOC100072404,LOC100146987,LOC100072206,LOC100072211,LOC100072325,LOC100058393,LOC100072375,LOC100072345,LOC100072408,LOC100072254,LOC100072330,LOC100072335,LOC100072245
01	155795652	156657881	862230	1	1	LOC100072296,LOC100072258,LOC100072414,LOC100072179,LOC100072447,LOC100072426,LOC100072215,LOC100072251,LOC100058354,LOC100072237,LOC100072429,LOC100072368,LOC100072386,LOC100072192,LOC100072351,LOC100072271,LOC100072432,LOC100072275,LOC100072202,LOC100072265,LOC100072279,LOC100072306,LOC100072261,LOC100072184,LOC100072398,LOC100072342,LOC100072288,LOC100072317,LOC100072300,LOC100072380,LOC100072227,LOC100072195,LOC100072365,LOC100072443,LOC100058435,LOC100072402,LOC100072282,LOC100072290,LOC100072423,LOC100072320,LOC100072338,LOC100072221,LOC100072392,LOC100072354,LOC100072404,LOC100146987,LOC100072206,LOC100072211,LOC100072325,LOC100058393,LOC100072375,LOC100072345,LOC100072408,LOC100072254,LOC100072330,LOC100072335,LOC100072245
01	155954895	156012981	58087	1	1	LOC100072227,LOC100072221
01	155954895	156358692	403798	1	2	LOC100072296,LOC100072258,LOC100072251,LOC100072237,LOC100072271,LOC100072275,LOC100072265,LOC100072279,LOC100072306,LOC100072261,LOC100072342,LOC100072288,LOC100072317,LOC100072300,LOC100072227,LOC100072282,LOC100072290,LOC100072320,LOC100072338,LOC100072221,LOC100146987,LOC100072325,LOC100072345,LOC100072254,LOC100072330,LOC100072335,LOC100072245
01	155954895	156529837	574943	1	1	LOC100072296,LOC100072258,LOC100072251,LOC100072237,LOC100072368,LOC100072386,LOC100072351,LOC100072271,LOC100072275,LOC100072265,LOC100072279,LOC100072306,LOC100072261,LOC100072398,LOC100072342,LOC100072288,LOC100072317,LOC100072300,LOC100072380,LOC100072227,LOC100072365,LOC100072402,LOC100072282,LOC100072290,LOC100072320,LOC100072338,LOC100072221,LOC100072392,LOC100072354,LOC100072404,LOC100146987,LOC100072325,LOC100072375,LOC100072345,LOC100072408,LOC100072254,LOC100072330,LOC100072335,LOC100072245

Chrom	Start	End	Size	Cn	Samples (n)	Genes
01	155954895	156657881	702987	1	6	LOC100072296,LOC100072258,LOC100072414,LOC100072447,LOC100072426,LOC100072251,LOC100072237,LOC100072429,LOC100072368,LOC100072386,LOC100072351,LOC100072271,LOC100072432,LOC100072275,LOC100072265,LOC100072279,LOC100072306,LOC100072261,LOC100072398,LOC100072342,LOC100072288,LOC100072317,LOC100072300,LOC100072380,LOC100072227,LOC100072365,LOC100072443,LOC100058435,LOC100072402,LOC100072282,LOC100072290,LOC100072423,LOC100072320,LOC100072338,LOC100072221,LOC100072392,LOC100072354,LOC100072404,LOC100146987,LOC100072325,LOC100072375,LOC100072345,LOC100072408,LOC100072254,LOC100072330,LOC100072335,LOC100072245
01	156002161	156462763	460603	1	1	LOC100072296,LOC100072258,LOC100072251,LOC100072237,LOC100072368,LOC100072386,LOC100072351,LOC100072271,LOC100072275,LOC100072265,LOC100072279,LOC100072306,LOC100072261,LOC100072342,LOC100072288,LOC100072317,LOC100072300,LOC100072380,LOC100072365,LOC100072282,LOC100072290,LOC100072320,LOC100072338,LOC100072354,LOC100146987,LOC100072325,LOC100072375,LOC100072345,LOC100072254,LOC100072330,LOC100072335,LOC100072245
01	156125915	156358067	232153	0	1	LOC100072296,LOC100072306,LOC100072342,LOC100072288,LOC100072317,LOC100072300,LOC100072290,LOC100072320,LOC100072338,LOC100146987,LOC100072325,LOC100072345,LOC100072330,LOC100072335
01	156125915	156358692	232778	0	4	LOC100072296,LOC100072306,LOC100072342,LOC100072288,LOC100072317,LOC100072300,LOC100072290,LOC100072320,LOC100072338,LOC100146987,LOC100072325,LOC100072345,LOC100072330,LOC100072335
01	156125915	156425245	299331	1	1	LOC100072296,LOC100072368,LOC100072351,LOC100072306,LOC100072342,LOC100072288,LOC100072317,LOC100072300,LOC100072365,LOC100072290,LOC100072320,LOC100072338,LOC100072354,LOC100146987,LOC100072325,LOC100072345,LOC100072330,LOC100072335
01	156125915	156449080	323166	1	3	LOC100072296,LOC100072368,LOC100072351,LOC100072306,LOC100072342,LOC100072288,LOC100072317,LOC100072300,LOC100072380,LOC100072365,LOC100072290,LOC100072320,LOC100072338,LOC100072354,LOC100146987,LOC100072325,LOC100072375,LOC100072345,LOC100072330,LOC100072335
01	156125915	156462763	336849	1	4	LOC100072296,LOC100072368,LOC100072386,LOC100072351,LOC100072306,LOC100072342,LOC100072288,LOC100072317,LOC100072300,LOC100072380,LOC100072365,LOC100072290,LOC100072320,LOC100072338,LOC100072354,LOC100146987,LOC100072325,LOC100072375,LOC100072345,LOC100072330,LOC100072335
01	156125915	156491374	365460	0,1	1,6	LOC100072296,LOC100072368,LOC100072386,LOC100072351,LOC100072306,LOC100072398,LOC100072342,LOC100072288,LOC100072317,LOC100072300,LOC100072380,LOC100072365,LOC100072290,LOC100072320,LOC100072338,LOC100072392,LOC100072354,LOC100146987,LOC100072325,LOC100072375,LOC100072345,LOC100072330,LOC100072335
01	156135693	156358692	223000	0	1	LOC100072296,LOC100072306,LOC100072342,LOC100072288,LOC100072317,LOC100072300,LOC100072290,LOC100072320,LOC100072338,LOC100146987,LOC100072325,LOC100072345,LOC100072330,LOC100072335
01	156135693	156462763	327071	1	2	LOC100072296,LOC100072368,LOC100072386,LOC100072351,LOC100072306,LOC100072342,LOC100072288,LOC100072317,LOC100072300,LOC100072380,LOC100072365,LOC100072290,LOC100072320,LOC100072338,LOC100072354,LOC100146987,LOC100072325,LOC100072375,LOC100072345,LOC100072330,LOC100072335
01	156358067	156491374	133308	1	3	LOC100072368,LOC100072386,LOC100072351,LOC100072398,LOC100072380,LOC100072365,LOC100072392,LOC100072354,LOC100072375
01	156425245	156491374	66130	0	3	LOC100072386,LOC100072398,LOC100072380,LOC100072392,LOC100072375
01	156425245	156529837	104593	0	1	LOC100072386,LOC100072398,LOC100072380,LOC100072402,LOC100072392,LOC100072404,LOC100072375,LOC100072408
01	156425245	156646801	221557	1	1	LOC100072414,LOC100072447,LOC100072426,LOC100072429,LOC100072386,LOC100072432,LOC100072398,LOC100072380,LOC100072443,LOC100072402,LOC100072423,LOC100072392,LOC100072404,LOC100072375,LOC100072408
01	156425245	156657881	232637	0	1	LOC100072414,LOC100072447,LOC100072426,LOC100072429,LOC100072386,LOC100072432,LOC100072398,LOC100072380,LOC100072443,LOC100058435,LOC100072402,LOC100072423,LOC100072392,LOC100072404,LOC100072375,LOC100072408
01	156449080	156491374	42295	0	2	LOC100072386,LOC100072398,LOC100072392
01	156449080	156657881	208802	0	2	LOC100072414,LOC100072447,LOC100072426,LOC100072429,LOC100072386,LOC100072432,LOC100072398,LOC100072443,LOC100058435,LOC100072402,LOC100072423,LOC100072392,LOC100072404,LOC100072408
01	156449080	156870455	421376	1	11	LOC100072414,LOC100072447,LOC100072426,LOC100072429,LOC100072386,LOC100072432,LOC100072464,LOC100072493,LOC100072499,LOC100072398,LOC100072469,LOC100629829,LOC100072443,LOC100058435,LOC100072495,LOC100072402,LOC100072423,LOC100072392,LOC100072404,LOC100072457,LOC100072479,LOC100072473,LOC100072408,LOC100072489,LOC100072477,LOC100072452
01	156462763	156818876	356114	1	1	LOC100072414,LOC100072447,LOC100072426,LOC100072429,LOC100072386,LOC100072432,LOC100072464,LOC100072398,LOC100072469,LOC100629829,LOC100072443,LOC100058435,LOC100072402,LOC100072423,LOC100072392,LOC100072404,LOC100072457,LOC100072479,LOC100072473,LOC100072408,LOC100072489,LOC100072477,LOC100072452
01	156529837	156657881	128045	1	1	LOC100072414,LOC100072447,LOC100072426,LOC100072429,LOC100072432,LOC100072443,LOC100058435,LOC100072423
01	156529837	156870455	340619	1	5	LOC100072414,LOC100072447,LOC100072426,LOC100072429,LOC100072432,LOC100072464,LOC100072493,LOC100072499,LOC100072469,LOC100629829,LOC100072443,LOC100058435,LOC100072495,LOC100072423,LOC100072457,LOC100072479,LOC100072473,LOC100072489,LOC100072477,LOC100072452
01	156599954	156657881	57928	1	1	LOC100072447,LOC100072429,LOC100072432,LOC100072443,LOC100058435
01	156633245	156870455	237211	1	2	LOC100072447,LOC100072464,LOC100072493,LOC100072499,LOC100072469,LOC100629829,LOC100072443,LOC100058435,LOC100072495,LOC100072457,LOC100072479,LOC100072473,LOC100072489,LOC100072477,LOC100072452
01	156681241	156870455	189215	1	2	LOC100072464,LOC100072493,LOC100072499,LOC100072469,LOC100629829,LOC100072495,LOC100072457,LOC100072479,LOC100072473,LOC100072489,LOC100072477
01	165267754	166217268	949515	1	1	LOC100054183,LOC100049876
01	165267754	166232546	964793	1	1	LOC100054183,LOC100049876
01	165267754	166724938	1457185	1	1	LOC100054183,LOC100049876,LOC100054736,LOC100146246

Chrom	Start	End	Size	Cn	Samples (n)	Genes
01	165794587	165970661	176075	1	1	
01	165794587	166232546	437960	1	1	
01	168355661	168547792	192132	1	1	LOC100050137,STRN3,COCH,HECTD1
01	174036149	174073247	37099	1	1	
01	174036149	174130028	93880	1	1	TTC6
01	175713115	175785528	72414	1	1	
01	177538112	178573079	1034968	1	1	
01	178337817	178844926	507110	1	2	LOC100062668,LOC100629700
01	178553782	178573079	19298	1	6	
01	178553782	178798394	244613	1	1	LOC100062668,LOC100629700
01	178553782	178815370	261589	1	1	LOC100062668,LOC100629700
01	178798269	178918130	119862	3	1	LOC100062668
01	178798269	179550475	752207	3	1	LOC100062807,LOC100062668,LOC100062944
01	178918130	179550475	632346	3	1	LOC100062807,LOC100062944
01	179087860	179550475	462616	3	1	LOC100062807,LOC100062944
01	180772718	181266487	493770	1	1	LOC100064278
01	180772718	181447348	674631	1	1	LOC100064278,MDGA2
01	180772718	182014222	1241505	1	1	LOC100064278,MDGA2
02	4712044	6816185	2104142	3	1	LOC100061716,LOC100060596,LOC100629783,ZYG11A,LOC100060768,LOC100061061,LOC100060994,LRP8,LOC100050223,LOC100061753,ZYG11B,LOC100060843,LOC100060530,LOC100060633,YIPF1,LOC100061450,DIO1,LOC100060426,LOC100060883,LOC100060494,LOC100061034,LRRC42,LOC100061338,DHCR24,LOC100060319,LOC100060394,LOC100050590,TMEM48,PARS2,LOC100060561,LOC100050090,LOC100060286,SLC1A7,ACOT11,LOC100050156,PODN
02	9531884	12293422	2761539	3	1	AGBL4,LOC100051622,FAAH,LOC100064407,LOC100064466,LOC100063364,LOC100063738,LOC100064092,LOC100630182,LOC100630028,LOC100064521,LOC100147237,LOC100064782,LOC100063464,SLC5A9,LOC100064176,LOC100051481,LOC100629887,LOC100064057,LOC100063826,LOC100630164,LOC100064020,STIL,LOC100051878,LOC100064146,LOC100051817,LOC100052061,LOC100147048,LOC100064287,LOC100063993,LOC100630232,LOC100052003,LOC100051696,LRRC41,LOC100630320,LOC100051552
02	10124702	12513524	2388823	3	1	AGBL4,LOC100064866,LOC100051622,FAAH,LOC100064407,LOC100064466,LOC100063364,LOC100063738,LOC100064092,LOC100630182,LOC100630028,LOC100064521,LOC100147237,LOC100064782,LOC100052122,LOC100063464,SLC5A9,LOC100064176,LOC100051481,LOC100629887,LOC100064057,LOC100063826,LOC100630164,LOC100064020,STIL,LOC100051878,LOC100064146,LOC100051817,LOC100052061,LOC100147048,LOC100064287,LOC100063993,LOC100630232,LOC100052003,LOC100051696,LRRC41,LOC100630320,LOC100065035,LOC100051552
02	15112913	15284362	171450	0	8	LOC100629289,LOC100066795,LOC100629267,LOC100053497,LOC100066777
02	23990961	26123601	2132641	4	1	LOC100070474,LOC100070319,LOC100070149,LOC100055884,RNF19B,LOC100630406,LOC100055706,LOC100070112,LOC100070175,ZBTB8A,BSDC1,LOC100070183,LOC100055923,LOC100070339,LOC100070402,SPOCD1,COL16A1,SERINC2,LOC100056122,LOC100056331,LOC100070507,YARS,BAI2,LOC100056288,TMEM39B,LOC100070162,LOC100070089,LOC100146454,LOC100070525,LOC100070281,PUM1,LOC100070490,LOC100070269,TXLNA,TINAGL1,LOC100070377,LCK,HCRTR1,LOC100070307,LOC100070330,EIF3I,LOC100055965,LOC100056375,FABP3,LOC100056044,ZBTB8B
02	28585912	31193015	2607104	3	1	PAFAH2,LOC100057526,LOC100071521,LOC100071501,LOC100071263,LOC100057451,LOC100057411,LOC100071195,LOC100070978,LIN28A,RPS6KA1,LOC100071031,ZNF683,DHDDS,GPATCH3,LOC100071320,GRHL3,SLC9A1,LOC100057007,LOC100070986,LOC100071048,LOC100629964,LOC100071125,LOC100071135,CEP85,LOC100057288,TRIM63,LOC100071168,LOC100071257,LOC100071282,LOC100071373,LOC100071457,LOC100071487,LOC100057607,SRSF10,CNR2,IL22RA1,LOC100071559,LOC100057686,SH3BGRL3,MYOM3,LOC100057775,LOC100629738,LOC100057723,CNKSR1,NIPAL3,EXTL1,LOC100071467,LOC100630531,LOC100071309,LOC100071364,RUNX3,LOC100071356,LOC100071327,LOC100071216,LOC100071130,PIGV,LOC100070967,LOC100057082,SLC30A2,LOC100071485,LOC100070998,LOC100057488,FUCA1
02	35255192	37787790	2532599	4	1	DDI2,LOC100068854,KLHDC7A,LOC100053258,SDHB,LOC100050294,LOC100053349,LOC100052965,LOC100053694,LOC100054520,LOC100054283,EPHA2,LOC100054423,PADI6,PADI2,LOC100054472,LOC100052755,LOC100629268,LOC100050157,ARHGEF19,FBXO42,LOC100053896,LOC100050670,DNAJC16,ARHGEF10L,CASP9,SPEN,PLEKHM2,ATP13A2,PADI1,ZBTB17,PADI4,PADI3,LOC100050360,LOC100050514,KAZN,LOC100629431,LOC100053498,LOC100050750,LOC100054040,LOC100147498,LOC100053306,LOC100053742
02	38347002	40904075	2557074	3	1	LOC100051196,LOC100056965,CASZ1,LOC100055537,PRDM2,LOC100056123,LOC100055840,MTHFR,LOC100050902,PTCHD2,LOC100056730,EXOSC10,LOC100056045,MASP2,MFN2,TNFRSF8,CLCN6,LOC100056289,LOC100051269,LOC100057041,ANGPTL7,LOC100056805,LOC100051122,LOC100055924,LOC100055371,NPPA,LOC100055412,LOC100051052,MTOR,TARDBP,LOC100056376,LOC100056417,LOC100055454,VPS13D
02	38559501	39915694	1356194	3	1	LOC100055537,PRDM2,LOC100056123,LOC100055840,MTHFR,LOC100050902,LOC100056045,MFN2,TNFRSF8,CLCN6,LOC100056289,LOC100055924,LOC100055371,NPPA,LOC100055412,LOC100051052,LOC100055454,VPS13D
02	38942608	40904075	1961468	3	1	LOC100051196,LOC100056965,CASZ1,LOC100055537,LOC100056123,LOC100055840,MTHFR,LOC100050902,PTCHD2,LOC100056730,EXOSC10,LOC100056045,MASP2,MFN2,TNFRSF8,CLCN6,LOC100056289,LOC100051269,LOC100057041,ANGPTL7,LOC100056805,LOC100051122,LOC100055924,LOC100055371,NPPA,LOC100055412,LOC100051052,MTOR,TARDBP,LOC100056376,LOC100056417,LOC100055454,VPS13D

Chrom	Start	End	Size	Cn	Samples (n)	Genes
02	39529471	40904075	1374605	3	1	LOC100051196,LOC100056965,CASZ1,LOC100056123,LOC100055840,MTHFR,LOC100050902,PTCHD2,LOC100056730,EXOSC10,LOC100056045,MASP2,MFN2,TNFRSF8,CLCN6,LOC100056289,LOC100051269,LOC100057041,ANGPTL7,LOC100056805,LOC100051122,LOC100055924,NPPA,LOC100051052,MTOR,TARDBP,LOC100056376,LOC100056417
02	43728534	48779927	5051394	3	1	LOC100629449,CCDC27,LOC100054405,LOC100064361,DNAJC11,CDK11A,LOC100052479,LOC100064482,LOC100066364,LOC100066420,LOC100066195,LOC100065961,LOC100146191,LOC100059116,LOC100059160,LOC100059540,LOC100059424,NPHP4,LRRC47,CEP104,PRDM16,LOC100061240,LOC100061363,LOC100061283,LOC100064300,LOC100064240,LOC100065322,LOC100065291,LOC100630652,LOC100052308,LOC100060427,MMEL1,LOC100059575,TP73,ARHGEF16,MIR551A,TNFRSF14,PLEKHG5,C-SKI,LOC100147590,LOC100064452,LOC100064074,LOC100052422,LOC100052362,LOC100147497,RNF207,LOC100146710,LOC100066254,LOC100065860,LOC100147589,LOC100629488,SAMD11,MIR200B,KLHL17,MIR200A,LOC100066228,LOC100146604,LOC100066278,MIR429,LOC100066088,CPSF3L,LOC100147200,LOC100066167,MORN1,LOC100066142,LOC100061534,LOC100059643,LOC100066014,NOC2L,LOC100061495,TAS1R1,LOC100147495,LOC100060696,LOC100065915,LOC100147581,LOC100060185,LOC100060634,ZBTB48,NOL9,CAMTA1,LOC100059680,LOC100059082,MEGF6,LOC100065083,GNB1,LOC100064187,LOC100054501,LOC100146417,LOC100061432,CHD5
02	44252586	45099470	846885	4	1	NPHP4,LOC100052308,LOC100059575,LOC100147590,LOC100052362,RNF207,LOC100059643,LOC100059680,CHD5
02	44490205	45036187	545983	4	1	NPHP4,LOC100052362
02	44698998	45036187	337190	4	1	
02	46021900	48976680	2954781	3	1	LOC100629449,CCDC27,LOC100054405,LOC100064361,CDK11A,LOC100052479,LOC100064482,LOC100066364,LOC100066420,LOC100066195,LOC100065961,LOC100146191,LRRC47,CEP104,PRDM16,LOC100061240,LOC100061363,LOC100061283,LOC100064300,LOC100064240,LOC100065322,LOC100065291,LOC100630652,LOC100060427,MMEL1,TP73,ARHGEF16,MIR551A,TNFRSF14,C-SKI,LOC100064452,LOC100064074,LOC100146710,LOC100066254,LOC100065860,LOC100147589,LOC100629488,SAMD11,MIR200B,KLHL17,MIR200A,LOC100066228,LOC100146604,LOC100066278,MIR429,LOC100066088,CPSF3L,LOC100147200,LOC100066167,MORN1,LOC100066142,LOC100061534,LOC100066014,NOC2L,LOC100061495,LOC100147495,LOC100060696,LOC100065915,LOC100147581,LOC100060634,MEGF6,LOC100065083,GNB1,LOC100064187,LOC100054501,LOC100146417,LOC100061432
02	46081697	48976680	2894984	3	1	LOC100629449,CCDC27,LOC100054405,LOC100064361,CDK11A,LOC100052479,LOC100064482,LOC100066364,LOC100066420,LOC100066195,LOC100065961,LOC100146191,LRRC47,CEP104,PRDM16,LOC100061240,LOC100061363,LOC100061283,LOC100064300,LOC100064240,LOC100065322,LOC100065291,LOC100630652,LOC100060427,MMEL1,TP73,ARHGEF16,MIR551A,TNFRSF14,C-SKI,LOC100064452,LOC100064074,LOC100146710,LOC100066254,LOC100065860,LOC100147589,LOC100629488,SAMD11,MIR200B,KLHL17,MIR200A,LOC100066228,LOC100146604,LOC100066278,MIR429,LOC100066088,CPSF3L,LOC100147200,LOC100066167,MORN1,LOC100066142,LOC100061534,LOC100066014,NOC2L,LOC100061495,LOC100147495,LOC100060696,LOC100065915,LOC100147581,LOC100060634,MEGF6,LOC100065083,GNB1,LOC100064187,LOC100054501,LOC100146417,LOC100061432
02	46772377	50111576	3339200	3	1	LOC100629449,LOC100054405,LOC100064361,CDK11A,LOC100064482,LOC100066364,LOC100066420,LOC100066195,LOC100065961,LOC100146191,PRDM16,LOC100061240,LOC100061363,LOC100061283,LOC100064300,LOC100064240,LOC100065322,LOC100065291,MMEL1,TNFRSF14,C-SKI,LOC100064452,LOC100064074,LZTS1,LOC100146710,LOC100066254,LOC100065860,LPL,LOC100147589,LOC100629488,SAMD11,MIR200B,KLHL17,MIR200A,LOC100066228,LOC100146604,LOC100066278,MIR429,LOC100066088,CPSF3L,LOC100147200,LOC100066167,MORN1,LOC100066142,LOC100055525,LOC100061534,LOC100066014,NOC2L,LOC100061495,LOC100147495,LOC100065915,LOC100147581,LOC100065083,GNB1,LOC100064187,LOC100054501,LOC100146417,LOC100061432,SLC18A1
02	47018622	52282521	5263900	3	1	LOC100629449,LOC100057476,CHMP7,LOC100054405,LOC100064361,CDK11A,LOC100064482,LOC100057715,LOC100058083,LOC100066364,LOC100066420,PHYHIP,LOC100057635,LOC100057554,LOC100056584,LOC100066195,LOC100065961,LOC100146191,LOC100061240,LOC100061363,LOC100061283,LOC100064300,LOC100064240,LOC100065322,LOC100065291,MMEL1,TNFRSF14,LOC100053879,C-SKI,LOC100057812,LOC100064452,LOC100064074,LOC100057232,PIWIL2,LOC100056631,LZTS1,LOC100146710,LOC100066254,LOC100057117,LOC100056874,LOC100065860,XPO7,LPL,LOC100147589,LOC100629488,SAMD11,MIR200B,ENTPD4,GFRA2,KLHL17,DOK2,MIR200A,NPM2,LOC100066228,LOC100057997,RHOBTB2,LOC100146604,LOC100066278,MIR429,LOC100066088,LOC100054026,CPSF3L,LOC100147200,LOC100066167,SORBS3,MORN1,LOC100066142,LOC100057357,LOC100055525,LOC100061534,LOC100066014,EGR3,NOC2L,LOC100061495,EPB49,LOC100147495,LOC100065915,LOC100147581,LOC100056444,HR,LOC100146676,BMP1,LGI3,LOC100053773,LOC100065083,GNB1,LOC100064187,LOC100054501,LOC100146417,LOC100061432,SLC18A1,LOC100630167
02	47069434	48976680	1907247	3	5	LOC100629449,LOC100054405,LOC100064361,CDK11A,LOC100064482,LOC100066364,LOC100066420,LOC100066195,LOC100065961,LOC100146191,LOC100061363,LOC100061283,LOC100064300,LOC100064240,LOC100065322,LOC100065291,MMEL1,TNFRSF14,C-SKI,LOC100064452,LOC100064074,LOC100146710,LOC100066254,LOC100065860,LOC100147589,LOC100629488,SAMD11,MIR200B,KLHL17,MIR200A,LOC100066228,LOC100146604,LOC100066278,MIR429,LOC100066088,CPSF3L,LOC100147200,LOC100066167,MORN1,LOC100066142,LOC100061534,LOC100066014,NOC2L,LOC100061495,LOC100147495,LOC100065915,LOC100147581,LOC100065083,GNB1,LOC100064187,LOC100054501,LOC100146417,LOC100061432
02	47109096	48665517	1556422	3	1	LOC100629449,LOC100054405,LOC100064361,CDK11A,LOC100064482,LOC100066364,LOC100066420,LOC100066195,LOC100065961,LOC100146191,LOC100061363,LOC100061283,LOC100064300,LOC100064240,LOC100065322,LOC100065291,MMEL1,TNFRSF14,C-SKI,LOC100064452,LOC100064074,LOC100146710,LOC100066254,LOC100065860,LOC100147589,LOC100629488,SAMD11,MIR200B,KLHL17,MIR200A,LOC100066228,LOC100146604,LOC100066278,MIR429,LOC100066088,CPSF3L,LOC100147200,LOC100066167,MORN1,LOC100066142,LOC100061534,LOC100066014,NOC2L,LOC100061495,LOC100147495,LOC100065915,LOC100147581,LOC100065083,GNB1,LOC100064187,LOC100054501,LOC100146417,LOC100061432

Chrom	Start	End	Size	Cn	Samples (n)	Genes
02	47109096	49214276	2105181	3	1	LOC100629449,LOC100054405,LOC100064361,CDK11A,LOC100064482,LOC100066364,LOC100066420,LOC100066195,LOC100065961,LOC100146191,LOC100061363,LOC100061283,LOC100064300,LOC100064240,LOC100065322,LOC100065291,MMEL1,TNFRSF14,C-SKI,LOC100064452,LOC100064074,LOC100146710,LOC100066254,LOC100065860,LPL,LOC100147589,LOC100629488,SAMD11,MIR200B,KLHL17,MIR200A,LOC100066228,LOC100146604,LOC100066278,MIR429,LOC100066088,CPSF3L,LOC100147200,LOC100066167,MORN1,LOC100066142,LOC100061534,LOC100066014,NOC2L,LOC100061495,LOC100147495,LOC100065915,LOC100147581,LOC100065083,GNB1,LOC100064187,LOC100054501,LOC100146417,LOC100061432
02	48510917	49214276	703360	4	1	LOC100066364,LOC100066420,LOC100146191,LOC100066254,LPL,SAMD11,KLHL17,LOC100146604,LOC100066278,NOC2L
02	55097850	56579811	1481962	3	1	PTK2B,LOC100054365,LOC100054410,SCARA3,LOC100054505,LOC100054461,LOC100060483,LOC100060683,LOC100060380,CLU,ESCO2,LOC100060789,LOC100060414,LOC100060341,LOC100054633
02	61115011	61293831	178821	1	1	HPGD
02	61115011	61408178	293168	1	1	HPGD,CEP44,FBXO8
02	63946952	64615153	668202	1	1	LOC100066817,LOC100066796
02	64250557	64615153	364597	1	1	
02	64250557	64711429	460873	1	2	
02	64286359	64711429	425071	1	1	
02	64527025	64711429	184405	1	1	
02	66273248	67115399	842152	1	1	DDX60L,DDX60,LOC100067630,PALLD,LOC100067586
02	66334525	67812127	1477603	1	1	DDX60L,DDX60,LOC100067630,LOC100067676,SPOCK3,PALLD,LOC100067586
02	66421859	67559539	1137681	1	1	DDX60L,DDX60,LOC100067630,LOC100067676,PALLD,LOC100067586
02	67372188	67524812	152625	1	1	
02	67812127	67972657	160531	1	1	SPOCK3
02	70212772	70850201	637430	1	1	LOC100630121,LOC100061618
02	70250056	70423370	173315	1	1	
02	70250056	70583259	333204	1	2	
02	70367017	70850201	483185	1	1	LOC100630121,LOC100061618
02	72030268	73088779	1058512	1	1	FSTL5,LOC100630190
02	72363378	73832997	1469620	1	1	FSTL5,LOC100630341
02	74343328	74420255	76928	4	1	
02	76399235	77695624	1296390	1	1	PDGFC,LOC100069308,GLRB,GRIA2
02	76470475	77900734	1430260	1	1	PDGFC,LOC100069308,GLRB,GRIA2
02	82272295	82893361	621067	1	1	LOC100062429,LOC100062495,SH3D19
02	93508775	94100843	592069	1	1	LOC100071530
02	96664019	96701792	37774	1	1	
02	97254688	98634954	1380267	1	1	LOC100629764
02	106062109	106063099	991	0	1	
02	106062109	106063373	1265	0	17	
02	108805508	109132021	326514	3	1	LOC100072455,NDST3,LOC100629617
02	110298220	110703036	404817	1	1	
02	110298220	110878106	579887	1	1	
02	110487412	110669459	182048	1	1	
02	113831365	114606325	774961	1	1	LOC100072722,ALPK1,LOC100064257,LOC100629547
02	113908861	114606325	697465	1	1	LOC100072722,LOC100629547
03	1158391	2148623	990233	3	1	ABCC12,LONP2,LOC100057532,N4BP1,LOC100057458
03	16360994	17054428	693435	3	1	LOC100065253,LOC100629653
03	16360994	18371411	2010418	3,4	4,1	LRRC36,LCAT,PSKH1,LOC100066295,RANBP10,GFOD2,LOC100066184,LOC100066101,LOC100066082,LOC100053217,LOC100053074,LOC100147214,LOC100065668,PDP2,CCDC79,LOC100065490,LOC100065253,LOC100053509,LOC100065396,LOC100065583,LOC100065750,CDH16,LOC100053119,LOC100066033,MIR328,LOC100066156,LOC100053553,LOC100066381,LOC100066355,DUS2L,LOC100053459,FHOD1,HSF4,LOC100053028,LOC100629653,TK2,LOC100629814,LOC100052927,HSD11B2,LOC100065651,LOC100053269,CTCF,LOC100066270,LOC100066215,EDC4,NAE1,DPEP2,LOC100066464,ATP6V0D1,PARD6A,CES3,LOC100629739,LOC100629698,LOC100065469,PLEKHG4,LOC100629765,LOC100065726,LOC100065795,LOC100065878,LOC100065953,LOC100066008,LOC100066409,FAM96B,LOC100065824,LOC100053706

Chrom	Start	End	Size	Cn	Samples (n)	Genes
03	17464411	18550363	1085953	4	1	LRRC36,LOC100053856,LCAT,PSKH1,LOC100066295,RANBP10,GFOD2,LOC100066184,LOC100066101,LOC100066082,LOC100053217,LOC100053074,LOC100147214,LOC100065668,LOC100053509,LOC100065750,LOC100053119,LOC100066033,MIR328,LOC100066156,LOC100053553,LOC100066381,LOC100066355,DUS2L,LOC100053459,FHOD1,HSF4,LOC100053028,LOC100052927,HSD11B2,LOC100053269,CTCF,LOC100066270,LOC100066215,EDC4,DPEP2,LOC100066464,PLA2G15,ATP6V0D1,PARD6A,CES3,LOC100066658,PLEKHG4,LOC100066567,LOC100065726,LOC100065795,LOC100065878,LOC100065953,LOC100066008,LOC100066409,FAM96B,LOC100065824,LOC100053706
03	20395815	20636965	241151	4	1	
03	20446074	20636965	190892	4	1	
03	25592835	28454042	2861208	3	1	LOC100069343,LOC100069257,LOC100147597,LOC100629542,LOC100629658,VAT1L,WWOX,LOC100069465,ADAMTS18,LOC100055505,LOC100146831
03	26678067	29269059	2590993	4	1	LOC100629755,LOC100069767,LOC100630087,LOC100055628,GAN,PKD1L2,LOC100629658,BCMO1,ATMIN,WWOX,CDYL2,LOC100069465,LOC100146831
03	26921988	28275761	1353774	3	1	WWOX,LOC100069465,LOC100146831
03	26921988	29459812	2537825	3	1	PLCG2,LOC100069901,LOC100629755,LOC100069767,LOC100630087,LOC100055628,GAN,PKD1L2,LOC100629658,BCMO1,ATMIN,WWOX,CDYL2,LOC100069465,LOC100146831
03	27483994	29368360	1884367	3	1	LOC100069901,LOC100629755,LOC100069767,LOC100630087,LOC100055628,GAN,PKD1L2,LOC100629658,BCMO1,ATMIN,CDYL2,LOC100069465,LOC100146831
03	28847004	29640856	793853	1	2	PLCG2,LOC100146214,LOC100069901,LOC100629755,LOC100055628,GAN,PKD1L2,LOC100055715,BCMO1
03	29244933	29553559	308627	4	1	PLCG2,LOC100069901
03	31028562	33143624	2115063	3	1	COXIV,LOC100070389,LOC100070233,LOC100070154,LOC100070186,LOC100070396,LOC100070301,LOC100070274,CRISPLD2,KCNG4,LOC100055931,OSGIN1,CDH13,LOC100070093,LOC100055805,ADAD2,KLHL36,LOC100056089,LOC100070367,MBTPS1,LRRC50,SLC38A8,LOC100055972,LOC100070049,USP10,LOC100630488,ATP2C2,LOC100070195,LOC100056131,IRF8
03	31044613	32376517	1331905	3	1	LOC100070233,LOC100070154,LOC100070186,LOC100070301,LOC100070274,CRISPLD2,KCNG4,LOC100055931,OSGIN1,CDH13,LOC100070093,LOC100055805,ADAD2,KLHL36,LOC100056089,MBTPS1,LRRC50,SLC38A8,LOC100055972,LOC100070049,USP10,ATP2C2,LOC100070195
03	31103014	35661804	4558791	3	1	LOC100146434,FOXC2,LOC100630583,LOC100630312,MVD,LOC100051426,COXIV,LOC100070389,LOC100070233,LOC100070154,LOC100070186,ACSF3,LOC100051284,LOC100050994,CTU2,BANP,ZCCHC14,FBXO31,LOC100070396,LOC100070301,LOC100070274,CRISPLD2,KCNG4,LOC100055931,OSGIN1,CDH13,LOC100070093,LOC100055805,ADAD2,KLHL36,LOC100056089,LOC100070367,MBTPS1,LRRC50,SLC38A8,LOC100055972,LOC100070049,USP10,LOC100630488,SLC7A5,LOC100050042,CDT1,LOC100051357,LOC100147110,LOC100050842,LOC100051771,LOC100050917,LOC100050529,LOC100051212,LOC100050766,LOC100056257,LOC100630367,LOC100070642,ATP2C2,LOC100070195,LOC100056131,IRF8,LOC100070485,LOC100070617,LOC100050687,LOC100049843
03	31985444	33418808	1433365	3	1	LOC100146434,FOXC2,COXIV,LOC100070389,LOC100070396,LOC100070301,LOC100070274,CRISPLD2,LOC100056089,LOC100070367,LOC100630488,LOC100056131,IRF8,LOC100070485
03	32241051	32376517	135467	3,4	1,3	
03	32241051	32472807	231757	3	1	
03	32241051	33143624	902574	3	1	COXIV,LOC100070389,LOC100070396,LOC100070367,LOC100630488,LOC100056131,IRF8
03	34842306	35661804	819499	3,4	1,1	MVD,LOC100051426,ACSF3,LOC100051284,LOC100050994,CTU2,LOC100050042,CDT1,LOC100051357,LOC100050842,LOC100051771,LOC100050917,LOC100050529,LOC100051212,LOC100050766,LOC100050687,LOC100049843
03	35084304	35661804	577501	3,4	2,1	MVD,LOC100051426,ACSF3,LOC100051284,LOC100050994,CTU2,LOC100050042,CDT1,LOC100051357,LOC100050842,LOC100051771,LOC100050917,LOC100051212,LOC100050766,LOC100050687,LOC100049843
03	35284842	35321182	36341	1	1	LOC100051284,LOC100050994,CDT1,LOC100051212
03	35284842	35953765	668924	1	1	LOC100051426,ACSF3,LOC100051284,LOC100050994,LOC100050042,CDT1,LOC100051357,LOC100051771,LOC100051834,LOC100051212,ANKRD11,SPG7
03	35284842	36614830	1329989	1	1	LOC100629183,LOC100051426,CDK10,DPEP1,GAS8,LOC100050173,LOC100053365,LOC100053318,MC1R,LOC100050244,LOC100052260,ACSF3,LOC100051284,LOC100050994,LOC100050042,CDT1,LOC100051357,SPIRE2,FANCA,CPNE7,DEF8,LOC100053030,LOC100053607,LOC100051771,LOC100146535,LOC100053168,LOC100052875,LOC100052665,LOC100052376,LOC100051834,LOC100051212,ANKRD11,SPG7,ZNF276,LOC100052929
03	37121854	37957776	835923	3	1	LOC100630481,BANK1,LOC100067894,LOC100067947,LOC100067821
03	41567820	41635136	67317	3	4	
03	45003697	47970675	2966979	1	1	LOC100064679,GRID2
03	45062312	45062839	528	1	1	GRID2
03	45062312	45063260	949	1	1	GRID2
03	45062312	47573471	2511160	1	1	LOC100064679,GRID2
03	47355091	47970675	615585	1	1	LOC100064679
03	47452823	47573471	120649	1	1	LOC100064679
03	49581906	49650451	68546	1	1	PPM1K
03	49641059	49650451	9393	1	2	
03	51525184	51729578	204395	1	1	MAPK10,ARHGAP24
03	65705932	65951800	245869	3	1	LOC100067903,LOC100066472,LOC100629168,LOC100067952,LOC100067974,LOC100067994,LOC100629252,LOC100629210,LOC100066501,LOC100068012
03	65932710	65951800	19091	1	3	LOC100066472,LOC100629252,LOC100066501

Chrom	Start	End	Size	Cn	Samples (n)	Genes
03	65932710	66369357	436648	4	1	LOC100146884,LOC100067686,LOC100066444,LOC100066472,UGT2B4,LOC100067777,LOC100629252,LOC100067660,LOC100067640,LOC100066501,LOC100066415,LOC100066386,LOC100067616
03	70240358	71834264	1593907	1	1	LOC100629818,LPHN3
03	70630601	70845155	214555	1	1	
03	70630601	72064340	1433740	1	1	LOC100629818,LPHN3
03	73562856	74557812	994957	1	1	LOC100629837
03	73848680	74495490	646811	1	2	
03	73881123	74557812	676690	1	1	
03	74052752	74495490	442739	1	1	
03	75235988	75491946	255959	1	2	
03	76305217	76393636	88420	1	2	AASDH,PAICS,PPAT,SRP72
03	76305217	76430682	125466	1	1	AASDH,PAICS,LOC100058662,PPAT,SRP72
03	82126801	83150021	1023221	1	1	GABRA2,LOC100054705
03	82686862	82929276	242415	1	1	
03	90364982	96218268	5853287	1	1	LOC100065825,PCDH7,DTHD1,LOC100066217,ARAP2,LOC100066157
03	91213313	91353260	139948	1	1	
03	92567541	96218268	3650728	1	1	LOC100065825,PCDH7,LOC100066217,LOC100066157
03	92650248	92841550	191303	1	1	
03	93187203	94418288	1231086	1	1	LOC100065825
03	103586464	103835174	248711	1	1	SLIT2
03	103687667	104073331	385665	1	1	SLIT2
03	104695238	104986284	291047	1	1	
03	107201471	107304237	102767	4	1	PROM1
03	114386529	116903728	2517200	3	1	SH3TC1,LOC100056426,SORCS2,LOC100070368,LOC100146688,RGS12,LOC100070466,LOC100070406,ACOX3,LOC100070415,LOC100070323,LOC100056472,LOC100056606,TBC1D14,AFAP1,LOC100054064,LOC100070335,MIR95,LOC100056382,LOC100070438,LOC100056513,LOC100054020,LOC100056560
03	114602741	114825361	222621	1	1	TBC1D14,LOC100056560
03	114825361	116740303	1914943	3	2	SH3TC1,SORCS2,LOC100070368,LOC100070466,LOC100070406,ACOX3,LOC100070415,LOC100070323,LOC100056606,TBC1D14,AFAP1,LOC100054064,LOC100070335,MIR95,LOC100070438
03	114825361	116903728	2078368	3	1	SH3TC1,SORCS2,LOC100070368,LOC100146688,RGS12,LOC100070466,LOC100070406,ACOX3,LOC100070415,LOC100070323,LOC100056606,TBC1D14,AFAP1,LOC100054064,LOC100070335,MIR95,LOC100070438,LOC100054020
03	114951458	116903728	1952271	3	1	SH3TC1,SORCS2,LOC100070368,LOC100146688,RGS12,LOC100070466,LOC100070406,ACOX3,LOC100070415,AFAP1,LOC100054064,MIR95,LOC100070438,LOC100054020
03	116036468	116740303	703836	3	2	LOC100070466,ACOX3,LOC100070415,LOC100054064,LOC100070438
03	116036468	116818260	781793	3	1	LOC100146688,RGS12,LOC100070466,ACOX3,LOC100070415,LOC100054064,LOC100070438,LOC100054020
03	116036468	116903728	867261	3	1	LOC100146688,RGS12,LOC100070466,ACOX3,LOC100070415,LOC100054064,LOC100070438,LOC100054020
03	118147141	119456949	1309809	3	1	LOC100052572,LOC100050943,FGFR3,LOC100050793,LOC100051730,IDUA,LOC100051976,TMEM175,LOC100052156,LOC100052514,LOC100052397,LETM1,LOC100052280,GAK,LOC100629290,TACC3,MFSD7,LOC100630123,LOC100052893,LOC100051591,WHSC1,LOC100052841,PIGG,LOC100630904,CTBP1
03	118524879	119259174	734296	1	1	LOC100050943,IDUA,LOC100051976,TMEM175,LOC100052156,LOC100052397,LOC100052280,GAK,LOC100630123,LOC100630904,CTBP1
03	118810649	119238061	427413	1	1	LOC100050943,IDUA,LOC100051976,TMEM175,LOC100052156,GAK,LOC100630123,LOC100630904,CTBP1
03	118844264	119210792	366529	1	1	LOC100050943,IDUA,LOC100051976,TMEM175,LOC100052156,GAK,LOC100630123,LOC100630904,CTBP1
03	119147934	119374499	226566	3	1	LOC100050943,LOC100050793,LOC100051730,GAK,LOC100629290,MFSD7,LOC100051591
03	119221121	119374499	153379	3,4	5,2	LOC100050943,LOC100050793,LOC100051730,LOC100629290,MFSD7,LOC100051591
03	119221121	119456949	235829	3,4	2,1	LOC100050943,LOC100050793,LOC100051730,LOC100629290,MFSD7,LOC100051591,PIGG
03	119238061	119374499	136439	3,4	1,6	LOC100050943,LOC100050793,LOC100051730,LOC100629290,MFSD7,LOC100051591
03	119238061	119456949	218889	3	1	LOC100050943,LOC100050793,LOC100051730,LOC100629290,MFSD7,LOC100051591,PIGG
03	119259174	119374499	115326	3,4	1,2	LOC100050793,LOC100051730,LOC100629290,MFSD7,LOC100051591
03	119259174	119456949	197776	3	2	LOC100050793,LOC100051730,LOC100629290,MFSD7,LOC100051591,PIGG
03	119271034	119374499	103466	4	4	LOC100050793,LOC100051730,LOC100629290,MFSD7,LOC100051591
03	119271034	119456949	185916	3	1	LOC100050793,LOC100051730,LOC100629290,MFSD7,LOC100051591,PIGG
03	119274852	119374499	99648	4	2	LOC100050793,LOC100051730,LOC100629290,MFSD7,LOC100051591
04	9121425	9500292	378868	1	3	LOC100062122,LOC100061824,LOC100629239,LOC100061654
04	9374294	9500292	125999	1	6	LOC100062122

Chrom	Start	End	Size	Cn	Samples (n)	Genes
04	9404330	9500292	95963	1	2	LOC100062122
04	9436497	9500292	63796	0	1	
04	12120524	12316520	195997	1	1	
04	14943417	16836115	1892699	3	1	LOC100065225,LOC100065563,DDX56,LOC100629965,LOC100065283,LOC100065012,LOC100064946,LOC100065542,LOC100065072,LOC100146667,IGFBP-1,IGFBP-3,MYO1G,CCM2,LOC100146368,LOC100147367,LOC100051895,GCK,LOC100052020,LOC100629504,ZMIZ2,LOC100065105,LOC100065443,LOC100065195
04	22227867	23809020	1581154	1	1	LOC100630391,LOC100067293,LOC100067342,LOC100630459
04	22737039	23809020	1071982	1	1	LOC100630459
04	22802309	23158551	356243	1	1	LOC100630459
04	23011397	23158551	147155	1	3	
04	26341606	28114554	1772949	1	1	LOC100630689,LOC100630418,LOC100049848,LOC100057788,SEMA3E,LOC100057533
04	26703271	26885157	181887	1	1	LOC100057533
04	27610865	27682684	71820	1	1	LOC100057788
04	27610865	28114554	503690	1	1	LOC100057788,SEMA3E
04	29161357	29680020	518664	1	2	SEMA3D
04	29536635	29680020	143386	1	1	SEMA3D
04	31767732	32854849	1087118	1	1	ABCB1,LOC100050248,DBF4,ADAM22,LOC100059619,STEAP4,RUNDC3B,LOC100147513
04	31767732	33747940	1980209	1	1	ABCB1,LOC100050248,LOC100050533,DBF4,ADAM22,LOC100059619,STEAP4,ZNF804B,RUNDC3B,LOC100147513
04	32262280	33976948	1714669	1	1	LOC100050533,ADAM22,LOC100059619,STEAP4,ZNF804B
04	33744397	33747940	3544	0	1	
04	42536178	43036361	500184	1	1	LOC100064528
04	42536178	43480801	944624	1	1	LOC100064528
04	44608273	49234027	4625755	1	1	LOC100052666,BZW2,ETV1,LOC100066103,LOC100629346,AGMO,LOC100064843,ISPD,LOC100064947,LOC100052612,VWDE,LOC100146333,DGKB,LOC100052826,LOC100052877,LOC100066034,ANKMY2
04	48124299	48966208	841910	1	2	AGMO,ISPD,LOC100052826
04	48343348	48710310	366963	1	1	ISPD
04	50765212	51725167	959956	1	1	LOC100630499,TWIST1,LOC100053170,LOC100053221,TWISTNB,LOC100053273
04	52424614	52612016	187403	3	1	LOC100067077
04	70612792	70795004	182213	1	1	LOC100070896,GPR85
04	75575124	76181703	606580	1	1	LOC100071333
04	83786209	84541074	754866	4	1	LOC100071949,NRF1,SMO,IRF5,AHCYL2,LOC100071778,LOC100071795,LOC100071801,TRNAY-AUA,LOC100629702,LOC100071840,MIR182,MIR183,MIR96
04	88967074	90022761	1055688	3	1	LOC100067251,CNOT4,LOC100630135,LOC100067510,LOC100065089,LOC100065055,LOC100067707,SLC13A4,LOC100146184,LOC100147185,LOC100067668,LOC100067445,LOC100629333,LOC100064995,LOC100067491,LOC100630834
04	96985336	97011232	25897	3,4	14,5	LOC100057252
04	97565169	99317651	1752483	1	1	CNTNAP2,LOC100058787,LOC100058622
04	98289000	98326437	37438	1	1	
04	101271134	101389161	118028	1	1	TRNAC-GCA,TRNAC-GCA,TRNAC-GCA,ZNF212,TRNAC-GCA,TRNAC-GCA,TRNAC-GCA,LOC100060969,TRNAC-GCA
04	107915962	108032391	116430	1	1	PTPRN2
05	914733	1456712	541980	4	1	TRNAK-UUU,PIK3C2B,NFASC,LRRN2,MDM4,TRNAK-UUU
05	914733	3359264	2444532	3	1	LOC100055720,LOC100055853,DSTYK,LOC100055118,EIF2D,LOC100055588,LOC100629326,NUAK2,SLC45A3,LOC100050612,LOC100050179,TRNAK-UUU,PIK3C2B,NFASC,LRRN2,LOC100054663,MIR135B,KLHDC8A,LOC100050250,ELK4,LOC100055030,SLC41A1,LOC100050458,SLC26A9,LOC100629681,LOC100050772,LOC100054804,LOC100629703,LOC100055210,SRGAP3,LOC100051000,IKBKE,IL10,LOC100055510,LOC100055632,LOC100055675,CTSE,PFKFB2,LOC100055935,LOC100056094,PIGR,RBBP5,YOD1,LOC100055466,AVPR1B,LOC100629583,MDM4,TRNAK-UUU
05	29575610	31691289	2115680	3	1	LOC100060786,LOC100060678,LOC100057656,PLXNA2,LOC100051718,MIR29C,LOC100146314,LOC100057176,LOC100146620,MIR29C-2,LOC100629269,LOC100060905,LOC100060824,LOC100057335,LOC100057500,MIR29B-2,LOC100060860,LOC100057298
05	29996193	31861781	1865589	3	1	LOC100060786,LOC100060678,LOC100057656,PLXNA2,LOC100051718,MIR29C,LOC100146314,LOC100058551,LOC100057176,LOC100146620,MIR29C-2,LOC100629269,LOC100060905,LOC100060824,LOC100058590,LOC100057335,LOC100057500,MIR29B-2,LOC100060860,LOC100057298
05	30779774	32111740	1331967	4	1	LOC100060786,LOC100060678,LOC100060443,LOC100057656,LOC100058551,LOC100629269,LOC100060905,ALDH9A1,LOC100146211,LOC100058514,LOC100060824,LOC100058590,LOC100060860
05	37840041	37916448	76408	0	10	LOC100053475,LOC100057667

Chrom	Start	End	Size	Cn	Samples (n)	Genes
05	41866007	44433546	2567540	3	1	LOC100063783,ILF2,LOC100061699,LOC100061663,LOC100061598,LOC100630205,S100A7,LOC100056390,LOC100056352,LOC100147656,LOC100056181,LOC100147270,INTS3,NUP210L,LOC100061766,LOC100056619,DENND4B,UBAP2L,LOC100062988,LOC100062963,ADAR,LOC100062475,LOC100063751,LOC100057102,TRIM46,LOC100063378,ADAM15,LOC100063514,GON4L,LOC100063673,ASH1L,LOC100063574,LOC100146589,LOC100630789,LOC100057624,LOC100064105,LOC100057221,LOC100063842,LOC100629772,LOC100057746,LMNA,MIR1905B,MEX3A,LOC100063909,LOC100147069,LOC100630377,LOC100063608,FDPS,LOC100063479,SYT11,THBS3,LOC100063307,LOC100063240,LOC100057186,DCST2,FLAD1,LOC100063415,LOC100630884,UBE2Q1,LOC100062544,LOC100062621,LOC100062408,LOC100063020,LOC100056786,CREB3L4,CRTC2,LOC100062274,LOC100146983,LOC100057540,NPR1,LOC100056751,LOC100056268,LOC100061923,LOC100056230,S100A6,LOC100061796,LOC100056434,LOC100061565,LOC100061461,LOC100061531,LOC100061730,TRNAM-CAU,ARHGEF2,SHC1,LOC100061631,LOC100630030,LOC100056308,LOC100629559,LOC100062377,LOC100056899,LOC100057020,ZBTB7B,LOC100063346,LOC100057345,LOC100057304,LOC100057427,LOC100057704,SEMA4A,CCT3,LOC100630233,LOC100056061,LOC100061891,GATAD2B,MIR190B,LOC100056861,LOC100629225,DCST1,MIR92B,CLK2,LOC100063939,LOC100064070,TMEM79,LOC100064158
05	43408448	43672120	263673	1	1	NUP210L,LOC100056619,DENND4B,UBAP2L,LOC100062475,LOC100062408,LOC100056786,CREB3L4,CRTC2,LOC100062274,LOC100056751,LOC100629559,LOC100062377,LOC100056899,MIR190B,LOC100056861,LOC100629225
05	46644366	47558116	913751	4	1	LOC100054581,TRNAN-GUU,RPRD2,LOC100054535,LOC100630631,LOC100058752,LOC100054058,LOC100054015,LOC100053968,TRNAE-UUC,TRNAG-CCC,LOC100054253,TRNAN-GUU,LOC100630206,TRNAV-CAC,TRNAG-CCC,LOC100059700,TRNAN-GUU,TRNAH-GUG,TRNAN-GUU,TRNAH-GUG,TRNAE-UUC,TRNAG-CCC,LOC100059796,LOC100068789,LOC100054211,LOC100146788,OTUD7B,LOC100058935,TRNAE-UUC,LOC100058378,LOC100058587,LOC100054302,LOC100058630,VPS45,LOC100054493,LOC100058902,PRPF3,TRNAH-GUG,TRNAE-UUC,TRNAG-CCC,TRNAE-UUC,LOC100059737,TRNAV-CAC,TRNAN-GUU,TRNAH-GUG,TRNAN-GUU,TRNAH-GUG,TRNAG-CCC,LOC100058466,LOC100054164,LOC100054113,LOC100054350
05	47239959	47259141	19183	4	1	LOC100068789
05	47359920	47399241	39322	3,4	1,1	TRNAG-CCC,LOC100059796
05	50444342	50508657	64316	1	1	TBX15
05	52955124	53121994	166871	1	1	SLC22A15,LOC100059622
05	58180075	58242387	62313	1	1	AHCYL1,LOC100061953
05	61394410	61474450	80041	1	1	LOC100060031
05	61877738	62238822	361085	1	1	LOC100630089
05	61877738	62844610	966873	1	1	LOC100054441,LOC100630089
05	63531114	64695513	1164400	1	1	LOC100049922,OLFM3
05	67648876	67922015	273140	1	2	DPYD
05	67648876	68145001	496126	1	1	DPYD
05	67745265	67922015	176751	1	1	DPYD
05	67745265	67984476	239212	1	1	DPYD
05	69413874	69630650	216777	1	1	
05	73047724	73094727	47004	1	8	
05	77418462	77435333	16872	1	1	CLCA4
05	81925834	82555936	630103	1	1	LPHN2,LOC100629645
05	83237729	83572583	334855	1	1	
05	88016086	88820623	804538	1	1	LRRIQ3
05	88196782	88349479	152698	3	1	
05	88243192	88258862	15671	3,4	6,1	
05	88243192	88327924	84733	4	1	
05	89912682	90369184	456503	3	1	NEGR1
05	97113497	98659617	1546121	3	1	KANK4,ATG4C,ANGPTL3,USP1,LOC100054206,LOC100070493,INADL,LOC100070600,LOC100070529,DOCK7,LOC100070451
05	99595549	99631010	35462	3,4	1,9	LOC100070758
06	4565099	4690824	125726	1	1	LOC100050318
06	4645237	4690824	45588	1	2	LOC100050318
06	6733254	7792337	1059084	3	1	TNP1,LOC100057976,TNS1
06	7691534	8828001	1136468	3	1	LOC100055855,LOC100055936,TTLL4,TUBA4A,LOC100056264,LOC100056303,TUBA1B,LOC100146135,SLC23A3,LOC100058824,LOC100147528,IHH,CCDC108,LOC100059090,LOC100056095,LOC100056056,LOC100058895,GLB1L,ANKZF1,LOC100629459,PRKAG3,LOC100056136,ABCB6,TNS1,LOC100058624,LOC100055552,LOC100055590,LOC100058459,LOC100058331,LOC100058291,LOC100058415,LOC100055634,PNKD,USP37,VIL1,LOC100058665,ZNF142,RUFY4,RNF25,LOC100056346,LOC100058931,BCS1L,SLC11A1

Chrom	Start	End	Size	Cn	Samples (n)	Genes
06	7970048	8991955	1021908	3	1	LOC100055855,LOC100055936,TTLL4,TUBA4A,LOC100056264,LOC100056303,TUBA1B,LOC100059355,LOC100146135,SLC23A3,LOC100058824,LOC100147528,IHH,CCDC108,LOC100059090,LOC100056095,LOC100056056,LOC100058895,GLB1L,MIR153-2,SPEG,ANKZF1,LOC100629459,PRKAG3,LOC100056136,ABCB6,LOC100058624,LOC100058459,LOC100055634,PNKD,USP37,VIL1,LOC100058665,ZNF142,RNF25,LOC100059397,LOC100629481,LOC100056346,LOC100058931,BCS1L,SLC11A1
06	13553472	14001425	447954	1	1	DOCK10,LOC100061790
06	14349141	14562760	213620	1	1	
06	19226771	19684498	457728	1	1	LOC100064682,LOC100057254,LOC100064555,DIS3L2,LOC100057215,LOC100147527,LOC100064653
06	21483552	22950180	1466629	3	1	LOC100630851,LOC100065981,LOC100065931,CXCR7,IQCA1,LOC100630909,SH3BP4
06	24181261	25327436	1146176	3	1	ILKAP,LOC100066710,LOC100067014,TRAF3IP1,PER2
06	24181261	27067185	2885925	3	1	LOC100147141,LOC100067872,ILKAP,SEPT2,LOC100067436,PASK,LOC100067459,LOC100067149,LOC100066710,LOC100057698,LOC100067014,LOC100057739,TRAF3IP1,LOC100067126,LOC100067193,LOC100067241,MIR149,PER2,LOC100067268,KIF1A,LOC100067478,LOC100147336,MTERFD2,LOC100057882,ANO7,FARP2,LOC100067637,LOC100146642,LOC100067736,LOC100067850,LOC100067824,LOC100067898,ATG4B,LOC100067948,THAP4,LOC100146641,SNED1,LOC100067218,LOC100067171,LOC100057791,LOC100067104
06	25794652	26243259	448608	3	1	LOC100067436,MIR149,LOC100067268,KIF1A,LOC100147336,LOC100146642
06	25794652	26582849	788198	4	1	LOC100067436,PASK,LOC100067459,MIR149,LOC100067268,KIF1A,LOC100067478,LOC100147336,MTERFD2,LOC100057882,ANO7,LOC100067637,LOC100146642,SNED1
06	25794652	26723584	928933	3	1	SEPT2,LOC100067436,PASK,LOC100067459,MIR149,LOC100067268,KIF1A,LOC100067478,LOC100147336,MTERFD2,LOC100057882,ANO7,FARP2,LOC100067637,LOC100146642,SNED1
06	25908752	26126581	217830	3,4	6,17	LOC100067436,MIR149,LOC100067268,LOC100147336,LOC100146642
06	25908752	26582849	674098	3,4	1,2	LOC100067436,PASK,LOC100067459,MIR149,LOC100067268,KIF1A,LOC100067478,LOC100147336,MTERFD2,LOC100057882,ANO7,LOC100067637,LOC100146642,SNED1
06	25908752	27594478	1685727	4	1	LOC100147141,LOC100054442,LOC100067872,USP18,SEPT2,LOC100067436,PASK,LOC100067459,MIR149,LOC100067268,KIF1A,LOC100067478,LOC100147336,MTERFD2,LOC100057882,ANO7,FARP2,LOC100067637,LOC100146642,LOC100067736,LOC100067850,LOC100067824,LOC100067898,ATG4B,LOC100067948,LOC100054664,TUBA8,LOC100054988,ATP6V1E1,THAP4,BCL2L13,LOC100054807,LOC100146641,SNED1
06	26029975	26723584	693610	1	1	SEPT2,LOC100067436,PASK,LOC100067459,KIF1A,LOC100067478,LOC100147336,MTERFD2,LOC100057882,ANO7,FARP2,LOC100067637,LOC100146642,SNED1
06	26086675	26126581	39907	3,4	7,5	LOC100067436,LOC100147336
06	26086675	26582849	496175	3,4	1,2	LOC100067436,PASK,LOC100067459,KIF1A,LOC100067478,LOC100147336,MTERFD2,LOC100057882,ANO7,LOC100067637,SNED1
06	26102028	26126581	24554	3,4	6,1	LOC100067436
06	26104234	26109288	5055	0	2	
06	26104234	26118925	14692	1,3	2,1	LOC100067436
06	26109163	26126581	17419	3	1	LOC100067436
06	31542235	31877018	334784	3	1	PARP11,LOC100057792
06	35622725	35648056	25332	1	1	LOC100053416
06	36115483	38906872	2791390	1	1	LOC100053808,LOC100062618,LOC100630334,LOC100062929,LOC100630523,LOC100065343,LOC100065075,LY49D,LOC100062060,LOC100061763,LOC100061692,A2M,LOC100061791,LOC100630090,LOC100053558,LOC100630149,LOC100061988,LOC100630105,LOC100062337,LOC100053863,LOC100062303,LOC100062688,LOC100062540,LOC100062789,LOC100063085,LOC100062846,LY49B,LOC100629907,STYK1,LY49C,LOC100065316,LOC100065376,LOC100065228,LOC100065445,LOC100062092,LOC100065197,LOC100053615,LOC100065421,LOC100146813,LOC100065399,LOC100065286,LOC100063267,LOC100065107,LY49E,LOC100063178,LY49F,LOC100062904,LOC100062823,LOC100062756,LOC100053963,LOC100053911,LOC100062403,LOC100062370,LOC100053758,CLEC12A,LOC100061827
06	36171791	38906872	2735082	1	1	LOC100053808,LOC100062618,LOC100630334,LOC100062929,LOC100630523,LOC100065343,LOC100065075,LY49D,LOC100062060,LOC100061763,LOC100061692,LOC100061791,LOC100630090,LOC100053558,LOC100630149,LOC100061988,LOC100630105,LOC100062337,LOC100053863,LOC100062303,LOC100062688,LOC100062540,LOC100062789,LOC100063085,LOC100062846,LY49B,LOC100629907,STYK1,LY49C,LOC100065316,LOC100065376,LOC100065228,LOC100065445,LOC100062092,LOC100065197,LOC100053615,LOC100065421,LOC100146813,LOC100065399,LOC100065286,LOC100063267,LOC100065107,LY49E,LOC100063178,LY49F,LOC100062904,LOC100062823,LOC100062756,LOC100053963,LOC100053911,LOC100062403,LOC100062370,LOC100053758,CLEC12A,LOC100061827
06	37137122	37769135	632014	1	1	LOC100053808,LOC100062618,LOC100630334,LOC100053863,LOC100062688,LOC100062540,LOC100062789,LOC100062846,LOC100062823,LOC100062756,LOC100053963,LOC100053911,LOC100062403,LOC100062370
06	37137122	38906872	1769751	1	1	LOC100053808,LOC100062618,LOC100630334,LOC100062929,LOC100630523,LOC100065343,LOC100065075,LY49D,LOC100053863,LOC100062688,LOC100062540,LOC100062789,LOC100063085,LOC100062846,LY49B,STYK1,LY49C,LOC100065316,LOC100065376,LOC100065228,LOC100065445,LOC100065197,LOC100065421,LOC100146813,LOC100065399,LOC100065286,LOC100063267,LOC100065107,LY49E,LOC100063178,LY49F,LOC100062904,LOC100062823,LOC100062756,LOC100053963,LOC100053911,LOC100062403,LOC100062370
06	37342589	37433398	90810	1	1	LOC100062540
06	37349867	37433398	83532	1	1	LOC100062540
06	37380955	38278874	897920	1	1	LOC100062618,LOC100630334,LOC100062929,LOC100630523,LOC100062688,LOC100062789,LOC100063085,LOC100062846,LY49B,LY49F,LOC100062904,LOC100062823,LOC100062756,LOC100053963

Chrom	Start	End	Size	Cn	Samples (n)	Genes
06	37769135	38278874	509740	3	1	LOC100062929,LOC100630523,LOC100063085,LY49B,LY49F,LOC100062904
06	38278097	38421550	143454	3	1	LY49B,LY49C,LY49E,LOC100063178,LY49F
06	44222679	45239839	1017161	1	2	PIK3C2G,LOC100067737
06	45232251	45239839	7589	1	1	PIK3C2G
06	57940767	58683033	742267	1	1	KIF21A,CPNE8
06	68956938	70285441	1328504	3	1	LOC100146158,KRT86,LOC100062930,LOC100061487,LOC100062757,LOC100061525,LOC100063215,LOC100063410,LOC100061828,TENC1,LOC100062061,LOC100062026,ESPL1,LOC100062123,KRT4,LOC100061693,KRT5,LOC100062582,KRT7,LOC100061353,LOC100062985,KRT82,LOC100146665,LOC100061560,LOC100063179,LOC100063342,LOC100061764,LOC100147555,LOC100063542,EIF4B,SOAT2,LOC100063712,SPRYD3,AAAS,LOC100062093,ITGB7,CSAD,IGFBP-6,LOC100063570,LOC100063511,KRT79,LOC100063376,KRT77,LOC100063302,LOC100146152,KRT73,KRT71,LOC100061595,LOC100061458,LOC100061422,LOC100146362,LOC100062875,KRT80,LOC100062689,ACVR1B,ACVRL1,LOC100630598,SCN8A
06	69044079	70385588	1341510	4	1	LOC100146158,KRT86,LOC100062930,LOC100061487,LOC100062757,LOC100061525,LOC100063215,LOC100063410,LOC100061828,TENC1,LOC100062061,LOC100062026,ESPL1,LOC100062123,KRT4,LOC100061693,KRT5,LOC100062582,KRT7,LOC100061353,LOC100062985,KRT82,LOC100146665,LOC100061560,LOC100063179,LOC100063342,LOC100061764,LOC100147555,LOC100063542,EIF4B,SOAT2,LOC100063712,SPRYD3,SP7,AMHR2,LOC100629527,AAAS,LOC100062093,ITGB7,CSAD,IGFBP-6,LOC100063570,LOC100063511,KRT79,LOC100063376,KRT77,LOC100063302,LOC100146152,KRT73,KRT71,LOC100061595,LOC100061458,LOC100061422,LOC100146362,LOC100062875,KRT80,LOC100062689,ACVR1B,ACVRL1
06	69187193	70285441	1098249	3	1	KRT86,LOC100062930,LOC100061487,LOC100062757,LOC100061525,LOC100063215,LOC100063410,LOC100061828,TENC1,LOC100062061,LOC100062026,ESPL1,LOC100062123,KRT4,LOC100061693,KRT5,KRT7,LOC100061353,LOC100062985,KRT82,LOC100146665,LOC100061560,LOC100063179,LOC100063342,LOC100061764,LOC100147555,LOC100063542,EIF4B,SOAT2,LOC100063712,SPRYD3,AAAS,LOC100062093,ITGB7,CSAD,IGFBP-6,LOC100063570,LOC100063511,KRT79,LOC100063376,KRT77,LOC100063302,LOC100146152,KRT73,KRT71,LOC100061595,LOC100061458,LOC100061422,LOC100146362,LOC100062875,KRT80,LOC100062689
06	69280891	70385588	1104698	4	1	KRT86,LOC100062930,LOC100061487,LOC100062757,LOC100061525,LOC100063215,LOC100063410,LOC100061828,TENC1,LOC100062061,LOC100062026,ESPL1,LOC100062123,KRT4,LOC100061693,KRT5,KRT7,LOC100062985,KRT82,LOC100146665,LOC100061560,LOC100063179,LOC100063342,LOC100061764,LOC100147555,LOC100063542,EIF4B,SOAT2,LOC100063712,SPRYD3,SP7,AMHR2,LOC100629527,AAAS,LOC100062093,ITGB7,CSAD,IGFBP-6,LOC100063570,LOC100063511,KRT79,LOC100063376,KRT77,LOC100063302,LOC100146152,KRT73,KRT71,LOC100061595,LOC100061458,LOC100061422,LOC100146362,LOC100062875,KRT80
06	71872446	72101344	228899	0	1	LOC100053971,LOC100054452,LOC100054400,LOC100630091,LOC100054497,LOC100054585,LOC100050555,LOC100054214,LOC100054306,LOC100054063,LOC100054671,LOC100054771,LOC100146580,LOC100054626,LOC100054539,LOC100054354,LOC100054256,LOC100054118
06	71872446	72493903	621458	1	1	LOC100053971,LOC100054452,LOC100054400,LOC100630091,LOC100050632,LOC100055222,LOC100055352,LOC100055645,LOC100055600,LOC100055986,LOC100055946,LOC100055778,LOC100146480,LOC100054497,LOC100054585,LOC100050555,LOC100054214,LOC100054306,LOC100054063,LOC100054671,LOC100147637,LOC100054771,LOC100055272,LOC100146474,LOC100055559,LOC100055434,LOC100055688,LOC100055866,LOC100055818,LOC100055734,LOC100050712,LOC100055520,LOC100147260,LOC100055477,LOC100055308,LOC100055176,LOC100055082,LOC100055039,LOC100054995,LOC100054952,LOC100054904,LOC100054856,LOC100054816,LOC100146580,LOC100054626,LOC100054539,LOC100054354,LOC100054256,LOC100054118
06	71872446	72603288	730843	1	1	LOC100053971,LOC100054452,LOC100054400,LOC100630091,LOC100050632,LOC100055222,LOC100055352,LOC100055645,LOC100055600,LOC100055986,LOC100055946,LOC100055778,LOC100146480,LOC100054497,LOC100054585,LOC100050555,LOC100054214,LOC100054306,LOC100054063,LOC100054671,LOC100147637,LOC100054771,LOC100055272,LOC100146474,LOC100055559,LOC100055434,LOC100055688,LOC100056234,LOC100056025,LOC100056186,LOC100630654,LOC100056100,LOC100055866,LOC100055818,LOC100055734,LOC100050712,LOC100055520,LOC100147260,LOC100055477,LOC100055308,LOC100055176,LOC100055082,LOC100055039,LOC100054995,LOC100054952,LOC100054904,LOC100054856,LOC100054816,LOC100146580,LOC100054626,LOC100054539,LOC100054354,LOC100054256,LOC100054118
06	72032729	72101344	68616	0,1	11,2	LOC100630091,LOC100054585,LOC100050555,LOC100054671,LOC100054771,LOC100146580,LOC100054626
06	72032729	72127862	95134	1	4	LOC100630091,LOC100054585,LOC100050555,LOC100054671,LOC100054771,LOC100054904,LOC100054856,LOC100054816,LOC100146580,LOC100054626
06	72032729	72423001	390273	1	1	LOC100630091,LOC100050632,LOC100055222,LOC100055352,LOC100055645,LOC100055600,LOC100055778,LOC100054585,LOC100050555,LOC100054671,LOC100147637,LOC100054771,LOC100055272,LOC100146474,LOC100055559,LOC100055434,LOC100055688,LOC100055734,LOC100050712,LOC100055520,LOC100147260,LOC100055477,LOC100055308,LOC100055176,LOC100055082,LOC100055039,LOC100054995,LOC100054952,LOC100054904,LOC100054856,LOC100054816,LOC100146580,LOC100054626
06	72032729	72485833	453105	1	6	LOC100630091,LOC100050632,LOC100055222,LOC100055352,LOC100055645,LOC100055600,LOC100055946,LOC100055778,LOC100146480,LOC100054585,LOC100050555,LOC100054671,LOC100147637,LOC100054771,LOC100055272,LOC100146474,LOC100055559,LOC100055434,LOC100055688,LOC100055866,LOC100055818,LOC100055734,LOC100050712,LOC100055520,LOC100147260,LOC100055477,LOC100055308,LOC100055176,LOC100055082,LOC100055039,LOC100054995,LOC100054952,LOC100054904,LOC100054856,LOC100054816,LOC100146580,LOC100054626
06	72032729	72493903	461175	0,1,3	5,46,1	LOC100630091,LOC100050632,LOC100055222,LOC100055352,LOC100055645,LOC100055600,LOC100055986,LOC100055946,LOC100055778,LOC100146480,LOC100054585,LOC100050555,LOC100054671,LOC100147637,LOC100054771,LOC100055272,LOC100146474,LOC100055559,LOC100055434,LOC100055688,LOC100055866,LOC100055818,LOC100055734,LOC100050712,LOC100055520,LOC100147260,LOC100055477,LOC100055308,LOC100055176,LOC100055082,LOC100055039,LOC100054995,LOC100054952,LOC100054904,LOC100054856,LOC100054816,LOC100146580,LOC100054626

Chrom	Start	End	Size	Cn	Samples (n)	Genes
06	72032729	72603288	570560	1	2	LOC100630091,LOC100050632,LOC100055222,LOC100055352,LOC100055645,LOC100055600,LOC100055986,LOC100055946,LOC100055778,LOC100146480,LOC100054585,LOC100050555,LOC100054671,LOC100147637,LOC100054771,LOC100055272,LOC100146474,LOC100055559,LOC100055434,LOC100055688,LOC100056234,LOC100056025,LOC100056186,LOC100630654,LOC100056100,LOC100055866,LOC100055818,LOC100055734,LOC100050712,LOC100055520,LOC100147260,LOC100055477,LOC100055308,LOC100055176,LOC100055082,LOC100055039,LOC100054995,LOC100054952,LOC100054904,LOC100054856,LOC100054816,LOC100146580,LOC100054626
06	72032729	72607543	574815	1	2	LOC100630091,LOC100050632,LOC100055222,LOC100055352,LOC100055645,LOC100055600,LOC100055986,LOC100055946,LOC100055778,LOC100146480,LOC100054585,LOC100050555,LOC100054671,LOC100147637,LOC100054771,LOC100055272,LOC100146474,LOC100055559,LOC100055434,LOC100055688,LOC100056234,LOC100056025,LOC100056186,LOC100630654,LOC100056100,LOC100055866,LOC100055818,LOC100055734,LOC100050712,LOC100055520,LOC100147260,LOC100055477,LOC100055308,LOC100055176,LOC100055082,LOC100055039,LOC100054995,LOC100054952,LOC100054904,LOC100054856,LOC100054816,LOC100146580,LOC100054626
06	72032729	72646366	613638	1	1	LOC100630091,LOC100050632,LOC100055222,LOC100055352,LOC100055645,LOC100055600,LOC100055986,LOC100146967,LOC100055946,LOC100056394,LOC100146670,LOC100055778,LOC100146480,LOC100054585,LOC100050555,LOC100054671,LOC100147637,LOC100054771,LOC100055272,LOC100146474,LOC100055559,LOC100055434,LOC100055688,LOC100056234,LOC100056025,LOC100056355,LOC100056186,LOC100630654,LOC100056100,LOC100055866,LOC100055818,LOC100055734,LOC100050712,LOC100055520,LOC100147260,LOC100055477,LOC100055308,LOC100055176,LOC100055082,LOC100055039,LOC100054995,LOC100054952,LOC100054904,LOC100054856,LOC100054816,LOC100146580,LOC100054626
06	72032729	72655562	622834	1	1	LOC100630091,LOC100050632,LOC100055222,LOC100055352,LOC100055645,LOC100055600,LOC100055986,LOC100146967,LOC100055946,LOC100056394,LOC100146670,LOC100055778,LOC100146480,LOC100054585,LOC100050555,LOC100054671,LOC100147637,LOC100054771,LOC100055272,LOC100146474,LOC100055559,LOC100055434,LOC100055688,LOC100056234,LOC100056025,LOC100056438,LOC100056355,LOC100056186,LOC100630654,LOC100056100,LOC100055866,LOC100055818,LOC100055734,LOC100050712,LOC100055520,LOC100147260,LOC100055477,LOC100055308,LOC100055176,LOC100055082,LOC100055039,LOC100054995,LOC100054952,LOC100054904,LOC100054856,LOC100054816,LOC100146580,LOC100054626
06	72050269	72493903	443635	1	2	LOC100630091,LOC100050632,LOC100055222,LOC100055352,LOC100055645,LOC100055600,LOC100055986,LOC100055946,LOC100055778,LOC100146480,LOC100050555,LOC100054671,LOC100147637,LOC100054771,LOC100055272,LOC100146474,LOC100055559,LOC100055434,LOC100055688,LOC100055866,LOC100055818,LOC100055734,LOC100050712,LOC100055520,LOC100147260,LOC100055477,LOC100055308,LOC100055176,LOC100055082,LOC100055039,LOC100054995,LOC100054952,LOC100054904,LOC100054856,LOC100054816,LOC100146580
06	72325156	72485833	160678	0	1	LOC100055645,LOC100055600,LOC100055946,LOC100055778,LOC100146480,LOC100055559,LOC100055688,LOC100055866,LOC100055818,LOC100055734,LOC100050712,LOC100055520,LOC100147260
06	72325156	72493903	168748	0,1	12,4	LOC100055645,LOC100055600,LOC100055986,LOC100055946,LOC100055778,LOC100146480,LOC100055559,LOC100055688,LOC100055866,LOC100055818,LOC100055734,LOC100050712,LOC100055520,LOC100147260
06	72325156	72607543	282388	1	1	LOC100055645,LOC100055600,LOC100055986,LOC100055946,LOC100055778,LOC100146480,LOC100055559,LOC100055688,LOC100056234,LOC100056025,LOC100056186,LOC100630654,LOC100056100,LOC100055866,LOC100055818,LOC100055734,LOC100050712,LOC100055520,LOC100147260
06	72422847	72493903	71057	3	1	LOC100055986,LOC100055946,LOC100146480,LOC100055866,LOC100055818
06	73072905	73412971	340067	1	1	LOC100057669,LOC100057893,LOC100057944,LOC100058303,LOC100051162,LOC100058635,LOC100058428,LOC100058516,LOC100057754,LOC100057803,LOC100057708,LOC100058076,LOC100057988,LOC100058345,LOC100058592,LOC100051089,LOC100058554,LOC100058471,LOC100058384,LOC100058210,LOC100058122,LOC100058035,LOC100057844,LOC100051018,LOC100146966
07	620	435683	435064	1	1	LOC100068855,IRF4,TRNAN-GUU,LOC100068840,LOC100068825,TRNAF-GAA,STK11,LOC100147613,PTBP1,LOC100065431
07	620	589331	588712	1	1	LOC100068855,IRF4,TRNAN-GUU,LOC100068840,LOC100146835,TCF3,LOC100147519,MIR1905C,LOC100629298,LOC100060547,LOC100060620,LOC100146126,LOC100068825,TRNAF-GAA,STK11,LOC100147613,PTBP1,LOC100069627,LOC100065431
07	620	788386	787767	4	1	ATP8B3,LOC100068660,LOC100068855,LOC100068675,IRF4,TRNAN-GUU,LOC100068840,LOC100146835,REXO1,LOC100146322,TCF3,LOC100147519,MIR1905C,LOC100629298,LOC100060547,LOC100060620,LOC100146126,LOC100068825,TRNAF-GAA,STK11,LOC100147613,PTBP1,LOC100069627,LOC100065431
07	620	1056097	1055478	1	1	ATP8B3,LOC100068660,LOC100068855,LOC100068675,DOT1L,IRF4,TRNAN-GUU,LOC100068840,LOC100146835,REXO1,LOC100147038,LOC100068440,LOC100068522,LOC100068453,LOC100068599,LOC100146322,TCF3,LOC100147519,MIR1905C,LOC100629298,LOC100060547,LOC100060620,LOC100146126,LOC100068825,TRNAF-GAA,STK11,LOC100147613,PTBP1,LOC100069627,LOC100146117,LOC100065431
07	254594	518487	263894	1	1	LOC100068855,IRF4,TRNAN-GUU,LOC100068840,LOC100146835,LOC100629298,LOC100060547,LOC100060620,LOC100146126,LOC100068825,TRNAF-GAA,STK11
07	1846745	2845681	998937	1	1	LOC100147429,ATCAY,LOC100061536,ANKRD24,LOC100067327,LOC100147319,LOC100146445,LOC100630136,LOC100063190,LOC100063125,LOC100061437,LOC100063061,FSD1,LOC100146444,LOC100146122,LOC100062552,LOC100146236,LOC100146738,LOC100062939,LOC100147610,LOC100062073,LOC100063153,PIAS4,DAPK3,LOC100146548,TJP3,LOC100146733,LOC100062859,LOC100062595,DPP9,FZR1
07	1846745	2954763	1108019	1	1	LOC100147429,ATCAY,LOC100061536,ANKRD24,LOC100067327,LOC100147319,LOC100146445,LOC100630136,LOC100063190,LOC100063125,LOC100061437,LOC100063061,FSD1,MIR7-2,LOC100146226,LOC100146444,LOC100146122,TRNAG-UCC,LOC100062552,LOC100146236,LOC100146738,LOC100062939,LOC100147610,LOC100062073,LOC100063153,PIAS4,DAPK3,LOC100146548,TJP3,LOC100146733,LOC100062859,LOC100062595,DPP9,FZR1
07	1969080	2432657	463578	1	1	LOC100147429,ATCAY,LOC100061536,LOC100147319,LOC100146445,LOC100630136,LOC100061437,PIAS4,DAPK3,LOC100146548,TJP3,LOC100146733,FZR1

Chrom	Start	End	Size	Cn	Samples (n)	Genes
07	2250740	2432657	181918	1	1	ATCAY,LOC100061536,LOC100146445,LOC100630136,LOC100061437,PIAS4,DAPK3,LOC100146548,TJP3
07	2413892	2845681	431790	4	1	ANKRD24,LOC100063190,LOC100063125,LOC100063061,FSD1,LOC100146444,LOC100146122,LOC100062552,LOC100146236,LOC100146738,LOC100062939,LOC100147610,LOC100062073,LOC100063153,PIAS4,LOC100062859,LOC100062595,DPP9
07	2602672	3369076	766405	1	1	FSD1,TICAM1,MIR7-2,LOC100146226,LOC100146444,LOC100146122,UHRF1,LOC100062036,TRNAG-UCC,LOC100062552,LOC100146236,LOC100146738,LOC100062939,LOC100147610,LOC100062859,ARRDC5,LOC100062103,LOC100062595,DPP9
07	8475870	9412777	936908	1	1	LOC100630115
07	9818064	10363881	545818	1	1	CNTN5
07	17864036	17926192	62157	4	2	DDX10
07	20587454	20687920	100467	1	1	IL18,LOC100062062,LOC100629334,LOC100062094,LOC100070477,DLAT
07	27228729	27825917	597189	4	1	TRIM29,LOC100071480,LOC100063411,LOC100071489,LOC100071454
07	31406445	31520626	114182	1	1	LOC100071915,LOC100071904,LOC100071894,LOC100071910,LOC100630881
07	31406445	31520977	114533	0,1	1,2	LOC100071915,LOC100071904,LOC100071894,LOC100071910,LOC100630881
07	31406445	31529855	123411	1	3	LOC100071915,LOC100071904,LOC100071894,LOC100071920,LOC100071910,LOC100630881
07	31414203	31520977	106775	1	2	LOC100071915,LOC100071904,LOC100071910,LOC100630881
07	47972002	50006770	2034769	4	1	LOC100146395,ZNF709,LOC100146392,PRKCSH,LOC100629291,CARM1,KRI1,LOC100058341,LOC100055350,LOC100055431,LOC100055392,RAVER1,DOCK6,LOC100056621,LOC100629919,LOC100057022,LOC100146999,LOC100057105,LOC100057224,LOC100629179,LOC100057187,LOC100057389,LOC100630733,LOC100054992,LOC100057430,LOC100057750,ATG4D,SLC44A2,LOC100147184,LOC100055218,LOC100055305,LOC100055863,P2RY11,LOC100058249,LOC100058468,QTRT1,LOC100056232,LOC100147388,LOC100058712,LOC100058671,LOC100058589,LOC100058425,LOC100058299,LOC100058207,LOC100146897,LOC100058074,LOC100146182,LOC100055173,LOC100147080,LOC100055128,LOC100055078,ILF3,LOC100057627,LOC100146288,LOC100054949,LOC100054901,LOC100054853,LOC100057146,LOC100147390,LOC100056901,ECSIT,LOC100054768,LOC100056704,LOC100630748,LOC100056435,LOC100055983
07	48651819	49701722	1049904	1	1	ZNF709,LOC100146392,PRKCSH,LOC100629291,CARM1,KRI1,RAVER1,DOCK6,LOC100056621,LOC100629919,LOC100057022,LOC100146999,LOC100057105,LOC100057224,LOC100629179,LOC100057187,LOC100057389,LOC100054992,LOC100057430,LOC100057750,ATG4D,SLC44A2,LOC100147184,LOC100055218,LOC100055305,QTRT1,LOC100058207,LOC100146897,LOC100058074,LOC100146182,LOC100055173,LOC100147080,LOC100055128,LOC100055078,ILF3,LOC100057627,LOC100146288,LOC100054949,LOC100054901,LOC100054853,LOC100057146,LOC100147390,LOC100056901,ECSIT,LOC100054768,LOC100056704,LOC100630748
07	48789377	49927901	1138525	1	1	LOC100146392,PRKCSH,LOC100629291,CARM1,KRI1,LOC100058341,LOC100055350,RAVER1,DOCK6,LOC100056621,LOC100057022,LOC100146999,LOC100057105,LOC100057224,LOC100629179,LOC100057187,LOC100057389,LOC100054992,LOC100057430,LOC100057750,ATG4D,SLC44A2,LOC100147184,LOC100055218,LOC100055305,P2RY11,LOC100058249,LOC100058468,QTRT1,LOC100147388,LOC100058589,LOC100058425,LOC100058299,LOC100058207,LOC100146897,LOC100058074,LOC100146182,LOC100055173,LOC100147080,LOC100055128,LOC100055078,ILF3,LOC100057627,LOC100146288,LOC100054949,LOC100054901,LOC100054853,LOC100057146,LOC100147390,LOC100056901,ECSIT,LOC100054768,LOC100056704
07	49199227	49927901	728675	1	1	LOC100629291,CARM1,KRI1,LOC100058341,LOC100055350,RAVER1,LOC100054992,LOC100057430,LOC100057750,ATG4D,SLC44A2,LOC100147184,LOC100055218,LOC100055305,P2RY11,LOC100058249,LOC100058468,QTRT1,LOC100147388,LOC100058589,LOC100058425,LOC100058299,LOC100058207,LOC100146897,LOC100058074,LOC100146182,LOC100055173,LOC100147080,LOC100055128,LOC100055078,ILF3,LOC100057627,LOC100146288
07	49701722	50006770	305049	1	1	LOC100058341,LOC100055350,LOC100055431,LOC100055392,P2RY11,LOC100058249,LOC100058468,LOC100147388,LOC100058712,LOC100058671,LOC100058589,LOC100058425,LOC100058299
07	49970817	50006770	35954	4	2	LOC100055431,LOC100055392,LOC100058712,LOC100058671
07	49970817	50202506	231690	3	1	LOC100055431,LOC100055392,LOC100059134,ZNF846,LOC100058712,LOC100059098,LOC100058982,LOC100058671
07	52328145	52677786	349642	4	1	LOC100146282,LOC100063843,LOC100064357,LOC100064235,LOC100064296,ZNF558,LOC100063871,LOC100063717,LOC100629766
07	52610482	52654017	43536	3	1	LOC100146282,LOC100064296
07	52610482	52677786	67305	3	1	LOC100146282,LOC100064357,LOC100064296
07	52610482	52765072	154591	3	1	LOC100146282,LOC100064357,LOC100055731,LOC100064595,LOC100064420,LOC100064296
07	52654017	52677786	23770	3	1	LOC100064357
07	52654017	52765072	111056	3	2	LOC100064357,LOC100055731,LOC100064595,LOC100064420
07	56458055	56540470	82416	3	2	LOC100059915,LOC100050258,NAALAD2
07	56458055	56549568	91514	3	1	LOC100059915,LOC100050258,NAALAD2
07	67450899	70565957	3115059	3	1	LOC100051651,TRNAP-UGG,LOC100064387,MAP6,LOC100064184,GDPD5,LOC100064740,LOC100051780,XRRA1,CHRDL2,NEU3,LOC100052085,PGM2L1,LOC100052144,LOC100065565,PAAF1,LOC100065672,LOC100064817,P2RY2,LOC100064445,LOC100063749,ARRB1,LOC100630860,SLCO2B1,RNF169,LOC100065518,LOC100065545,C2CD3,ARHGEF17,LOC100052387,LOC100629588,LOC100052206,LOC100065447,LOC100065317,LOC100065260,LOC100065199,LOC100065109,POLD3,LOC100051902,LOC100064846,LOC100064767,MIR326,LOC100064622,SERPINH1,UVRAG,TRNAP-AGG,PRKRIR,LOC100063671
07	70677039	70782467	105429	1	1	FCHSD2

Chrom	Start	End	Size	Cn	Samples (n)	Genes
07	70677039	71547591	870553	1	1	LOC100052674,LOC100066065,LOC100629820,RNF121,LOC100066133,PDE2A,ARAP1,LOC100146693,LOC100052446,LOC100066161,NUMA1,LOC100066037,LOC100052779,LOC100066106,LOC100052723,LOC100066084,CLPB,MIR139,LOC100052563,FCHSD2
07	73083306	73197149	113844	0,1	1,9	LOC100146503,LOC100067901,LOC100067972,LOC100067873,LOC100068010,LOC100067992,LOC100067950,LOC100067928,LOC100147186,LOC100146803
07	83977865	87915272	3937408	3	1	LOC100071719,PIK3C2A,ABCC8,LOC100629169,SAAL1,LOC100629928,LOC100629188,HPS5,SERGEF,LDHA,LOC100057256,UEVLD,LOC100071946,E2F8,LOC100629227,MYOD1,SOX6,SAA1,LOC100146998,KCNJ11,LOC100056936,LOC100071780,SPTY2D1,LOC100057301,ZDHHC13,NAV2,LOC100071950,LOC100071938,PTPN5,LOC100071921,TSG101,LOC100057054,GTF2H1,LOC100056819,SAA,LOC100056698,TPH1,KCNC1,LOC100071797,NUCB2,LOC100056386,LOC100630838,PLEKHA7
07	98111795	98426293	314499	1	1	CCDC73,LOC100072747,EIF3M
08	133552	1347166	1213615	3	1	SMPD4,LOC100049858,LOC100630369,P2RX6,LOC100052447,LOC100146247,LOC100053038,LOC100052885,LOC100050121,UPB1,SUSD2,LOC100147354,LOC100052938,GGT1,GGT5,LOC100053375,LOC100053226,LOC100050055,ADORA2A,LOC100049992,LOC100052724,LOC100052564,LOC100052334,LOC100051298,LOC100051442,LOC100051652,LOC100051903,LOC100052145,KLHL22
08	133552	3726860	3593309	4	1	LOC100146559,LOC100147157,LOC100629490,LOC100146659,LOC100055979,SLC7A4,LOC100629278,LOC100629565,LOC100629759,LOC100054210,LOC100629174,LOC100056657,LOC100629605,RIMBP3,LOC100057341,MIR130B,LOC100057701,LOC100051078,PPM1F,LOC100147447,LOC100054068,LOC100146461,SMPD4,LOC100049858,LOC100630369,P2RX6,LOC100052447,LOC100146247,LOC100053038,LOC100052885,LOC100050121,UPB1,SUSD2,LOC100147354,LOC100053419,SLC2A11,LOC100053561,LOC100630766,LOC100053714,LOC100054110,LOC100146774,LOC100056784,LOC100630819,LOC100050322,LOC100053914,LOC100056897,TOP3B,LOC100057302,LOC100146658,HIC2,MIR301B,SERPIND1,LOC100630004,LOC100050930,AIFM3,LZTR1,PI4KA,LOC100629255,SMARCB1,LOC100056349,LOC100054251,LOC100056225,LOC100146961,LOC100065808,LOC100055901,LOC100146352,LOC100050544,LOC100629342,LOC100629299,LOC100065759,LOC100630137,LOC100630898,LOC100052938,GGT1,GGT5,LOC100630122,LOC100050855,ZNF70,LOC100056615,MMP11,LOC100056480,LOC100056265,LOC100629319,LOC100054057,LOC100630726,LOC100050390,LOC100053468,LOC100053375,LOC100053226,LOC100050055,ADORA2A,LOC100049992,LOC100052724,LOC100052564,LOC100052334,LOC100051298,LOC100051442,LOC100051652,LOC100051903,LOC100052145,KLHL22,LOC100053618
08	2213144	2712678	499535	3	1	LOC100147157,LOC100629490,LOC100146659,LOC100055979,SLC7A4,LOC100629565,LOC100629605,SMARCB1,LOC100056349,LOC100056225,LOC100146961,LOC100055901,LOC100050544,MMP11,LOC100056480,LOC100056265
08	2213144	3216080	1002937	3	1	LOC100147157,LOC100629490,LOC100146659,LOC100055979,SLC7A4,LOC100629565,LOC100629759,LOC100056657,LOC100629605,LOC100147447,LOC100054068,LOC100146461,LOC100056784,LOC100056897,SERPIND1,LOC100050930,AIFM3,LZTR1,PI4KA,SMARCB1,LOC100056349,LOC100056225,LOC100146961,LOC100055901,LOC100050544,LOC100050855,ZNF70,LOC100056615,MMP11,LOC100056480,LOC100056265
08	2213144	3726860	1513717	3	1	LOC100147157,LOC100629490,LOC100146659,LOC100055979,SLC7A4,LOC100629565,LOC100629759,LOC100056657,LOC100629605,RIMBP3,LOC100057341,MIR130B,LOC100057701,LOC100051078,PPM1F,LOC100147447,LOC100054068,LOC100146461,LOC100056784,LOC100056897,TOP3B,LOC100057302,LOC100146658,HIC2,MIR301B,SERPIND1,LOC100630004,LOC100050930,AIFM3,LZTR1,PI4KA,SMARCB1,LOC100056349,LOC100056225,LOC100146961,LOC100055901,LOC100050544,LOC100630137,LOC100630122,LOC100050855,ZNF70,LOC100056615,MMP11,LOC100056480,LOC100056265
08	2213144	5149554	2936411	3	1	LOC100147157,LOC100629490,LOC100146659,LOC100055979,SLC7A4,LOC100629565,LOC100629759,LOC100056657,LOC100629605,RIMBP3,LOC100057341,MIR130B,LOC100057701,LOC100051078,LOC100630286,LOC100059976,LOC100070802,LOC100630440,LOC100630335,PPM1F,LOC100630460,LOC100630618,LOC100147447,LOC100054068,LOC100146461,LOC100056784,LOC100147255,SLC5A1,PRAME,LOC100060608,LOC100058200,LOC100630600,LOC100060228,LOC100146359,LOC100062472,LOC100630370,LOC100056897,LOC100147544,LOC100630192,TOP3B,LOC100057302,LOC100146658,HIC2,MIR301B,SERPIND1,LOC100630004,LOC100050930,AIFM3,LZTR1,PI4KA,SMARCB1,LOC100056349,LOC100056225,LOC100146961,LOC100055901,LOC100050544,LOC100630176,LOC100630137,LOC100630575,LOC100146858,LOC100630122,LOC100050855,ZNF70,LOC100056615,MMP11,LOC100056480,LOC100056265
08	2489368	3821757	1332390	3	2	SLC7A4,LOC100629565,LOC100629759,LOC100056657,LOC100629605,RIMBP3,LOC100057341,MIR130B,LOC100057701,LOC100051078,LOC100630286,LOC100059976,PPM1F,LOC100147447,LOC100054068,LOC100146461,LOC100056784,LOC100056897,LOC100630192,TOP3B,LOC100057302,LOC100146658,HIC2,MIR301B,SERPIND1,LOC100630004,LOC100050930,AIFM3,LZTR1,PI4KA,SMARCB1,LOC100056349,LOC100056225,LOC100630176,LOC100630137,LOC100630122,LOC100050855,ZNF70,LOC100056615,MMP11,LOC100056480,LOC100056265
08	2897484	3726860	829377	4	2	RIMBP3,LOC100057341,MIR130B,LOC100057701,LOC100051078,PPM1F,LOC100147447,LOC100054068,LOC100056897,TOP3B,LOC100057302,LOC100146658,HIC2,MIR301B,SERPIND1,LOC100630004,LOC100050930,AIFM3,LZTR1,PI4KA,LOC100630137,LOC100630122,LOC100050855
08	2897484	3821757	924274	3	2	RIMBP3,LOC100057341,MIR130B,LOC100057701,LOC100051078,LOC100630286,LOC100059976,PPM1F,LOC100147447,LOC100054068,LOC100056897,LOC100630192,TOP3B,LOC100057302,LOC100146658,HIC2,MIR301B,SERPIND1,LOC100630004,LOC100050930,AIFM3,LZTR1,PI4KA,LOC100630176,LOC100630137,LOC100630122,LOC100050855
08	2897484	4430473	1532990	4	1	RIMBP3,LOC100057341,MIR130B,LOC100057701,LOC100051078,LOC100630286,LOC100059976,LOC100070802,LOC100630440,LOC100630335,PPM1F,LOC100630460,LOC100147447,LOC100054068,LOC100147255,LOC100060608,LOC100630600,LOC100060228,LOC100146359,LOC100062472,LOC100630370,LOC100056897,LOC100147544,LOC100630192,TOP3B,LOC100057302,LOC100146658,HIC2,MIR301B,SERPIND1,LOC100630004,LOC100050930,AIFM3,LZTR1,PI4KA,LOC100630176,LOC100630137,LOC100630575,LOC100146858,LOC100630122,LOC100050855
08	2897484	4745101	1847618	3,4	3,1	RIMBP3,LOC100057341,MIR130B,LOC100057701,LOC100051078,LOC100630286,LOC100059976,LOC100070802,LOC100630440,LOC100630335,PPM1F,LOC100630460,LOC100147447,LOC100054068,LOC100147255,PRAME,LOC100060608,LOC100630600,LOC100060228,LOC100146359,LOC100062472,LOC100630370,LOC100056897,LOC100147544,LOC100630192,TOP3B,LOC100057302,LOC100146658,HIC2,MIR301B,SERPIND1,LOC100630004,LOC100050930,AIFM3,LZTR1,PI4KA,LOC100630176,LOC100630137,LOC100630575,LOC100146858,LOC100630122,LOC100050855

Chrom	Start	End	Size	Cn	Samples (n)	Genes
08	3565276	4183178	617903	3	2	LOC100630286,LOC100059976,LOC100070802,LOC100630440,LOC100630335,PPM1F,LOC100630460,LOC100147255,LOC100060608,LOC100630600,LOC100060228,LOC100146359,LOC100062472,LOC100630370,LOC100147544,LOC100630192,TOP3B,LOC100630176,LOC100630137,LOC100630575,LOC100146858,LOC100630122
08	3726675	3821757	95083	3	4	LOC100630286,LOC100059976,LOC100630192,LOC100630176
08	3726675	4183178	456504	3	1	LOC100630286,LOC100059976,LOC100070802,LOC100630440,LOC100630335,LOC100630460,LOC100147255,LOC100060608,LOC100630600,LOC100060228,LOC100146359,LOC100062472,LOC100630370,LOC100147544,LOC100630192,LOC100630176,LOC100630575,LOC100146858
08	3726675	4430473	703799	3	1	LOC100630286,LOC100059976,LOC100070802,LOC100630440,LOC100630335,LOC100630460,LOC100147255,LOC100060608,LOC100630600,LOC100060228,LOC100146359,LOC100062472,LOC100630370,LOC100147544,LOC100630192,LOC100630176,LOC100630575,LOC100146858
08	3726675	4539344	812670	3	1	LOC100630286,LOC100059976,LOC100070802,LOC100630440,LOC100630335,LOC100630460,LOC100147255,LOC100060608,LOC100630600,LOC100060228,LOC100146359,LOC100062472,LOC100630370,LOC100147544,LOC100630192,LOC100630176,LOC100630575,LOC100146858
08	3726675	4559972	833298	3	1	LOC100630286,LOC100059976,LOC100070802,LOC100630440,LOC100630335,LOC100630460,LOC100147255,LOC100060608,LOC100630600,LOC100060228,LOC100146359,LOC100062472,LOC100630370,LOC100147544,LOC100630192,LOC100630176,LOC100630575,LOC100146858
08	3726675	4579477	852803	3	1	LOC100630286,LOC100059976,LOC100070802,LOC100630440,LOC100630335,LOC100630460,LOC100147255,LOC100060608,LOC100630600,LOC100060228,LOC100146359,LOC100062472,LOC100630370,LOC100147544,LOC100630192,LOC100630176,LOC100630575,LOC100146858
08	4183178	4430473	247296	1	1	LOC100062472
08	4280605	4430473	149869	0,1	1,6	LOC100062472
08	4391896	4430473	38578	1	2	LOC100062472
08	4391896	4559972	168077	1	1	LOC100062472
08	4391896	4579477	187582	3	1	LOC100062472
08	4430473	4618457	187985	1	2	LOC100062472
08	4430473	4621044	190572	1	10	LOC100062472
08	4430473	4646812	216340	1	1	LOC100062472
08	4537919	4559972	22054	4	1	LOC100062472
08	4537919	4618457	80539	1	2	LOC100062472
08	4537919	4621044	83126	0,1	3,12	LOC100062472
08	4537919	4646812	108894	1	1	LOC100062472
08	4559972	4621044	61073	1	2	LOC100062472
08	5025956	6545985	1520030	3	1	DEPDC5,LOC100063052,LOC100063118,LOC100063088,INPP5J,OSBP2,LOC100063545,SMTN,EIF4ENIF1,LOC100063573,LOC100063639,SEC14L4,LOC100063781,PES1,LOC100058667,MORC2,LOC100630784,LOC100058463,LOC100063270,DRG1,LIMK2,LOC100062878,SLC5A1,PRR14L,LOC100058200,LOC100146357,SELM,PLA2G3,PATZ1
08	12227264	12580862	353599	4	1	CORO1C,LOC100066847,SART3,LOC100066887,SELPLG,FICD
08	12327800	12623847	296048	4	1	LOC100066847,SART3,LOC100066887,LOC100059916,SELPLG,FICD
08	14392687	16808026	2415340	3	1	TAOK3,VSIG10,RFC5,LOC100055124,LOC100629981,LOC100055343,LOC100050779,NOS1,LOC100050856,FBXO21,LOC100051008,KSR2,LOC100055213,LOC100054898,RNFT2,LOC100054163,LOC100054578
08	16085130	16171233	86104	0	6	NOS1
08	24030822	24188533	157712	4	2	LOC100146720
08	24030822	24411688	380867	4	1	LOC100061529,LOC100146720
08	28260573	28814835	554263	4	1	GPR133,LOC100062511
08	28574183	29419499	845317	1	1	SFSWAP,LOC100062511,EP400,NOC4L,LOC100061962,MMP17,ULK1,DDX51,LOC100062278
08	28574183	29449442	875260	1	1	SFSWAP,LOC100062511,EP400,NOC4L,LOC100061962,MMP17,ULK1,DDX51,LOC100062278
08	28730876	29449442	718567	1	1	SFSWAP,LOC100062511,EP400,NOC4L,LOC100061962,MMP17,ULK1,DDX51,LOC100062278
08	37207338	37629004	421667	1	1	LOC100630847,CEP192,LOC100050392,CEP76,LOC100058028,LOC100057795
08	41758072	41869404	111333	3	1	
08	47584249	47958740	374492	1	1	LOC100051844,TAF4B,LOC100064417
08	51065161	51853981	788821	1	1	LOC100065708,LOC100630216
08	64161502	64316647	155146	1	1	SETBP1
08	65338188	65348845	10658	1	1	
08	81506440	81550319	43880	1	1	
08	85645153	85699984	54832	1	1	SOCS6
08	86092029	86506742	414714	1	1	
08	86092029	86910236	818208	1	1	
08	93030075	93398026	367952	4	1	LOC100065346,NFATC1
09	11911481	12146646	235166	1	1	

Chrom	Start	End	Size	Cn	Samples (n)	Genes
09	29889627	29892897	3271	0	20	
09	31489471	31853289	363819	1	1	PXDNL
09	31548212	31601825	53614	1	1	PXDNL
09	31574454	31574969	516	1	3	PXDNL
09	33119854	33223660	103807	1	1	SNTG1
09	33119854	33295888	176035	3	1	SNTG1
09	44946791	45279882	333092	1	1	VPS13B,LOC100060745
09	44946791	45518773	571983	1	3	VPS13B,LOC100060745
09	44946791	46122801	1176011	1	1	RGS22,LOC100630612,RNF19A,FBXO43,VPS13B,LOC100055903,LOC100060745,LOC100055813
09	50803693	50808048	4356	0,1	1,1	
09	50803693	51315697	512005	1	1	OXR1
09	52528450	52647501	119052	1	1	RSPO2
09	52528450	52727466	199017	1	1	EIF3E,RSPO2
09	55890153	56708866	818714	1	1	CSMD3
09	55890153	58126904	2236752	1	1	CSMD3
09	55890153	58398339	2508187	1	1	LOC100056785,CSMD3
09	56125503	56708866	583364	1	1	CSMD3
09	57667896	57759678	91783	3	1	
09	62509239	63222931	713693	3	1	DEPTOR,COL14A1,LOC100066414,DSCC1,TAF2,TRNAK-UUU,ENPP2
09	70971456	71442946	471491	3	1	LOC100068561,LOC100068406,LOC100068503,LOC100068487,ASAP1
09	78784622	80784834	2000213	3	1	PTK2,SLC45A4,MIR151,LOC100058202,TRAPPC9,DENND3,LOC100069651,LOC100058296,EIF2C2,LOC100629742
09	80113000	82511098	2398099	3	1	LOC100630449,LOC100146671,LOC100146864,LOC100065550,SCRIB,LOC100146581,LOC100065805,LOC100065937,LOC100147645,LOC100146968,LOC100066253,LOC100066111,LOC100063645,LOC100066475,LOC100146865,SLC45A4,ZC3H3,DENND3,LOC100069651,TSNARE1,LOC100058296,LOC100066505,LOC100063721,LOC100063680,JRK,LOC100066141,LOC100066193,LOC100146674,LOC100063612,LOC100147366,LOC100065757,LOC100063519,LOC100147640,TSTA3,LOC100629422,LOC100629403,LOC100630161
09	80113000	82631266	2518267	3	1	LOC100630449,LOC100147456,LOC100065320,SPATC1,LOC100146671,LOC100630638,LOC100147064,LOC100146864,LOC100065550,SCRIB,LOC100146581,LOC100065805,LOC100065937,LOC100147645,LOC100146968,LOC100066253,LOC100066111,LOC100063645,LOC100066475,LOC100146865,SLC45A4,ZC3H3,DENND3,LOC100069651,TSNARE1,LOC100058296,LOC100066505,LOC100063721,LOC100063680,JRK,LOC100066141,LOC100066193,LOC100146674,LOC100063612,LOC100147366,LOC100065757,LOC100063519,LOC100147640,GRINA,TSTA3,LOC100629422,LOC100629403,LOC100630161
09	80329751	82511098	2181348	3	1	LOC100630449,LOC100146671,LOC100146864,LOC100065550,SCRIB,LOC100146581,LOC100065805,LOC100065937,LOC100147645,LOC100146968,LOC100066253,LOC100066111,LOC100063645,LOC100066475,LOC100146865,ZC3H3,LOC100069651,TSNARE1,LOC100058296,LOC100066505,LOC100063721,LOC100063680,JRK,LOC100066141,LOC100066193,LOC100146674,LOC100063612,LOC100147366,LOC100065757,LOC100063519,LOC100147640,TSTA3,LOC100629422,LOC100629403,LOC100630161
09	80555535	82095444	1539910	3	1	LOC100065937,LOC100066253,LOC100066111,LOC100063645,LOC100066475,LOC100146865,ZC3H3,TSNARE1,LOC100066505,LOC100063721,LOC100063680,JRK,LOC100066141,LOC100066193,LOC100146674,LOC100063612,LOC100629403,LOC100630161
09	80588672	82631266	2042595	3	1	LOC100630449,LOC100147456,LOC100065320,SPATC1,LOC100146671,LOC100630638,LOC100147064,LOC100146864,LOC100065550,SCRIB,LOC100146581,LOC100065805,LOC100065937,LOC100147645,LOC100146968,LOC100066253,LOC100066111,LOC100063645,LOC100066475,LOC100146865,ZC3H3,TSNARE1,LOC100066505,LOC100063721,LOC100063680,JRK,LOC100066141,LOC100066193,LOC100146674,LOC100063612,LOC100147366,LOC100065757,LOC100063519,LOC100147640,GRINA,TSTA3,LOC100629422,LOC100629403,LOC100630161
09	80613827	82511098	1897272	3	1	LOC100630449,LOC100146671,LOC100146864,LOC100065550,SCRIB,LOC100146581,LOC100065805,LOC100065937,LOC100147645,LOC100146968,LOC100066253,LOC100066111,LOC100063645,LOC100066475,LOC100146865,ZC3H3,TSNARE1,LOC100066505,LOC100063721,LOC100063680,JRK,LOC100066141,LOC100066193,LOC100146674,LOC100063612,LOC100147366,LOC100065757,LOC100063519,LOC100147640,TSTA3,LOC100629422,LOC100629403,LOC100630161
09	80719964	82795695	2075732	3	1	LOC100146668,LOC100630449,LOC100065112,LOC100063419,LOC100065171,LOC100147456,LOC100065320,SPATC1,LOC100146671,LOC100630638,LOC100147064,LOC100146864,LOC100065550,SCRIB,LOC100146581,LOC100065805,LOC100065937,LOC100147645,LOC100146968,LOC100066253,LOC100066111,LOC100063645,LOC100066475,LOC100146865,ZC3H3,LOC100065232,SCX,TSNARE1,LOC100066505,LOC100063721,LOC100063680,JRK,LOC100066141,LOC100066193,LOC100146674,LOC100063612,LOC100147366,LOC100065757,LOC100063519,LOC100147640,GRINA,TSTA3,LOC100065082,LOC100065139,LOC100063382,LOC100629422,LOC100629403,LOC100630161
09	80783889	83494725	2710837	3	1	LOC100064875,LOC100064273,LOC100064332,ZNF251,LOC100064451,LOC100064664,ARHGAP39,LRRC14,CYHR1,LOC100146668,LOC100147459,LOC100064793,LOC100147065,LOC100064823,LOC100063277,ADCK5,FBXL6,LOC100064717,LOC100630449,LOC100065112,LOC100063419,LOC100065171,LOC100147456,LOC100065320,SPATC1,LOC100146671,LOC100630638,LOC100147064,LOC100146864,LOC100065550,SCRIB,LOC100146581,LOC100063350,LOC100065805,LOC100065937,LOC100147645,LOC100146968,LOC100066253,LOC100066111,LOC100063645,LOC100066475,LOC100146865,ZC3H3,LOC100065232,SCX,TSNARE1,LOC100066505,LOC100063721,LOC100063680,JRK,LOC100066141,LOC100066193,LOC100146674,LOC100063612,LOC100147366,LOC100065757,LOC100063519,LOC100147640,GRINA,BOP1,GPR172B,LOC100147359,RECQL4,LOC100064599,LOC100064109,LOC100064163,TSTA3,ZNF250,LOC100063246,LOC100065082,LOC100065139,LOC100063382,LOC100629422,LOC100629403,LOC100630161

Chrom	Start	End	Size	Cn	Samples (n)	Genes
09	80860049	80985923	125875	3	1	
09	81322899	82631266	1308368	3	1	LOC100630449,LOC100147456,LOC100065320,SPATC1,LOC100146671,LOC100630638,LOC100147064,LOC100146864,LOC100065550,SCRIB,LOC100146581,LOC100065805,LOC100065937,LOC100147645,LOC100146968,LOC100066253,LOC100066111,LOC100063645,LOC100066475,LOC100146865,ZC3H3,LOC100066505,LOC100063721,LOC100063680,JRK,LOC100066141,LOC100066193,LOC100146674,LOC100063612,LOC100147366,LOC100065757,LOC100063519,LOC100147640,GRINA,TSTA3,LOC100629422,LOC100629403,LOC100630161
09	81322899	82795695	1472797	3,4	1,1	LOC100146668,LOC100630449,LOC100065112,LOC100063419,LOC100065171,LOC100147456,LOC100065320,SPATC1,LOC100146671,LOC100630638,LOC100147064,LOC100146864,LOC100065550,SCRIB,LOC100146581,LOC100065805,LOC100065937,LOC100147645,LOC100146968,LOC100066253,LOC100066111,LOC100063645,LOC100066475,LOC100146865,ZC3H3,LOC100065232,SCX,LOC100066505,LOC100063721,LOC100063680,JRK,LOC100066141,LOC100066193,LOC100146674,LOC100063612,LOC100147366,LOC100065757,LOC100063519,LOC100147640,GRINA,TSTA3,LOC100065082,LOC100065139,LOC100063382,LOC100629422,LOC100629403,LOC100630161
09	81398267	81690732	292466	4	1	LOC100063645,LOC100066475,LOC100146865,LOC100066505,LOC100063721,LOC100063680,JRK,LOC100063612,LOC100630161
09	81398267	82209483	811217	4	1	LOC100065805,LOC100065937,LOC100147645,LOC100066253,LOC100066111,LOC100063645,LOC100066475,LOC100146865,ZC3H3,LOC100066505,LOC100063721,LOC100063680,JRK,LOC100066141,LOC100066193,LOC100146674,LOC100063612,LOC100147366,LOC100063519,LOC100629403,LOC100630161
09	82340798	82435310	94513	1	1	LOC100146671,LOC100065550,SCRIB,LOC100146968
10	674485	1045694	371210	3	1	LOC100053533
10	674485	1141923	467439	3	1	LOC100053533,LOC100053634
10	674485	1271225	596741	3	4	LOC100053533,LOC100053634
10	782630	854532	71903	1	1	
10	3566834	4669643	1102810	4	1	PEPD,LOC100630820,SLC7A10,LRP3,WDR88,LOC100049940,LOC100056200,LOC100056158,CEP89,SLC7A9,LOC100055997,LOC100629396,LOC100055611,LOC100629343,LOC100629320,DPY19L3,ZNF507
10	3849488	4669643	820156	4	1	PEPD,LOC100630820,SLC7A10,LRP3,WDR88,LOC100049940,LOC100056200,LOC100056158,CEP89,SLC7A9,LOC100055997,LOC100629396,LOC100055611,LOC100629343,LOC100629320
10	4094636	4669643	575008	4	1	PEPD,LOC100630820,SLC7A10,LRP3,WDR88,LOC100049940,LOC100056200,LOC100056158,CEP89,SLC7A9
10	8471996	14618796	6146801	3	1	LOC100069898,LOC100069866,LOC100065329,CD79A,LOC100629122,LOC100147673,ATP1A3,LOC100069742,ZNF574,POU2F2,LOC100065272,LOC100146293,LOC100069693,ETF1,LOC100147683,LOC100066750,LOC100069673,LOC100065240,LOC100065213,LOC100147203,LOC100147106,MEGF8,LOC100069615,LOC100069598,LOC100630773,IL28B,LOC100069585,LOC100069572,LOC100630749,LOC100069549,LOC100064427,LOC100630655,LOC100069522,LOC100065118,LOC100147014,PAF1,LOC100069407,LOC100069314,LOC100630667,LOC100069287,TRNAI-UAU,LOC100069277,LOC100630611,LOC100630516,LOC100630497,LOC100069166,LOC100630429,LOC100630409,LOC100630385,LOC100630359,LOC100065058,LOC100065028,BCKDHA,LOC100064997,LOC100630482,LOC100146820,TGFB1,LOC100064964,HNRNPUL1,AXL,LOC100147675,LOC100068618,LOC100630234,LOC100068603,LOC100147010,LOC100068569,LOC100630152,LOC100068510,LOC100068495,LOC100146413,LOC100146204,CYP2A13,LOC100630054,LOC100068334,LOC100068315,LOC100068295,LOC100064859,LOC100064830,MIA,LOC100068274,LOC100068235,ITPKC,ADCK4,NUMBL,LOC100064750,LOC100630098,LOC100146416,LOC100068145,LOC100068129,LOC100068110,LOC100068085,LOC100068062,PLD3,LOC100068049,AKT2,LOC100146308,MAP3K10,LOC100067940,EQUCABV1R901,ZNF91,LOC100067812,ZNF546,PSMC4,LOC100146404,LOC100064571,LOC100147011,LOC100067694,LOC100067648,LOC100067604,SUPT5H,LOC100064489,PLEKHG2,SAMD4B,LOC100067447,LOC100629870,LOC100067228,PAK4,LOC100066651,LOC100064399,LOC100629402,LOC100146611,HNRNPL,LOC100066427,LOC100064337,LOC100066370,LOC100066343,MAP4K1,LOC100066117,LOC100064192,LOC100064166,LOC100064140,YIF1B,LOC100064080,LOC100629152,LOC100064014,LOC100063981,LOC100063688,WDR87,LOC100063034,ZNF383,LOC100629669,ZNF527,ZNF569,LOC100630785,ZNF91,LOC100064247,LOC100064307,LOC100146816,LOC100066399,LOC100066514,LOC100064368,LOC100066584,LOC100066608,CADM4,LOC100147096,LOC100070243,LOC100070229,LOC100070215,XRCC1,LOC100070189,LOC100146195,LOC100070137,LOC100070126,LOC100629397,LOC100070087,BSP2,BSP1,LOC100629383,LOC100065413,LOC100070041,LOC100070023,LOC100146292,LOC100146603,LOC100069985,LOC1
10	8969099	9610442	641344	4	1	LOC100066117,LOC100064192,LOC100064166,LOC100064140,YIF1B,LOC100064080,LOC100629152,LOC100064014,LOC100063981,LOC100063688,WDR87,LOC100063034,LOC100064247
10	8969099	10223651	1254553	3	1	LOC100147683,LOC100066750,LOC100147106,IL28B,LOC100064427,SAMD4B,LOC100067447,LOC100629870,LOC100067228,PAK4,LOC100066651,LOC100064399,LOC100629402,LOC100146611,HNRNPL,LOC100066427,LOC100064337,LOC100066370,LOC100066343,MAP4K1,LOC100066117,LOC100064192,LOC100064166,LOC100064140,YIF1B,LOC100064080,LOC100629152,LOC100064014,LOC100063981,LOC100063688,WDR87,LOC100063034,LOC100064247,LOC100064307,LOC100146816,LOC100066399,LOC100066514,LOC100064368,LOC100066584,LOC100066608

Chrom	Start	End	Size	Cn	Samples (n)	Genes
10	8969099	13004730	4035632	3	1	LOC100147683,LOC100066750,LOC100147106,IL28B,LOC100064427,PAF1,LOC100069407,LOC100069314,LOC100630667,LOC100069287,TRNAI-UAU,LOC100069277,LOC100630611,LOC100630516,LOC100630497,LOC100069166,LOC100630429,LOC100630409,LOC100630385,LOC100630359,LOC100065058,LOC100065028,BCKDHA,LOC100064997,LOC100630482,LOC100146820,TGFB1,LOC100064964,HNRNPUL1,AXL,LOC100147675,LOC100068618,LOC100630234,LOC100068603,LOC100147010,LOC100068569,LOC100630152,LOC100068510,LOC100068495,LOC100146413,LOC100146204,CYP2A13,LOC100630054,LOC100068334,LOC100068315,LOC100068295,LOC100064859,LOC100064830,MIA,LOC100068274,LOC100068235,ITPKC,ADCK4,NUMBL,LOC100064750,LOC100630098,LOC100146416,LOC100068145,LOC100068129,LOC100068110,LOC100068085,LOC100068062,PLD3,LOC100068049,AKT2,LOC100146308,MAP3K10,LOC100067940,EQUCABV1R901,ZNF91,LOC100067812,ZNF546,PSMC4,LOC100146404,LOC100064571,LOC100147011,LOC100067694,LOC100067648,LOC100067604,SUPT5H,LOC100064489,PLEKHG2,SAMD4B,LOC100067447,LOC100629870,LOC100067228,PAK4,LOC100066651,LOC100064399,LOC100629402,LOC100146611,HNRNPL,LOC100066427,LOC100064337,LOC100066370,LOC100066343,MAP4K1,LOC100066117,LOC100064192,LOC100064166,LOC100064140,YIF1B,LOC100064080,LOC100629152,LOC100064014,LOC100063981,LOC100063688,WDR87,LOC100063034,LOC100064247,LOC100064307,LOC100146816,LOC100066399,LOC100066514,LOC100064368,LOC100066584,LOC100066608
10	9044349	11201154	2156806	4	1	LOC100147683,LOC100066750,LOC100147106,IL28B,LOC100064427,PAF1,TRNAI-UAU,LOC100068315,LOC100068295,LOC100064859,LOC100064830,MIA,LOC100068274,LOC100068235,ITPKC,ADCK4,NUMBL,LOC100064750,LOC100630098,LOC100146416,LOC100068145,LOC100068129,LOC100068110,LOC100068085,LOC100068062,PLD3,LOC100068049,AKT2,LOC100146308,MAP3K10,LOC100067940,EQUCABV1R901,ZNF91,LOC100067812,ZNF546,PSMC4,LOC100146404,LOC100064571,LOC100147011,LOC100067694,LOC100067648,LOC100067604,SUPT5H,LOC100064489,PLEKHG2,SAMD4B,LOC100067447,LOC100629870,LOC100067228,PAK4,LOC100066651,LOC100064399,LOC100629402,LOC100146611,HNRNPL,LOC100066427,LOC100064337,LOC100066370,LOC100066343,MAP4K1,LOC100066117,LOC100064192,LOC100064166,LOC100064140,YIF1B,LOC100064080,LOC100629152,LOC100064014,LOC100063981,LOC100063688,WDR87,LOC100064247,LOC100064307,LOC100146816,LOC100066399,LOC100066514,LOC100064368,LOC100066584,LOC100066608
10	10907542	10947011	39470	1	1	LOC100146416
10	10907542	10983696	76155	1	1	LOC100064750,LOC100630098,LOC100146416
10	11160323	14618796	3458474	3	1	LOC100069898,LOC100069866,LOC100065329,CD79A,LOC100629122,LOC100147673,ATP1A3,LOC100069742,ZNF574,POU2F2,LOC100065272,LOC100146293,LOC100069693,ETF1,LOC100069673,LOC100065240,LOC100065213,LOC100147203,MEGF8,LOC100069615,LOC100069598,LOC100630773,LOC100069585,LOC100069572,LOC100630749,LOC100069549,LOC100630655,LOC100069522,LOC100065118,LOC100147014,LOC100069407,LOC100069314,LOC100630667,LOC100069287,LOC100069277,LOC100630611,LOC100630516,LOC100630497,LOC100069166,LOC100630429,LOC100630409,LOC100630385,LOC100630359,LOC100065058,LOC100065028,BCKDHA,LOC100064997,LOC100630482,LOC100146820,TGFB1,LOC100064964,HNRNPUL1,AXL,LOC100147675,LOC100068618,LOC100630234,LOC100068603,LOC100147010,LOC100068569,LOC100630152,LOC100068510,LOC100068495,LOC100146413,LOC100146204,CYP2A13,LOC100630054,LOC100068334,CADM4,LOC100147096,LOC100070243,LOC100070229,LOC100070215,XRCC1,LOC100070189,LOC100146195,LOC100070137,LOC100070126,LOC100629397,LOC100070087,BSP2,BSP1,LOC100629383,LOC100065413,LOC100070041,LOC100070023,LOC100146292,LOC100146603,LOC100069985,LOC100065387,LOC100629292,LOC100069954,LOC100069940,LOC100069923,LOC100069909
10	11713642	17202862	5489221	3	1	LOC100069898,LOC100069866,LOC100065329,CD79A,LOC100629122,LOC100147673,ATP1A3,LOC100069742,ZNF574,POU2F2,LOC100065272,LOC100146293,LOC100069693,ETF1,LOC100069673,LOC100065240,LOC100065213,LOC100147203,MEGF8,LOC100069615,LOC100069598,LOC100630773,LOC100069585,LOC100069572,LOC100630749,LOC100069549,LOC100630655,LOC100069522,LOC100065118,LOC100147014,LOC100069407,LOC100069314,LOC100630667,LOC100069287,LOC100069277,LOC100630611,LOC100630516,LOC100630497,LOC100069166,LOC100630429,LOC100630409,LOC100630385,LOC100630359,LOC100065058,LOC100065028,BCKDHA,LOC100064997,LOC100630482,LOC100146820,TGFB1,LOC100064964,HNRNPUL1,AXL,LOC100147675,LOC100068618,LOC100630234,LOC100146607,NPAS1,LOC100065944,LOC100071193,LOC100146913,LOC100147490,LOC100071175,LOC100146513,LOC100071165,LOC100630827,LOC100071157,LOC100071150,LOC100071123,CCDC8,PPP5C,HIF3A,LOC100071088,MIR769,LOC100071074,LOC100630711,MILL,LOC100071047,LOC100071039,LOC100146821,LOC100071026,MIR769B,LOC100071017,LOC100071008,LOC100065786,LOC100065765,LOC100146414,IRF2BP1,LOC100070957,SYMPK,LOC100070937,LOC100070931,LOC100146609,FBXO46,LOC100065716,LOC100065699,LOC100070901,MIR330,LOC100070888,LOC100070869,LOC100070858,LOC100070851,LOC100070843,LOC100147095,TRNASTOP-UCA,LOC100065680,LOC100065660,LOC100070825,LOC100146706,LOC100070804,LOC100147291,LOC100065641,MARK4,LOC100146291,LOC100070719,LOC100630138,LOC100065619,LOC100065597,ZNF296,LOC100065554,LOC100070664,CLPTM1,LOC100065532,LOC100065506,LOC100065481,LOC100070639,LOC100146715,PVRL2,BCAM,LOC100147299,LOC100146304,CEACAM16,LOC100629940,LOC100070567,LOC100146196,ZNF180,LOC100629827,LOC100070542,ZFP112,ZNF235,LOC100070487,LOC100629711,ZNF234,LOC100070401,ZNF404,LOC100070361,EQUCABV1R-PS906,LOC100147197,LOC100147493,LOC100070296,LOC100070288,LOC100070279,PLAUR,CADM4,LOC100147096,LOC100070243,LOC100070229,LOC100070215,XRCC1,LOC100070189,LOC100146195,LOC100070137,LOC100070126,LOC100629397,LOC100070087,BSP2,BSP1,LOC100629383,LOC100065413,LOC100070041,LOC100070023,LOC100146292,LOC
10	12130644	14618796	2488153	3	1	LOC100069898,LOC100069866,LOC100065329,CD79A,LOC100629122,LOC100147673,ATP1A3,LOC100069742,ZNF574,POU2F2,LOC100065272,LOC100146293,LOC100069693,ETF1,LOC100069673,LOC100065240,LOC100065213,LOC100147203,MEGF8,LOC100069615,LOC100069598,LOC100630773,LOC100069585,LOC100069572,LOC100630749,LOC100069549,LOC100630655,LOC100069522,LOC100065118,LOC100147014,LOC100069407,LOC100069314,LOC100630667,LOC100069287,LOC100069277,LOC100630611,LOC100630516,LOC100630497,LOC100069166,LOC100630429,LOC100630409,LOC100630385,LOC100630359,CADM4,LOC100147096,LOC100070243,LOC100070229,LOC100070215,XRCC1,LOC100070189,LOC100146195,LOC100070137,LOC100070126,LOC100629397,LOC100070087,BSP2,BSP1,LOC100629383,LOC100065413,LOC100070041,LOC100070023,LOC100146292,LOC100146603,LOC100069985,LOC100065387,LOC100629292,LOC100069954,LOC100069940,LOC100069923,LOC100069909

Chrom	Start	End	Size	Cn	Samples (n)	Genes
10	12130644	20112024	7981381	3	1	LOC100069898,LOC100069866,LOC100065329,CD79A,LOC100629122,LOC100147673,ATP1A3,LOC100069742,ZNF574,POU2F2,LOC100065272,LOC100146293,LOC100069693,ETF1,LOC100069673,LOC100065240,LOC100065213,LOC100147203,MEGF8,LOC100069615,LOC100069598,LOC100630773,LOC100069585,LOC100069572,LOC100630749,LOC100069549,LOC100630655,LOC100069522,LOC100065118,LOC100147014,LOC100069407,LOC100069314,LOC100630667,LOC100069287,LOC100069277,LOC100630611,LOC100630516,LOC100630497,LOC100069166,LOC100630429,LOC100630409,LOC100630385,LOC100630359,SYT3,LOC100146202,LOC100062997,LOC100146518,POLD1,NR1H2,LOC100057548,NAPSA,LOC100630525,LOC100056948,ZNF473,LOC100052342,LOC100147018,LOC100146705,LOC100056315,LOC100147304,TBC1D17,LOC100052282,PNKP,LOC100056188,LOC100056147,LOC100052158,AP2A1,LOC100056067,CPT1C,PRMT1,LOC100055909,LOC100055868,LOC100055821,LOC100055781,LOC100146713,LOC100055647,LOC100052038,LOC100055602,LOC100147583,MIR150,LOC100051978,LOC100051915,LOC100055522,LOC100055479,LOC100051855,LOC100146907,LOC100051792,LOC100055398,LOC100055354,TEAD2,LOC100055274,LOC100055225,LOC100055178,HRC,LOC100054955,LOC100146401,LOC100054907,LOC100147009,LOC100054859,LHB,LOC100051665,GYS1,FTL,LOC100054674,LOC100051521,LOC100054628,TULP2,LOC100051382,PLEKHA4,LOC100051311,BCAT2,LOC100054542,LOC100054499,LOC100054454,LOC100054403,LOC100054356,LOC100054308,LOC100054259,LOC100054216,LOC100054170,LOC100054121,LOC100146301,SPHK2,RPL18,LOC100630545,LOC100147298,LOC100147584,CYTH2,LOC100146909,GRWD1,LOC100050944,LOC100050869,TMEM143,LOC100050794,LOC100053569,LOC100053528,EQUCABV1R-PS954,EQUCABV1R936,EQUCABV1R935,EQUCABV1R934,EQUCABV1R933,LOC100629445,LIG1,LOC100050634,LOC100053135,LOC100629393,LOC100050557,LOC100052993,LOC100050329,LOC100052843,LOC100050400,SULT2A1,LOC100050266,SEPW1,ZNF541,LOC100066095,LOC100146519,LOC100146819,MEIS3,DHX34,LOC100071231,LOC100065997,LOC100146412,LOC100146714,LOC100071215,ZC3H4,LOC100146607,NPAS1,LOC100065944,LOC100071193,LOC100146913,LOC100147490,LOC100071175,LOC100146513,LOC10007116
10	15251397	16996377	1744981	3,4	1,1	LOC100146913,LOC100147490,LOC100071175,LOC100146513,LOC100071165,LOC100630827,LOC100071157,LOC100071150,LOC100071123,CCDC8,PPP5C,HIF3A,LOC100071088,MIR769,LOC100071074,LOC100630711,MILL,LOC100071047,LOC100071039,LOC100146821,LOC100071026,MIR769B,LOC100071017,LOC100071008,LOC100065786,LOC100065765,LOC100146414,IRF2BP1,LOC100070957,SYMPK,LOC100070937,LOC100070931,LOC100146609,FBXO46,LOC100065716,LOC100065699,LOC100070901,MIR330,LOC100070888,LOC100070869,LOC100070858,LOC100070851,LOC100070843,LOC100147095,TRNASTOP-UCA,LOC100065680,LOC100065660,LOC100070825,LOC100146706,LOC100070804,LOC100147291,LOC100065641,MARK4,LOC100146291,LOC100070719,LOC100630138,LOC100065619,LOC100065597,ZNF296,LOC100065554,LOC100070664,CLPTM1,LOC100065532,LOC100065506,LOC100065481,LOC100070639,LOC100146715,PVRL2,BCAM,LOC100147299,LOC100146304,CEACAM16,LOC100629940,LOC100070567,LOC100146196
10	15868347	17594851	1726505	4	1	ZNF541,LOC100066095,LOC100146519,LOC100146819,MEIS3,DHX34,LOC100071231,LOC100065997,LOC100146412,LOC100146714,LOC100071215,ZC3H4,LOC100146607,NPAS1,LOC100065944,LOC100071193,LOC100146913,LOC100147490,LOC100071175,LOC100146513,LOC100071165,LOC100630827,LOC100071157,LOC100071150,LOC100071123,CCDC8,PPP5C,HIF3A,LOC100071088,MIR769,LOC100071074,LOC100630711,MILL,LOC100071047,LOC100071039,LOC100146821,LOC100071026,MIR769B,LOC100071017,LOC100071008,LOC100065786,LOC100065765,LOC100146414,IRF2BP1,LOC100070957,SYMPK,LOC100070937,LOC100070931,LOC100146609,FBXO46,LOC100065716,LOC100065699,LOC100070901,MIR330,LOC100070888,LOC100070869,LOC100070858,LOC100070851,LOC100070843,LOC100147095,TRNASTOP-UCA,LOC100065680,LOC100065660,LOC100070825,LOC100146706,LOC100070804,LOC100147291,LOC100065641,MARK4
10	16265485	21210260	4944776	4	1	LOC100066069,LOC100066085,LOC100066110,LOC100066140,LOC100066165,LOC100066192,LOC100066226,LOC100066252,LOC100066276,LOC100066363,LOC100066389,LOC100066419,LOC100066474,LOC100629327,LOC100066601,LOC100062799,LOC100147297,LOC100062828,LOC100066622,LOC100630910,SIGLEC10,LOC100066668,LOC100066691,LOC100066716,LOC100066744,LOC100066764,LOC100066785,LOC100066828,LOC100066849,LOC100066865,LOC100062855,LOC100147293,LOC100067040,LOC100067063,LOC100067108,LOC100067130,LOC100146904,LOC100067196,LOC100067301,LOC100146194,LOC100146510,LOC100067348,LOC100067485,LOC100067532,LOC100147398,KLK1E2,LOC100067618,KLK1E1,LOC100067778,LOC100147302,LOC100067854,LOC100146911,LOC100630637,SYT3,LOC100146202,LOC100062997,LOC100146518,POLD1,NR1H2,LOC100057548,NAPSA,LOC100630525,LOC100056948,ZNF473,LOC100052342,LOC100147018,LOC100146705,LOC100056315,LOC100147304,TBC1D17,LOC100052282,PNKP,LOC100056188,LOC100056147,LOC100052158,AP2A1,LOC100056067,CPT1C,PRMT1,LOC100055909,LOC100055868,LOC100055821,LOC100055781,LOC100146713,LOC100055647,LOC100052038,LOC100055602,LOC100147583,MIR150,LOC100051978,LOC100051915,LOC100055522,LOC100055479,LOC100051855,LOC100146907,LOC100051792,LOC100055398,LOC100055354,TEAD2,LOC100055274,LOC100055225,LOC100055178,HRC,LOC100054955,LOC100146401,LOC100054907,LOC100147009,LOC100054859,LHB,LOC100051665,GYS1,FTL,LOC100054674,LOC100051521,LOC100054628,TULP2,LOC100051382,PLEKHA4,LOC100051311,BCAT2,LOC100054542,LOC100054499,LOC100054454,LOC100054403,LOC100054356,LOC100054308,LOC100054259,LOC100054216,LOC100054170,LOC100054121,LOC100146301,SPHK2,RPL18,LOC100630545,LOC100147298,LOC100147584,CYTH2,LOC100146909,GRWD1,LOC100050944,LOC100050869,TMEM143,LOC100050794,LOC100053569,LOC100053528,EQUCABV1R-PS954,EQUCABV1R936,EQUCABV1R935,EQUCABV1R934,EQUCABV1R933,LOC100629445,LIG1,LOC100050634,LOC100053135,LOC100629393,LOC100050557,LOC100052993,LOC100050329,LOC100052843,LOC100050400,SULT2A1,LOC100050266,SEPW1,ZNF541,LOC100066095,LOC100146519,LOC100146819,MEIS3,DHX34,LOC100071231,LOC100065997
10	18395136	19691565	1296430	1	1	LOC100147018,LOC100146705,LOC100056315,LOC100147304,TBC1D17,LOC100052282,PNKP,LOC100056188,LOC100056147,LOC100052158,AP2A1,LOC100056067,CPT1C,PRMT1,LOC100055909,LOC100055868,LOC100055821,LOC100055781,LOC100146713,LOC100055647,LOC100052038,LOC100055602,LOC100147583,MIR150,LOC100051978,LOC100051915,LOC100055522,LOC100055479,LOC100051855,LOC100146907,LOC100051792,LOC100055398,LOC100055354,TEAD2,LOC100055274,LOC100055225,LOC100055178,HRC,LOC100054955,LOC100146401,LOC100054907,LOC100147009,LOC100054859,LHB,LOC100051665,GYS1,FTL,LOC100054674,LOC100051521,LOC100054628,TULP2,LOC100051382,PLEKHA4,LOC100051311,BCAT2,LOC100054542,LOC100054499,LOC100054454,LOC100054403,LOC100054356,LOC100054308,LOC100054259,LOC100054216,LOC100054170,LOC100054121,LOC100146301,SPHK2,RPL18,LOC100630545,LOC100147298,LOC100147584,CYTH2,LOC100146909,GRWD1,LOC100050944,LOC100050869,TMEM143,LOC100050794,LOC100053569,LOC100053528

Chrom	Start	End	Size	Cn	Samples (n)	Genes
10	18440965	19264042	823078	1	1	LOC100051792,LOC100055398,LOC100055354,TEAD2,LOC100055274,LOC100055225,LOC100055178,HRC,LOC100054955,LOC100146401,LOC100054907,LOC100147009,LOC100054859,LHB,LOC100051665,GYS1,FTL,LOC100054674,LOC100051521,LOC100054628,TULP2,LOC100051382,PLEKHA4,LOC100051311,BCAT2,LOC100054542,LOC100054499,LOC100054454,LOC100054403,LOC100054356,LOC100054308,LOC100054259,LOC100054216,LOC100054170,LOC100054121,LOC100146301,SPHK2,RPL18,LOC100630545,LOC100147298,LOC100147584,CYTH2,LOC100146909,GRWD1,LOC100050944,LOC100050869,TMEM143,LOC100050794
10	18534246	20766310	2232065	3	1	LOC100066691,LOC100066716,LOC100066744,LOC100066764,LOC100066785,LOC100066828,LOC100066849,LOC100066865,LOC100062855,LOC100147293,LOC100067040,LOC100067063,LOC100067108,LOC100067130,LOC100146904,LOC100067196,LOC100067301,LOC100146194,LOC100146510,LOC100067348,LOC100067485,LOC100067532,LOC100147398,KLK1E2,LOC100067618,KLK1E1,LOC100067778,LOC100147302,LOC100067854,LOC100146911,LOC100630637,SYT3,LOC100146202,LOC100062997,LOC100146518,POLD1,NR1H2,LOC100057548,NAPSA,LOC100630525,LOC100056948,ZNF473,LOC100052342,LOC100147018,LOC100146705,LOC100056315,LOC100147304,TBC1D17,LOC100052282,PNKP,LOC100056188,LOC100056147,LOC100052158,AP2A1,LOC100056067,CPT1C,PRMT1,LOC100055909,LOC100055868,LOC100055821,LOC100055781,LOC100146713,LOC100055647,LOC100052038,LOC100055602,LOC100147583,MIR150,LOC100051978,LOC100051915,LOC100055522,LOC100055479,LOC100051855,LOC100146907,LOC100051792,LOC100055398,LOC100055354,TEAD2,LOC100055274,LOC100055225,LOC100055178,HRC,LOC100054955,LOC100146401,LOC100054907,LOC100147009,LOC100054859,LHB,LOC100051665,GYS1,FTL,LOC100054674,LOC100051521,LOC100054628,TULP2,LOC100051382,PLEKHA4,LOC100051311,BCAT2,LOC100054542,LOC100054499,LOC100054454,LOC100054403,LOC100054356,LOC100054308,LOC100054259,LOC100054216,LOC100054170,LOC100054121,LOC100146301,SPHK2,RPL18,LOC100630545,LOC100147298,LOC100147584,CYTH2,LOC100146909
10	18865342	20112024	1246683	3	1	SYT3,LOC100146202,LOC100062997,LOC100146518,POLD1,NR1H2,LOC100057548,NAPSA,LOC100630525,LOC100056948,ZNF473,LOC100052342,LOC100147018,LOC100146705,LOC100056315,LOC100147304,TBC1D17,LOC100052282,PNKP,LOC100056188,LOC100056147,LOC100052158,AP2A1,LOC100056067,CPT1C,PRMT1,LOC100055909,LOC100055868,LOC100055821,LOC100055781,LOC100146713,LOC100055647,LOC100052038,LOC100055602,LOC100147583,MIR150,LOC100051978,LOC100051915,LOC100055522,LOC100055479,LOC100051855,LOC100146907,LOC100051792,LOC100055398,LOC100055354,TEAD2,LOC100055274,LOC100055225,LOC100055178,HRC,LOC100054955,LOC100146401,LOC100054907,LOC100147009,LOC100054859,LHB,LOC100051665,GYS1,FTL,LOC100054674,LOC100051521,LOC100054628,TULP2,LOC100051382
10	18865342	21511879	2646538	3	1	LOC100062696,LOC100062735,LOC100062768,LOC100146614,MIR125A,MIRLET7E,MIR99B,LOC100065804,LOC100065833,LOC100065936,ZNF175,LOC100066069,LOC100066085,LOC100066110,LOC100066140,LOC100066165,LOC100066192,LOC100066226,LOC100066252,LOC100066276,LOC100066363,LOC100066389,LOC100066419,LOC100066474,LOC100629327,LOC100066601,LOC100062799,LOC100147297,LOC100062828,LOC100066622,LOC100630910,SIGLEC10,LOC100066668,LOC100066691,LOC100066716,LOC100066744,LOC100066764,LOC100066785,LOC100066828,LOC100066849,LOC100066865,LOC100062855,LOC100147293,LOC100067040,LOC100067063,LOC100067108,LOC100067130,LOC100146904,LOC100067196,LOC100067301,LOC100146194,LOC100146510,LOC100067348,LOC100067485,LOC100067532,LOC100147398,KLK1E2,LOC100067618,KLK1E1,LOC100067778,LOC100147302,LOC100067854,LOC100146911,LOC100630637,SYT3,LOC100146202,LOC100062997,LOC100146518,POLD1,NR1H2,LOC100057548,NAPSA,LOC100630525,LOC100056948,ZNF473,LOC100052342,LOC100147018,LOC100146705,LOC100056315,LOC100147304,TBC1D17,LOC100052282,PNKP,LOC100056188,LOC100056147,LOC100052158,AP2A1,LOC100056067,CPT1C,PRMT1,LOC100055909,LOC100055868,LOC100055821,LOC100055781,LOC100146713,LOC100055647,LOC100052038,LOC100055602,LOC100147583,MIR150,LOC100051978,LOC100051915,LOC100055522,LOC100055479,LOC100051855,LOC100146907,LOC100051792,LOC100055398,LOC100055354,TEAD2,LOC100055274,LOC100055225,LOC100055178,HRC,LOC100054955,LOC100146401,LOC100054907,LOC100147009,LOC100054859,LHB,LOC100051665,GYS1,FTL,LOC100054674,LOC100051521,LOC100054628,TULP2,LOC100051382
10	18865342	24017196	5151855	3	1	LOC100054783,LOC100629212,LOC100054737,LOC100054684,LOC100054637,LOC100054595,LOC100054551,LOC100054464,LOC100146192,LOC100054414,LOC100054370,LILRB3,LOC100054272,ILT11B,LILRA6,LOC100147015,LOC100054029,LOC100630721,LOC100053883,LENG8,LOC100053777,LOC100629821,LOC100053579,LOC100053534,LOC100053487,ILT11A,KIR-ILTA,LILRA,LOC100053092,LOC100053055,LOC100053002,LOC100052952,LOC100052851,LOC100052743,TMC4,LOC100052583,LOC100049807,PRPF31,LOC100052231,LOC100052169,LOC100051988,LOC100629668,LOC100630386,LOC100630360,LOC100051802,LOC100147407,LOC100147684,LOC100062937,PRKCG,LOC100062591,LOC100062992,NLRP12,MIR371,LOC100063058,DPRX,LOC100063223,LOC100630153,LOC100063245,EQUCABV1R912,EQUCABV1R913,EQUCABV1R914,EQUCABV1R-PS937,EQUCABV1R-PS938,LOC100063381,EQUCABV1R-PS939,ZNF665,EQUCABV1R915,EQUCABV1R-PS940,EQUCABV1R916,EQUCABV1R-PS941,EQUCABV1R917,EQUCABV1R-PS942,LOC100630055,EQUCABV1R918,EQUCABV1R-PS943,LOC100064037,LOC100629506,ZNF677,ZNF729,EQUCABV1R-PS944,EQUCABV1R919,EQUCABV1R-PS945,EQUCABV1R920,EQUCABV1R-PS946,EQUCABV1R921,EQUCABV1R-PS947,EQUCABV1R922,EQUCABV1R923,EQUCABV1R924,EQUCABV1R-PS949,LOC100062627,EQUCABV1R-PS950,EQUCABV1R925,EQUCABV1R-PS951,EQUCABV1R926,LOC100062658,EQUCABV1R927,LOC100629908,EQUCABV1R928,EQUCABV1R-PS952,EQUCABV1R-PS953,EQUCABV1R929,EQUCABV1R930,EQUCABV1R931,ZNF615,LOC100065020,LOC100065081,ZNF432,LOC100065170,LOC100065203,LOC100065231,ZNF577,LOC100629767,LOC100065524,LOC100062696,LOC100062735,LOC100062768,LOC100146614,MIR125A,MIRLET7E,MIR99B,LOC100065804,LOC100065833,LOC100065936,ZNF175,LOC100066069,LOC100066085,LOC100066110,LOC100066140,LOC100066165,LOC100066192,LOC100066226,LOC100066252,LOC100066276,LOC100066363,LOC100066389,LOC100066419,LOC100066474,LOC100629327,LOC100066601,LOC100062799,LOC100147297,LOC100062828,LOC100066622,LOC100630910,SIGLEC10,LOC100066668,LOC100066691,LOC100066716,LOC100066744,LOC100066764,LOC100066785,LOC100066828,LOC100066849,LOC100066865,LOC100062855,LOC100147293,LOC100067040,LOC100067063,LOC100067108,LOC100067130,LOC
10	19222030	19691565	469536	1	1	LOC100147018,LOC100146705,LOC100056315,LOC100147304,TBC1D17,LOC100052282,PNKP,LOC100056188,LOC100056147,LOC100052158,AP2A1,LOC100056067,CPT1C,PRMT1,LOC100055909,LOC100055868,LOC100055821,LOC100055781,LOC100146713,LOC100055647,LOC100052038,LOC100055602,LOC100147583,MIR150,LOC100051978,LOC100051915,LOC100055522,LOC100055479,LOC100051855,LOC100146907,LOC100051792,LOC100055398

Chrom	Start	End	Size	Cn	Samples (n)	Genes
10	23498268	25390163	1891896	3	1	LOC100057957,ZNF582,ZNF583,ZNF667,LOC100057519,LOC100630069,NLRP5,NLRP13,NLRP4,LOC100057362,LOC100057316,LOC100629941,LOC100629921,LOC100629902,EQUCABV1R-PS909,EQUCABV1R-PS908,LOC100057074,EQUCABV1R903,EQUCABV1R902,LOC100056917,EQUCABV1R-PS907,EPN1,U2AF2,LOC100050649,LOC100146606,LOC100146612,LOC100056720,LOC100147199,LOC100146199,LOC100056498,LOC100056449,LOC100147098,LOC100056406,SSC5D,LOC100072727,LOC100056074,LOC100629670,LOC100056037,LOC100629624,SUV420H2,LOC100055654,BRSK1,LOC100050496,PPP6R1,LOC100055488,PTPRH,LOC100050341,LOC100055404,TNNI3,LOC100055363,PPP1R12C,LOC100147201,RDH13,LOC100055234,NLRP2,LOC100055141,LOC100050552,LOC100050474,LOC100055096,LOC100055052,LOC100055007,LOC100054965,LOC100054918,FCAR,KIR3DL,LOC100054826,LOC100054783,LOC100629212,LOC100054737,LOC100054684,LOC100054637,LOC100054595,LOC100054551,LOC100054464,LOC100146192,LOC100054414,LOC100054370,LILRB3,LOC100054272,ILT11B,LILRA6,LOC100147015,LOC100054029,LOC100630721,LOC100053883,LENG8,LOC100053777,LOC100629821,LOC100053579
10	24251883	26051935	1800053	3	1	DUXA,LOC100059339,LOC100051103,LOC100058997,LOC100058913,LOC100630379,LOC100058842,LOC100058808,LOC100630346,LOC100630317,LOC100630300,LOC100630245,LOC100630226,LOC100050959,LOC100630193,LOC100630476,LOC100147097,ZNF71,ZNF470,LOC100058088,LOC100057957,ZNF582,ZNF583,ZNF667,LOC100057519,LOC100630069,NLRP5,NLRP13,NLRP4,LOC100057362,LOC100057316,LOC100629941,LOC100629921,LOC100629902,EQUCABV1R-PS909,EQUCABV1R-PS908,LOC100057074,EQUCABV1R903,EQUCABV1R902,LOC100056917,EQUCABV1R-PS907,EPN1,U2AF2,LOC100050649,LOC100146606,LOC100146612,LOC100056720,LOC100147199,LOC100146199,LOC100056498,LOC100056449,LOC100147098,LOC100056406,SSC5D,LOC100072727,LOC100056074,LOC100629670,LOC100056037,LOC100629624,SUV420H2,LOC100055654,BRSK1,LOC100050496,PPP6R1,LOC100055488,PTPRH,LOC100050341,LOC100055404,TNNI3,LOC100055363,PPP1R12C,LOC100147201,RDH13,LOC100055234
10	26471267	26654044	182778	1	2	EQUCABV1R-PS912,LOC100630829,EQUCABV1R-PS911,LOC100061052,LOC100051465,LOC100060917,EQUCABV1R-PS910,EQUCABV1R907,LOC100060687
10	30107493	30642716	535224	4	1	LOC100068859,MYO6,SENP6,FILIP1
10	30642716	30827421	184706	3	1	LOC100068859
10	30764949	30827421	62473	3	2	
10	46665195	47155462	490268	1	1	LOC100065897
10	61852414	62121939	269526	1	1	LOC100629998
10	67731804	68407608	675805	1	1	
10	70162613	70531397	368785	1	1	TRDN,CLVS2
10	73096930	73250750	153821	3	1	LOC100629154
10	74942096	75277588	335493	1	1	PTPRK
11	20571	2521729	2501159	3,4	2,2	LOC100056244,LOC100050276,LOC100050207,MIR338,LOC100056201,AZI1,LOC100056115,LOC100056075,BAHCC1,ACTG1,LOC100050073,LOC100055917,NPLOC4,LOC100055829,LOC100055790,LOC100055744,LOC100055700,LOC100629157,LOC100055613,LOC100050007,LOC100055445,GCGR,LOC100055281,LOC100055235,LOC100055188,LOC100049942,LOC100055097,LOC100055053,LOC100146540,LOC100055008,LOC100054966,LOC100147615,LOC100049879,LOC100054919,LOC100054869,LOC100054827,LOC100054784,LRRC45,LOC100054738,LOC100147313,LOC100146938,LOC100057395,LOC100056238,DUS1L,FASN,MCT4,LOC100057193,LOC100057153,LOC100057113,LOC100629534,LOC100147039,LOC100056872,LOC100056827,HEXDC,LOC100056149,NARF,TRNAM-CAU,FOXK2,WDR45L,LOC100056579,LOC100056107,LOC100056532,ZNF750,LOC100056490,LOC100056441,LOC100056359
11	20571	3658961	3638391	4	1	LOC100050497,LOC100057000,LOC100056959,LOC100050416,LOC100146225,TBC1D16,CCDC40,GAA,EIF4A3,CARD14,LOC100056499,SLC26A11,LOC100056407,LOC100056365,LOC100056324,LOC100056244,LOC100050276,LOC100050207,MIR338,LOC100056201,AZI1,LOC100056115,LOC100056075,BAHCC1,ACTG1,LOC100050073,LOC100055917,NPLOC4,LOC100055829,LOC100055790,LOC100055744,LOC100055700,LOC100629157,LOC100055613,LOC100050007,LOC100055445,GCGR,LOC100055281,LOC100055235,LOC100055188,LOC100049942,LOC100055097,LOC100055053,LOC100146540,LOC100055008,LOC100054966,LOC100147615,LOC100049879,LOC100054919,LOC100054869,LOC100054827,LOC100054784,LRRC45,LOC100054738,LOC100147313,LOC100146938,LOC100057395,LOC100056238,DUS1L,FASN,MCT4,LOC100057193,LOC100057153,LOC100057113,LOC100629534,LOC100147039,LOC100056872,LOC100056827,HEXDC,LOC100056149,NARF,TRNAM-CAU,FOXK2,WDR45L,LOC100056579,LOC100056107,LOC100056532,ZNF750,LOC100056490,LOC100056441,LOC100056359
11	20571	3966651	3946081	3	1	CYTH1,USP36,TIMP2,LOC100629722,LOC100050650,LGALS3BP,CANT1,LOC100057363,LOC100057317,LOC100057280,LOC100050497,LOC100057000,LOC100056959,LOC100050416,LOC100146225,TBC1D16,CCDC40,GAA,EIF4A3,CARD14,LOC100056499,SLC26A11,LOC100056407,LOC100056365,LOC100056324,LOC100056244,LOC100050276,LOC100050207,MIR338,LOC100056201,AZI1,LOC100056115,LOC100056075,BAHCC1,ACTG1,LOC100050073,LOC100055917,NPLOC4,LOC100055829,LOC100055790,LOC100055744,LOC100055700,LOC100629157,LOC100055613,LOC100050007,LOC100055445,GCGR,LOC100055281,LOC100055235,LOC100055188,LOC100049942,LOC100055097,LOC100055053,LOC100146540,LOC100055008,LOC100054966,LOC100147615,LOC100049879,LOC100054919,LOC100054869,LOC100054827,LOC100054784,LRRC45,LOC100054738,LOC100147313,LOC100146938,LOC100057395,LOC100056238,DUS1L,FASN,MCT4,LOC100057193,LOC100057153,LOC100057113,LOC100629534,LOC100147039,LOC100056872,LOC100056827,HEXDC,LOC100056149,NARF,TRNAM-CAU,FOXK2,WDR45L,LOC100056579,LOC100056107,LOC100056532,ZNF750,LOC100056490,LOC100056441,LOC100056359
11	464390	780294	315905	4	2	LOC100056827,HEXDC,LOC100056149,NARF,TRNAM-CAU,FOXK2,WDR45L,LOC100056579,LOC100056107,LOC100056532,LOC100056490

Chrom	Start	End	Size	Cn	Samples (n)	Genes
11	464390	2521729	2057340	3,4	1,1	LOC100056244,LOC100050276,LOC100050207,MIR338,LOC100056201,AZI1,LOC100056115,LOC100056075,BAHCC1,ACTG1,LOC100050073,LOC100055917,NPLOC4,LOC100055829,LOC100055790,LOC100055744,LOC100055700,LOC100629157,LOC100055613,LOC100050007,LOC100055445,GCGR,LOC100055281,LOC100055235,LOC100055188,LOC100049942,LOC100055097,LOC100055053,LOC100146540,LOC100055008,LOC100054966,LOC100147615,LOC100049879,LOC100054919,LOC100054869,LOC100054827,LOC100054784,LRRC45,LOC100054738,LOC100147313,LOC100146938,LOC100057395,LOC100056238,DUS1L,FASN,MCT4,LOC100057193,LOC100057153,LOC100057113,LOC100629534,LOC100147039,LOC100056872,LOC100056827,HEXDC,LOC100056149,NARF,TRNAM-CAU,FOXK2,WDR45L,LOC100056579,LOC100056107,LOC100056532,LOC100056490
11	464390	3372177	2907788	3	1	LOC100057000,LOC100056959,LOC100050416,LOC100146225,TBC1D16,CCDC40,GAA,EIF4A3,CARD14,LOC100056499,SLC26A11,LOC100056407,LOC100056365,LOC100056324,LOC100056244,LOC100050276,LOC100050207,MIR338,LOC100056201,AZI1,LOC100056115,LOC100056075,BAHCC1,ACTG1,LOC100050073,LOC100055917,NPLOC4,LOC100055829,LOC100055790,LOC100055744,LOC100055700,LOC100629157,LOC100055613,LOC100050007,LOC100055445,GCGR,LOC100055281,LOC100055235,LOC100055188,LOC100049942,LOC100055097,LOC100055053,LOC100146540,LOC100055008,LOC100054966,LOC100147615,LOC100049879,LOC100054919,LOC100054869,LOC100054827,LOC100054784,LRRC45,LOC100054738,LOC100147313,LOC100146938,LOC100057395,LOC100056238,DUS1L,FASN,MCT4,LOC100057193,LOC100057153,LOC100057113,LOC100629534,LOC100147039,LOC100056872,LOC100056827,HEXDC,LOC100056149,NARF,TRNAM-CAU,FOXK2,WDR45L,LOC100056579,LOC100056107,LOC100056532,LOC100056490
11	551696	780294	228599	4	2	LOC100056827,HEXDC,LOC100056149,NARF,TRNAM-CAU,FOXK2,WDR45L,LOC100056579
11	551696	2164104	1612409	4	1	LOC100056244,LOC100050276,LOC100050207,MIR338,LOC100056201,AZI1,LOC100056115,LOC100056075,BAHCC1,ACTG1,LOC100050073,LOC100055917,NPLOC4,LOC100055829,LOC100055790,LOC100055744,LOC100055700,LOC100629157,LOC100055613,LOC100050007,LOC100055445,GCGR,LOC100055281,LOC100055235,LOC100055188,LOC100049942,LOC100055097,LOC100055053,LOC100146540,LOC100055008,LOC100054966,LOC100147615,LOC100049879,LOC100054919,LOC100054869,LOC100054827,LOC100054784,LRRC45,LOC100054738,LOC100147313,LOC100146938,LOC100057395,LOC100056238,DUS1L,FASN,MCT4,LOC100057193,LOC100057153,LOC100057113,LOC100629534,LOC100147039,LOC100056872,LOC100056827,HEXDC,LOC100056149,NARF,TRNAM-CAU,FOXK2,WDR45L,LOC100056579
11	635764	780294	144531	3,4	4,2	LOC100056827,HEXDC,LOC100056149,NARF,TRNAM-CAU,FOXK2
11	1926549	2164104	237556	4	1	LOC100056244,LOC100050276,LOC100050207,MIR338,LOC100056201
11	1926549	2521729	595181	3	2	LOC100056244,LOC100050276,LOC100050207,MIR338,LOC100056201
11	1926549	3372177	1445629	3	1	LOC100057000,LOC100056959,LOC100050416,LOC100146225,TBC1D16,CCDC40,GAA,EIF4A3,CARD14,LOC100056499,SLC26A11,LOC100056407,LOC100056365,LOC100056324,LOC100056244,LOC100050276,LOC100050207,MIR338,LOC100056201
11	3319426	3966651	647226	3	1	CYTH1,USP36,TIMP2,LOC100629722,LOC100050650,LGALS3BP,CANT1,LOC100057363,LOC100057317,LOC100057280,LOC100050497
11	5749396	6027822	278427	1	1	GALR2,LOC100630768,EXOC7,FOXJ1,RNF157,LOC100630195,LOC100058952,LOC100058914,SPHK1,LOC100058843
11	5749396	6089542	340147	1	1	EVPL,LOC100051325,GALR2,LOC100630768,EXOC7,FOXJ1,RNF157,LOC100630195,LOC100058952,LOC100058914,SPHK1,LOC100058843
11	18058613	18692852	634240	1	1	LOC100064169,NMT1,LOC100064142,LOC100064116,LOC100050208,LOC100064083,FMNL1,LOC100063951,MAP3K14,ARHGAP27,PLEKHM1,LOC100063851,LOC100063820,LOC100063794,LOC100063763,LOC100049943,WNT9B,WNT3
11	23500463	23517022	16560	1	2	LOC100147135
11	28575714	29079022	503309	1	2	LOC100070445
11	32952044	33040164	88121	1	1	LOC100057075
11	32952044	33337791	385748	1	1	LOC100057236,LOC100057203,LOC100057162,MIR301A,MIR454,LOC100629882,TRIM37,LOC100057075
11	32952044	34124425	1172382	1	1	MED13,LOC100071124,RPS6KB1,LOC100057442,MIR21,VMP1,LOC100071096,CLTC,LOC100071065,LOC100057318,LOC100057281,LOC100057236,LOC100057203,LOC100057162,MIR301A,MIR454,LOC100629882,TRIM37,LOC100057075
11	33040164	33337791	297628	1	1	LOC100057236,LOC100057203,LOC100057162,MIR301A,MIR454,LOC100629882,TRIM37,LOC100057075
11	33248110	33337791	89682	3	3	LOC100057236,LOC100057203,LOC100057162,MIR301A,MIR454,LOC100629882,TRIM37
11	37696549	42071159	4374611	3	1	LOC100629155,INOS,LOC100072001,LOC100629469,LOC100071991,LOC100146437,LOC100071980,LOC100629374,LOC100071959,LOC100071956,LOC100071952,LOC100629314,LOC100071927,LOC100629272,LOC100629244,LOC100629223,LOC100058771,KSR1,WSB1,OMG,LOC100058650,LOC100630810,NF1,RAB11FIP4,MIR193A,MIR365,LOC100071846,UTP6,LOC100071831,CRLF3,ATAD5,LOC100071813,ADAP2,LOC100147422,RHOT1,RHBDL3,LOC100058439,MIR632,ZNF207,LOC100071770,LOC100071764,LOC100071750,LOC100071736,SPACA3,ACCN1,CCL2,LOC100071714,CCL11,CCL8,CCL13,LOC100071685,LOC100058222,LOC100071676,LIG3,RFFL,LOC100071664,LOC100071659,NLE1,UNC45B,SLFN5,LOC100071627
11	38064567	38168907	104341	1	2	
11	38734872	39193063	458192	4	1	ACCN1
11	41342963	41539378	196416	3	1	KSR1
11	41342963	41652090	309128	4	1	LOC100629314,LOC100071927,LOC100629272,LOC100629244,LOC100629223,LOC100058771,KSR1
11	41342963	41786263	443301	4	4	LOC100146437,LOC100071980,LOC100629374,LOC100071959,LOC100071956,LOC100071952,LOC100629314,LOC100071927,LOC100629272,LOC100629244,LOC100629223,LOC100058771,KSR1
11	41342963	41900301	557339	3	1	INOS,LOC100072001,LOC100629469,LOC100071991,LOC100146437,LOC100071980,LOC100629374,LOC100071959,LOC100071956,LOC100071952,LOC100629314,LOC100071927,LOC100629272,LOC100629244,LOC100629223,LOC100058771,KSR1
11	47491735	48015457	523723	4	1	LOC100072734,GGT6,MYBBP1A,SPNS2,SPNS3,LOC100072717,ANKFY1,LOC100060725,LOC100072696

Chrom	Start	End	Size	Cn	Samples (n)	Genes
11	47960147	48015457	55311	3	2	LOC100072734,GGT6,MYBBP1A
11	48728501	49541824	813324	4	1	LOC100072840,KIF1C,ZFP3,LOC100629491,LOC100629477,LOC100629451,RABEP1,NUP88,LOC100061089,LOC100072802,LOC100072797,LOC100061027,LOC100060987,LOC100629516,LOC100060955,NLRP1
11	54640169	54651986	11818	1	1	
11	54640169	54714505	74337	1	3	
11	54640169	54929094	288926	1	1	
11	54645681	54651986	6306	1	1	
11	54645681	54714505	68825	1	4	
11	54645681	54812394	166714	1	2	
11	54645681	54929094	283414	1	1	
11	59722467	59737161	14695	1	1	LOC100050716
11	60659633	61282299	622667	3	1	LOC100147042,MAP2K3,LOC100053429,LOC100053330,LOC100053286,USP22,LOC100051166,MPRIP
11	61095995	61282299	186305	3	1	LOC100147042,MAP2K3
12	3653174	4794442	1141269	1	1	LOC100053145
12	3653174	5637713	1984540	1	1	LOC100630247,LOC100053145
12	6063806	6109895	46090	1	1	LRRC4C
12	8736137	14777981	6041845	3	1	LOC100058077,LOC100147282,LOC100058036,LOC100057989,LOC100057945,LOC100057894,LOC100057845,LOC100057804,LOC100057756,LOC100057709,LOC100057670,LOC100057589,LOC100057547,LOC100057513,LOC100057472,LOC100057433,LOC100057393,LOC100057352,LOC100057310,LOC100147286,LOC100146990,LOC100057269,LOC100057228,LOC100057191,LOC100057110,LOC100146600,LOC100057026,LOC100050714,LOC100056989,LOC100056947,LOC100056906,LOC100056869,LOC100146809,LOC100067978,LOC100067957,LOC100056826,LOC100056793,LOC100056755,LOC100147000,LOC100056708,LOC100056626,LOC100056578,LOC100056531,LOC100056488,LOC100629158,LOC100050633,LOC100056395,LOC100056356,LOC100056314,LOC100056273,LOC100056235,LOC100056187,LOC100056146,LOC100630788,LOC100629923,LOC100056026,LOC100055988,LOC100146992,LOC100055947,LOC100147569,LOC100146290,LOC100055908,LOC100055867,LOC100055780,LOC100630577,LOC100055646,LOC100055601,LOC100055561,LOC100055521,LOC100055478,LOC100055435,LOC100055397,LOC100055353,LOC100055309,LOC100055273,LOC100055224,LOC100055177,LOC100055129,LOC100055083,LOC100052898,LOC100147187,LOC100054954,LOC100054906,LOC100054858,LOC100054773,LOC100054722,LOC100054673,LOC100054627,LOC100054586,LOC100054541,LOC100054498,LOC100054453,LOC100146383,LOC100054355,LOC100054307,LOC100054258,LOC100054215,LOC100054169,LOC100054120,LOC100054065,LOC100054021,LOC100050556,LOC100053872,LOC100050476,LOC100053820,LOC100053768,LOC100629822,LOC100053672,LOC100053625,LOC100053568,LOC100053527,LOC100147669,LOC100053479,LOC100053427,LOC100053382,LOC100053328,LOC100053284,LOC100053236,LOC100053185,LOC100053134,LOC100053085,LOC100053045,LOC100052992,LOC100146502,LOC100052946,LOC100146388,LOC100052842,LOC100147383,LOC100052788,LOC100052732,LOC100050399,LOC100052683,LOC100052629,LOC100052515,LOC100052454,LOC100052398,LOC100052341,LOC100052217,LOC100052157,LOC100052096,LOC100052037,LOC100146189,LOC100051977,LOC100051914,LOC100146810,LOC100050328,LOC100051731,LOC100051664,LOC100051592,LOC100051520,LOC100051452,LOC100051381,LOC100050265,LOC10063083
12	9131478	10526206	1394729	3	3	MAPK8IP1,LOC100056637,SLC35C1,LOC100056544,MIR493A,LOC100056452,LOC100056367,LOC100050141,LOC100056203,LOC100056000,LOC100055958
12	9727155	15073209	5346055	3	1	LOC100058472,LOC100058429,LOC100058385,LOC100058346,LOC100146506,LOC100058253,LOC100147086,LOC100630034,LOC100058212,LOC100058168,LOC100058123,LOC100058077,LOC100147282,LOC100058036,LOC100057989,LOC100057945,LOC100057894,LOC100057845,LOC100057804,LOC100057756,LOC100057709,LOC100057670,LOC100057589,LOC100057547,LOC100057513,LOC100057472,LOC100057433,LOC100057393,LOC100057352,LOC100057310,LOC100147286,LOC100146990,LOC100057269,LOC100057228,LOC100057191,LOC100057110,LOC100146600,LOC100057026,LOC100050714,LOC100056989,LOC100056947,LOC100056906,LOC100056869,LOC100146809,LOC100067978,LOC100067957,LOC100056826,LOC100056793,LOC100056755,LOC100147000,LOC100056708,LOC100056626,LOC100056578,LOC100056531,LOC100056488,LOC100629158,LOC100050633,LOC100056395,LOC100056356,LOC100056314,LOC100056273,LOC100056235,LOC100056187,LOC100056146,LOC100630788,LOC100629923,LOC100056026,LOC100055988,LOC100146992,LOC100055947,LOC100147569,LOC100146290,LOC100055908,LOC100055867,LOC100055780,LOC100630577,LOC100055646,LOC100055601,LOC100055561,LOC100055521,LOC100055478,LOC100055435,LOC100055397,LOC100055353,LOC100055309,LOC100055273,LOC100055224,LOC100055177,LOC100055129,LOC100055083,LOC100052898,LOC100147187,LOC100054954,LOC100054906,LOC100054858,LOC100054773,LOC100054722,LOC100054673,LOC100054627,LOC100054586,LOC100054541,LOC100054498,LOC100054453,LOC100146383,LOC100054355,LOC100054307,LOC100054258,LOC100054215,LOC100054169,LOC100054120,LOC100054065,LOC100054021,LOC100050556,LOC100053872,LOC100050476,LOC100053820,LOC100053768,LOC100629822,LOC100053672,LOC100053625,LOC100053568,LOC100053527,LOC100147669,LOC100053479,LOC100053427,LOC100053382,LOC100053328,LOC100053284,LOC100053236,LOC100053185,LOC100053134,LOC100053085,LOC100053045,LOC100052992,LOC100146502,LOC100052946,LOC100146388,LOC100052842,LOC100147383,LOC100052788,LOC100052732,LOC100050399,LOC100052683,LOC100052629,LOC100052515,LOC100052454,LOC100052398,LOC100052341,LOC100052217,LOC100052157,LOC100052096,LOC100052037,LOC100146189,LOC10005197

Chrom	Start	End	Size	Cn	Samples (n)	Genes
12	10001331	15073209	5071879	3	1	LOC100058472,LOC100058429,LOC100058385,LOC100058346,LOC100146506,LOC100058253,LOC100147086,LOC100630034,LOC100058212,LOC100058168,LOC100058123,LOC100058077,LOC100147282,LOC100058036,LOC100057989,LOC100057945,LOC100057894,LOC100057845,LOC100057804,LOC100057756,LOC100057709,LOC100057670,LOC100057589,LOC100057547,LOC100057513,LOC100057472,LOC100057433,LOC100057393,LOC100057352,LOC100057310,LOC100147286,LOC100146990,LOC100057269,LOC100057228,LOC100057191,LOC100057110,LOC100146600,LOC100057026,LOC100050714,LOC100056989,LOC100056947,LOC100056906,LOC100056869,LOC100146809,LOC100067978,LOC100067957,LOC100056826,LOC100056793,LOC100056755,LOC100147000,LOC100056708,LOC100056626,LOC100056578,LOC100056531,LOC100056488,LOC100629158,LOC100050633,LOC100056395,LOC100056356,LOC100056314,LOC100056273,LOC100056235,LOC100056187,LOC100056146,LOC100630788,LOC100629923,LOC100056026,LOC100055988,LOC100146992,LOC100055947,LOC100147569,LOC100146290,LOC100055908,LOC100055867,LOC100055780,LOC100630577,LOC100055646,LOC100055601,LOC100055561,LOC100055521,LOC100055478,LOC100055435,LOC100055397,LOC100055353,LOC100055309,LOC100055273,LOC100055224,LOC100055177,LOC100055129,LOC100055083,LOC100052898,LOC100147187,LOC100054954,LOC100054906,LOC100054858,LOC100054773,LOC100054722,LOC100054673,LOC100054627,LOC100054586,LOC100054541,LOC100054498,LOC100054453,LOC100146383,LOC100054355,LOC100054307,LOC100054258,LOC100054215,LOC100054169,LOC100054120,LOC100054065,LOC100054021,LOC100050556,LOC100053872,LOC100050476,LOC100053820,LOC100053768,LOC100629822,LOC100053672,LOC100053625,LOC100053568,LOC100053527,LOC100147669,LOC100053479,LOC100053427,LOC100053382,LOC100053328,LOC100053284,LOC100053236,LOC100053185,LOC100053134,LOC100053085,LOC100053045,LOC100052992,LOC100146502,LOC100052946,LOC100146388,LOC100052842,LOC100147383,LOC100052788,LOC100052732,LOC100050399,LOC100052683,LOC100052629,LOC100052515,LOC100052454,LOC100052398,LOC100052341,LOC100052217,LOC100052157,LOC100052096,LOC100052037,LOC100146189,LOC10005197
12	12232275	15073209	2840935	3	1	LOC100058472,LOC100058429,LOC100058385,LOC100058346,LOC100146506,LOC100058253,LOC100147086,LOC100630034,LOC100058212,LOC100058168,LOC100058123,LOC100058077,LOC100147282,LOC100058036,LOC100057989,LOC100057945,LOC100057894,LOC100057845,LOC100057804,LOC100057756,LOC100057709,LOC100057670,LOC100057589,LOC100057547,LOC100057513,LOC100057472,LOC100057433,LOC100057393,LOC100057352,LOC100057310,LOC100147286,LOC100146990,LOC100057269,LOC100057228,LOC100057191,LOC100057110,LOC100146600,LOC100057026,LOC100050714,LOC100056989,LOC100056947,LOC100056906,LOC100056869,LOC100146809,LOC100067978,LOC100067957,LOC100056826,LOC100056793,LOC100056755,LOC100147000,LOC100056708,LOC100056626,LOC100056578,LOC100056531,LOC100056488,LOC100629158,LOC100050633,LOC100056395,LOC100056356,LOC100056314,LOC100056273,LOC100056235,LOC100056187,LOC100056146,LOC100630788,LOC100629923,LOC100056026,LOC100055988,LOC100146992,LOC100055947,LOC100147569,LOC100146290,LOC100055908,LOC100055867,LOC100055780,LOC100630577,LOC100055646,LOC100055601,LOC100055561,LOC100055521,LOC100055478,LOC100055435,LOC100055397,LOC100055353,LOC100055309,LOC100055273,LOC100055224,LOC100055177,LOC100055129,LOC100055083,LOC100052898,LOC100147187,LOC100054954,LOC100054906,LOC100054858,LOC100054773,LOC100054722,LOC100054673,LOC100054627,LOC100054586,LOC100054541,LOC100054498,LOC100054453,LOC100146383,LOC100054355,LOC100054307,LOC100054258,LOC100054215,LOC100054169,LOC100054120,LOC100054065,LOC100054021,LOC100050556,LOC100053872,LOC100050476,LOC100053820,LOC100053768,LOC100629822,LOC100053672,LOC100053625,LOC100053568,LOC100053527,LOC100147669,LOC100053479,LOC100053427,LOC100053382,LOC100053328,LOC100053284,LOC100053236,LOC100053185,LOC100053134,LOC100053085,LOC100053045,LOC100052992,LOC100146502,LOC100052946,LOC100146388,LOC100052842,LOC100147383,LOC100052788,LOC100052732,LOC100050399,LOC100052683,LOC100052629,LOC100052515,LOC100052454,LOC100052398,LOC100052341,LOC100052217,LOC100052157,LOC100052096,LOC100052037,LOC100146189,LOC10005197
12	12333201	12594142	260942	3	1	LOC100051664,LOC100051592,LOC100051520,LOC100051452,LOC100051381,LOC100050265,LOC100630831,LOC100051236,LOC100146284,LOC100058772,LOC100058728,PTPRJ
12	12333201	13170959	837759	3	1	LOC100053820,LOC100053768,LOC100629822,LOC100053672,LOC100053625,LOC100053568,LOC100053527,LOC100147669,LOC100053479,LOC100053427,LOC100053382,LOC100053328,LOC100053284,LOC100053236,LOC100053185,LOC100053134,LOC100053085,LOC100053045,LOC100052992,LOC100146502,LOC100052946,LOC100146388,LOC100052842,LOC100147383,LOC100052788,LOC100052732,LOC100050399,LOC100052683,LOC100052629,LOC100052515,LOC100052454,LOC100052398,LOC100052341,LOC100052217,LOC100052157,LOC100052096,LOC100052037,LOC100146189,LOC100051977,LOC100051914,LOC100146810,LOC100050328,LOC100051731,LOC100051664,LOC100051592,LOC100051520,LOC100051452,LOC100051381,LOC100050265,LOC100630831,LOC100051236,LOC100146284,LOC100058772,LOC100058728,PTPRJ
12	12333201	13946509	1613309	4	1	LOC100056146,LOC100630788,LOC100629923,LOC100056026,LOC100055988,LOC100146992,LOC100055947,LOC100147569,LOC100146290,LOC100055908,LOC100055867,LOC100055780,LOC100630577,LOC100055646,LOC100055601,LOC100055561,LOC100055521,LOC100055478,LOC100055435,LOC100055397,LOC100055353,LOC100055309,LOC100055273,LOC100055224,LOC100055177,LOC100055129,LOC100055083,LOC100052898,LOC100147187,LOC100054954,LOC100054906,LOC100054858,LOC100054773,LOC100054722,LOC100054673,LOC100054627,LOC100054586,LOC100054541,LOC100054498,LOC100054453,LOC100146383,LOC100054355,LOC100054307,LOC100054258,LOC100054215,LOC100054169,LOC100054120,LOC100054065,LOC100054021,LOC100050556,LOC100053872,LOC100050476,LOC100053820,LOC100053768,LOC100629822,LOC100053672,LOC100053625,LOC100053568,LOC100053527,LOC100147669,LOC100053479,LOC100053427,LOC100053382,LOC100053328,LOC100053284,LOC100053236,LOC100053185,LOC100053134,LOC100053085,LOC100053045,LOC100052992,LOC100146502,LOC100052946,LOC100146388,LOC100052842,LOC100147383,LOC100052788,LOC100052732,LOC100050399,LOC100052683,LOC100052629,LOC100052515,LOC100052454,LOC100052398,LOC100052341,LOC100052217,LOC100052157,LOC100052096,LOC100052037,LOC100146189,LOC100051977,LOC100051914,LOC100146810,LOC100050328,LOC100051731,LOC100051664,LOC100051592,LOC100051520,LOC100051452,LOC100051381,LOC100050265,LOC100630831,LOC100051236,LOC100146284,LOC100058772,LOC100058728,PTPRJ

Chrom	Start	End	Size	Cn	Samples (n)	Genes
12	12333201	14128309	1795109	3	2	LOC100056626,LOC100056578,LOC100056531,LOC100056488,LOC100629158,LOC100050633,LOC100056395,LOC100056356,LOC100056314,LOC100056273,LOC100056235,LOC100056187,LOC100056146,LOC100630788,LOC100629923,LOC100056026,LOC100055988,LOC100146992,LOC100055947,LOC100147569,LOC100146290,LOC100055908,LOC100055867,LOC100055780,LOC100630577,LOC100055646,LOC100055601,LOC100055561,LOC100055521,LOC100055478,LOC100055435,LOC100055397,LOC100055353,LOC100055309,LOC100055273,LOC100055224,LOC100055177,LOC100055129,LOC100055083,LOC100052898,LOC100147187,LOC100054954,LOC100054906,LOC100054858,LOC100054773,LOC100054722,LOC100054673,LOC100054627,LOC100054586,LOC100054541,LOC100054498,LOC100054453,LOC100146383,LOC100054355,LOC100054307,LOC100054258,LOC100054215,LOC100054169,LOC100054120,LOC100054065,LOC100054021,LOC100050556,LOC100053872,LOC100050476,LOC100053820,LOC100053768,LOC100629822,LOC100053672,LOC100053625,LOC100053568,LOC100053527,LOC100147669,LOC100053479,LOC100053427,LOC100053382,LOC100053328,LOC100053284,LOC100053236,LOC100053185,LOC100053134,LOC100053085,LOC100053045,LOC100052992,LOC100146502,LOC100052946,LOC100146388,LOC100052842,LOC100147383,LOC100052788,LOC100052732,LOC100050399,LOC100052683,LOC100052629,LOC100052515,LOC100052454,LOC100052398,LOC100052341,LOC100052217,LOC100052157,LOC100052096,LOC100052037,LOC100146189,LOC100051977,LOC100051914,LOC100146810,LOC100050328,LOC100051731,LOC100051664,LOC100051592,LOC100051520,LOC100051452,LOC100051381,LOC100050265,LOC100630831,LOC100051236,LOC100146284,LOC100058772,LOC100058728,PTPRJ
12	12333201	14354314	2021114	3	1	LOC100056947,LOC100056906,LOC100056869,LOC100146809,LOC100067978,LOC100067957,LOC100056826,LOC100056793,LOC100056755,LOC100147000,LOC100056708,LOC100056626,LOC100056578,LOC100056531,LOC100056488,LOC100629158,LOC100050633,LOC100056395,LOC100056356,LOC100056314,LOC100056273,LOC100056235,LOC100056187,LOC100056146,LOC100630788,LOC100629923,LOC100056026,LOC100055988,LOC100146992,LOC100055947,LOC100147569,LOC100146290,LOC100055908,LOC100055867,LOC100055780,LOC100630577,LOC100055646,LOC100055601,LOC100055561,LOC100055521,LOC100055478,LOC100055435,LOC100055397,LOC100055353,LOC100055309,LOC100055273,LOC100055224,LOC100055177,LOC100055129,LOC100055083,LOC100052898,LOC100147187,LOC100054954,LOC100054906,LOC100054858,LOC100054773,LOC100054722,LOC100054673,LOC100054627,LOC100054586,LOC100054541,LOC100054498,LOC100054453,LOC100146383,LOC100054355,LOC100054307,LOC100054258,LOC100054215,LOC100054169,LOC100054120,LOC100054065,LOC100054021,LOC100050556,LOC100053872,LOC100050476,LOC100053820,LOC100053768,LOC100629822,LOC100053672,LOC100053625,LOC100053568,LOC100053527,LOC100147669,LOC100053479,LOC100053427,LOC100053382,LOC100053328,LOC100053284,LOC100053236,LOC100053185,LOC100053134,LOC100053085,LOC100053045,LOC100052992,LOC100146502,LOC100052946,LOC100146388,LOC100052842,LOC100147383,LOC100052788,LOC100052732,LOC100050399,LOC100052683,LOC100052629,LOC100052515,LOC100052454,LOC100052398,LOC100052341,LOC100052217,LOC100052157,LOC100052096,LOC100052037,LOC100146189,LOC100051977,LOC100051914,LOC100146810,LOC100050328,LOC100051731,LOC100051664,LOC100051592,LOC100051520,LOC100051452,LOC100051381,LOC100050265,LOC100630831,LOC100051236,LOC100146284,LOC100058772,LOC100058728,PTPRJ
12	12333201	14391372	2058172	3	1	LOC100057026,LOC100050714,LOC100056989,LOC100056947,LOC100056906,LOC100056869,LOC100146809,LOC100067978,LOC100067957,LOC100056826,LOC100056793,LOC100056755,LOC100147000,LOC100056708,LOC100056626,LOC100056578,LOC100056531,LOC100056488,LOC100629158,LOC100050633,LOC100056395,LOC100056356,LOC100056314,LOC100056273,LOC100056235,LOC100056187,LOC100056146,LOC100630788,LOC100629923,LOC100056026,LOC100055988,LOC100146992,LOC100055947,LOC100147569,LOC100146290,LOC100055908,LOC100055867,LOC100055780,LOC100630577,LOC100055646,LOC100055601,LOC100055561,LOC100055521,LOC100055478,LOC100055435,LOC100055397,LOC100055353,LOC100055309,LOC100055273,LOC100055224,LOC100055177,LOC100055129,LOC100055083,LOC100052898,LOC100147187,LOC100054954,LOC100054906,LOC100054858,LOC100054773,LOC100054722,LOC100054673,LOC100054627,LOC100054586,LOC100054541,LOC100054498,LOC100054453,LOC100146383,LOC100054355,LOC100054307,LOC100054258,LOC100054215,LOC100054169,LOC100054120,LOC100054065,LOC100054021,LOC100050556,LOC100053872,LOC100050476,LOC100053820,LOC100053768,LOC100629822,LOC100053672,LOC100053625,LOC100053568,LOC100053527,LOC100147669,LOC100053479,LOC100053427,LOC100053382,LOC100053328,LOC100053284,LOC100053236,LOC100053185,LOC100053134,LOC100053085,LOC100053045,LOC100052992,LOC100146502,LOC100052946,LOC100146388,LOC100052842,LOC100147383,LOC100052788,LOC100052732,LOC100050399,LOC100052683,LOC100052629,LOC100052515,LOC100052454,LOC100052398,LOC100052341,LOC100052217,LOC100052157,LOC100052096,LOC100052037,LOC100146189,LOC100051977,LOC100051914,LOC100146810,LOC100050328,LOC100051731,LOC100051664,LOC100051592,LOC100051520,LOC100051452,LOC100051381,LOC100050265,LOC100630831,LOC100051236,LOC100146284,LOC100058772,LOC100058728,PTPRJ
12	12333201	14777981	2444781	3,4	1,1	LOC100058077,LOC100147282,LOC100058036,LOC100057989,LOC100057945,LOC100057894,LOC100057845,LOC100057804,LOC100057756,LOC100057709,LOC100057670,LOC100057589,LOC100057547,LOC100057513,LOC100057472,LOC100057433,LOC100057393,LOC100057352,LOC100057310,LOC100147286,LOC100146990,LOC100057269,LOC100057228,LOC100057191,LOC100057110,LOC100146600,LOC100057026,LOC100050714,LOC100056989,LOC100056947,LOC100056906,LOC100056869,LOC100146809,LOC100067978,LOC100067957,LOC100056826,LOC100056793,LOC100056755,LOC100147000,LOC100056708,LOC100056626,LOC100056578,LOC100056531,LOC100056488,LOC100629158,LOC100050633,LOC100056395,LOC100056356,LOC100056314,LOC100056273,LOC100056235,LOC100056187,LOC100056146,LOC100630788,LOC100629923,LOC100056026,LOC100055988,LOC100146992,LOC100055947,LOC100147569,LOC100146290,LOC100055908,LOC100055867,LOC100055780,LOC100630577,LOC100055646,LOC100055601,LOC100055561,LOC100055521,LOC100055478,LOC100055435,LOC100055397,LOC100055353,LOC100055309,LOC100055273,LOC100055224,LOC100055177,LOC100055129,LOC100055083,LOC100052898,LOC100147187,LOC100054954,LOC100054906,LOC100054858,LOC100054773,LOC100054722,LOC100054673,LOC100054627,LOC100054586,LOC100054541,LOC100054498,LOC100054453,LOC100146383,LOC100054355,LOC100054307,LOC100054258,LOC100054215,LOC100054169,LOC100054120,LOC100054065,LOC100054021,LOC100050556,LOC100053872,LOC100050476,LOC100053820,LOC100053768,LOC100629822,LOC100053672,LOC100053625,LOC100053568,LOC100053527,LOC100147669,LOC100053479,LOC100053427,LOC100053382,LOC100053328,LOC100053284,LOC100053236,LOC100053185,LOC100053134,LOC100053085,LOC100053045,LOC100052992,LOC100146502,LOC100052946,LOC100146388,LOC100052842,LOC100147383,LOC100052788,LOC100052732,LOC100050399,LOC100052683,LOC100052629,LOC100052515,LOC100052454,LOC100052398,LOC100052341,LOC100052217,LOC100052157,LOC100052096,LOC100052037,LOC100146189,LOC100051977,LOC100051914,LOC100146810,LOC100050328,LOC100051731,LOC100051664,LOC100051592,LOC100051520,LOC100051452,LOC100051381,LOC100050265,LOC10063083

Chrom	Start	End	Size	Cn	Samples (n)	Genes
12	12346938	14391372	2044435	0	1	LOC100057026,LOC100050714,LOC100056989,LOC100056947,LOC100056906,LOC100056869,LOC100146809,LOC100067978,LOC100067957,LOC100056826,LOC100056793,LOC100056755,LOC100147000,LOC100056708,LOC100056626,LOC100056578,LOC100056531,LOC100056488,LOC100629158,LOC100050633,LOC100056395,LOC100056356,LOC100056314,LOC100056273,LOC100056235,LOC100056187,LOC100056146,LOC100630788,LOC100629923,LOC100056026,LOC100055988,LOC100146992,LOC100055947,LOC100147569,LOC100146290,LOC100055908,LOC100055867,LOC100055780,LOC100630577,LOC100055646,LOC100055601,LOC100055561,LOC100055521,LOC100055478,LOC100055435,LOC100055397,LOC100055353,LOC100055309,LOC100055273,LOC100055224,LOC100055177,LOC100055129,LOC100055083,LOC100052898,LOC100147187,LOC100054954,LOC100054906,LOC100054858,LOC100054773,LOC100054722,LOC100054673,LOC100054627,LOC100054586,LOC100054541,LOC100054498,LOC100054453,LOC100146383,LOC100054355,LOC100054307,LOC100054258,LOC100054215,LOC100054169,LOC100054120,LOC100054065,LOC100054021,LOC100050556,LOC100053872,LOC100050476,LOC100053820,LOC100053768,LOC100629822,LOC100053672,LOC100053625,LOC100053568,LOC100053527,LOC100147669,LOC100053479,LOC100053427,LOC100053382,LOC100053328,LOC100053284,LOC100053236,LOC100053185,LOC100053134,LOC100053085,LOC100053045,LOC100052992,LOC100146502,LOC100052946,LOC100146388,LOC100052842,LOC100147383,LOC100052788,LOC100052732,LOC100050399,LOC100052683,LOC100052629,LOC100052515,LOC100052454,LOC100052398,LOC100052341,LOC100052217,LOC100052157,LOC100052096,LOC100052037,LOC100146189,LOC100051977,LOC100051914,LOC100146810,LOC100050328,LOC100051731,LOC100051664,LOC100051592,LOC100051520,LOC100051452,LOC100051381,LOC100050265,LOC100630831,LOC100051236,LOC100146284,LOC100058772,LOC100058728
12	12524489	13149957	625469	3	1	LOC100053672,LOC100053625,LOC100053568,LOC100053527,LOC100147669,LOC100053479,LOC100053427,LOC100053382,LOC100053328,LOC100053284,LOC100053236,LOC100053185,LOC100053134,LOC100053085,LOC100053045,LOC100052992,LOC100146502,LOC100052946,LOC100146388,LOC100052842,LOC100147383,LOC100052788,LOC100052732,LOC100050399,LOC100052683,LOC100052629,LOC100052515,LOC100052454,LOC100052398,LOC100052341,LOC100052217,LOC100052157,LOC100052096,LOC100052037,LOC100146189,LOC100051977,LOC100051914,LOC100146810,LOC100050328,LOC100051731,LOC100051664,LOC100051592,LOC100051520,LOC100051452,LOC100051381,LOC100050265,LOC100630831
12	12524489	13170959	646471	1,3,4	1,2,3	LOC100053820,LOC100053768,LOC100629822,LOC100053672,LOC100053625,LOC100053568,LOC100053527,LOC100147669,LOC100053479,LOC100053427,LOC100053382,LOC100053328,LOC100053284,LOC100053236,LOC100053185,LOC100053134,LOC100053085,LOC100053045,LOC100052992,LOC100146502,LOC100052946,LOC100146388,LOC100052842,LOC100147383,LOC100052788,LOC100052732,LOC100050399,LOC100052683,LOC100052629,LOC100052515,LOC100052454,LOC100052398,LOC100052341,LOC100052217,LOC100052157,LOC100052096,LOC100052037,LOC100146189,LOC100051977,LOC100051914,LOC100146810,LOC100050328,LOC100051731,LOC100051664,LOC100051592,LOC100051520,LOC100051452,LOC100051381,LOC100050265,LOC100630831
12	12524489	13364132	839644	4	1	LOC100054215,LOC100054169,LOC100054120,LOC100054065,LOC100054021,LOC100050556,LOC100053872,LOC100050476,LOC100053820,LOC100053768,LOC100629822,LOC100053672,LOC100053625,LOC100053568,LOC100053527,LOC100147669,LOC100053479,LOC100053427,LOC100053382,LOC100053328,LOC100053284,LOC100053236,LOC100053185,LOC100053134,LOC100053085,LOC100053045,LOC100052992,LOC100146502,LOC100052946,LOC100146388,LOC100052842,LOC100147383,LOC100052788,LOC100052732,LOC100050399,LOC100052683,LOC100052629,LOC100052515,LOC100052454,LOC100052398,LOC100052341,LOC100052217,LOC100052157,LOC100052096,LOC100052037,LOC100146189,LOC100051977,LOC100051914,LOC100146810,LOC100050328,LOC100051731,LOC100051664,LOC100051592,LOC100051520,LOC100051452,LOC100051381,LOC100050265,LOC100630831
12	12524489	13379725	855237	3	2	LOC100054258,LOC100054215,LOC100054169,LOC100054120,LOC100054065,LOC100054021,LOC100050556,LOC100053872,LOC100050476,LOC100053820,LOC100053768,LOC100629822,LOC100053672,LOC100053625,LOC100053568,LOC100053527,LOC100147669,LOC100053479,LOC100053427,LOC100053382,LOC100053328,LOC100053284,LOC100053236,LOC100053185,LOC100053134,LOC100053085,LOC100053045,LOC100052992,LOC100146502,LOC100052946,LOC100146388,LOC100052842,LOC100147383,LOC100052788,LOC100052732,LOC100050399,LOC100052683,LOC100052629,LOC100052515,LOC100052454,LOC100052398,LOC100052341,LOC100052217,LOC100052157,LOC100052096,LOC100052037,LOC100146189,LOC100051977,LOC100051914,LOC100146810,LOC100050328,LOC100051731,LOC100051664,LOC100051592,LOC100051520,LOC100051452,LOC100051381,LOC100050265,LOC100630831
12	12524489	13398392	873904	3	1	LOC100054355,LOC100054307,LOC100054258,LOC100054215,LOC100054169,LOC100054120,LOC100054065,LOC100054021,LOC100050556,LOC100053872,LOC100050476,LOC100053820,LOC100053768,LOC100629822,LOC100053672,LOC100053625,LOC100053568,LOC100053527,LOC100147669,LOC100053479,LOC100053427,LOC100053382,LOC100053328,LOC100053284,LOC100053236,LOC100053185,LOC100053134,LOC100053085,LOC100053045,LOC100052992,LOC100146502,LOC100052946,LOC100146388,LOC100052842,LOC100147383,LOC100052788,LOC100052732,LOC100050399,LOC100052683,LOC100052629,LOC100052515,LOC100052454,LOC100052398,LOC100052341,LOC100052217,LOC100052157,LOC100052096,LOC100052037,LOC100146189,LOC100051977,LOC100051914,LOC100146810,LOC100050328,LOC100051731,LOC100051664,LOC100051592,LOC100051520,LOC100051452,LOC100051381,LOC100050265,LOC100630831
12	12524489	13401991	877503	4	1	LOC100146383,LOC100054355,LOC100054307,LOC100054258,LOC100054215,LOC100054169,LOC100054120,LOC100054065,LOC100054021,LOC100050556,LOC100053872,LOC100050476,LOC100053820,LOC100053768,LOC100629822,LOC100053672,LOC100053625,LOC100053568,LOC100053527,LOC100147669,LOC100053479,LOC100053427,LOC100053382,LOC100053328,LOC100053284,LOC100053236,LOC100053185,LOC100053134,LOC100053085,LOC100053045,LOC100052992,LOC100146502,LOC100052946,LOC100146388,LOC100052842,LOC100147383,LOC100052788,LOC100052732,LOC100050399,LOC100052683,LOC100052629,LOC100052515,LOC100052454,LOC100052398,LOC100052341,LOC100052217,LOC100052157,LOC100052096,LOC100052037,LOC100146189,LOC100051977,LOC100051914,LOC100146810,LOC100050328,LOC100051731,LOC100051664,LOC100051592,LOC100051520,LOC100051452,LOC100051381,LOC100050265,LOC100630831

Chrom	Start	End	Size	Cn	Samples (n)	Genes
12	12524489	13439146	914658	3	1	LOC100054498,LOC100054453,LOC100146383,LOC100054355,LOC100054307,LOC100054258,LOC100054215,LOC100054169,LOC100054120,LOC100054065,LOC100054021,LOC100050556,LOC100053872,LOC100050476,LOC100053820,LOC100053768,LOC100629822,LOC100053672,LOC100053625,LOC100053568,LOC100053527,LOC100147669,LOC100053479,LOC100053427,LOC100053382,LOC100053328,LOC100053284,LOC100053236,LOC100053185,LOC100053134,LOC100053085,LOC100053045,LOC100052992,LOC100146502,LOC100052946,LOC100146388,LOC100052842,LOC100147383,LOC100052788,LOC100052732,LOC100050399,LOC100052683,LOC100052629,LOC100052515,LOC100052454,LOC100052398,LOC100052341,LOC100052217,LOC100052157,LOC100052096,LOC100052037,LOC100146189,LOC100051977,LOC100051914,LOC100146810,LOC100050328,LOC100051731,LOC100051664,LOC100051592,LOC100051520,LOC100051452,LOC100051381,LOC100050265,LOC100630831
12	12524489	13488187	963699	3	1	LOC100054673,LOC100054627,LOC100054586,LOC100054541,LOC100054498,LOC100054453,LOC100146383,LOC100054355,LOC100054307,LOC100054258,LOC100054215,LOC100054169,LOC100054120,LOC100054065,LOC100054021,LOC100050556,LOC100053872,LOC100050476,LOC100053820,LOC100053768,LOC100629822,LOC100053672,LOC100053625,LOC100053568,LOC100053527,LOC100147669,LOC100053479,LOC100053427,LOC100053382,LOC100053328,LOC100053284,LOC100053236,LOC100053185,LOC100053134,LOC100053085,LOC100053045,LOC100052992,LOC100146502,LOC100052946,LOC100146388,LOC100052842,LOC100147383,LOC100052788,LOC100052732,LOC100050399,LOC100052683,LOC100052629,LOC100052515,LOC100052454,LOC100052398,LOC100052341,LOC100052217,LOC100052157,LOC100052096,LOC100052037,LOC100146189,LOC100051977,LOC100051914,LOC100146810,LOC100050328,LOC100051731,LOC100051664,LOC100051592,LOC100051520,LOC100051452,LOC100051381,LOC100050265,LOC100630831
12	12524489	13573356	1048868	3	2	LOC100147187,LOC100054954,LOC100054906,LOC100054858,LOC100054773,LOC100054722,LOC100054673,LOC100054627,LOC100054586,LOC100054541,LOC100054498,LOC100054453,LOC100146383,LOC100054355,LOC100054307,LOC100054258,LOC100054215,LOC100054169,LOC100054120,LOC100054065,LOC100054021,LOC100050556,LOC100053872,LOC100050476,LOC100053820,LOC100053768,LOC100629822,LOC100053672,LOC100053625,LOC100053568,LOC100053527,LOC100147669,LOC100053479,LOC100053427,LOC100053382,LOC100053328,LOC100053284,LOC100053236,LOC100053185,LOC100053134,LOC100053085,LOC100053045,LOC100052992,LOC100146502,LOC100052946,LOC100146388,LOC100052842,LOC100147383,LOC100052788,LOC100052732,LOC100050399,LOC100052683,LOC100052629,LOC100052515,LOC100052454,LOC100052398,LOC100052341,LOC100052217,LOC100052157,LOC100052096,LOC100052037,LOC100146189,LOC100051977,LOC100051914,LOC100146810,LOC100050328,LOC100051731,LOC100051664,LOC100051592,LOC100051520,LOC100051452,LOC100051381,LOC100050265,LOC100630831
12	12524489	13678953	1154465	1,3	1,4	LOC100055353,LOC100055309,LOC100055273,LOC100055224,LOC100055177,LOC100055129,LOC100055083,LOC100052898,LOC100147187,LOC100054954,LOC100054906,LOC100054858,LOC100054773,LOC100054722,LOC100054673,LOC100054627,LOC100054586,LOC100054541,LOC100054498,LOC100054453,LOC100146383,LOC100054355,LOC100054307,LOC100054258,LOC100054215,LOC100054169,LOC100054120,LOC100054065,LOC100054021,LOC100050556,LOC100053872,LOC100050476,LOC100053820,LOC100053768,LOC100629822,LOC100053672,LOC100053625,LOC100053568,LOC100053527,LOC100147669,LOC100053479,LOC100053427,LOC100053382,LOC100053328,LOC100053284,LOC100053236,LOC100053185,LOC100053134,LOC100053085,LOC100053045,LOC100052992,LOC100146502,LOC100052946,LOC100146388,LOC100052842,LOC100147383,LOC100052788,LOC100052732,LOC100050399,LOC100052683,LOC100052629,LOC100052515,LOC100052454,LOC100052398,LOC100052341,LOC100052217,LOC100052157,LOC100052096,LOC100052037,LOC100146189,LOC100051977,LOC100051914,LOC100146810,LOC100050328,LOC100051731,LOC100051664,LOC100051592,LOC100051520,LOC100051452,LOC100051381,LOC100050265,LOC100630831
12	12524489	13946509	1422021	3	1	LOC100056146,LOC100630788,LOC100629923,LOC100056026,LOC100055988,LOC100146992,LOC100055947,LOC100147569,LOC100146290,LOC100055908,LOC100055867,LOC100055780,LOC100630577,LOC100055646,LOC100055601,LOC100055561,LOC100055521,LOC100055478,LOC100055435,LOC100055397,LOC100055353,LOC100055309,LOC100055273,LOC100055224,LOC100055177,LOC100055129,LOC100055083,LOC100052898,LOC100147187,LOC100054954,LOC100054906,LOC100054858,LOC100054773,LOC100054722,LOC100054673,LOC100054627,LOC100054586,LOC100054541,LOC100054498,LOC100054453,LOC100146383,LOC100054355,LOC100054307,LOC100054258,LOC100054215,LOC100054169,LOC100054120,LOC100054065,LOC100054021,LOC100050556,LOC100053872,LOC100050476,LOC100053820,LOC100053768,LOC100629822,LOC100053672,LOC100053625,LOC100053568,LOC100053527,LOC100147669,LOC100053479,LOC100053427,LOC100053382,LOC100053328,LOC100053284,LOC100053236,LOC100053185,LOC100053134,LOC100053085,LOC100053045,LOC100052992,LOC100146502,LOC100052946,LOC100146388,LOC100052842,LOC100147383,LOC100052788,LOC100052732,LOC100050399,LOC100052683,LOC100052629,LOC100052515,LOC100052454,LOC100052398,LOC100052341,LOC100052217,LOC100052157,LOC100052096,LOC100052037,LOC100146189,LOC100051977,LOC100051914,LOC100146810,LOC100050328,LOC100051731,LOC100051664,LOC100051592,LOC100051520,LOC100051452,LOC100051381,LOC100050265,LOC100630831
12	12524489	14128309	1603821	3	6	LOC100056626,LOC100056578,LOC100056531,LOC100056488,LOC100629158,LOC100050633,LOC100056395,LOC100056356,LOC100056314,LOC100056273,LOC100056235,LOC100056187,LOC100056146,LOC100630788,LOC100629923,LOC100056026,LOC100055988,LOC100146992,LOC100055947,LOC100147569,LOC100146290,LOC100055908,LOC100055867,LOC100055780,LOC100630577,LOC100055646,LOC100055601,LOC100055561,LOC100055521,LOC100055478,LOC100055435,LOC100055397,LOC100055353,LOC100055309,LOC100055273,LOC100055224,LOC100055177,LOC100055129,LOC100055083,LOC100052898,LOC100147187,LOC100054954,LOC100054906,LOC100054858,LOC100054773,LOC100054722,LOC100054673,LOC100054627,LOC100054586,LOC100054541,LOC100054498,LOC100054453,LOC100146383,LOC100054355,LOC100054307,LOC100054258,LOC100054215,LOC100054169,LOC100054120,LOC100054065,LOC100054021,LOC100050556,LOC100053872,LOC100050476,LOC100053820,LOC100053768,LOC100629822,LOC100053672,LOC100053625,LOC100053568,LOC100053527,LOC100147669,LOC100053479,LOC100053427,LOC100053382,LOC100053328,LOC100053284,LOC100053236,LOC100053185,LOC100053134,LOC100053085,LOC100053045,LOC100052992,LOC100146502,LOC100052946,LOC100146388,LOC100052842,LOC100147383,LOC100052788,LOC100052732,LOC100050399,LOC100052683,LOC100052629,LOC100052515,LOC100052454,LOC100052398,LOC100052341,LOC100052217,LOC100052157,LOC100052096,LOC100052037,LOC100146189,LOC100051977,LOC100051914,LOC100146810,LOC100050328,LOC100051731,LOC100051664,LOC100051592,LOC100051520,LOC100051452,LOC100051381,LOC100050265,LOC100630831

Chrom	Start	End	Size	Cn	Samples (n)	Genes
12	12524489	14297056	1772568	1	2	LOC100056869,LOC100146809,LOC100067978,LOC100067957,LOC100056826,LOC100056793,LOC100056755,LOC100147000,LOC100056708,LOC100056626,LOC100056578,LOC100056531,LOC100056488,LOC100629158,LOC100050633,LOC100056395,LOC100056356,LOC100056314,LOC100056273,LOC100056235,LOC100056187,LOC100056146,LOC100630788,LOC100629923,LOC100056026,LOC100055988,LOC100146992,LOC100055947,LOC100147569,LOC100146290,LOC100055908,LOC100055867,LOC100055780,LOC100630577,LOC100055646,LOC100055601,LOC100055561,LOC100055521,LOC100055478,LOC100055435,LOC100055397,LOC100055353,LOC100055309,LOC100055273,LOC100055224,LOC100055177,LOC100055129,LOC100055083,LOC100052898,LOC100147187,LOC100054954,LOC100054906,LOC100054858,LOC100054773,LOC100054722,LOC100054673,LOC100054627,LOC100054586,LOC100054541,LOC100054498,LOC100054453,LOC100146383,LOC100054355,LOC100054307,LOC100054258,LOC100054215,LOC100054169,LOC100054120,LOC100054065,LOC100054021,LOC100050556,LOC100053872,LOC100050476,LOC100053820,LOC100053768,LOC100629822,LOC100053672,LOC100053625,LOC100053568,LOC100053527,LOC100147669,LOC100053479,LOC100053427,LOC100053382,LOC100053328,LOC100053284,LOC100053236,LOC100053185,LOC100053134,LOC100053085,LOC100053045,LOC100052992,LOC100146502,LOC100052946,LOC100146388,LOC100052842,LOC100147383,LOC100052788,LOC100052732,LOC100050399,LOC100052683,LOC100052629,LOC100052515,LOC100052454,LOC100052398,LOC100052341,LOC100052217,LOC100052157,LOC100052096,LOC100052037,LOC100146189,LOC100051977,LOC100051914,LOC100146810,LOC100050328,LOC100051731,LOC100051664,LOC100051592,LOC100051520,LOC100051452,LOC100051381,LOC100050265,LOC100630831
12	12524489	14354314	1829826	3	2	LOC100056947,LOC100056906,LOC100056869,LOC100146809,LOC100067978,LOC100067957,LOC100056826,LOC100056793,LOC100056755,LOC100147000,LOC100056708,LOC100056626,LOC100056578,LOC100056531,LOC100056488,LOC100629158,LOC100050633,LOC100056395,LOC100056356,LOC100056314,LOC100056273,LOC100056235,LOC100056187,LOC100056146,LOC100630788,LOC100629923,LOC100056026,LOC100055988,LOC100146992,LOC100055947,LOC100147569,LOC100146290,LOC100055908,LOC100055867,LOC100055780,LOC100630577,LOC100055646,LOC100055601,LOC100055561,LOC100055521,LOC100055478,LOC100055435,LOC100055397,LOC100055353,LOC100055309,LOC100055273,LOC100055224,LOC100055177,LOC100055129,LOC100055083,LOC100052898,LOC100147187,LOC100054954,LOC100054906,LOC100054858,LOC100054773,LOC100054722,LOC100054673,LOC100054627,LOC100054586,LOC100054541,LOC100054498,LOC100054453,LOC100146383,LOC100054355,LOC100054307,LOC100054258,LOC100054215,LOC100054169,LOC100054120,LOC100054065,LOC100054021,LOC100050556,LOC100053872,LOC100050476,LOC100053820,LOC100053768,LOC100629822,LOC100053672,LOC100053625,LOC100053568,LOC100053527,LOC100147669,LOC100053479,LOC100053427,LOC100053382,LOC100053328,LOC100053284,LOC100053236,LOC100053185,LOC100053134,LOC100053085,LOC100053045,LOC100052992,LOC100146502,LOC100052946,LOC100146388,LOC100052842,LOC100147383,LOC100052788,LOC100052732,LOC100050399,LOC100052683,LOC100052629,LOC100052515,LOC100052454,LOC100052398,LOC100052341,LOC100052217,LOC100052157,LOC100052096,LOC100052037,LOC100146189,LOC100051977,LOC100051914,LOC100146810,LOC100050328,LOC100051731,LOC100051664,LOC100051592,LOC100051520,LOC100051452,LOC100051381,LOC100050265,LOC100630831
12	12524489	14391372	1866884	0,1,3,4	19,57,11,1	LOC100057026,LOC100050714,LOC100056989,LOC100056947,LOC100056906,LOC100056869,LOC100146809,LOC100067978,LOC100067957,LOC100056826,LOC100056793,LOC100056755,LOC100147000,LOC100056708,LOC100056626,LOC100056578,LOC100056531,LOC100056488,LOC100629158,LOC100050633,LOC100056395,LOC100056356,LOC100056314,LOC100056273,LOC100056235,LOC100056187,LOC100056146,LOC100630788,LOC100629923,LOC100056026,LOC100055988,LOC100146992,LOC100055947,LOC100147569,LOC100146290,LOC100055908,LOC100055867,LOC100055780,LOC100630577,LOC100055646,LOC100055601,LOC100055561,LOC100055521,LOC100055478,LOC100055435,LOC100055397,LOC100055353,LOC100055309,LOC100055273,LOC100055224,LOC100055177,LOC100055129,LOC100055083,LOC100052898,LOC100147187,LOC100054954,LOC100054906,LOC100054858,LOC100054773,LOC100054722,LOC100054673,LOC100054627,LOC100054586,LOC100054541,LOC100054498,LOC100054453,LOC100146383,LOC100054355,LOC100054307,LOC100054258,LOC100054215,LOC100054169,LOC100054120,LOC100054065,LOC100054021,LOC100050556,LOC100053872,LOC100050476,LOC100053820,LOC100053768,LOC100629822,LOC100053672,LOC100053625,LOC100053568,LOC100053527,LOC100147669,LOC100053479,LOC100053427,LOC100053382,LOC100053328,LOC100053284,LOC100053236,LOC100053185,LOC100053134,LOC100053085,LOC100053045,LOC100052992,LOC100146502,LOC100052946,LOC100146388,LOC100052842,LOC100147383,LOC100052788,LOC100052732,LOC100050399,LOC100052683,LOC100052629,LOC100052515,LOC100052454,LOC100052398,LOC100052341,LOC100052217,LOC100052157,LOC100052096,LOC100052037,LOC100146189,LOC100051977,LOC100051914,LOC100146810,LOC100050328,LOC100051731,LOC100051664,LOC100051592,LOC100051520,LOC100051452,LOC100051381,LOC100050265,LOC100630831
12	12524489	14587232	2062744	1	9	LOC100057472,LOC100057433,LOC100057393,LOC100057352,LOC100057310,LOC100147286,LOC100146990,LOC100057269,LOC100057228,LOC100057191,LOC100057110,LOC100146600,LOC100057026,LOC100050714,LOC100056989,LOC100056947,LOC100056906,LOC100056869,LOC100146809,LOC100067978,LOC100067957,LOC100056826,LOC100056793,LOC100056755,LOC100147000,LOC100056708,LOC100056626,LOC100056578,LOC100056531,LOC100056488,LOC100629158,LOC100050633,LOC100056395,LOC100056356,LOC100056314,LOC100056273,LOC100056235,LOC100056187,LOC100056146,LOC100630788,LOC100629923,LOC100056026,LOC100055988,LOC100146992,LOC100055947,LOC100147569,LOC100146290,LOC100055908,LOC100055867,LOC100055780,LOC100630577,LOC100055646,LOC100055601,LOC100055561,LOC100055521,LOC100055478,LOC100055435,LOC100055397,LOC100055353,LOC100055309,LOC100055273,LOC100055224,LOC100055177,LOC100055129,LOC100055083,LOC100052898,LOC100147187,LOC100054954,LOC100054906,LOC100054858,LOC100054773,LOC100054722,LOC100054673,LOC100054627,LOC100054586,LOC100054541,LOC100054498,LOC100054453,LOC100146383,LOC100054355,LOC100054307,LOC100054258,LOC100054215,LOC100054169,LOC100054120,LOC100054065,LOC100054021,LOC100050556,LOC100053872,LOC100050476,LOC100053820,LOC100053768,LOC100629822,LOC100053672,LOC100053625,LOC100053568,LOC100053527,LOC100147669,LOC100053479,LOC100053427,LOC100053382,LOC100053328,LOC100053284,LOC100053236,LOC100053185,LOC100053134,LOC100053085,LOC100053045,LOC100052992,LOC100146502,LOC100052946,LOC100146388,LOC100052842,LOC100147383,LOC100052788,LOC100052732,LOC100050399,LOC100052683,LOC100052629,LOC100052515,LOC100052454,LOC100052398,LOC100052341,LOC100052217,LOC100052157,LOC100052096,LOC100052037,LOC100146189,LOC100051977,LOC100051914,LOC100146810,LOC100050328,LOC100051731,LOC100051664,LOC100051592,LOC100051520,LOC100051452,LOC100051381,LOC100050265,LOC100630831

Chrom	Start	End	Size	Cn	Samples (n)	Genes
12	12524489	14747048	2222560	3	1	LOC100057945,LOC100057894,LOC100057845,LOC100057804,LOC100057756,LOC100057709,LOC100057670,LOC100057589,LOC100057547,LOC100057513,LOC100057472,LOC100057433,LOC100057393,LOC100057352,LOC100057310,LOC100147286,LOC100146990,LOC100057269,LOC100057228,LOC100057191,LOC100057110,LOC100146600,LOC100057026,LOC100050714,LOC100056989,LOC100056947,LOC100056906,LOC100056869,LOC100146809,LOC100067978,LOC100067957,LOC100056826,LOC100056793,LOC100056755,LOC100147000,LOC100056708,LOC100056626,LOC100056578,LOC100056531,LOC100056488,LOC100629158,LOC100050633,LOC100056395,LOC100056356,LOC100056314,LOC100056273,LOC100056235,LOC100056187,LOC100056146,LOC100630788,LOC100629923,LOC100056026,LOC100055988,LOC100146992,LOC100055947,LOC100147569,LOC100146290,LOC100055908,LOC100055867,LOC100055780,LOC100630577,LOC100055646,LOC100055601,LOC100055561,LOC100055521,LOC100055478,LOC100055435,LOC100055397,LOC100055353,LOC100055309,LOC100055273,LOC100055224,LOC100055177,LOC100055129,LOC100055083,LOC100052898,LOC100147187,LOC100054954,LOC100054906,LOC100054858,LOC100054773,LOC100054722,LOC100054673,LOC100054627,LOC100054586,LOC100054541,LOC100054498,LOC100054453,LOC100146383,LOC100054355,LOC100054307,LOC100054258,LOC100054215,LOC100054169,LOC100054120,LOC100054065,LOC100054021,LOC100050556,LOC100053872,LOC100050476,LOC100053820,LOC100053768,LOC100629822,LOC100053672,LOC100053625,LOC100053568,LOC100053527,LOC100147669,LOC100053479,LOC100053427,LOC100053382,LOC100053328,LOC100053284,LOC100053236,LOC100053185,LOC100053134,LOC100053085,LOC100053045,LOC100052992,LOC100146502,LOC100052946,LOC100146388,LOC100052842,LOC100147383,LOC100052788,LOC100052732,LOC100050399,LOC100052683,LOC100052629,LOC100052515,LOC100052454,LOC100052398,LOC100052341,LOC100052217,LOC100052157,LOC100052096,LOC100052037,LOC100146189,LOC100051977,LOC100051914,LOC100146810,LOC100050328,LOC100051731,LOC100051664,LOC100051592,LOC100051520,LOC100051452,LOC100051381,LOC100050265,LOC100630831
12	12524489	14777981	2253493	1,3,4	12,10,3	LOC100058077,LOC100147282,LOC100058036,LOC100057989,LOC100057945,LOC100057894,LOC100057845,LOC100057804,LOC100057756,LOC100057709,LOC100057670,LOC100057589,LOC100057547,LOC100057513,LOC100057472,LOC100057433,LOC100057393,LOC100057352,LOC100057310,LOC100147286,LOC100146990,LOC100057269,LOC100057228,LOC100057191,LOC100057110,LOC100146600,LOC100057026,LOC100050714,LOC100056989,LOC100056947,LOC100056906,LOC100056869,LOC100146809,LOC100067978,LOC100067957,LOC100056826,LOC100056793,LOC100056755,LOC100147000,LOC100056708,LOC100056626,LOC100056578,LOC100056531,LOC100056488,LOC100629158,LOC100050633,LOC100056395,LOC100056356,LOC100056314,LOC100056273,LOC100056235,LOC100056187,LOC100056146,LOC100630788,LOC100629923,LOC100056026,LOC100055988,LOC100146992,LOC100055947,LOC100147569,LOC100146290,LOC100055908,LOC100055867,LOC100055780,LOC100630577,LOC100055646,LOC100055601,LOC100055561,LOC100055521,LOC100055478,LOC100055435,LOC100055397,LOC100055353,LOC100055309,LOC100055273,LOC100055224,LOC100055177,LOC100055129,LOC100055083,LOC100052898,LOC100147187,LOC100054954,LOC100054906,LOC100054858,LOC100054773,LOC100054722,LOC100054673,LOC100054627,LOC100054586,LOC100054541,LOC100054498,LOC100054453,LOC100146383,LOC100054355,LOC100054307,LOC100054258,LOC100054215,LOC100054169,LOC100054120,LOC100054065,LOC100054021,LOC100050556,LOC100053872,LOC100050476,LOC100053820,LOC100053768,LOC100629822,LOC100053672,LOC100053625,LOC100053568,LOC100053527,LOC100147669,LOC100053479,LOC100053427,LOC100053382,LOC100053328,LOC100053284,LOC100053236,LOC100053185,LOC100053134,LOC100053085,LOC100053045,LOC100052992,LOC100146502,LOC100052946,LOC100146388,LOC100052842,LOC100147383,LOC100052788,LOC100052732,LOC100050399,LOC100052683,LOC100052629,LOC100052515,LOC100052454,LOC100052398,LOC100052341,LOC100052217,LOC100052157,LOC100052096,LOC100052037,LOC100146189,LOC100051977,LOC100051914,LOC100146810,LOC100050328,LOC100051731,LOC100051664,LOC100051592,LOC100051520,LOC100051452,LOC100051381,LOC100050265,LOC10063083
12	12524489	15073209	2548721	3	1	LOC100058472,LOC100058429,LOC100058385,LOC100058346,LOC100146506,LOC100058253,LOC100147086,LOC100630034,LOC100058212,LOC100058168,LOC100058123,LOC100058077,LOC100147282,LOC100058036,LOC100057989,LOC100057945,LOC100057894,LOC100057845,LOC100057804,LOC100057756,LOC100057709,LOC100057670,LOC100057589,LOC100057547,LOC100057513,LOC100057472,LOC100057433,LOC100057393,LOC100057352,LOC100057310,LOC100147286,LOC100146990,LOC100057269,LOC100057228,LOC100057191,LOC100057110,LOC100146600,LOC100057026,LOC100050714,LOC100056989,LOC100056947,LOC100056906,LOC100056869,LOC100146809,LOC100067978,LOC100067957,LOC100056826,LOC100056793,LOC100056755,LOC100147000,LOC100056708,LOC100056626,LOC100056578,LOC100056531,LOC100056488,LOC100629158,LOC100050633,LOC100056395,LOC100056356,LOC100056314,LOC100056273,LOC100056235,LOC100056187,LOC100056146,LOC100630788,LOC100629923,LOC100056026,LOC100055988,LOC100146992,LOC100055947,LOC100147569,LOC100146290,LOC100055908,LOC100055867,LOC100055780,LOC100630577,LOC100055646,LOC100055601,LOC100055561,LOC100055521,LOC100055478,LOC100055435,LOC100055397,LOC100055353,LOC100055309,LOC100055273,LOC100055224,LOC100055177,LOC100055129,LOC100055083,LOC100052898,LOC100147187,LOC100054954,LOC100054906,LOC100054858,LOC100054773,LOC100054722,LOC100054673,LOC100054627,LOC100054586,LOC100054541,LOC100054498,LOC100054453,LOC100146383,LOC100054355,LOC100054307,LOC100054258,LOC100054215,LOC100054169,LOC100054120,LOC100054065,LOC100054021,LOC100050556,LOC100053872,LOC100050476,LOC100053820,LOC100053768,LOC100629822,LOC100053672,LOC100053625,LOC100053568,LOC100053527,LOC100147669,LOC100053479,LOC100053427,LOC100053382,LOC100053328,LOC100053284,LOC100053236,LOC100053185,LOC100053134,LOC100053085,LOC100053045,LOC100052992,LOC100146502,LOC100052946,LOC100146388,LOC100052842,LOC100147383,LOC100052788,LOC100052732,LOC100050399,LOC100052683,LOC100052629,LOC100052515,LOC100052454,LOC100052398,LOC100052341,LOC100052217,LOC100052157,LOC100052096,LOC100052037,LOC100146189,LOC10005197

Chrom	Start	End	Size	Cn	Samples (n)	Genes
12	12524489	16525629	4001141	3	1	LOC100066553,LOC100066503,LOC100066447,LOC100066417,LOC100066361,LOC100066225,LOC100066190,LOC100066164,LOC100630861,LOC100066108,LOC100066068,LOC100147059,LOC100066040,LOC100062734,LOC100066011,LOC100065987,LOC100146657,LOC100065959,LOC100065934,LOC100147543,LOC100065911,LOC100065887,LOC100065858,LOC100065831,LOC100065803,LOC100065755,LOC100065735,LOC100065693,LOC100065674,LOC100065655,LOC100065634,LOC100146350,LOC100065610,LOC100065591,LOC100065549,LOC100146953,LOC100065500,LOC100065474,LOC100062694,LOC100065451,LOC100065423,LOC100065402,LOC100065380,LOC100065349,LOC100065319,LOC100065288,LOC100146469,LOC100065263,LOC100147060,LOC100065230,LOC100065201,LOC100065168,LOC100065136,LOC100065111,LOC100065079,LOC100065048,LOC100065019,LOC100630301,LOC100064956,LOC100064924,LOC100062657,LOC100064900,LOC100064874,LOC100064848,LOC100064820,LOC100064792,LOC100064769,LOC100064716,LOC100064690,LOC100062590,LOC100062626,LOC100064662,LOC100630007,LOC100064597,LOC100064562,LOC100058472,LOC100058429,LOC100058385,LOC100058346,LOC100146506,LOC100058253,LOC100147086,LOC100630034,LOC100058212,LOC100058168,LOC100058123,LOC100058077,LOC100147282,LOC100058036,LOC100057989,LOC100057945,LOC100057894,LOC100057845,LOC100057804,LOC100057756,LOC100057709,LOC100057670,LOC100057589,LOC100057547,LOC100057513,LOC100057472,LOC100057433,LOC100057393,LOC100057352,LOC100057310,LOC100147286,LOC100146990,LOC100057269,LOC100057228,LOC100057191,LOC100057110,LOC100146600,LOC100057026,LOC100050714,LOC100056989,LOC100056947,LOC100056906,LOC100056869,LOC100146809,LOC100067978,LOC100067957,LOC100056826,LOC100056793,LOC100056755,LOC100147000,LOC100056708,LOC100056626,LOC100056578,LOC100056531,LOC100056488,LOC100629158,LOC100050633,LOC100056395,LOC100056356,LOC100056314,LOC100056273,LOC100056235,LOC100056187,LOC100056146,LOC100630788,LOC100629923,LOC100056026,LOC100055988,LOC100146992,LOC100055947,LOC100147569,LOC100146290,LOC100055908,LOC100055867,LOC100055780,LOC100630577,LOC100055646,LOC100055601,LOC10005556
12	12594142	13379725	785584	3	2	LOC100054258,LOC100054215,LOC100054169,LOC100054120,LOC100054065,LOC100054021,LOC100050556,LOC100053872,LOC100050476,LOC100053820,LOC100053768,LOC100629822,LOC100053672,LOC100053625,LOC100053568,LOC100053527,LOC100147669,LOC100053479,LOC100053427,LOC100053382,LOC100053328,LOC100053284,LOC100053236,LOC100053185,LOC100053134,LOC100053085,LOC100053045,LOC100052992,LOC100146502,LOC100052946,LOC100146388,LOC100052842,LOC100147383,LOC100052788,LOC100052732,LOC100050399,LOC100052683,LOC100052629,LOC100052515,LOC100052454,LOC100052398,LOC100052341,LOC100052217,LOC100052157,LOC100052096,LOC100052037,LOC100146189,LOC100051977,LOC100051914,LOC100146810,LOC100050328,LOC100051731,LOC100051664
12	12594142	14354314	1760173	3	1	LOC100056947,LOC100056906,LOC100056869,LOC100146809,LOC100067978,LOC100067957,LOC100056826,LOC100056793,LOC100056755,LOC100147000,LOC100056708,LOC100056626,LOC100056578,LOC100056531,LOC100056488,LOC100629158,LOC100050633,LOC100056395,LOC100056356,LOC100056314,LOC100056273,LOC100056235,LOC100056187,LOC100056146,LOC100630788,LOC100629923,LOC100056026,LOC100055988,LOC100146992,LOC100055947,LOC100147569,LOC100146290,LOC100055908,LOC100055867,LOC100055780,LOC100630577,LOC100055646,LOC100055601,LOC100055561,LOC100055521,LOC100055478,LOC100055435,LOC100055397,LOC100055353,LOC100055309,LOC100055273,LOC100055224,LOC100055177,LOC100055129,LOC100055083,LOC100052898,LOC100147187,LOC100054954,LOC100054906,LOC100054858,LOC100054773,LOC100054722,LOC100054673,LOC100054627,LOC100054586,LOC100054541,LOC100054498,LOC100054453,LOC100146383,LOC100054355,LOC100054307,LOC100054258,LOC100054215,LOC100054169,LOC100054120,LOC100054065,LOC100054021,LOC100050556,LOC100053872,LOC100050476,LOC100053820,LOC100053768,LOC100629822,LOC100053672,LOC100053625,LOC100053568,LOC100053527,LOC100147669,LOC100053479,LOC100053427,LOC100053382,LOC100053328,LOC100053284,LOC100053236,LOC100053185,LOC100053134,LOC100053085,LOC100053045,LOC100052992,LOC100146502,LOC100052946,LOC100146388,LOC100052842,LOC100147383,LOC100052788,LOC100052732,LOC100050399,LOC100052683,LOC100052629,LOC100052515,LOC100052454,LOC100052398,LOC100052341,LOC100052217,LOC100052157,LOC100052096,LOC100052037,LOC100146189,LOC100051977,LOC100051914,LOC100146810,LOC100050328,LOC100051731,LOC100051664
12	12594142	14391372	1797231	1,3	1,2	LOC100057026,LOC100050714,LOC100056989,LOC100056947,LOC100056906,LOC100056869,LOC100146809,LOC100067978,LOC100067957,LOC100056826,LOC100056793,LOC100056755,LOC100147000,LOC100056708,LOC100056626,LOC100056578,LOC100056531,LOC100056488,LOC100629158,LOC100050633,LOC100056395,LOC100056356,LOC100056314,LOC100056273,LOC100056235,LOC100056187,LOC100056146,LOC100630788,LOC100629923,LOC100056026,LOC100055988,LOC100146992,LOC100055947,LOC100147569,LOC100146290,LOC100055908,LOC100055867,LOC100055780,LOC100630577,LOC100055646,LOC100055601,LOC100055561,LOC100055521,LOC100055478,LOC100055435,LOC100055397,LOC100055353,LOC100055309,LOC100055273,LOC100055224,LOC100055177,LOC100055129,LOC100055083,LOC100052898,LOC100147187,LOC100054954,LOC100054906,LOC100054858,LOC100054773,LOC100054722,LOC100054673,LOC100054627,LOC100054586,LOC100054541,LOC100054498,LOC100054453,LOC100146383,LOC100054355,LOC100054307,LOC100054258,LOC100054215,LOC100054169,LOC100054120,LOC100054065,LOC100054021,LOC100050556,LOC100053872,LOC100050476,LOC100053820,LOC100053768,LOC100629822,LOC100053672,LOC100053625,LOC100053568,LOC100053527,LOC100147669,LOC100053479,LOC100053427,LOC100053382,LOC100053328,LOC100053284,LOC100053236,LOC100053185,LOC100053134,LOC100053085,LOC100053045,LOC100052992,LOC100146502,LOC100052946,LOC100146388,LOC100052842,LOC100147383,LOC100052788,LOC100052732,LOC100050399,LOC100052683,LOC100052629,LOC100052515,LOC100052454,LOC100052398,LOC100052341,LOC100052217,LOC100052157,LOC100052096,LOC100052037,LOC100146189,LOC100051977,LOC100051914,LOC100146810,LOC100050328,LOC100051731,LOC100051664
12	12829176	12858531	29356	1	2	LOC100052732,LOC100050399,LOC100052683
12	12829176	13149957	320782	1	4	LOC100053672,LOC100053625,LOC100053568,LOC100053527,LOC100147669,LOC100053479,LOC100053427,LOC100053382,LOC100053328,LOC100053284,LOC100053236,LOC100053185,LOC100053134,LOC100053085,LOC100053045,LOC100052992,LOC100146502,LOC100052946,LOC100146388,LOC100052842,LOC100147383,LOC100052788,LOC100052732,LOC100050399,LOC100052683
12	12829176	13170959	341784	4	1	LOC100053820,LOC100053768,LOC100629822,LOC100053672,LOC100053625,LOC100053568,LOC100053527,LOC100147669,LOC100053479,LOC100053427,LOC100053382,LOC100053328,LOC100053284,LOC100053236,LOC100053185,LOC100053134,LOC100053085,LOC100053045,LOC100052992,LOC100146502,LOC100052946,LOC100146388,LOC100052842,LOC100147383,LOC100052788,LOC100052732,LOC100050399,LOC100052683

Chrom	Start	End	Size	Cn	Samples (n)	Genes
12	12829176	13401991	572816	3	2	LOC100146383,LOC100054355,LOC100054307,LOC100054258,LOC100054215,LOC100054169,LOC100054120,LOC100054065,LOC100054021,LOC100050556,LOC100053872,LOC100050476,LOC100053820,LOC100053768,LOC100629822,LOC100053672,LOC100053625,LOC100053568,LOC100053527,LOC100147669,LOC100053479,LOC100053427,LOC100053382,LOC100053328,LOC100053284,LOC100053236,LOC100053185,LOC100053134,LOC100053085,LOC100053045,LOC100052992,LOC100146502,LOC100052946,LOC100146388,LOC100052842,LOC100147383,LOC100052788,LOC100052732,LOC100050399,LOC100052683
12	12829176	13422256	593081	3	1	LOC100054453,LOC100146383,LOC100054355,LOC100054307,LOC100054258,LOC100054215,LOC100054169,LOC100054120,LOC100054065,LOC100054021,LOC100050556,LOC100053872,LOC100050476,LOC100053820,LOC100053768,LOC100629822,LOC100053672,LOC100053625,LOC100053568,LOC100053527,LOC100147669,LOC100053479,LOC100053427,LOC100053382,LOC100053328,LOC100053284,LOC100053236,LOC100053185,LOC100053134,LOC100053085,LOC100053045,LOC100052992,LOC100146502,LOC100052946,LOC100146388,LOC100052842,LOC100147383,LOC100052788,LOC100052732,LOC100050399,LOC100052683
12	12829176	13573356	744181	1	1	LOC100147187,LOC100054954,LOC100054906,LOC100054858,LOC100054773,LOC100054722,LOC100054673,LOC100054627,LOC100054586,LOC100054541,LOC100054498,LOC100054453,LOC100146383,LOC100054355,LOC100054307,LOC100054258,LOC100054215,LOC100054169,LOC100054120,LOC100054065,LOC100054021,LOC100050556,LOC100053872,LOC100050476,LOC100053820,LOC100053768,LOC100629822,LOC100053672,LOC100053625,LOC100053568,LOC100053527,LOC100147669,LOC100053479,LOC100053427,LOC100053382,LOC100053328,LOC100053284,LOC100053236,LOC100053185,LOC100053134,LOC100053085,LOC100053045,LOC100052992,LOC100146502,LOC100052946,LOC100146388,LOC100052842,LOC100147383,LOC100052788,LOC100052732,LOC100050399,LOC100052683
12	12829176	13678953	849778	1	4	LOC100055353,LOC100055309,LOC100055273,LOC100055224,LOC100055177,LOC100055129,LOC100055083,LOC100052898,LOC100147187,LOC100054954,LOC100054906,LOC100054858,LOC100054773,LOC100054722,LOC100054673,LOC100054627,LOC100054586,LOC100054541,LOC100054498,LOC100054453,LOC100146383,LOC100054355,LOC100054307,LOC100054258,LOC100054215,LOC100054169,LOC100054120,LOC100054065,LOC100054021,LOC100050556,LOC100053872,LOC100050476,LOC100053820,LOC100053768,LOC100629822,LOC100053672,LOC100053625,LOC100053568,LOC100053527,LOC100147669,LOC100053479,LOC100053427,LOC100053382,LOC100053328,LOC100053284,LOC100053236,LOC100053185,LOC100053134,LOC100053085,LOC100053045,LOC100052992,LOC100146502,LOC100052946,LOC100146388,LOC100052842,LOC100147383,LOC100052788,LOC100052732,LOC100050399,LOC100052683
12	12829176	14128309	1299134	3,4	2,1	LOC100056626,LOC100056578,LOC100056531,LOC100056488,LOC100629158,LOC100050633,LOC100056395,LOC100056356,LOC100056314,LOC100056273,LOC100056235,LOC100056187,LOC100056146,LOC100630788,LOC100629923,LOC100056026,LOC100055988,LOC100146992,LOC100055947,LOC100147569,LOC100146290,LOC100055908,LOC100055867,LOC100055780,LOC100630577,LOC100055646,LOC100055601,LOC100055561,LOC100055521,LOC100055478,LOC100055435,LOC100055397,LOC100055353,LOC100055309,LOC100055273,LOC100055224,LOC100055177,LOC100055129,LOC100055083,LOC100052898,LOC100147187,LOC100054954,LOC100054906,LOC100054858,LOC100054773,LOC100054722,LOC100054673,LOC100054627,LOC100054586,LOC100054541,LOC100054498,LOC100054453,LOC100146383,LOC100054355,LOC100054307,LOC100054258,LOC100054215,LOC100054169,LOC100054120,LOC100054065,LOC100054021,LOC100050556,LOC100053872,LOC100050476,LOC100053820,LOC100053768,LOC100629822,LOC100053672,LOC100053625,LOC100053568,LOC100053527,LOC100147669,LOC100053479,LOC100053427,LOC100053382,LOC100053328,LOC100053284,LOC100053236,LOC100053185,LOC100053134,LOC100053085,LOC100053045,LOC100052992,LOC100146502,LOC100052946,LOC100146388,LOC100052842,LOC100147383,LOC100052788,LOC100052732,LOC100050399,LOC100052683
12	12829176	14297056	1467881	3	1	LOC100056869,LOC100146809,LOC100067978,LOC100067957,LOC100056826,LOC100056793,LOC100056755,LOC100147000,LOC100056708,LOC100056626,LOC100056578,LOC100056531,LOC100056488,LOC100629158,LOC100050633,LOC100056395,LOC100056356,LOC100056314,LOC100056273,LOC100056235,LOC100056187,LOC100056146,LOC100630788,LOC100629923,LOC100056026,LOC100055988,LOC100146992,LOC100055947,LOC100147569,LOC100146290,LOC100055908,LOC100055867,LOC100055780,LOC100630577,LOC100055646,LOC100055601,LOC100055561,LOC100055521,LOC100055478,LOC100055435,LOC100055397,LOC100055353,LOC100055309,LOC100055273,LOC100055224,LOC100055177,LOC100055129,LOC100055083,LOC100052898,LOC100147187,LOC100054954,LOC100054906,LOC100054858,LOC100054773,LOC100054722,LOC100054673,LOC100054627,LOC100054586,LOC100054541,LOC100054498,LOC100054453,LOC100146383,LOC100054355,LOC100054307,LOC100054258,LOC100054215,LOC100054169,LOC100054120,LOC100054065,LOC100054021,LOC100050556,LOC100053872,LOC100050476,LOC100053820,LOC100053768,LOC100629822,LOC100053672,LOC100053625,LOC100053568,LOC100053527,LOC100147669,LOC100053479,LOC100053427,LOC100053382,LOC100053328,LOC100053284,LOC100053236,LOC100053185,LOC100053134,LOC100053085,LOC100053045,LOC100052992,LOC100146502,LOC100052946,LOC100146388,LOC100052842,LOC100147383,LOC100052788,LOC100052732,LOC100050399,LOC100052683
12	12829176	14354314	1525139	3	2	LOC100056947,LOC100056906,LOC100056869,LOC100146809,LOC100067978,LOC100067957,LOC100056826,LOC100056793,LOC100056755,LOC100147000,LOC100056708,LOC100056626,LOC100056578,LOC100056531,LOC100056488,LOC100629158,LOC100050633,LOC100056395,LOC100056356,LOC100056314,LOC100056273,LOC100056235,LOC100056187,LOC100056146,LOC100630788,LOC100629923,LOC100056026,LOC100055988,LOC100146992,LOC100055947,LOC100147569,LOC100146290,LOC100055908,LOC100055867,LOC100055780,LOC100630577,LOC100055646,LOC100055601,LOC100055561,LOC100055521,LOC100055478,LOC100055435,LOC100055397,LOC100055353,LOC100055309,LOC100055273,LOC100055224,LOC100055177,LOC100055129,LOC100055083,LOC100052898,LOC100147187,LOC100054954,LOC100054906,LOC100054858,LOC100054773,LOC100054722,LOC100054673,LOC100054627,LOC100054586,LOC100054541,LOC100054498,LOC100054453,LOC100146383,LOC100054355,LOC100054307,LOC100054258,LOC100054215,LOC100054169,LOC100054120,LOC100054065,LOC100054021,LOC100050556,LOC100053872,LOC100050476,LOC100053820,LOC100053768,LOC100629822,LOC100053672,LOC100053625,LOC100053568,LOC100053527,LOC100147669,LOC100053479,LOC100053427,LOC100053382,LOC100053328,LOC100053284,LOC100053236,LOC100053185,LOC100053134,LOC100053085,LOC100053045,LOC100052992,LOC100146502,LOC100052946,LOC100146388,LOC100052842,LOC100147383,LOC100052788,LOC100052732,LOC100050399,LOC100052683

Chrom	Start	End	Size	Cn	Samples (n)	Genes
12	12829176	14391372	1562197	1,3	1,3	LOC100057026,LOC100050714,LOC100056989,LOC100056947,LOC100056906,LOC100056869,LOC100146809,LOC100067978,LOC100067957,LOC100056826,LOC100056793,LOC100056755,LOC100147000,LOC100056708,LOC100056626,LOC100056578,LOC100056531,LOC100056488,LOC100629158,LOC100050633,LOC100056395,LOC100056356,LOC100056314,LOC100056273,LOC100056235,LOC100056187,LOC100056146,LOC100630788,LOC100629923,LOC100056026,LOC100055988,LOC100146992,LOC100055947,LOC100147569,LOC100146290,LOC100055908,LOC100055867,LOC100055780,LOC100630577,LOC100055646,LOC100055601,LOC100055561,LOC100055521,LOC100055478,LOC100055435,LOC100055397,LOC100055353,LOC100055309,LOC100055273,LOC100055224,LOC100055177,LOC100055129,LOC100055083,LOC100052898,LOC100147187,LOC100054954,LOC100054906,LOC100054858,LOC100054773,LOC100054722,LOC100054673,LOC100054627,LOC100054586,LOC100054541,LOC100054498,LOC100054453,LOC100146383,LOC100054355,LOC100054307,LOC100054258,LOC100054215,LOC100054169,LOC100054120,LOC100054065,LOC100054021,LOC100050556,LOC100053872,LOC100050476,LOC100053820,LOC100053768,LOC100629822,LOC100053672,LOC100053625,LOC100053568,LOC100053527,LOC100147669,LOC100053479,LOC100053427,LOC100053382,LOC100053328,LOC100053284,LOC100053236,LOC100053185,LOC100053134,LOC100053085,LOC100053045,LOC100052992,LOC100146502,LOC100052946,LOC100146388,LOC100052842,LOC100147383,LOC100052788,LOC100052732,LOC100050399,LOC100052683
12	12829176	14587232	1758057	1	1	LOC100057472,LOC100057433,LOC100057393,LOC100057352,LOC100057310,LOC100147286,LOC100146990,LOC100057269,LOC100057228,LOC100057191,LOC100057110,LOC100146600,LOC100057026,LOC100050714,LOC100056989,LOC100056947,LOC100056906,LOC100056869,LOC100146809,LOC100067978,LOC100067957,LOC100056826,LOC100056793,LOC100056755,LOC100147000,LOC100056708,LOC100056626,LOC100056578,LOC100056531,LOC100056488,LOC100629158,LOC100050633,LOC100056395,LOC100056356,LOC100056314,LOC100056273,LOC100056235,LOC100056187,LOC100056146,LOC100630788,LOC100629923,LOC100056026,LOC100055988,LOC100146992,LOC100055947,LOC100147569,LOC100146290,LOC100055908,LOC100055867,LOC100055780,LOC100630577,LOC100055646,LOC100055601,LOC100055561,LOC100055521,LOC100055478,LOC100055435,LOC100055397,LOC100055353,LOC100055309,LOC100055273,LOC100055224,LOC100055177,LOC100055129,LOC100055083,LOC100052898,LOC100147187,LOC100054954,LOC100054906,LOC100054858,LOC100054773,LOC100054722,LOC100054673,LOC100054627,LOC100054586,LOC100054541,LOC100054498,LOC100054453,LOC100146383,LOC100054355,LOC100054307,LOC100054258,LOC100054215,LOC100054169,LOC100054120,LOC100054065,LOC100054021,LOC100050556,LOC100053872,LOC100050476,LOC100053820,LOC100053768,LOC100629822,LOC100053672,LOC100053625,LOC100053568,LOC100053527,LOC100147669,LOC100053479,LOC100053427,LOC100053382,LOC100053328,LOC100053284,LOC100053236,LOC100053185,LOC100053134,LOC100053085,LOC100053045,LOC100052992,LOC100146502,LOC100052946,LOC100146388,LOC100052842,LOC100147383,LOC100052788,LOC100052732,LOC100050399,LOC100052683
12	12829176	14777981	1948806	3	4	LOC100058077,LOC100147282,LOC100058036,LOC100057989,LOC100057945,LOC100057894,LOC100057845,LOC100057804,LOC100057756,LOC100057709,LOC100057670,LOC100057589,LOC100057547,LOC100057513,LOC100057472,LOC100057433,LOC100057393,LOC100057352,LOC100057310,LOC100147286,LOC100146990,LOC100057269,LOC100057228,LOC100057191,LOC100057110,LOC100146600,LOC100057026,LOC100050714,LOC100056989,LOC100056947,LOC100056906,LOC100056869,LOC100146809,LOC100067978,LOC100067957,LOC100056826,LOC100056793,LOC100056755,LOC100147000,LOC100056708,LOC100056626,LOC100056578,LOC100056531,LOC100056488,LOC100629158,LOC100050633,LOC100056395,LOC100056356,LOC100056314,LOC100056273,LOC100056235,LOC100056187,LOC100056146,LOC100630788,LOC100629923,LOC100056026,LOC100055988,LOC100146992,LOC100055947,LOC100147569,LOC100146290,LOC100055908,LOC100055867,LOC100055780,LOC100630577,LOC100055646,LOC100055601,LOC100055561,LOC100055521,LOC100055478,LOC100055435,LOC100055397,LOC100055353,LOC100055309,LOC100055273,LOC100055224,LOC100055177,LOC100055129,LOC100055083,LOC100052898,LOC100147187,LOC100054954,LOC100054906,LOC100054858,LOC100054773,LOC100054722,LOC100054673,LOC100054627,LOC100054586,LOC100054541,LOC100054498,LOC100054453,LOC100146383,LOC100054355,LOC100054307,LOC100054258,LOC100054215,LOC100054169,LOC100054120,LOC100054065,LOC100054021,LOC100050556,LOC100053872,LOC100050476,LOC100053820,LOC100053768,LOC100629822,LOC100053672,LOC100053625,LOC100053568,LOC100053527,LOC100147669,LOC100053479,LOC100053427,LOC100053382,LOC100053328,LOC100053284,LOC100053236,LOC100053185,LOC100053134,LOC100053085,LOC100053045,LOC100052992,LOC100146502,LOC100052946,LOC100146388,LOC100052842,LOC100147383,LOC100052788,LOC100052732,LOC100050399,LOC100052683
12	12841994	13149957	307964	1	1	LOC100053672,LOC100053625,LOC100053568,LOC100053527,LOC100147669,LOC100053479,LOC100053427,LOC100053382,LOC100053328,LOC100053284,LOC100053236,LOC100053185,LOC100053134,LOC100053085,LOC100053045,LOC100052992,LOC100146502,LOC100052946,LOC100146388,LOC100052842,LOC100147383,LOC100052788,LOC100052732,LOC100050399,LOC100052683
12	12841994	13379725	537732	3	1	LOC100054258,LOC100054215,LOC100054169,LOC100054120,LOC100054065,LOC100054021,LOC100050556,LOC100053872,LOC100050476,LOC100053820,LOC100053768,LOC100629822,LOC100053672,LOC100053625,LOC100053568,LOC100053527,LOC100147669,LOC100053479,LOC100053427,LOC100053382,LOC100053328,LOC100053284,LOC100053236,LOC100053185,LOC100053134,LOC100053085,LOC100053045,LOC100052992,LOC100146502,LOC100052946,LOC100146388,LOC100052842,LOC100147383,LOC100052788,LOC100052732,LOC100050399,LOC100052683
12	12841994	14391372	1549379	1,3	1,2	LOC100057026,LOC100050714,LOC100056989,LOC100056947,LOC100056906,LOC100056869,LOC100146809,LOC100067978,LOC100067957,LOC100056826,LOC100056793,LOC100056755,LOC100147000,LOC100056708,LOC100056626,LOC100056578,LOC100056531,LOC100056488,LOC100629158,LOC100050633,LOC100056395,LOC100056356,LOC100056314,LOC100056273,LOC100056235,LOC100056187,LOC100056146,LOC100630788,LOC100629923,LOC100056026,LOC100055988,LOC100146992,LOC100055947,LOC100147569,LOC100146290,LOC100055908,LOC100055867,LOC100055780,LOC100630577,LOC100055646,LOC100055601,LOC100055561,LOC100055521,LOC100055478,LOC100055435,LOC100055397,LOC100055353,LOC100055309,LOC100055273,LOC100055224,LOC100055177,LOC100055129,LOC100055083,LOC100052898,LOC100147187,LOC100054954,LOC100054906,LOC100054858,LOC100054773,LOC100054722,LOC100054673,LOC100054627,LOC100054586,LOC100054541,LOC100054498,LOC100054453,LOC100146383,LOC100054355,LOC100054307,LOC100054258,LOC100054215,LOC100054169,LOC100054120,LOC100054065,LOC100054021,LOC100050556,LOC100053872,LOC100050476,LOC100053820,LOC100053768,LOC100629822,LOC100053672,LOC100053625,LOC100053568,LOC100053527,LOC100147669,LOC100053479,LOC100053427,LOC100053382,LOC100053328,LOC100053284,LOC100053236,LOC100053185,LOC100053134,LOC100053085,LOC100053045,LOC100052992,LOC100146502,LOC100052946,LOC100146388,LOC100052842,LOC100147383,LOC100052788,LOC100052732,LOC100050399,LOC100052683

Chrom	Start	End	Size	Cn	Samples (n)	Genes
12	13149957	13678953	528997	3	1	LOC100055353,LOC100055309,LOC100055273,LOC100055224,LOC100055177,LOC100055129,LOC100055083,LOC100052898,LOC100147187,LOC100054954,LOC100054906,LOC100054858,LOC100054773,LOC100054722,LOC100054673,LOC100054627,LOC100054586,LOC100054541,LOC100054498,LOC100054453,LOC100146383,LOC100054355,LOC100054307,LOC100054258,LOC100054215,LOC100054169,LOC100054120,LOC100054065,LOC100054021,LOC100050556,LOC100053872,LOC100050476,LOC100053820,LOC100053768,LOC100629822
12	13149957	13946509	796553	3	1	LOC100056146,LOC100630788,LOC100629923,LOC100056026,LOC100055988,LOC100146992,LOC100055947,LOC100147569,LOC100146290,LOC100055908,LOC100055867,LOC100055780,LOC100630577,LOC100055646,LOC100055601,LOC100055561,LOC100055521,LOC100055478,LOC100055435,LOC100055397,LOC100055353,LOC100055309,LOC100055273,LOC100055224,LOC100055177,LOC100055129,LOC100055083,LOC100052898,LOC100147187,LOC100054954,LOC100054906,LOC100054858,LOC100054773,LOC100054722,LOC100054673,LOC100054627,LOC100054586,LOC100054541,LOC100054498,LOC100054453,LOC100146383,LOC100054355,LOC100054307,LOC100054258,LOC100054215,LOC100054169,LOC100054120,LOC100054065,LOC100054021,LOC100050556,LOC100053872,LOC100050476,LOC100053820,LOC100053768,LOC100629822
12	13149957	14108352	958396	3	1	LOC100056531,LOC100056488,LOC100629158,LOC100050633,LOC100056395,LOC100056356,LOC100056314,LOC100056273,LOC100056235,LOC100056187,LOC100056146,LOC100630788,LOC100629923,LOC100056026,LOC100055988,LOC100146992,LOC100055947,LOC100147569,LOC100146290,LOC100055908,LOC100055867,LOC100055780,LOC100630577,LOC100055646,LOC100055601,LOC100055561,LOC100055521,LOC100055478,LOC100055435,LOC100055397,LOC100055353,LOC100055309,LOC100055273,LOC100055224,LOC100055177,LOC100055129,LOC100055083,LOC100052898,LOC100147187,LOC100054954,LOC100054906,LOC100054858,LOC100054773,LOC100054722,LOC100054673,LOC100054627,LOC100054586,LOC100054541,LOC100054498,LOC100054453,LOC100146383,LOC100054355,LOC100054307,LOC100054258,LOC100054215,LOC100054169,LOC100054120,LOC100054065,LOC100054021,LOC100050556,LOC100053872,LOC100050476,LOC100053820,LOC100053768,LOC100629822
12	13149957	14128309	978353	3	2	LOC100056626,LOC100056578,LOC100056531,LOC100056488,LOC100629158,LOC100050633,LOC100056395,LOC100056356,LOC100056314,LOC100056273,LOC100056235,LOC100056187,LOC100056146,LOC100630788,LOC100629923,LOC100056026,LOC100055988,LOC100146992,LOC100055947,LOC100147569,LOC100146290,LOC100055908,LOC100055867,LOC100055780,LOC100630577,LOC100055646,LOC100055601,LOC100055561,LOC100055521,LOC100055478,LOC100055435,LOC100055397,LOC100055353,LOC100055309,LOC100055273,LOC100055224,LOC100055177,LOC100055129,LOC100055083,LOC100052898,LOC100147187,LOC100054954,LOC100054906,LOC100054858,LOC100054773,LOC100054722,LOC100054673,LOC100054627,LOC100054586,LOC100054541,LOC100054498,LOC100054453,LOC100146383,LOC100054355,LOC100054307,LOC100054258,LOC100054215,LOC100054169,LOC100054120,LOC100054065,LOC100054021,LOC100050556,LOC100053872,LOC100050476,LOC100053820,LOC100053768,LOC100629822
12	13149957	14297056	1147100	3	1	LOC100056869,LOC100146809,LOC100067978,LOC100067957,LOC100056826,LOC100056793,LOC100056755,LOC100147000,LOC100056708,LOC100056626,LOC100056578,LOC100056531,LOC100056488,LOC100629158,LOC100050633,LOC100056395,LOC100056356,LOC100056314,LOC100056273,LOC100056235,LOC100056187,LOC100056146,LOC100630788,LOC100629923,LOC100056026,LOC100055988,LOC100146992,LOC100055947,LOC100147569,LOC100146290,LOC100055908,LOC100055867,LOC100055780,LOC100630577,LOC100055646,LOC100055601,LOC100055561,LOC100055521,LOC100055478,LOC100055435,LOC100055397,LOC100055353,LOC100055309,LOC100055273,LOC100055224,LOC100055177,LOC100055129,LOC100055083,LOC100052898,LOC100147187,LOC100054954,LOC100054906,LOC100054858,LOC100054773,LOC100054722,LOC100054673,LOC100054627,LOC100054586,LOC100054541,LOC100054498,LOC100054453,LOC100146383,LOC100054355,LOC100054307,LOC100054258,LOC100054215,LOC100054169,LOC100054120,LOC100054065,LOC100054021,LOC100050556,LOC100053872,LOC100050476,LOC100053820,LOC100053768,LOC100629822
12	13149957	14354314	1204358	3	5	LOC100056947,LOC100056906,LOC100056869,LOC100146809,LOC100067978,LOC100067957,LOC100056826,LOC100056793,LOC100056755,LOC100147000,LOC100056708,LOC100056626,LOC100056578,LOC100056531,LOC100056488,LOC100629158,LOC100050633,LOC100056395,LOC100056356,LOC100056314,LOC100056273,LOC100056235,LOC100056187,LOC100056146,LOC100630788,LOC100629923,LOC100056026,LOC100055988,LOC100146992,LOC100055947,LOC100147569,LOC100146290,LOC100055908,LOC100055867,LOC100055780,LOC100630577,LOC100055646,LOC100055601,LOC100055561,LOC100055521,LOC100055478,LOC100055435,LOC100055397,LOC100055353,LOC100055309,LOC100055273,LOC100055224,LOC100055177,LOC100055129,LOC100055083,LOC100052898,LOC100147187,LOC100054954,LOC100054906,LOC100054858,LOC100054773,LOC100054722,LOC100054673,LOC100054627,LOC100054586,LOC100054541,LOC100054498,LOC100054453,LOC100146383,LOC100054355,LOC100054307,LOC100054258,LOC100054215,LOC100054169,LOC100054120,LOC100054065,LOC100054021,LOC100050556,LOC100053872,LOC100050476,LOC100053820,LOC100053768,LOC100629822
12	13149957	14391372	1241416	1,3	1,3	LOC100057026,LOC100050714,LOC100056989,LOC100056947,LOC100056906,LOC100056869,LOC100146809,LOC100067978,LOC100067957,LOC100056826,LOC100056793,LOC100056755,LOC100147000,LOC100056708,LOC100056626,LOC100056578,LOC100056531,LOC100056488,LOC100629158,LOC100050633,LOC100056395,LOC100056356,LOC100056314,LOC100056273,LOC100056235,LOC100056187,LOC100056146,LOC100630788,LOC100629923,LOC100056026,LOC100055988,LOC100146992,LOC100055947,LOC100147569,LOC100146290,LOC100055908,LOC100055867,LOC100055780,LOC100630577,LOC100055646,LOC100055601,LOC100055561,LOC100055521,LOC100055478,LOC100055435,LOC100055397,LOC100055353,LOC100055309,LOC100055273,LOC100055224,LOC100055177,LOC100055129,LOC100055083,LOC100052898,LOC100147187,LOC100054954,LOC100054906,LOC100054858,LOC100054773,LOC100054722,LOC100054673,LOC100054627,LOC100054586,LOC100054541,LOC100054498,LOC100054453,LOC100146383,LOC100054355,LOC100054307,LOC100054258,LOC100054215,LOC100054169,LOC100054120,LOC100054065,LOC100054021,LOC100050556,LOC100053872,LOC100050476,LOC100053820,LOC100053768,LOC100629822

Chrom	Start	End	Size	Cn	Samples (n)	Genes
12	13149957	14587232	1437276	3	1	LOC100057472,LOC100057433,LOC100057393,LOC100057352,LOC100057310,LOC100147286,LOC100146990,LOC100057269,LOC100057228,LOC100057191,LOC100057110,LOC100146600,LOC100057026,LOC100050714,LOC100056989,LOC100056947,LOC100056906,LOC100056869,LOC100146809,LOC100067978,LOC100067957,LOC100056826,LOC100056793,LOC100056755,LOC100147000,LOC100056708,LOC100056626,LOC100056578,LOC100056531,LOC100056488,LOC100629158,LOC100050633,LOC100056395,LOC100056356,LOC100056314,LOC100056273,LOC100056235,LOC100056187,LOC100056146,LOC100630788,LOC100629923,LOC100056026,LOC100055988,LOC100146992,LOC100055947,LOC100147569,LOC100146290,LOC100055908,LOC100055867,LOC100055780,LOC100630577,LOC100055646,LOC100055601,LOC100055561,LOC100055521,LOC100055478,LOC100055435,LOC100055397,LOC100055353,LOC100055309,LOC100055273,LOC100055224,LOC100055177,LOC100055129,LOC100055083,LOC100052898,LOC100147187,LOC100054954,LOC100054906,LOC100054858,LOC100054773,LOC100054722,LOC100054673,LOC100054627,LOC100054586,LOC100054541,LOC100054498,LOC100054453,LOC100146383,LOC100054355,LOC100054307,LOC100054258,LOC100054215,LOC100054169,LOC100054120,LOC100054065,LOC100054021,LOC100050556,LOC100053872,LOC100050476,LOC100053820,LOC100053768,LOC100629822
12	13149957	14747048	1597092	3	1	LOC100057945,LOC100057894,LOC100057845,LOC100057804,LOC100057756,LOC100057709,LOC100057670,LOC100057589,LOC100057547,LOC100057513,LOC100057472,LOC100057433,LOC100057393,LOC100057352,LOC100057310,LOC100147286,LOC100146990,LOC100057269,LOC100057228,LOC100057191,LOC100057110,LOC100146600,LOC100057026,LOC100050714,LOC100056989,LOC100056947,LOC100056906,LOC100056869,LOC100146809,LOC100067978,LOC100067957,LOC100056826,LOC100056793,LOC100056755,LOC100147000,LOC100056708,LOC100056626,LOC100056578,LOC100056531,LOC100056488,LOC100629158,LOC100050633,LOC100056395,LOC100056356,LOC100056314,LOC100056273,LOC100056235,LOC100056187,LOC100056146,LOC100630788,LOC100629923,LOC100056026,LOC100055988,LOC100146992,LOC100055947,LOC100147569,LOC100146290,LOC100055908,LOC100055867,LOC100055780,LOC100630577,LOC100055646,LOC100055601,LOC100055561,LOC100055521,LOC100055478,LOC100055435,LOC100055397,LOC100055353,LOC100055309,LOC100055273,LOC100055224,LOC100055177,LOC100055129,LOC100055083,LOC100052898,LOC100147187,LOC100054954,LOC100054906,LOC100054858,LOC100054773,LOC100054722,LOC100054673,LOC100054627,LOC100054586,LOC100054541,LOC100054498,LOC100054453,LOC100146383,LOC100054355,LOC100054307,LOC100054258,LOC100054215,LOC100054169,LOC100054120,LOC100054065,LOC100054021,LOC100050556,LOC100053872,LOC100050476,LOC100053820,LOC100053768,LOC100629822
12	13149957	14777981	1628025	3	13	LOC100058077,LOC100147282,LOC100058036,LOC100057989,LOC100057945,LOC100057894,LOC100057845,LOC100057804,LOC100057756,LOC100057709,LOC100057670,LOC100057589,LOC100057547,LOC100057513,LOC100057472,LOC100057433,LOC100057393,LOC100057352,LOC100057310,LOC100147286,LOC100146990,LOC100057269,LOC100057228,LOC100057191,LOC100057110,LOC100146600,LOC100057026,LOC100050714,LOC100056989,LOC100056947,LOC100056906,LOC100056869,LOC100146809,LOC100067978,LOC100067957,LOC100056826,LOC100056793,LOC100056755,LOC100147000,LOC100056708,LOC100056626,LOC100056578,LOC100056531,LOC100056488,LOC100629158,LOC100050633,LOC100056395,LOC100056356,LOC100056314,LOC100056273,LOC100056235,LOC100056187,LOC100056146,LOC100630788,LOC100629923,LOC100056026,LOC100055988,LOC100146992,LOC100055947,LOC100147569,LOC100146290,LOC100055908,LOC100055867,LOC100055780,LOC100630577,LOC100055646,LOC100055601,LOC100055561,LOC100055521,LOC100055478,LOC100055435,LOC100055397,LOC100055353,LOC100055309,LOC100055273,LOC100055224,LOC100055177,LOC100055129,LOC100055083,LOC100052898,LOC100147187,LOC100054954,LOC100054906,LOC100054858,LOC100054773,LOC100054722,LOC100054673,LOC100054627,LOC100054586,LOC100054541,LOC100054498,LOC100054453,LOC100146383,LOC100054355,LOC100054307,LOC100054258,LOC100054215,LOC100054169,LOC100054120,LOC100054065,LOC100054021,LOC100050556,LOC100053872,LOC100050476,LOC100053820,LOC100053768,LOC100629822
12	13149957	15073209	1923253	3	1	LOC100058472,LOC100058429,LOC100058385,LOC100058346,LOC100146506,LOC100058253,LOC100147086,LOC100630034,LOC100058212,LOC100058168,LOC100058123,LOC100058077,LOC100147282,LOC100058036,LOC100057989,LOC100057945,LOC100057894,LOC100057845,LOC100057804,LOC100057756,LOC100057709,LOC100057670,LOC100057589,LOC100057547,LOC100057513,LOC100057472,LOC100057433,LOC100057393,LOC100057352,LOC100057310,LOC100147286,LOC100146990,LOC100057269,LOC100057228,LOC100057191,LOC100057110,LOC100146600,LOC100057026,LOC100050714,LOC100056989,LOC100056947,LOC100056906,LOC100056869,LOC100146809,LOC100067978,LOC100067957,LOC100056826,LOC100056793,LOC100056755,LOC100147000,LOC100056708,LOC100056626,LOC100056578,LOC100056531,LOC100056488,LOC100629158,LOC100050633,LOC100056395,LOC100056356,LOC100056314,LOC100056273,LOC100056235,LOC100056187,LOC100056146,LOC100630788,LOC100629923,LOC100056026,LOC100055988,LOC100146992,LOC100055947,LOC100147569,LOC100146290,LOC100055908,LOC100055867,LOC100055780,LOC100630577,LOC100055646,LOC100055601,LOC100055561,LOC100055521,LOC100055478,LOC100055435,LOC100055397,LOC100055353,LOC100055309,LOC100055273,LOC100055224,LOC100055177,LOC100055129,LOC100055083,LOC100052898,LOC100147187,LOC100054954,LOC100054906,LOC100054858,LOC100054773,LOC100054722,LOC100054673,LOC100054627,LOC100054586,LOC100054541,LOC100054498,LOC100054453,LOC100146383,LOC100054355,LOC100054307,LOC100054258,LOC100054215,LOC100054169,LOC100054120,LOC100054065,LOC100054021,LOC100050556,LOC100053872,LOC100050476,LOC100053820,LOC100053768,LOC100629822
12	13364132	13401991	37860	0	1	LOC100146383,LOC100054355,LOC100054307,LOC100054258
12	13364132	13573356	209225	1,3	2,2	LOC100147187,LOC100054954,LOC100054906,LOC100054858,LOC100054773,LOC100054722,LOC100054673,LOC100054627,LOC100054586,LOC100054541,LOC100054498,LOC100054453,LOC100146383,LOC100054355,LOC100054307,LOC100054258
12	13364132	13678953	314822	3	1	LOC100055353,LOC100055309,LOC100055273,LOC100055224,LOC100055177,LOC100055129,LOC100055083,LOC100052898,LOC100147187,LOC100054954,LOC100054906,LOC100054858,LOC100054773,LOC100054722,LOC100054673,LOC100054627,LOC100054586,LOC100054541,LOC100054498,LOC100054453,LOC100146383,LOC100054355,LOC100054307,LOC100054258

Chrom	Start	End	Size	Cn	Samples (n)	Genes
12	13364132	14128309	764178	3	1	LOC100056626,LOC100056578,LOC100056531,LOC100056488,LOC100629158,LOC100050633,LOC100056395,LOC100056356,LOC100056314,LOC100056273,LOC100056235,LOC100056187,LOC100056146,LOC100630788,LOC100629923,LOC100056026,LOC100055988,LOC100146992,LOC100055947,LOC100147569,LOC100146290,LOC100055908,LOC100055867,LOC100055780,LOC100630577,LOC100055646,LOC100055601,LOC100055561,LOC100055521,LOC100055478,LOC100055435,LOC100055397,LOC100055353,LOC100055309,LOC100055273,LOC100055224,LOC100055177,LOC100055129,LOC100055083,LOC100052898,LOC100147187,LOC100054954,LOC100054906,LOC100054858,LOC100054773,LOC100054722,LOC100054673,LOC100054627,LOC100054586,LOC100054541,LOC100054498,LOC100054453,LOC100146383,LOC100054355,LOC100054307,LOC100054258
12	13364132	14354314	990183	3	2	LOC100056947,LOC100056906,LOC100056869,LOC100146809,LOC100067978,LOC100067957,LOC100056826,LOC100056793,LOC100056755,LOC100147000,LOC100056708,LOC100056626,LOC100056578,LOC100056531,LOC100056488,LOC100629158,LOC100050633,LOC100056395,LOC100056356,LOC100056314,LOC100056273,LOC100056235,LOC100056187,LOC100056146,LOC100630788,LOC100629923,LOC100056026,LOC100055988,LOC100146992,LOC100055947,LOC100147569,LOC100146290,LOC100055908,LOC100055867,LOC100055780,LOC100630577,LOC100055646,LOC100055601,LOC100055561,LOC100055521,LOC100055478,LOC100055435,LOC100055397,LOC100055353,LOC100055309,LOC100055273,LOC100055224,LOC100055177,LOC100055129,LOC100055083,LOC100052898,LOC100147187,LOC100054954,LOC100054906,LOC100054858,LOC100054773,LOC100054722,LOC100054673,LOC100054627,LOC100054586,LOC100054541,LOC100054498,LOC100054453,LOC100146383,LOC100054355,LOC100054307,LOC100054258
12	13364132	14391372	1027241	3	6	LOC100057026,LOC100050714,LOC100056989,LOC100056947,LOC100056906,LOC100056869,LOC100146809,LOC100067978,LOC100067957,LOC100056826,LOC100056793,LOC100056755,LOC100147000,LOC100056708,LOC100056626,LOC100056578,LOC100056531,LOC100056488,LOC100629158,LOC100050633,LOC100056395,LOC100056356,LOC100056314,LOC100056273,LOC100056235,LOC100056187,LOC100056146,LOC100630788,LOC100629923,LOC100056026,LOC100055988,LOC100146992,LOC100055947,LOC100147569,LOC100146290,LOC100055908,LOC100055867,LOC100055780,LOC100630577,LOC100055646,LOC100055601,LOC100055561,LOC100055521,LOC100055478,LOC100055435,LOC100055397,LOC100055353,LOC100055309,LOC100055273,LOC100055224,LOC100055177,LOC100055129,LOC100055083,LOC100052898,LOC100147187,LOC100054954,LOC100054906,LOC100054858,LOC100054773,LOC100054722,LOC100054673,LOC100054627,LOC100054586,LOC100054541,LOC100054498,LOC100054453,LOC100146383,LOC100054355,LOC100054307,LOC100054258
12	13364132	14777981	1413850	3	2	LOC100058077,LOC100147282,LOC100058036,LOC100057989,LOC100057945,LOC100057894,LOC100057845,LOC100057804,LOC100057756,LOC100057709,LOC100057670,LOC100057589,LOC100057547,LOC100057513,LOC100057472,LOC100057433,LOC100057393,LOC100057352,LOC100057310,LOC100147286,LOC100146990,LOC100057269,LOC100057228,LOC100057191,LOC100057110,LOC100146600,LOC100057026,LOC100050714,LOC100056989,LOC100056947,LOC100056906,LOC100056869,LOC100146809,LOC100067978,LOC100067957,LOC100056826,LOC100056793,LOC100056755,LOC100147000,LOC100056708,LOC100056626,LOC100056578,LOC100056531,LOC100056488,LOC100629158,LOC100050633,LOC100056395,LOC100056356,LOC100056314,LOC100056273,LOC100056235,LOC100056187,LOC100056146,LOC100630788,LOC100629923,LOC100056026,LOC100055988,LOC100146992,LOC100055947,LOC100147569,LOC100146290,LOC100055908,LOC100055867,LOC100055780,LOC100630577,LOC100055646,LOC100055601,LOC100055561,LOC100055521,LOC100055478,LOC100055435,LOC100055397,LOC100055353,LOC100055309,LOC100055273,LOC100055224,LOC100055177,LOC100055129,LOC100055083,LOC100052898,LOC100147187,LOC100054954,LOC100054906,LOC100054858,LOC100054773,LOC100054722,LOC100054673,LOC100054627,LOC100054586,LOC100054541,LOC100054498,LOC100054453,LOC100146383,LOC100054355,LOC100054307,LOC100054258
12	13364132	15073209	1709078	4	1	LOC100058472,LOC100058429,LOC100058385,LOC100058346,LOC100146506,LOC100058253,LOC100147086,LOC100630034,LOC100058212,LOC100058168,LOC100058123,LOC100058077,LOC100147282,LOC100058036,LOC100057989,LOC100057945,LOC100057894,LOC100057845,LOC100057804,LOC100057756,LOC100057709,LOC100057670,LOC100057589,LOC100057547,LOC100057513,LOC100057472,LOC100057433,LOC100057393,LOC100057352,LOC100057310,LOC100147286,LOC100146990,LOC100057269,LOC100057228,LOC100057191,LOC100057110,LOC100146600,LOC100057026,LOC100050714,LOC100056989,LOC100056947,LOC100056906,LOC100056869,LOC100146809,LOC100067978,LOC100067957,LOC100056826,LOC100056793,LOC100056755,LOC100147000,LOC100056708,LOC100056626,LOC100056578,LOC100056531,LOC100056488,LOC100629158,LOC100050633,LOC100056395,LOC100056356,LOC100056314,LOC100056273,LOC100056235,LOC100056187,LOC100056146,LOC100630788,LOC100629923,LOC100056026,LOC100055988,LOC100146992,LOC100055947,LOC100147569,LOC100146290,LOC100055908,LOC100055867,LOC100055780,LOC100630577,LOC100055646,LOC100055601,LOC100055561,LOC100055521,LOC100055478,LOC100055435,LOC100055397,LOC100055353,LOC100055309,LOC100055273,LOC100055224,LOC100055177,LOC100055129,LOC100055083,LOC100052898,LOC100147187,LOC100054954,LOC100054906,LOC100054858,LOC100054773,LOC100054722,LOC100054673,LOC100054627,LOC100054586,LOC100054541,LOC100054498,LOC100054453,LOC100146383,LOC100054355,LOC100054307,LOC100054258
12	13379725	13488187	108463	1	1	LOC100054673,LOC100054627,LOC100054586,LOC100054541,LOC100054498,LOC100054453,LOC100146383,LOC100054355,LOC100054307
12	13422256	14391372	969117	1	3	LOC100057026,LOC100050714,LOC100056989,LOC100056947,LOC100056906,LOC100056869,LOC100146809,LOC100067978,LOC100067957,LOC100056826,LOC100056793,LOC100056755,LOC100147000,LOC100056708,LOC100056626,LOC100056578,LOC100056531,LOC100056488,LOC100629158,LOC100050633,LOC100056395,LOC100056356,LOC100056314,LOC100056273,LOC100056235,LOC100056187,LOC100056146,LOC100630788,LOC100629923,LOC100056026,LOC100055988,LOC100146992,LOC100055947,LOC100147569,LOC100146290,LOC100055908,LOC100055867,LOC100055780,LOC100630577,LOC100055646,LOC100055601,LOC100055561,LOC100055521,LOC100055478,LOC100055435,LOC100055397,LOC100055353,LOC100055309,LOC100055273,LOC100055224,LOC100055177,LOC100055129,LOC100055083,LOC100052898,LOC100147187,LOC100054954,LOC100054906,LOC100054858,LOC100054773,LOC100054722,LOC100054673,LOC100054627,LOC100054586,LOC100054541,LOC100054498

Chrom	Start	End	Size	Cn	Samples (n)	Genes
12	13422256	14587232	1164977	1	1	LOC100057472,LOC100057433,LOC100057393,LOC100057352,LOC100057310,LOC100147286,LOC100146990,LOC100057269,LOC100057228,LOC100057191,LOC100057110,LOC100146600,LOC100057026,LOC100050714,LOC100056989,LOC100056947,LOC100056906,LOC100056869,LOC100146809,LOC100067978,LOC100067957,LOC100056826,LOC100056793,LOC100056755,LOC100147000,LOC100056708,LOC100056626,LOC100056578,LOC100056531,LOC100056488,LOC100629158,LOC100050633,LOC100056395,LOC100056356,LOC100056314,LOC100056273,LOC100056235,LOC100056187,LOC100056146,LOC100630788,LOC100629923,LOC100056026,LOC100055988,LOC100146992,LOC100055947,LOC100147569,LOC100146290,LOC100055908,LOC100055867,LOC100055780,LOC100630577,LOC100055646,LOC100055601,LOC100055561,LOC100055521,LOC100055478,LOC100055435,LOC100055397,LOC100055353,LOC100055309,LOC100055273,LOC100055224,LOC100055177,LOC100055129,LOC100055083,LOC100052898,LOC100147187,LOC100054954,LOC100054906,LOC100054858,LOC100054773,LOC100054722,LOC100054673,LOC100054627,LOC100054586,LOC100054541,LOC100054498
12	13422256	14777981	1355726	1	1	LOC100058077,LOC100147282,LOC100058036,LOC100057989,LOC100057945,LOC100057894,LOC100057845,LOC100057804,LOC100057756,LOC100057709,LOC100057670,LOC100057589,LOC100057547,LOC100057513,LOC100057472,LOC100057433,LOC100057393,LOC100057352,LOC100057310,LOC100147286,LOC100146990,LOC100057269,LOC100057228,LOC100057191,LOC100057110,LOC100146600,LOC100057026,LOC100050714,LOC100056989,LOC100056947,LOC100056906,LOC100056869,LOC100146809,LOC100067978,LOC100067957,LOC100056826,LOC100056793,LOC100056755,LOC100147000,LOC100056708,LOC100056626,LOC100056578,LOC100056531,LOC100056488,LOC100629158,LOC100050633,LOC100056395,LOC100056356,LOC100056314,LOC100056273,LOC100056235,LOC100056187,LOC100056146,LOC100630788,LOC100629923,LOC100056026,LOC100055988,LOC100146992,LOC100055947,LOC100147569,LOC100146290,LOC100055908,LOC100055867,LOC100055780,LOC100630577,LOC100055646,LOC100055601,LOC100055561,LOC100055521,LOC100055478,LOC100055435,LOC100055397,LOC100055353,LOC100055309,LOC100055273,LOC100055224,LOC100055177,LOC100055129,LOC100055083,LOC100052898,LOC100147187,LOC100054954,LOC100054906,LOC100054858,LOC100054773,LOC100054722,LOC100054673,LOC100054627,LOC100054586,LOC100054541,LOC100054498
12	13439146	14354314	915169	4	1	LOC100056947,LOC100056906,LOC100056869,LOC100146809,LOC100067978,LOC100067957,LOC100056826,LOC100056793,LOC100056755,LOC100147000,LOC100056708,LOC100056626,LOC100056578,LOC100056531,LOC100056488,LOC100629158,LOC100050633,LOC100056395,LOC100056356,LOC100056314,LOC100056273,LOC100056235,LOC100056187,LOC100056146,LOC100630788,LOC100629923,LOC100056026,LOC100055988,LOC100146992,LOC100055947,LOC100147569,LOC100146290,LOC100055908,LOC100055867,LOC100055780,LOC100630577,LOC100055646,LOC100055601,LOC100055561,LOC100055521,LOC100055478,LOC100055435,LOC100055397,LOC100055353,LOC100055309,LOC100055273,LOC100055224,LOC100055177,LOC100055129,LOC100055083,LOC100052898,LOC100147187,LOC100054954,LOC100054906,LOC100054858,LOC100054773,LOC100054722,LOC100054673,LOC100054627,LOC100054586,LOC100054541
12	13439146	14391372	952227	1	1	LOC100057026,LOC100050714,LOC100056989,LOC100056947,LOC100056906,LOC100056869,LOC100146809,LOC100067978,LOC100067957,LOC100056826,LOC100056793,LOC100056755,LOC100147000,LOC100056708,LOC100056626,LOC100056578,LOC100056531,LOC100056488,LOC100629158,LOC100050633,LOC100056395,LOC100056356,LOC100056314,LOC100056273,LOC100056235,LOC100056187,LOC100056146,LOC100630788,LOC100629923,LOC100056026,LOC100055988,LOC100146992,LOC100055947,LOC100147569,LOC100146290,LOC100055908,LOC100055867,LOC100055780,LOC100630577,LOC100055646,LOC100055601,LOC100055561,LOC100055521,LOC100055478,LOC100055435,LOC100055397,LOC100055353,LOC100055309,LOC100055273,LOC100055224,LOC100055177,LOC100055129,LOC100055083,LOC100052898,LOC100147187,LOC100054954,LOC100054906,LOC100054858,LOC100054773,LOC100054722,LOC100054673,LOC100054627,LOC100054586,LOC100054541
12	13439146	14587232	1148087	1	1	LOC100057472,LOC100057433,LOC100057393,LOC100057352,LOC100057310,LOC100147286,LOC100146990,LOC100057269,LOC100057228,LOC100057191,LOC100057110,LOC100146600,LOC100057026,LOC100050714,LOC100056989,LOC100056947,LOC100056906,LOC100056869,LOC100146809,LOC100067978,LOC100067957,LOC100056826,LOC100056793,LOC100056755,LOC100147000,LOC100056708,LOC100056626,LOC100056578,LOC100056531,LOC100056488,LOC100629158,LOC100050633,LOC100056395,LOC100056356,LOC100056314,LOC100056273,LOC100056235,LOC100056187,LOC100056146,LOC100630788,LOC100629923,LOC100056026,LOC100055988,LOC100146992,LOC100055947,LOC100147569,LOC100146290,LOC100055908,LOC100055867,LOC100055780,LOC100630577,LOC100055646,LOC100055601,LOC100055561,LOC100055521,LOC100055478,LOC100055435,LOC100055397,LOC100055353,LOC100055309,LOC100055273,LOC100055224,LOC100055177,LOC100055129,LOC100055083,LOC100052898,LOC100147187,LOC100054954,LOC100054906,LOC100054858,LOC100054773,LOC100054722,LOC100054673,LOC100054627,LOC100054586,LOC100054541
12	13488187	14128309	640123	3	1	LOC100056626,LOC100056578,LOC100056531,LOC100056488,LOC100629158,LOC100050633,LOC100056395,LOC100056356,LOC100056314,LOC100056273,LOC100056235,LOC100056187,LOC100056146,LOC100630788,LOC100629923,LOC100056026,LOC100055988,LOC100146992,LOC100055947,LOC100147569,LOC100146290,LOC100055908,LOC100055867,LOC100055780,LOC100630577,LOC100055646,LOC100055601,LOC100055561,LOC100055521,LOC100055478,LOC100055435,LOC100055397,LOC100055353,LOC100055309,LOC100055273,LOC100055224,LOC100055177,LOC100055129,LOC100055083,LOC100052898,LOC100147187,LOC100054954,LOC100054906,LOC100054858,LOC100054773,LOC100054722
12	13488187	14354314	866128	3	2	LOC100056947,LOC100056906,LOC100056869,LOC100146809,LOC100067978,LOC100067957,LOC100056826,LOC100056793,LOC100056755,LOC100147000,LOC100056708,LOC100056626,LOC100056578,LOC100056531,LOC100056488,LOC100629158,LOC100050633,LOC100056395,LOC100056356,LOC100056314,LOC100056273,LOC100056235,LOC100056187,LOC100056146,LOC100630788,LOC100629923,LOC100056026,LOC100055988,LOC100146992,LOC100055947,LOC100147569,LOC100146290,LOC100055908,LOC100055867,LOC100055780,LOC100630577,LOC100055646,LOC100055601,LOC100055561,LOC100055521,LOC100055478,LOC100055435,LOC100055397,LOC100055353,LOC100055309,LOC100055273,LOC100055224,LOC100055177,LOC100055129,LOC100055083,LOC100052898,LOC100147187,LOC100054954,LOC100054906,LOC100054858,LOC100054773,LOC100054722

Chrom	Start	End	Size	Cn	Samples (n)	Genes
12	13488187	14391372	903186	3	4	LOC100057026,LOC100050714,LOC100056989,LOC100056947,LOC100056906,LOC100056869,LOC100146809,LOC100067978,LOC100067957,LOC100056826,LOC100056793,LOC100056755,LOC100147000,LOC100056708,LOC100056626,LOC100056578,LOC100056531,LOC100056488,LOC100629158,LOC100050633,LOC100056395,LOC100056356,LOC100056314,LOC100056273,LOC100056235,LOC100056187,LOC100056146,LOC100630788,LOC100629923,LOC100056026,LOC100055988,LOC100146992,LOC100055947,LOC100147569,LOC100146290,LOC100055908,LOC100055867,LOC100055780,LOC100630577,LOC100055646,LOC100055601,LOC100055561,LOC100055521,LOC100055478,LOC100055435,LOC100055397,LOC100055353,LOC100055309,LOC100055273,LOC100055224,LOC100055177,LOC100055129,LOC100055083,LOC100052898,LOC100147187,LOC100054954,LOC100054906,LOC100054858,LOC100054773,LOC100054722
12	13488187	14777981	1289795	3	5	LOC100058077,LOC100147282,LOC100058036,LOC100057989,LOC100057945,LOC100057894,LOC100057845,LOC100057804,LOC100057756,LOC100057709,LOC100057670,LOC100057589,LOC100057547,LOC100057513,LOC100057472,LOC100057433,LOC100057393,LOC100057352,LOC100057310,LOC100147286,LOC100146990,LOC100057269,LOC100057228,LOC100057191,LOC100057110,LOC100146600,LOC100057026,LOC100050714,LOC100056989,LOC100056947,LOC100056906,LOC100056869,LOC100146809,LOC100067978,LOC100067957,LOC100056826,LOC100056793,LOC100056755,LOC100147000,LOC100056708,LOC100056626,LOC100056578,LOC100056531,LOC100056488,LOC100629158,LOC100050633,LOC100056395,LOC100056356,LOC100056314,LOC100056273,LOC100056235,LOC100056187,LOC100056146,LOC100630788,LOC100629923,LOC100056026,LOC100055988,LOC100146992,LOC100055947,LOC100147569,LOC100146290,LOC100055908,LOC100055867,LOC100055780,LOC100630577,LOC100055646,LOC100055601,LOC100055561,LOC100055521,LOC100055478,LOC100055435,LOC100055397,LOC100055353,LOC100055309,LOC100055273,LOC100055224,LOC100055177,LOC100055129,LOC100055083,LOC100052898,LOC100147187,LOC100054954,LOC100054906,LOC100054858,LOC100054773,LOC100054722
12	13573356	14128309	554954	3	1	LOC100056626,LOC100056578,LOC100056531,LOC100056488,LOC100629158,LOC100050633,LOC100056395,LOC100056356,LOC100056314,LOC100056273,LOC100056235,LOC100056187,LOC100056146,LOC100630788,LOC100629923,LOC100056026,LOC100055988,LOC100146992,LOC100055947,LOC100147569,LOC100146290,LOC100055908,LOC100055867,LOC100055780,LOC100630577,LOC100055646,LOC100055601,LOC100055561,LOC100055521,LOC100055478,LOC100055435,LOC100055397,LOC100055353,LOC100055309,LOC100055273,LOC100055224,LOC100055177,LOC100055129,LOC100055083,LOC100052898
12	13573356	14354314	780959	3,4	1,1	LOC100056947,LOC100056906,LOC100056869,LOC100146809,LOC100067978,LOC100067957,LOC100056826,LOC100056793,LOC100056755,LOC100147000,LOC100056708,LOC100056626,LOC100056578,LOC100056531,LOC100056488,LOC100629158,LOC100050633,LOC100056395,LOC100056356,LOC100056314,LOC100056273,LOC100056235,LOC100056187,LOC100056146,LOC100630788,LOC100629923,LOC100056026,LOC100055988,LOC100146992,LOC100055947,LOC100147569,LOC100146290,LOC100055908,LOC100055867,LOC100055780,LOC100630577,LOC100055646,LOC100055601,LOC100055561,LOC100055521,LOC100055478,LOC100055435,LOC100055397,LOC100055353,LOC100055309,LOC100055273,LOC100055224,LOC100055177,LOC100055129,LOC100055083,LOC100052898
12	13573356	14391372	818017	3,4	1,2	LOC100057026,LOC100050714,LOC100056989,LOC100056947,LOC100056906,LOC100056869,LOC100146809,LOC100067978,LOC100067957,LOC100056826,LOC100056793,LOC100056755,LOC100147000,LOC100056708,LOC100056626,LOC100056578,LOC100056531,LOC100056488,LOC100629158,LOC100050633,LOC100056395,LOC100056356,LOC100056314,LOC100056273,LOC100056235,LOC100056187,LOC100056146,LOC100630788,LOC100629923,LOC100056026,LOC100055988,LOC100146992,LOC100055947,LOC100147569,LOC100146290,LOC100055908,LOC100055867,LOC100055780,LOC100630577,LOC100055646,LOC100055601,LOC100055561,LOC100055521,LOC100055478,LOC100055435,LOC100055397,LOC100055353,LOC100055309,LOC100055273,LOC100055224,LOC100055177,LOC100055129,LOC100055083,LOC100052898
12	13573356	14777981	1204626	3	1	LOC100058077,LOC100147282,LOC100058036,LOC100057989,LOC100057945,LOC100057894,LOC100057845,LOC100057804,LOC100057756,LOC100057709,LOC100057670,LOC100057589,LOC100057547,LOC100057513,LOC100057472,LOC100057433,LOC100057393,LOC100057352,LOC100057310,LOC100147286,LOC100146990,LOC100057269,LOC100057228,LOC100057191,LOC100057110,LOC100146600,LOC100057026,LOC100050714,LOC100056989,LOC100056947,LOC100056906,LOC100056869,LOC100146809,LOC100067978,LOC100067957,LOC100056826,LOC100056793,LOC100056755,LOC100147000,LOC100056708,LOC100056626,LOC100056578,LOC100056531,LOC100056488,LOC100629158,LOC100050633,LOC100056395,LOC100056356,LOC100056314,LOC100056273,LOC100056235,LOC100056187,LOC100056146,LOC100630788,LOC100629923,LOC100056026,LOC100055988,LOC100146992,LOC100055947,LOC100147569,LOC100146290,LOC100055908,LOC100055867,LOC100055780,LOC100630577,LOC100055646,LOC100055601,LOC100055561,LOC100055521,LOC100055478,LOC100055435,LOC100055397,LOC100055353,LOC100055309,LOC100055273,LOC100055224,LOC100055177,LOC100055129,LOC100055083,LOC100052898
12	13573356	15073209	1499854	3,4	2,1	LOC100058472,LOC100058429,LOC100058385,LOC100058346,LOC100146506,LOC100058253,LOC100147086,LOC100630034,LOC100058212,LOC100058168,LOC100058123,LOC100058077,LOC100147282,LOC100058036,LOC100057989,LOC100057945,LOC100057894,LOC100057845,LOC100057804,LOC100057756,LOC100057709,LOC100057670,LOC100057589,LOC100057547,LOC100057513,LOC100057472,LOC100057433,LOC100057393,LOC100057352,LOC100057310,LOC100147286,LOC100146990,LOC100057269,LOC100057228,LOC100057191,LOC100057110,LOC100146600,LOC100057026,LOC100050714,LOC100056989,LOC100056947,LOC100056906,LOC100056869,LOC100146809,LOC100067978,LOC100067957,LOC100056826,LOC100056793,LOC100056755,LOC100147000,LOC100056708,LOC100056626,LOC100056578,LOC100056531,LOC100056488,LOC100629158,LOC100050633,LOC100056395,LOC100056356,LOC100056314,LOC100056273,LOC100056235,LOC100056187,LOC100056146,LOC100630788,LOC100629923,LOC100056026,LOC100055988,LOC100146992,LOC100055947,LOC100147569,LOC100146290,LOC100055908,LOC100055867,LOC100055780,LOC100630577,LOC100055646,LOC100055601,LOC100055561,LOC100055521,LOC100055478,LOC100055435,LOC100055397,LOC100055353,LOC100055309,LOC100055273,LOC100055224,LOC100055177,LOC100055129,LOC100055083,LOC100052898
12	13945011	14108352	163342	4	1	LOC100056531,LOC100056488,LOC100629158,LOC100050633,LOC100056395,LOC100056356,LOC100056314,LOC100056273,LOC100056235,LOC100056187,LOC100056146
12	13945011	14128309	183299	3,4	3,4	LOC100056626,LOC100056578,LOC100056531,LOC100056488,LOC100629158,LOC100050633,LOC100056395,LOC100056356,LOC100056314,LOC100056273,LOC100056235,LOC100056187,LOC100056146
12	13945011	14354314	409304	3	2	LOC100056947,LOC100056906,LOC100056869,LOC100146809,LOC100067978,LOC100067957,LOC100056826,LOC100056793,LOC100056755,LOC100147000,LOC100056708,LOC100056626,LOC100056578,LOC100056531,LOC100056488,LOC100629158,LOC100050633,LOC100056395,LOC100056356,LOC100056314,LOC100056273,LOC100056235,LOC100056187,LOC100056146

Chrom	Start	End	Size	Cn	Samples (n)	Genes
12	13945011	14391372	446362	3,4	6,2	LOC100057026,LOC100050714,LOC100056989,LOC100056947,LOC100056906,LOC100056869,LOC100146809,LOC100067978,LOC100067957,LOC100056826,LOC100056793,LOC100056755,LOC100147000,LOC100056708,LOC100056626,LOC100056578,LOC100056531,LOC100056488,LOC100629158,LOC100050633,LOC100056395,LOC100056356,LOC100056314,LOC100056273,LOC100056235,LOC100056187,LOC100056146
12	13945011	14777981	832971	3,4	7,3	LOC100058077,LOC100147282,LOC100058036,LOC100057989,LOC100057945,LOC100057894,LOC100057845,LOC100057804,LOC100057756,LOC100057709,LOC100057670,LOC100057589,LOC100057547,LOC100057513,LOC100057472,LOC100057433,LOC100057393,LOC100057352,LOC100057310,LOC100147286,LOC100146990,LOC100057269,LOC100057228,LOC100057191,LOC100057110,LOC100146600,LOC100057026,LOC100050714,LOC100056989,LOC100056947,LOC100056906,LOC100056869,LOC100146809,LOC100067978,LOC100067957,LOC100056826,LOC100056793,LOC100056755,LOC100147000,LOC100056708,LOC100056626,LOC100056578,LOC100056531,LOC100056488,LOC100629158,LOC100050633,LOC100056395,LOC100056356,LOC100056314,LOC100056273,LOC100056235,LOC100056187,LOC100056146
12	13945011	17011411	3066401	3	1	LRRC55,LOC100066827,LOC100066804,LOC100066784,LOC100066742,LOC100147445,LOC100062766,LOC100066715,LOC100066690,LOC100066643,LOC100066553,LOC100066503,LOC100066447,LOC100066417,LOC100066361,LOC100066225,LOC100066190,LOC100066164,LOC100630861,LOC100066108,LOC100066068,LOC100147059,LOC100066040,LOC100062734,LOC100066011,LOC100065987,LOC100146657,LOC100065959,LOC100065934,LOC100147543,LOC100065911,LOC100065887,LOC100065858,LOC100065831,LOC100065803,LOC100065755,LOC100065735,LOC100065693,LOC100065674,LOC100065655,LOC100065634,LOC100146350,LOC100065610,LOC100065591,LOC100065549,LOC100146953,LOC100065500,LOC100065474,LOC100062694,LOC100065451,LOC100065423,LOC100065402,LOC100065380,LOC100065349,LOC100065319,LOC100065288,LOC100146469,LOC100065263,LOC100147060,LOC100065230,LOC100065201,LOC100065168,LOC100065136,LOC100065111,LOC100065079,LOC100065048,LOC100065019,LOC100630301,LOC100064956,LOC100064924,LOC100062657,LOC100064900,LOC100064874,LOC100064848,LOC100064820,LOC100064792,LOC100064769,LOC100064716,LOC100064690,LOC100062590,LOC100062626,LOC100064662,LOC100630007,LOC100064597,LOC100064562,LOC100058472,LOC100058429,LOC100058385,LOC100058346,LOC100146506,LOC100058253,LOC100147086,LOC100630034,LOC100058212,LOC100058168,LOC100058123,LOC100058077,LOC100147282,LOC100058036,LOC100057989,LOC100057945,LOC100057894,LOC100057845,LOC100057804,LOC100057756,LOC100057709,LOC100057670,LOC100057589,LOC100057547,LOC100057513,LOC100057472,LOC100057433,LOC100057393,LOC100057352,LOC100057310,LOC100147286,LOC100146990,LOC100057269,LOC100057228,LOC100057191,LOC100057110,LOC100146600,LOC100057026,LOC100050714,LOC100056989,LOC100056947,LOC100056906,LOC100056869,LOC100146809,LOC100067978,LOC100067957,LOC100056826,LOC100056793,LOC100056755,LOC100147000,LOC100056708,LOC100056626,LOC100056578,LOC100056531,LOC100056488,LOC100629158,LOC100050633,LOC100056395,LOC100056356,LOC100056314,LOC100056273,LOC100056235,LOC100056187,LOC100056146
12	13945011	17084040	3139030	3	1	SSRP1,TNKS1BP1,LOC100066889,LRRC55,LOC100066827,LOC100066804,LOC100066784,LOC100066742,LOC100147445,LOC100062766,LOC100066715,LOC100066690,LOC100066643,LOC100066553,LOC100066503,LOC100066447,LOC100066417,LOC100066361,LOC100066225,LOC100066190,LOC100066164,LOC100630861,LOC100066108,LOC100066068,LOC100147059,LOC100066040,LOC100062734,LOC100066011,LOC100065987,LOC100146657,LOC100065959,LOC100065934,LOC100147543,LOC100065911,LOC100065887,LOC100065858,LOC100065831,LOC100065803,LOC100065755,LOC100065735,LOC100065693,LOC100065674,LOC100065655,LOC100065634,LOC100146350,LOC100065610,LOC100065591,LOC100065549,LOC100146953,LOC100065500,LOC100065474,LOC100062694,LOC100065451,LOC100065423,LOC100065402,LOC100065380,LOC100065349,LOC100065319,LOC100065288,LOC100146469,LOC100065263,LOC100147060,LOC100065230,LOC100065201,LOC100065168,LOC100065136,LOC100065111,LOC100065079,LOC100065048,LOC100065019,LOC100630301,LOC100064956,LOC100064924,LOC100062657,LOC100064900,LOC100064874,LOC100064848,LOC100064820,LOC100064792,LOC100064769,LOC100064716,LOC100064690,LOC100062590,LOC100062626,LOC100064662,LOC100630007,LOC100064597,LOC100064562,LOC100058472,LOC100058429,LOC100058385,LOC100058346,LOC100146506,LOC100058253,LOC100147086,LOC100630034,LOC100058212,LOC100058168,LOC100058123,LOC100058077,LOC100147282,LOC100058036,LOC100057989,LOC100057945,LOC100057894,LOC100057845,LOC100057804,LOC100057756,LOC100057709,LOC100057670,LOC100057589,LOC100057547,LOC100057513,LOC100057472,LOC100057433,LOC100057393,LOC100057352,LOC100057310,LOC100147286,LOC100146990,LOC100057269,LOC100057228,LOC100057191,LOC100057110,LOC100146600,LOC100057026,LOC100050714,LOC100056989,LOC100056947,LOC100056906,LOC100056869,LOC100146809,LOC100067978,LOC100067957,LOC100056826,LOC100056793,LOC100056755,LOC100147000,LOC100056708,LOC100056626,LOC100056578,LOC100056531,LOC100056488,LOC100629158,LOC100050633,LOC100056395,LOC100056356,LOC100056314,LOC100056273,LOC100056235,LOC100056187,LOC100056146
12	14108229	14128309	20081	1,3,4	1,2,1	LOC100056626,LOC100056578
12	14108229	14297056	188828	0	1	LOC100056869,LOC100146809,LOC100067978,LOC100067957,LOC100056826,LOC100056793,LOC100056755,LOC100147000,LOC100056708,LOC100056626,LOC100056578
12	14108229	14391372	283144	4	2	LOC100057026,LOC100050714,LOC100056989,LOC100056947,LOC100056906,LOC100056869,LOC100146809,LOC100067978,LOC100067957,LOC100056826,LOC100056793,LOC100056755,LOC100147000,LOC100056708,LOC100056626,LOC100056578
12	14108229	14777981	669753	3,4	3,1	LOC100058077,LOC100147282,LOC100058036,LOC100057989,LOC100057945,LOC100057894,LOC100057845,LOC100057804,LOC100057756,LOC100057709,LOC100057670,LOC100057589,LOC100057547,LOC100057513,LOC100057472,LOC100057433,LOC100057393,LOC100057352,LOC100057310,LOC100147286,LOC100146990,LOC100057269,LOC100057228,LOC100057191,LOC100057110,LOC100146600,LOC100057026,LOC100050714,LOC100056989,LOC100056947,LOC100056906,LOC100056869,LOC100146809,LOC100067978,LOC100067957,LOC100056826,LOC100056793,LOC100056755,LOC100147000,LOC100056708,LOC100056626,LOC100056578
12	14108229	15073209	964981	3	1	LOC100058472,LOC100058429,LOC100058385,LOC100058346,LOC100146506,LOC100058253,LOC100147086,LOC100630034,LOC100058212,LOC100058168,LOC100058123,LOC100058077,LOC100147282,LOC100058036,LOC100057989,LOC100057945,LOC100057894,LOC100057845,LOC100057804,LOC100057756,LOC100057709,LOC100057670,LOC100057589,LOC100057547,LOC100057513,LOC100057472,LOC100057433,LOC100057393,LOC100057352,LOC100057310,LOC100147286,LOC100146990,LOC100057269,LOC100057228,LOC100057191,LOC100057110,LOC100146600,LOC100057026,LOC100050714,LOC100056989,LOC100056947,LOC100056906,LOC100056869,LOC100146809,LOC100067978,LOC100067957,LOC100056826,LOC100056793,LOC100056755,LOC100147000,LOC100056708,LOC100056626,LOC100056578
12	14124730	14391372	266643	3	1	LOC100057026,LOC100050714,LOC100056989,LOC100056947,LOC100056906,LOC100056869,LOC100146809,LOC100067978,LOC100067957,LOC100056826,LOC100056793,LOC100056755,LOC100147000,LOC100056708

Chrom	Start	End	Size	Cn	Samples (n)	Genes
12	14297056	14391372	94317	4	1	LOC100057026,LOC100050714,LOC100056989,LOC100056947,LOC100056906
12	14297056	14777981	480926	3	1	LOC100058077,LOC100147282,LOC100058036,LOC100057989,LOC100057945,LOC100057894,LOC100057845,LOC100057804,LOC100057756,LOC100057709,LOC100057670,LOC100057589,LOC100057547,LOC100057513,LOC100057472,LOC100057433,LOC100057393,LOC100057352,LOC100057310,LOC100147286,LOC100146990,LOC100057269,LOC100057228,LOC100057191,LOC100057110,LOC100146600,LOC100057026,LOC100050714,LOC100056989,LOC100056947,LOC100056906
12	14354314	14777981	423668	3	1	LOC100058077,LOC100147282,LOC100058036,LOC100057989,LOC100057945,LOC100057894,LOC100057845,LOC100057804,LOC100057756,LOC100057709,LOC100057670,LOC100057589,LOC100057547,LOC100057513,LOC100057472,LOC100057433,LOC100057393,LOC100057352,LOC100057310,LOC100147286,LOC100146990,LOC100057269,LOC100057228,LOC100057191,LOC100057110,LOC100146600,LOC100057026,LOC100050714,LOC100056989
12	14587232	14777981	190750	1	1	LOC100058077,LOC100147282,LOC100058036,LOC100057989,LOC100057945,LOC100057894,LOC100057845,LOC100057804,LOC100057756,LOC100057709,LOC100057670,LOC100057589,LOC100057547,LOC100057513
12	15070581	15778263	707683	3	1	LOC100065474,LOC100062694,LOC100065451,LOC100065423,LOC100065402,LOC100065380,LOC100065349,LOC100065319,LOC100065288,LOC100146469,LOC100065263,LOC100147060,LOC100065230,LOC100065201,LOC100065168,LOC100065136,LOC100065111,LOC100065079,LOC100065048,LOC100065019,LOC100630301,LOC100064956,LOC100064924,LOC100062657,LOC100064900,LOC100064874,LOC100064848,LOC100064820,LOC100064792,LOC100064769,LOC100064716,LOC100064690,LOC100062590,LOC100062626,LOC100064662,LOC100630007,LOC100064597,LOC100064562
12	15070581	15968363	897783	3	1	LOC100065858,LOC100065831,LOC100065803,LOC100065755,LOC100065735,LOC100065693,LOC100065674,LOC100065655,LOC100065634,LOC100146350,LOC100065610,LOC100065591,LOC100065549,LOC100146953,LOC100065500,LOC100065474,LOC100062694,LOC100065451,LOC100065423,LOC100065402,LOC100065380,LOC100065349,LOC100065319,LOC100065288,LOC100146469,LOC100065263,LOC100147060,LOC100065230,LOC100065201,LOC100065168,LOC100065136,LOC100065111,LOC100065079,LOC100065048,LOC100065019,LOC100630301,LOC100064956,LOC100064924,LOC100062657,LOC100064900,LOC100064874,LOC100064848,LOC100064820,LOC100064792,LOC100064769,LOC100064716,LOC100064690,LOC100062590,LOC100062626,LOC100064662,LOC100630007,LOC100064597,LOC100064562
12	15070581	16608208	1537628	3	1	LOC100066553,LOC100066503,LOC100066447,LOC100066417,LOC100066361,LOC100066225,LOC100066190,LOC100066164,LOC100630861,LOC100066108,LOC100066068,LOC100147059,LOC100066040,LOC100062734,LOC100066011,LOC100065987,LOC100146657,LOC100065959,LOC100065934,LOC100147543,LOC100065911,LOC100065887,LOC100065858,LOC100065831,LOC100065803,LOC100065755,LOC100065735,LOC100065693,LOC100065674,LOC100065655,LOC100065634,LOC100146350,LOC100065610,LOC100065591,LOC100065549,LOC100146953,LOC100065500,LOC100065474,LOC100062694,LOC100065451,LOC100065423,LOC100065402,LOC100065380,LOC100065349,LOC100065319,LOC100065288,LOC100146469,LOC100065263,LOC100147060,LOC100065230,LOC100065201,LOC100065168,LOC100065136,LOC100065111,LOC100065079,LOC100065048,LOC100065019,LOC100630301,LOC100064956,LOC100064924,LOC100062657,LOC100064900,LOC100064874,LOC100064848,LOC100064820,LOC100064792,LOC100064769,LOC100064716,LOC100064690,LOC100062590,LOC100062626,LOC100064662,LOC100630007,LOC100064597,LOC100064562
12	15070581	17011411	1940831	3	1	LRRC55,LOC100066827,LOC100066804,LOC100066784,LOC100066742,LOC100147445,LOC100062766,LOC100066715,LOC100066690,LOC100066643,LOC100066553,LOC100066503,LOC100066447,LOC100066417,LOC100066361,LOC100066225,LOC100066190,LOC100066164,LOC100630861,LOC100066108,LOC100066068,LOC100147059,LOC100066040,LOC100062734,LOC100066011,LOC100065987,LOC100146657,LOC100065959,LOC100065934,LOC100147543,LOC100065911,LOC100065887,LOC100065858,LOC100065831,LOC100065803,LOC100065755,LOC100065735,LOC100065693,LOC100065674,LOC100065655,LOC100065634,LOC100146350,LOC100065610,LOC100065591,LOC100065549,LOC100146953,LOC100065500,LOC100065474,LOC100062694,LOC100065451,LOC100065423,LOC100065402,LOC100065380,LOC100065349,LOC100065319,LOC100065288,LOC100146469,LOC100065263,LOC100147060,LOC100065230,LOC100065201,LOC100065168,LOC100065136,LOC100065111,LOC100065079,LOC100065048,LOC100065019,LOC100630301,LOC100064956,LOC100064924,LOC100062657,LOC100064900,LOC100064874,LOC100064848,LOC100064820,LOC100064792,LOC100064769,LOC100064716,LOC100064690,LOC100062590,LOC100062626,LOC100064662,LOC100630007,LOC100064597,LOC100064562
12	15070581	17084040	2013460	3	1	SSRP1,TNKS1BP1,LOC100066889,LRRC55,LOC100066827,LOC100066804,LOC100066784,LOC100066742,LOC100147445,LOC100062766,LOC100066715,LOC100066690,LOC100066643,LOC100066553,LOC100066503,LOC100066447,LOC100066417,LOC100066361,LOC100066225,LOC100066190,LOC100066164,LOC100630861,LOC100066108,LOC100066068,LOC100147059,LOC100066040,LOC100062734,LOC100066011,LOC100065987,LOC100146657,LOC100065959,LOC100065934,LOC100147543,LOC100065911,LOC100065887,LOC100065858,LOC100065831,LOC100065803,LOC100065755,LOC100065735,LOC100065693,LOC100065674,LOC100065655,LOC100065634,LOC100146350,LOC100065610,LOC100065591,LOC100065549,LOC100146953,LOC100065500,LOC100065474,LOC100062694,LOC100065451,LOC100065423,LOC100065402,LOC100065380,LOC100065349,LOC100065319,LOC100065288,LOC100146469,LOC100065263,LOC100147060,LOC100065230,LOC100065201,LOC100065168,LOC100065136,LOC100065111,LOC100065079,LOC100065048,LOC100065019,LOC100630301,LOC100064956,LOC100064924,LOC100062657,LOC100064900,LOC100064874,LOC100064848,LOC100064820,LOC100064792,LOC100064769,LOC100064716,LOC100064690,LOC100062590,LOC100062626,LOC100064662,LOC100630007,LOC100064597,LOC100064562
12	15954830	16608208	653379	3	1	LOC100066553,LOC100066503,LOC100066447,LOC100066417,LOC100066361,LOC100066225,LOC100066190,LOC100066164,LOC100630861,LOC100066108,LOC100066068,LOC100147059,LOC100066040,LOC100062734,LOC100066011,LOC100065987,LOC100146657,LOC100065959,LOC100065934,LOC100147543,LOC100065911,LOC100065887,LOC100065858
12	16155847	16608208	452362	3	1	LOC100066553,LOC100066503,LOC100066447,LOC100066417,LOC100066361,LOC100066225,LOC100066190,LOC100066164,LOC100630861,LOC100066108
12	24308133	25795367	1487235	3	1	LOC100057444,PCNXL3,LOC100057319,LOC100051469,LOC100057282,LOC100057238,LOC100057163,LTBP3,SCYL1,LOC100629234,LOC100051398,DPF2,LOC100056801,POLA2,SLC22A20,LOC100051106,LOC100146776,SYVN1,LOC100056723,FAU,LOC100056638,TM7SF2,LOC100056545,LOC100056453,LOC100050887,LOC100056368,SAC3D1,LOC100050811,LOC100056286,LOC100056246,LOC100056117,PPP2R5B,LOC100050653,MIR194,MIR192,LOC100050576,LOC100056040,LOC100050500,MAP4K2,SF1,PYGM,LOC100055880,SLC22A12,LOC100050279,LOC100147442,LOC100050210,LOC100055615,LOC100055657,LOC100055490,LOC100050142,LOC100055447,PLCB3,LOC100055365,LOC100055318,LOC100055238,DNAJC4,LOC100050076,LOC100055143,FERMT3,LOC100055100,FLRT1,LOC100054829,LOC100630578,LOC100054687

Chrom	Start	End	Size	Cn	Samples (n)	Genes
12	24401247	24677514	276268	4	1	LOC100050279,LOC100147442,LOC100050210,LOC100055615,LOC100055657,LOC100055490,LOC100050142,LOC100055447,PLCB3,LOC100055365,LOC100055318,LOC100055238,DNAJC4,LOC100050076,LOC100055143,FERMT3,LOC100055100,FLRT1
12	26859692	33087564	6227873	3	1	LOC100054956,LOC100051094,RIC8A,LOC100054908,PSMD13,LOC100629511,LOC100146460,LOC100146757,LOC100050873,LOC100050797,LOC100050719,LOC100054725,LOC100050637,LOC100050560,B4GALNT4,LOC100147635,LOC100054544,ANO9,PTDSS2,LOC100050482,LOC100053977,LOC100147536,LOC100146958,LOC100052102,LOC100051525,LOC100051383,MUC5AC,LOC100629257,LOC100056318,CHID1,LOC100055228,LOC100056151,LOC100055181,LOC100146758,LOC100147341,LOC100055133,LOC100147623,LOC100055991,LOC100055949,LOC100146964,LOC100055871,LOC100147552,LOC100630455,LOC100146575,LOC100055087,PHRF1,LOC100055649,LOC100055604,LRRC56,LOC100055481,LOC100055356,LOC100061640,LOC100147251,LOC100060138,LOC100060043,LOC100060163,LOC100060198,LOC100630277,LOC100060336,LOC100060376,LOC100060412,LOC100060480,LOC100630547,LOC100146349,LOC100146648,LOC100060077,IGF2,MIR675,H19,MRPL23,LOC100147259,LOC100630197,LOC100060979,LOC100061396,LOC100061284,DHCR7,LOC100061466,LOC100061705,CTTN,LOC100630363,LOC100061802,ANO1,LOC100146649,LOC100060525,LOC100060488,LOC100630094,MRGPRF,LOC100060175,LOC100060147,IGHMBP2,LOC100053582,CPT1A,LOC100060054,LOC100630057,SAPS3,TRNAA-AGC,LRP5,LOC100059902,SUV420H1,CHKA,LOC100146660,LOC100053392,ALDH3B1,UNC93B1,LOC100053296,LOC100147545,LOC100146252,LOC100146569,LOC100059569,LOC100053249,LOC100059533,CABP2,LOC100146465,LOC100059453,AIP,LOC100147448,LOC100059382,GPR152,CORO1B,LOC100053058,LOC100053005,CARNS1,TBC1D10C,PPP1CA,LOC100059194,LOC100146651,LOC100059111,LOC100059077,ANKRD13D,LOC100059001,KDM2A,LOC100058917,SYT12,LOC100629674
12	27356605	33087564	5730960	3	1	LOC100054956,LOC100051094,RIC8A,LOC100054908,PSMD13,LOC100629511,LOC100146460,LOC100146757,LOC100050873,LOC100050797,LOC100050719,LOC100054725,LOC100050637,LOC100050560,B4GALNT4,LOC100147635,LOC100054544,ANO9,PTDSS2,LOC100050482,LOC100053977,LOC100147536,LOC100146958,LOC100052102,LOC100051525,LOC100051383,MUC5AC,LOC100629257,LOC100056318,CHID1,LOC100055228,LOC100056151,LOC100055181,LOC100146758,LOC100147341,LOC100055133,LOC100147623,LOC100055991,LOC100055949,LOC100146964,LOC100055871,LOC100147552,LOC100630455,LOC100146575,LOC100055087,PHRF1,LOC100055649,LOC100055604,LRRC56,LOC100055481,LOC100055356,LOC100061640,LOC100147251,LOC100060138,LOC100060043,LOC100060163,LOC100060198,LOC100630277,LOC100060336,LOC100060376,LOC100060412,LOC100060480,LOC100630547,LOC100146349,LOC100146648,LOC100060077,IGF2,MIR675,H19,MRPL23,LOC100147259,LOC100630197,LOC100060979,LOC100061396,LOC100061284,DHCR7,LOC100061466,LOC100061705,CTTN,LOC100630363,LOC100061802,ANO1,LOC100146649,LOC100060525,LOC100060488,LOC100630094,MRGPRF,LOC100060175,LOC100060147,IGHMBP2,LOC100053582,CPT1A,LOC100060054,LOC100630057,SAPS3,TRNAA-AGC,LRP5,LOC100059902,SUV420H1,CHKA,LOC100146660,LOC100053392,ALDH3B1,UNC93B1
12	30065284	32624253	2558970	4	1	LOC100052102,LOC100051525,LOC100051383,MUC5AC,LOC100629257,LOC100056318,CHID1,LOC100055228,LOC100056151,LOC100055181,LOC100146758,LOC100147341,LOC100055133,LOC100147623,LOC100055991,LOC100055949,LOC100146964,LOC100055871,LOC100147552,LOC100630455,LOC100146575,LOC100055087,PHRF1,LOC100055649,LOC100055604,LRRC56,LOC100055481,LOC100055356,LOC100061640,LOC100147251,LOC100060138,LOC100060043,LOC100060163,LOC100060198,LOC100630277,LOC100060336,LOC100060376,LOC100060412,LOC100060480,LOC100630547,LOC100146349,LOC100146648,LOC100060077,IGF2,MIR675,H19,MRPL23,LOC100147259,LOC100630197,LOC100060979,LOC100061396,LOC100061284
12	33067635	33087564	19930	1	1	LOC100054956,LOC100051094,RIC8A,LOC100054908
13	134277	1140437	1006161	4	1	LOC100629883,LOC100062881,LOC100062989,LOC100063055,TRRAP,SMURF1,KPNA7,ARPC1A,ARPC1B,LOC100063675,LOC100059325,LOC100063716,LOC100059364,LOC100059401
13	2032780	2038953	6174	1	1	LOC100061463
13	4107284	4616688	509405	4	1	LOC100059919,LOC100058937,AMZ1,LOC100060005,LOC100058981
13	6663555	9342079	2678525	4	1	LOC100060372,LOC100060333,LOC100060265,LOC100059592,LOC100059555,LOC100059516,PLOD3,LOC100060232,LOC100059438,LOC100059402,LOC100060194,PAI-1,TRIM56,LOC100147691,LOC100146427,LOC100069149,LOC100147412,SRRT,LOC100069114,SLC12A9,EPHB4,ZAN,EPO,LOC100067533,GIGYF1,GNB2,LOC100069035,TFR2,MOSPD3,PCOLCE,LOC100067395,LRCH4,LOC100630713,LOC100069009,AGFG2,LOC100067349,LOC100068974,LOC100068962,LOC100068948,MEPCE,ZCWPW1,LOC100068911,STAG3,LOC100146435,LOC100067323,LOC100147030,LOC100630492,LOC100068805,LOC100068786,LOC100068735,LOC100068717,TAF6,AP4M1,MIR106B,MIR93,MIR25,LOC100068673,LOC100068657,ZNF3,LOC100629620,LOC100068562,ZKSCAN1,LOC100067197,LOC100068490,LOC100068438,LOC100068290,LOC100068271,LOC100067175,LOC100068178,LOC100068159,LOC100068123,CYP3A97,CYP3A96,CYP3A95,CYP3A94,CYP3A89,CYP3A93,LOC100067876,ZNF498,LOC100630140,LOC100067831,LOC100147118,LOC100066997,ZNF394,LOC100067741
13	6663555	10430960	3767406	4	1	LOC100629706,LOC100059763,LOC100059731,SRCRB4D,LOC100060941,DTX2,LOC100060861,LOC100060825,RASA4,LOC100059695,LRWD1,LOC100059659,LOC100060645,LOC100059630,SH2B2,CUX1,LOC100060444,LOC100060372,LOC100060333,LOC100060265,LOC100059592,LOC100059555,LOC100059516,PLOD3,LOC100060232,LOC100059438,LOC100059402,LOC100060194,PAI-1,TRIM56,LOC100147691,LOC100146427,LOC100069149,LOC100147412,SRRT,LOC100069114,SLC12A9,EPHB4,ZAN,EPO,LOC100067533,GIGYF1,GNB2,LOC100069035,TFR2,MOSPD3,PCOLCE,LOC100067395,LRCH4,LOC100630713,LOC100069009,AGFG2,LOC100067349,LOC100068974,LOC100068962,LOC100068948,MEPCE,ZCWPW1,LOC100068911,STAG3,LOC100146435,LOC100067323,LOC100147030,LOC100630492,LOC100068805,LOC100068786,LOC100068735,LOC100068717,TAF6,AP4M1,MIR106B,MIR93,MIR25,LOC100068673,LOC100068657,ZNF3,LOC100629620,LOC100068562,ZKSCAN1,LOC100067197,LOC100068490,LOC100068438,LOC100068290,LOC100068271,LOC100067175,LOC100068178,LOC100068159,LOC100068123,CYP3A97,CYP3A96,CYP3A95,CYP3A94,CYP3A89,CYP3A93,LOC100067876,ZNF498,LOC100630140,LOC100067831,LOC100147118,LOC100066997,ZNF394,LOC100067741

Chrom	Start	End	Size	Cn	Samples (n)	Genes
13	6917885	9034502	2116618	4	1	LOC100147691,LOC100146427,LOC100069149,LOC100147412,SRRT,LOC100069114,SLC12A9,EPHB4,ZAN,EPO,LOC100067533,GIGYF1,GNB2,LOC100069035,TFR2,MOSPD3,PCOLCE,LOC100067395,LRCH4,LOC100630713,LOC100069009,AGFG2,LOC100067349,LOC100068974,LOC100068962,LOC100068948,MEPCE,ZCWPW1,LOC100068911,STAG3,LOC100146435,LOC100067323,LOC100147030,LOC100630492,LOC100068805,LOC100068786,LOC100068735,LOC100068717,TAF6,AP4M1,MIR106B,MIR93,MIR25,LOC100068673,LOC100068657,ZNF3,LOC100629620,LOC100068562,ZKSCAN1,LOC100067197,LOC100068490,LOC100068438,LOC100068290,LOC100068271,LOC100067175,LOC100068178,LOC100068159,LOC100068123,CYP3A97,CYP3A96,CYP3A95,CYP3A94,CYP3A89,CYP3A93,LOC100067876,ZNF498
13	8846442	10430960	1584519	3	1	LOC100629706,LOC100059763,LOC100059731,SRCRB4D,LOC100060941,DTX2,LOC100060861,LOC100060825,RASA4,LOC100059695,LRWD1,LOC100059659,LOC100060645,LOC100059630,SH2B2,CUX1,LOC100060444,LOC100060372,LOC100060333,LOC100060265,LOC100059592,LOC100059555,LOC100059516,PLOD3,LOC100060232,LOC100059438,LOC100059402,LOC100060194,PAI-1,TRIM56,LOC100147691,LOC100146427
13	9183989	10373343	1189355	3	1	LOC100629706,LOC100059763,LOC100059731,SRCRB4D,LOC100060941,DTX2,LOC100060861,LOC100060825,RASA4,LOC100059695,LRWD1,LOC100059659,LOC100060645,LOC100059630,SH2B2,CUX1,LOC100060444,LOC100060372,LOC100060333,LOC100060265
13	13961628	13968758	7131	1	2	
13	18726953	19939229	1212277	3	1	LOC100066402,ATP2A1,SH2B1,LOC100064341,ATXN2L,EIF3C,LOC100064282,LOC100066345,LOC100066317,LOC100064252,LOC100064194,LOC100064221,LOC100066262,BOLA2,CORO1A,LOC100066204,MAPK3,LOC100146273,LOC100064143,LOC100066147,ALDOA,PPP4C,LOC100066097,LOC100629851,DOC2A,LOC100064084,HIRIP3,TAOK2,LOC100064015,LOC100065972,LOC100065947,SEZ6L2,LOC100065899,MVP,LOC100063953,LOC100065869,LOC100146790,LOC100065842,LOC100065789,LOC100063917,LOC100063884,LOC100065768,LOC100065743,LOC100065719,TBC1D10B,LOC100065683,SEPT1,LOC100629627,LOC100065576,LOC100065556,SEPHS2,ITGAL,LOC100065483,LOC100065389,LOC100065362,PRR14,LOC100063732,SRCAP,LOC100063692,PHKG2,C13H16orf93,RNF40,ZNF629,LOC100063618,LOC100065094,LOC100065061,FBXL19,LOC100063559,SETD1A,LOC100063491,LOC100146591,LOC100063460,ZNF668,ZNF646
13	18780851	18804175	23325	1	1	SETD1A,LOC100063491,LOC100146591
13	18780851	19691396	910546	4	2	CORO1A,LOC100066204,MAPK3,LOC100146273,LOC100064143,LOC100066147,ALDOA,PPP4C,LOC100066097,LOC100629851,DOC2A,LOC100064084,HIRIP3,TAOK2,LOC100064015,LOC100065972,LOC100065947,SEZ6L2,LOC100065899,MVP,LOC100063953,LOC100065869,LOC100146790,LOC100065842,LOC100065789,LOC100063917,LOC100063884,LOC100065768,LOC100065743,LOC100065719,TBC1D10B,LOC100065683,SEPT1,LOC100629627,LOC100065576,LOC100065556,SEPHS2,ITGAL,LOC100065483,LOC100065389,LOC100065362,PRR14,LOC100063732,SRCAP,LOC100063692,PHKG2,C13H16orf93,RNF40,ZNF629,LOC100063618,LOC100065094,LOC100065061,FBXL19,LOC100063559,SETD1A,LOC100063491,LOC100146591
13	28665470	32671716	4006247	3	1	LOC100055833,LOC100055746,LOC100055616,LOC100147264,LOC100050888,ERCC4,MKL2,MIR193B,MIR365-2,LOC100050655,BFAR,LOC100054324,RRN3,LOC100053937,LOC100053887,LOC100630548,LOC100053782,LOC100050501,LOC100050421,MYH11,LOC100630456,ABCC1,ABCC6,LOC100053583,XYLT1
13	31854826	33188451	1333626	3	1	LITAF,LOC100051037,TXNDC11,ZC3H7A,LOC100056041,GSPT1,LOC100055833,LOC100055746,LOC100055616,LOC100147264
13	39491095	42556495	3065401	3	1	IL9R,LOC100630131,LOC100066312,LOC100066340,LOC100065027,NPRL3,HBZ1,HBA,LOC100147150,LUC7L,LOC100066481,LOC100066511,LOC100146456,LOC100066556,AXIN1,LOC100066606,TMEM8A,LOC100066649,LOC100065146,LOC100066724,LOC100146243,PIGQ,LOC100066853,WFIKKN1,LOC100065210,TRNAG-CCC,LOC100629842,WDR90,RHOT2,RHBDL1,LOC100066977,LOC100066999,LOC100147047,WDR24,LOC100147621,LOC100147618,LOC100067088,LOC100067114,LOC100067135,LOC100065271,LOC100067178,LOC100067203,LOC100065297,LOC100067225,LOC100065327,LOC100067328,LOC100067353,LOC100067398,LOC100067422,LOC100067446,LOC100067492,LOC100067538,RET,LOC100065385,LOC100067554,LOC100065411,LOC100067580,LOC100067623,CCDC154,LOC100067691,PTX4,LOC100067764,LOC100065461,IFT140,LOC100065479,LOC100065505,MAPK8IP3,LOC100146137,LOC100146452,LOC100067860,LOC100065531,LOC100067912,LOC100067938,LOC100067958,LOC100146640,SEPX1,LOC100068030,LOC100065553,LOC100065572,LOC100068047,TBL3,LOC100068060,LOC100065617,LOC100065638,ZNF598,LOC100629171,LOC100146142,LOC100065659,TSC2,LOC100068126,RAB26,TRAF7,CASKIN1,LOC100068216,LOC100068232,MIR1842,LOC100068257,LOC100068273,LOC100065713,LOC100068292,LOC100065738,LOC100068313,LOC100068332,LOC100068378,LOC100068393,LOC100147434,TBC1D24,LOC100065783,LOC100068441,LOC100630736,LOC100068494,LOC100068526,LOC100068549,LOC100630678,LOC100630656,LOC100068644,LOC100630613,LOC100630594,LOC100068690,LOC100068704,LOC100068723,LOC100068741,LOC100068757,LOC100068774,LOC100068790,LOC100629991,LOC100068826,LOC100068842,LOC100146843,PKMYT1,LOC100067620,LOC100067643,LOC100146755,LOC100065863,LOC100068915,LOC100068930,LOC100068942,LOC100065894,ZSCAN10,LOC100068965,LOC100068977,TRNAR-CCG,TRNAR-CCU,TRNAK-CUU,TRNAP-UGG,TRNAP-AGG,TRNAK-CUU,TRNAP-AGG,TRNAK-CUU,TRNAP-UGG,TRNAK-CUU,TRNAP-CGG,TRNAR-CCU,TRNAK-CUU,TRNAK-CUU,TRNAP-UGG,TRNAP-AGG,TRNAK-CUU,TRNAP-AGG,TRNAR-CCU,TRNAK-CUU,LOC100069220,LOC100069235,LOC100069249,ZNF200,LOC100069286,ZNF263
13	39934602	42335152	2400551	1	1	LOC100066511,LOC100146456,LOC100066556,AXIN1,LOC100066606,TMEM8A,LOC100066649,LOC100065146,LOC100066724,LOC100146243,PIGQ,LOC100066853,WFIKKN1,LOC100065210,TRNAG-CCC,LOC100629842,WDR90,RHOT2,RHBDL1,LOC100066977,LOC100066999,LOC100147047,WDR24,LOC100147621,LOC100147618,LOC100067088,LOC100067114,LOC100067135,LOC100065271,LOC100067178,LOC100067203,LOC100065297,LOC100067225,LOC100065327,LOC100067328,LOC100067353,LOC100067398,LOC100067422,LOC100067446,LOC100067492,LOC100067538,RET,LOC100065385,LOC100067554,LOC100065411,LOC100067580,LOC100067623,CCDC154,LOC100067691,PTX4,LOC100067764,LOC100065461,IFT140,LOC100065479,LOC100065505,MAPK8IP3,LOC100146137,LOC100146452,LOC100067860,LOC100065531,LOC100067912,LOC100067938,LOC100067958,LOC100146640,SEPX1,LOC100068030,LOC100065553,LOC100065572,LOC100068047,TBL3,LOC100068060,LOC100065617,LOC100065638,ZNF598,LOC100629171,LOC100146142,LOC100065659,TSC2,LOC100068126,RAB26,TRAF7,CASKIN1,LOC100068216,LOC100068232,MIR1842,LOC100068257,LOC100068273,LOC100065713,LOC100068292,LOC100065738,LOC100068313,LOC100068332,LOC100068378,LOC100068393,LOC100147434,TBC1D24,LOC100065783,LOC100068441,LOC100630736,LOC100068494,LOC100068526,LOC100068549,LOC100630678,LOC100630656,LOC100068644,LOC100630613,LOC100630594,LOC100068690,LOC100068704,LOC100068723,LOC100068741

Chrom	Start	End	Size	Cn	Samples (n)	Genes
13	40469638	41049706	580069	1	1	MAPK8IP3,LOC100146137,LOC100146452,LOC100067860,LOC100065531,LOC100067912,LOC100067938,LOC100067958,LOC100146640,SEPX1,LOC100068030,LOC100065553,LOC100065572,LOC100068047,TBL3,LOC100068060,LOC100065617,LOC100065638,ZNF598,LOC100629171,LOC100146142,LOC100065659,TSC2,LOC100068126,RAB26,TRAF7,CASKIN1,LOC100068216,LOC100068232,MIR1842,LOC100068257,LOC100068273,LOC100065713,LOC100068292,LOC100065738,LOC100068313,LOC100068332
13	41856301	42285668	429368	1	1	AXIN1,LOC100066606,TMEM8A,LOC100066649,LOC100065146,LOC100066724,LOC100146243,PIGQ,LOC100066853,WFIKKN1,LOC100065210,TRNAG-CCC,LOC100629842,WDR90,RHOT2,RHBDL1,LOC100066977,LOC100066999,LOC100147047,WDR24,LOC100147621,LOC100147618,LOC100067088,LOC100067114,LOC100067135,LOC100065271,LOC100067178,LOC100067203,LOC100065297,LOC100067225,LOC100065327
13	42125751	42285668	159918	1	1	AXIN1,LOC100066606,TMEM8A,LOC100066649,LOC100065146,LOC100066724
14	2889669	4274260	1384592	3	1	LOC100068381,CLK4,LOC100068220,ZNF354A,LOC100068050,LOC100068033,LOC100068018,ZNF354B,LOC100067839,LOC100067731,ZNF879,LOC100630632,RUFY1
14	5315935	6313114	997180	3	1	LOC100058810,LOC100058774,LOC100068979,LOC100068955,LOC100058730,MIR1271,LOC100068932,LOC100058691,LOC100068903,LOC100068882,FAF2,LOC100629300,RNF44,CDHR2,LOC100068777,LOC100629228,LOC100068758,LOC100146751
14	17586672	17826590	239919	1	1	GABRG2
14	17713486	17758781	45296	1	1	
14	17713486	17826590	113105	1	1	GABRG2
14	26211323	28493479	2282157	3	1	PCYOX1L,LOC100060593,MIR143,MIR145,CSNK1A1,LOC100071691,MIR378,PPARGC1B,LOC100060489,SLC26A2,TIGD14,HMGXB3,CSF1R,LOC100071665,LOC100071660,LOC100060390,CAMK2A,LOC100071647,TCOF1,LOC100071644,LOC100071636,LOC100630629,NDST1,SYNPO,LOC100060280,LOC100060252,DCTN4,LOC100630236,LOC100071601,LOC100071598,LOC100071590,GPX3,TNIP1,ANXA6,LOC100071558,GM2A,SLC36A3,SLC36A2,SLC36A1,LOC100071515,SPARC,LOC100071499,LOC100060090,GLRA1,LOC100630423
14	26477000	27547553	1070554	3	1	NDST1,SYNPO,LOC100060280,LOC100060252,DCTN4,LOC100630236,LOC100071601,LOC100071598,LOC100071590,GPX3,TNIP1,ANXA6,LOC100071558,GM2A,SLC36A3,SLC36A2,SLC36A1,LOC100071515,SPARC
14	26527029	28493479	1966451	4	1	PCYOX1L,LOC100060593,MIR143,MIR145,CSNK1A1,LOC100071691,MIR378,PPARGC1B,LOC100060489,SLC26A2,TIGD14,HMGXB3,CSF1R,LOC100071665,LOC100071660,LOC100060390,CAMK2A,LOC100071647,TCOF1,LOC100071644,LOC100071636,LOC100630629,NDST1,SYNPO,LOC100060280,LOC100060252,DCTN4,LOC100630236,LOC100071601,LOC100071598,LOC100071590,GPX3,TNIP1,ANXA6,LOC100071558,GM2A,SLC36A3,SLC36A2,SLC36A1,LOC100071515,SPARC
14	27431061	28884750	1453690	3	1	SH3TC2,ABLIM3,LOC100071710,LOC100071700,PCYOX1L,LOC100060593,MIR143,MIR145,CSNK1A1,LOC100071691,MIR378,PPARGC1B,LOC100060489,SLC26A2,TIGD14,HMGXB3,CSF1R,LOC100071665,LOC100071660,LOC100060390,CAMK2A,LOC100071647,TCOF1,LOC100071644,LOC100071636,LOC100630629,NDST1
14	32162507	32166676	4170	1	1	
14	34605933	34794798	188866	1	1	
14	34605933	35308148	702216	1	1	LOC100072169,PCDH1,LOC100072156,PCDH12,LOC100061332,LOC100061298,LOC100072138,LOC100072121
14	35162634	35223848	61215	1	1	PCDH1
14	36755452	36765044	9593	1	2	LOC100072444
14	40163696	43242410	3078715	3	1	SLC22A4,SLC22A5,LOC100063253,IL5,RAD50,IL-13,IL4,KIF3A,SEPT8,LOC100063101,LOC100072831,GDF9,LOC100629810,AFF4,HSPA4,FSTL4,LOC100072795,LOC100062974,LOC100072786,LOC100062946,LOC100072780,CDKL3,LOC100062920,LOC100072766,PHF15,LOC100062866,SEC24A,LOC100062812,DDX46,LOC100062781,LOC100072729,LOC100630290,LOC100072710,LOC100072706,H2AFY,LOC100072703,LOC100072700,LOC100072697,LOC100072690,LOC100062710,LOC100072685
14	41275242	42044346	769105	3	1	LOC100072795,LOC100062974,LOC100072786,LOC100062946,LOC100072780,CDKL3,LOC100062920,LOC100072766,PHF15
14	41275242	42378987	1103746	4	1	FSTL4,LOC100072795,LOC100062974,LOC100072786,LOC100062946,LOC100072780,CDKL3,LOC100062920,LOC100072766,PHF15
14	41622288	43242410	1620123	3	1	SLC22A4,SLC22A5,LOC100063253,IL5,RAD50,IL-13,IL4,KIF3A,SEPT8,LOC100063101,LOC100072831,GDF9,LOC100629810,AFF4,HSPA4,FSTL4,LOC100072795,LOC100062974,LOC100072786
14	41779891	42378987	599097	3	1	FSTL4
14	42232591	42378987	146397	3	1	FSTL4
14	54951956	55309228	357273	1	1	
14	59460837	59463001	2165	3	6	
14	60192501	60678855	486355	1	1	TSLP,WDR36,LOC100073192,LOC100073191
14	64189586	65560963	1371378	1	1	LOC100073204,LOC100073203
14	64218405	65370657	1152253	1	1	LOC100073204,LOC100073203
14	64664037	65086623	422587	1	1	
14	64933907	64951664	17758	1	1	
14	64933907	65086623	152717	3	1	
14	80491410	81334051	842642	1	1	LOC100073245
14	85242665	85274390	31726	1	1	LOC100073258,ANKRD34B
14	85989014	86057100	68087	1	1	HOMER1

Chrom	Start	End	Size	Cn	Samples (n)	Genes
14	90182169	93832868	3650700	3	1	LOC100060865,LOC100061083,WNT9A,LOC100061145,LOC100060911,LOC100629259,LOC100061285,LOC100147433,LOC100146449,LOC100061497,LOC100060943,LOC100061535,LOC100061017,LOC100061573,LOC100060980,LOC100061639,LOC100061672,LOC100061707,LOC100073324,EQUCABV1R-PS925,ZNF496,LOC100073322,LOC100073321,LOC100073320,LOC100073319,LOC100146557,LOC100073315,LOC100073313,LOC100073312,LOC100630679,LOC100073310,LOC100073309,LOC100073308,RAD17,MARVELD2,OCLN,GTF2H2,NAIP,LOC100065744,LOC100630550,LOC100073302,LOC100073301,LOC100073300,LOC100065720,LOC100073298,LOC100073297,ZNF366,LOC100073295,TNPO1,FCHO2,LOC100065684,LOC100073293,LOC100065663,LOC100073292,LOC100065643,LOC100073291
15	3886181	5192396	1306216	3,4	1,1	LOC100056002,LOC100050078,TGFBRAP1,LOC100050013,LOC100049947,NCK2,C15H2orf40,UXS1
15	12041831	12394965	353135	3	1	ADRA2B,ASTL,LOC100051611,STARD7,LOC100062454,CIAO1,SNRNP200,LOC100062394,NCAPH,LOC100062289,LOC100062248
15	21073741	21139258	65518	1	3	
15	25166888	26582817	1415930	1	1	LOC100068530
15	26042054	26582817	540764	1	1	LOC100068530
15	35232680	35233452	773	1	1	
15	38257100	38770379	513280	1	1	EHBP1,LOC100051685,LOC100064050,LOC100063987
15	43024601	43642747	618147	1	1	
15	43285422	43632884	347463	1	1	
15	43306470	43474526	168057	1	1	
15	43306470	43632884	326415	1	1	
15	46663949	48337765	1673817	1	1	LOC100052856,MIR1261
15	47039045	47821545	782501	1	1	MIR1261
15	48199824	48278494	78671	1	1	
15	51937699	51973518	35820	4	1	TTC7A
15	55332517	55553663	221147	4	1	LOC100069207
15	56513560	57018352	504793	1	1	LOC100629144
15	64551633	64775259	223627	1	1	LOC100147678,LOC100054555,LOC100054514,LOC100070614
15	64551633	64960936	409304	1	3	LOC100147678,LOC100054555,LOC100054514,LOC100070614
15	77767792	77771242	3451	1	1	
16	31097426	31105067	7642	1	1	
16	33875613	38391940	4516328	4	1	MIR711,COL7A1,LOC100053786,LOC100629726,LOC100063620,LOC100146927,IP6K2,LOC100146116,LOC100063527,ARIH2,P4HTM,WDR6,DALRD3,MIR191,LOC100063288,IMPDH2,LOC100063232,LOC100053641,USP19,LAMC1,LOC100146127,LOC100063133,KLHDC8B,LOC100063006,CCDC36,LOC100053494,USP4,GPX1,LOC100053343,LOC100053301,AMT,LOC100053200,DAG1,BSN,APEH,RNF123,LOC100062712,LOC100052959,IP6K1,LOC100062639,CDHR4,LOC100052858,UBA7,LOC100630702,TRAIP,CAMKV,LOC100062566,MST1R,MON1A,LOC100630308,RBM6,RBM5,SEMA3F,LOC100062183,SLC38A3,GNAI2,SEMA3B,LOC100062012,LOC100061975,HYAL3,LOC100061906,HYAL1,HYAL2,LOC100052473,LOC100061779,LOC100146221,LOC100061750,LOC100061714,LOC100146836,LOC100061645,LOC100052357,LOC100061549,LOC100061510,LOC100061475,LOC100061446,DOCK3,MANF,LOC100061254,VPRBP,RAD54L2,LOC100147234,GRM2,LOC100060959,LOC100060924,LOC100060881,LOC100060840,LOC100629486,RRP9,PARP3,LOC100147130,LOC100060660,LOC100052116,LOC100629427,ACY1,LOC100052055,LOC100060559,LOC100060527,ALAS1,TLR9,TWF2,LOC100060391,MIRLET7G,LOC100060351,GLYCTK,MIR135A,LOC100630730,DNAH1,BAP1,PHF7,SEMA3G,LOC100051811,NISCH,STAB1,NT5DC2,LOC100629375,PBRM1,LOC100060056,LOC100051614,LOC100629294,NEK4,LOC100051474,ITIH3,ITIH4,LOC100059872,SFMBT1,RFT1,LOC100051333,TKT,DCP1A,CACNA1D,CHDH,LOC100059421,LOC100059386,SELK
16	34156020	37599838	3443819	3	1	BSN,APEH,RNF123,LOC100062712,LOC100052959,IP6K1,LOC100062639,CDHR4,LOC100052858,UBA7,LOC100630702,TRAIP,CAMKV,LOC100062566,MST1R,MON1A,LOC100630308,RBM6,RBM5,SEMA3F,LOC100062183,SLC38A3,GNAI2,SEMA3B,LOC100062012,LOC100061975,HYAL3,LOC100061906,HYAL1,HYAL2,LOC100052473,LOC100061779,LOC100146221,LOC100061750,LOC100061714,LOC100146836,LOC100061645,LOC100052357,LOC100061549,LOC100061510,LOC100061475,LOC100061446,DOCK3,MANF,LOC100061254,VPRBP,RAD54L2,LOC100147234,GRM2,LOC100060959,LOC100060924,LOC100060881,LOC100060840,LOC100629486,RRP9,PARP3,LOC100147130,LOC100060660,LOC100052116,LOC100629427,ACY1,LOC100052055,LOC100060559,LOC100060527,ALAS1,TLR9,TWF2,LOC100060391,MIRLET7G,LOC100060351,GLYCTK,MIR135A,LOC100630730,DNAH1,BAP1,PHF7,SEMA3G,LOC100051811,NISCH,STAB1,NT5DC2,LOC100629375,PBRM1,LOC100060056,LOC100051614,LOC100629294,NEK4,LOC100051474,ITIH3,ITIH4,LOC100059872,SFMBT1,RFT1,LOC100051333,TKT,DCP1A,CACNA1D
16	34652647	38391940	3739294	3	2	MIR711,COL7A1,LOC100053786,LOC100629726,LOC100063620,LOC100146927,IP6K2,LOC100146116,LOC100063527,ARIH2,P4HTM,WDR6,DALRD3,MIR191,LOC100063288,IMPDH2,LOC100063232,LOC100053641,USP19,LAMC1,LOC100146127,LOC100063133,KLHDC8B,LOC100063006,CCDC36,LOC100053494,USP4,GPX1,LOC100053343,LOC100053301,AMT,LOC100053200,DAG1,BSN,APEH,RNF123,LOC100062712,LOC100052959,IP6K1,LOC100062639,CDHR4,LOC100052858,UBA7,LOC100630702,TRAIP,CAMKV,LOC100062566,MST1R,MON1A,LOC100630308,RBM6,RBM5,SEMA3F,LOC100062183,SLC38A3,GNAI2,SEMA3B,LOC100062012,LOC100061975,HYAL3,LOC100061906,HYAL1,HYAL2,LOC100052473,LOC100061779,LOC100146221,LOC100061750,LOC100061714,LOC100146836,LOC100061645,LOC100052357,LOC100061549,LOC100061510,LOC100061475,LOC100061446,DOCK3,MANF,LOC100061254,VPRBP,RAD54L2,LOC100147234,GRM2,LOC100060959,LOC100060924,LOC100060881,LOC100060840,LOC100629486,RRP9,PARP3,LOC100147130,LOC100060660,LOC100052116,LOC100629427,ACY1,LOC100052055,LOC100060559,LOC100060527,ALAS1,TLR9,TWF2,LOC100060391,MIRLET7G,LOC100060351,GLYCTK,MIR135A,LOC100630730,DNAH1,BAP1,PHF7,SEMA3G,LOC100051811,NISCH,STAB1,NT5DC2,LOC100629375,PBRM1,LOC100060056,LOC100051614,LOC100629294,NEK4,LOC100051474,ITIH3,ITIH4,LOC100059872,SFMBT1,RFT1

Chrom	Start	End	Size	Cn	Samples (n)	Genes
16	35286678	36867056	1580379	3	1	LOC100061645,LOC100052357,LOC100061549,LOC100061510,LOC100061475,LOC100061446,DOCK3,MANF,LOC100061254,VPRBP,RAD54L2,LOC100147234,GRM2,LOC100060959,LOC100060924,LOC100060881,LOC100060840,LOC100629486,RRP9,PARP3,LOC100147130,LOC100060660,LOC100052116,LOC100629427,ACY1,LOC100052055,LOC100060559,LOC100060527,ALAS1,TLR9,TWF2,LOC100060391,MIRLET7G,LOC100060351,GLYCTK,MIR135A,LOC100630730,DNAH1,BAP1,PHF7
16	36159889	38488369	2328481	3	1	ATRIP,LOC100063767,LOC100063734,PFKFB4,LOC100630156,MIR711,COL7A1,LOC100053786,LOC100629726,LOC100063620,LOC100146927,IP6K2,LOC100146116,LOC100063527,ARIH2,P4HTM,WDR6,DALRD3,MIR191,LOC100063288,IMPDH2,LOC100063232,LOC100053641,USP19,LAMC1,LOC100146127,LOC100063133,KLHDC8B,LOC100063006,CCDC36,LOC100053494,USP4,GPX1,LOC100053343,LOC100053301,AMT,LOC100053200,DAG1,BSN,APEH,RNF123,LOC100062712,LOC100052959,IP6K1,LOC100062639,CDHR4,LOC100052858,UBA7,LOC100630702,TRAIP,CAMKV,LOC100062566,MST1R,MON1A,LOC100630308,RBM6,RBM5,SEMA3F,LOC100062183,SLC38A3,GNAI2,SEMA3B,LOC100062012,LOC100061975,HYAL3,LOC100061906,HYAL1,HYAL2,LOC100052473,LOC100061779,LOC100146221,LOC100061750,LOC100061714,LOC100146836,LOC100061645,LOC100052357,LOC100061549,LOC100061510,LOC100061475,LOC100061446,DOCK3,MANF,LOC100061254,VPRBP
16	36620506	36867056	246551	3	1	LOC100061645,LOC100052357,LOC100061549,LOC100061510,LOC100061475
16	38646249	38748409	102161	1	1	ECATH-3,LOC100063988,LOC100630242
16	38714465	38748409	33945	0,1	1,1	
16	39172944	39970655	797712	1	1	LOC100054327,PTH1R,LOC100054188,NBEAL2,LOC100064912,SETD2,LOC100054139,KIF9,LOC100064728,LOC100064703,PTPN23,SCAP,LOC100064547,LOC100064520,SMARCC1
16	47816883	47827828	10946	1	2	GOLGA4
16	48604265	49785128	1180864	3	1	MIR128,ARPP21
16	55142549	55232051	89503	1	1	LOC100058444,LOC100058400
16	70180289	71071114	890826	3	1	EPHB1
16	79424659	79502415	77757	1	1	PLOD2
16	80679310	80729142	49833	1	1	
17	18604248	18922753	318506	1	1	LOC100069247,LOC100630841,LOC100054677,LOC100146458,FOXO1
17	31082282	31229201	146920	1	1	
17	31229132	31341563	112432	3	1	
17	31473311	33290509	1817199	3	1	LOC100630189,LOC100062568,LOC100062357,LOC100630118
17	32426470	32654057	227588	3	1	
17	36846001	36977325	131325	1	1	LOC100063921
17	36846001	38412777	1566777	1	1	LOC100064376,LOC100064343,LOC100064316,LOC100064054,LOC100063921
17	37158169	38472830	1314662	1	1	LOC100064376,LOC100064343,LOC100064316,LOC100064054
17	40407642	40613435	205794	1	1	LOC100630661,LOC100630641
17	43527706	43886971	359266	1	1	KLHL1
17	49997510	51490247	1492738	1	1	LOC100049956,EDNRB,SLAIN1,SCEL,LOC100053014
17	52805734	53071134	265401	1	1	
17	52805734	61220361	8414628	1	1	LOC100050355,LOC100057448,LOC100057368,SLITRK5,SLITRK6,LOC100056206,SLITRK1,LOC100050152,LOC100055659
17	52880895	53716268	835374	1	1	
17	54202284	54921730	719447	1	1	LOC100050152,LOC100055659
17	56309236	57743864	1434629	1	1	SLITRK6,LOC100056206
17	56394003	57420379	1026377	1	1	SLITRK6,LOC100056206
17	57079825	57420379	340555	1	1	SLITRK6
17	57079825	57983714	903890	1	1	SLITRK6
17	57184436	57743864	559429	1	1	
17	57184436	58094803	910368	1	1	
17	57211170	57420379	209210	1	1	
17	57317295	57420379	103085	1	4	
17	57337550	57338181	632	1	1	
17	57337550	57420379	82830	1	1	
17	79582659	80647854	1065196	3	1	LOC100069263,LOC100630815,LOC100069151,LOC100069135,LOC100069163,LOC100067334,LOC100067313,LOC100067285,LOC100630737,LOC100067211,CUL4A,PCID2,LOC100067119,LOC100067094,LOC100067072,MCF2L,ATP11A
18	2910687	3956680	1045994	1	1	LOC100052625,BIN1,LOC100051659,LOC100050708,MAP3K2,PROC,LOC100051085,LOC100050863,IWS1,MYO7B,LOC100067531,WDR33,LOC100069214,AMMECR1L
18	7182586	7589140	406555	1	1	
18	8538709	11533872	2995164	3	1	LOC100629370,LOC100060942,LOC100072623,LOC100147415,LOC100060909,LOC100629172,LOC100629146,LOC100068987,CLASP1,LOC100055172,LOC100055077,LOC100147031,PTPN4,EPB41L5,TMEM185B,LOC100060199,LOC100067535,LOC100629454,LOC100067420,LOC100629385,LOC100146719,LOC100060304

Chrom	Start	End	Size	Cn	Samples (n)	Genes
18	9359667	11026066	1666400	3,4	1,1	LOC100629370,LOC100060942,LOC100072623,LOC100147415,LOC100060909,LOC100629172,LOC100629146,LOC100068987,EPB41L5,TMEM185B,LOC100060199,LOC100067535,LOC100629454,LOC100067420,LOC100629385,LOC100146719,LOC100060304
18	9407244	10881369	1474126	4	1	LOC100629370,LOC100060942,LOC100072623,LOC100147415,LOC100060909,LOC100629172,LOC100629146,LOC100067535,LOC100629454,LOC100067420,LOC100629385,LOC100146719,LOC100060304
18	9479897	11026066	1546170	3	4	LOC100629370,LOC100060942,LOC100072623,LOC100147415,LOC100060909,LOC100629172,EPB41L5,TMEM185B,LOC100060199,LOC100067535,LOC100629454,LOC100067420,LOC100629385,LOC100146719,LOC100060304
18	9746042	10758708	1012667	3	1	LOC100629370,LOC100629454,LOC100067420,LOC100629385,LOC100146719,LOC100060304
18	9746042	11026066	1280025	3	1	LOC100629370,EPB41L5,TMEM185B,LOC100060199,LOC100067535,LOC100629454,LOC100067420,LOC100629385,LOC100146719,LOC100060304
18	9746042	12161633	2415592	3	1	LOC100629370,LOC100052243,LOC100049893,STEAP3,LOC100629302,DBI,LOC100051693,LOC100051620,LOC100147031,PTPN4,EPB41L5,TMEM185B,LOC100060199,LOC100067535,LOC100629454,LOC100067420,LOC100629385,LOC100146719,LOC100060304
18	9856035	11533872	1677838	3	1	LOC100147031,PTPN4,EPB41L5,TMEM185B,LOC100060199,LOC100067535,LOC100629454,LOC100067420,LOC100629385,LOC100146719
18	10211221	10758708	547488	3	1	LOC100629454,LOC100067420
18	10340383	11026066	685684	3	1	EPB41L5,TMEM185B,LOC100060199,LOC100067535,LOC100629454,LOC100067420
18	10645374	12970620	2325247	3	1	LOC100049957,LOC100052304,LOC100052243,LOC100049893,STEAP3,LOC100629302,DBI,LOC100051693,LOC100051620,LOC100147031,PTPN4,EPB41L5,TMEM185B,LOC100060199,LOC100067535,LOC100629454
18	10645374	13650973	3005600	3	1	DDX18,LOC100052648,LOC100050087,CCDC93,LOC100049957,LOC100052304,LOC100052243,LOC100049893,STEAP3,LOC100629302,DBI,LOC100051693,LOC100051620,LOC100147031,PTPN4,EPB41L5,TMEM185B,LOC100060199,LOC100067535,LOC100629454
18	11026066	12399073	1373008	3	1	LOC100052304,LOC100052243,LOC100049893,STEAP3,LOC100629302,DBI,LOC100051693,LOC100051620,LOC100147031,PTPN4,EPB41L5
18	11660478	12345200	684723	3	1	LOC100052304,LOC100052243,LOC100049893,STEAP3,LOC100629302,DBI
18	11660478	12399073	738596	3	1	LOC100052304,LOC100052243,LOC100049893,STEAP3,LOC100629302,DBI
18	23014171	24870435	1856265	1	1	
18	23598943	25534223	1935281	1	1	
18	23848200	25039153	1190954	4	1	
18	32369597	32369919	323	1	1	
18	38240449	38345198	104750	1	4	GALNT5
18	40012486	40342808	330323	1	1	BAZ2B,WDSUB1
18	40012486	40498958	486473	1	1	LY5,MARCH7,BAZ2B,WDSUB1
18	40187377	40498958	311582	1	1	LY5,MARCH7
18	60519874	60601238	81365	1	1	NCKAP1
18	61648648	61806107	157460	1	1	ZNF804A
18	61648648	62193317	544670	1	1	ZNF804A
18	67087964	67160167	72204	1	1	
18	68744107	69289952	545846	1	1	
18	68744107	69669086	924980	1	1	
18	69276563	69343747	67185	3	1	
18	75760478	76129508	369031	3	1	LOC100067910,ORC2,NIF3L1,LOC100067857,CLK1,BZW1,LOC100067956,LOC100067782
18	75813682	76129508	315827	4	1	LOC100067910,ORC2,NIF3L1,LOC100067857,CLK1,BZW1,LOC100067956,LOC100067782
18	81986880	82017901	31022	1	1	
19	1326805	1396027	69223	3	1	
19	1346278	1396027	49750	3	2	
19	7254565	7606478	351914	1	1	
19	13011448	13196606	185159	1	1	LOC100057967
19	15072254	15248736	176483	0	1	NAALADL2
19	16177301	16177592	292	1	1	
19	32598913	32600181	1269	0,1	16,8	DLG1
19	32598913	32621031	22119	1	2	DLG1
19	51656828	51924633	267806	1	2	
19	54585286	54622173	36888	1	1	LOC100072249,ABI3BP
20	27710724	27865962	155239	3	1	LOC100058980,LOC100059020,LOC100059058,LOC100146689,LOC100059132,LOC100059179,LOC100059213,LOC100059255,LOC100059324,LOC100059362,LOC100059400
20	27984017	28089836	105820	1	2	LOC100058467,LOC100058512,LOC100058550,LOC100058588,LOC100058631,LOC100058670

Chrom	Start	End	Size	Cn	Samples (n)	Genes
20	27984017	28206968	222952	1	3	LOC100058118,LOC100058164,LOC100058205,LOC100058247,LOC100058298,LOC100147391,LOC100058338,LOC100058379,LOC100058467,LOC100058512,LOC100058550,LOC100058588,LOC100058631,LOC100058670
20	30344473	30812222	467750	1	2	LOC100053668,LOC100053764,LOC100053918,LOC100050473,LOC100050550,LOC100054114,LOC100054254,LOC100054303,LOC100054397,LOC100629613,LOC100050626
20	31931706	32182629	250924	1	1	LOC100059844,LOC100059681,LOC100059644
20	31961012	32210308	249297	3	1	LOC100059844,LOC100059681,LOC100059644
20	31961093	32210308	249216	1	1	LOC100059844,LOC100059681,LOC100059644
20	32059082	32210308	151227	0,1	1,2	LOC100059844,LOC100059681
20	32127612	32210308	82697	0	10	
20	32127869	32210308	82440	0,1,3	4,1,1	
20	32131071	32210308	79238	0,3	5,1	
20	33534788	34689953	1155166	4	1	LOC100062360,LOC100629770,LOC100629238,LOC100061879,MLN,LOC100061821,IP6K3,LOC100146183,ITPR3,LOC100061784,LOC100061755,ZBTB9,SYNGAP1,LOC100061649,PHF1,KIFC1,LOC100146901,ZBTB22,TAPBP,LOC100052864,LOC100052815,WDR46,LOC100052704,LOC100052654,VPS52,LOC100061478,LOC100061452,MIR219,LOC100061414,LOC100061379,LOC100061303,COL11A2,LOC100061127,LOC100052542
20	33944043	34396948	452906	3	1	LOC100061879,MLN,LOC100061821,IP6K3,LOC100146183,ITPR3
20	34019567	34689953	670387	3	1	LOC100062360,LOC100629770,LOC100629238,LOC100061879,MLN,LOC100061821,IP6K3,LOC100146183
20	34255597	34396948	141352	3	2	LOC100061879
20	44371944	44399130	27187	1	1	
20	44390007	44434681	44675	1	1	
20	45342945	45343265	321	0	1	
21	976423	3708096	2731674	3	1	LOC100147158,LOC100069669,LOC100069652,LOC100071132,LOC100071129,TMEM161A,LOC100069638,ARMC6,SUGP2,LOC100146769,DDX49,COPE,LOC100069594,LOC100071079,UPF1,COMP,LOC100071052,KLHL26,LOC100146655,LOC100146959,LOC100071014,UBA52,LOC100070992,FKBP8,ELL,LOC100147538,LOC100070963,LOC100070946,LOC100070928,LOC100070916,LOC100070899,LOC100069542,LOC100070886,LOC100070878,LOC100146348,LOC100146664,LOC100146965,LOC100070547,LOC100070531,LOC100146857,LOC100146861,LOC100147159,JAK3,INSL3,LOC100146466,LOC100146770,LOC100070399,LOC100146356,GLT25D1,LOC100147252,LOC100070210,LOC100070188,LOC100070181,LOC100630452,LOC100070171,LOC100146856,LOC100147156,GTPBP3,LOC100146761,LOC100630400,LOC100630373,ABHD8,ANKLE1,LOC100069531,USHBP1,LOC100070036,LOC100070019,LOC100070008,MYO9B,LOC100069981,LOC100069965,LOC100069950,SIN3B,LOC100069920,LOC100069904,LOC100069519,LOC100069894,LOC100069507,CHERP,LOC100069877,LOC100069862,EPS15L1,AP1M1,LOC100069816,LOC100630026,LOC100069789,LOC100147248,LOC100147533,LOC100069781,LOC100069770,LOC100069758,LOC100069747,LOC100069709,LOC100070775,LOC100070768,LOC100070761,LOC100070750,LOC100070172,LOC100070159,LOC100070143
21	2164675	3969240	1804566	4	1	LOC100629525,CILP2,LOC100071173,LOC100146253,LOC100146574,GATAD2A,MAU2,LOC100071143,TM6SF2,LOC100146860,LOC100147158,LOC100069669,LOC100069652,LOC100071132,LOC100071129,TMEM161A,LOC100069638,ARMC6,SUGP2,LOC100146769,DDX49,COPE,LOC100069594,LOC100071079,UPF1,COMP,LOC100071052,KLHL26,LOC100146655,LOC100146959,LOC100071014,UBA52,LOC100070992,FKBP8,ELL,LOC100147538,LOC100070963,LOC100070946,LOC100070928,LOC100070916,LOC100070899,LOC100069542,LOC100070886,LOC100070878,LOC100146348,LOC100146664,LOC100146965,LOC100070547,LOC100070531,LOC100146857,LOC100146861,LOC100147159,JAK3,INSL3,LOC100146466,LOC100146770,LOC100070399,LOC100146356,GLT25D1,LOC100147252,LOC100070210,LOC100070188,LOC100070181,LOC100630452,LOC100070171,LOC100146856,LOC100147156,GTPBP3,LOC100146761,LOC100630400,LOC100630373,ABHD8,ANKLE1,LOC100069531,USHBP1,LOC100070036,LOC100070019,LOC100070008,MYO9B
21	2164675	4011553	1846879	4	1	GMIP,LOC100071198,LOC100629525,CILP2,LOC100071173,LOC100146253,LOC100146574,GATAD2A,MAU2,LOC100071143,TM6SF2,LOC100146860,LOC100147158,LOC100069669,LOC100069652,LOC100071132,LOC100071129,TMEM161A,LOC100069638,ARMC6,SUGP2,LOC100146769,DDX49,COPE,LOC100069594,LOC100071079,UPF1,COMP,LOC100071052,KLHL26,LOC100146655,LOC100146959,LOC100071014,UBA52,LOC100070992,FKBP8,ELL,LOC100147538,LOC100070963,LOC100070946,LOC100070928,LOC100070916,LOC100070899,LOC100069542,LOC100070886,LOC100070878,LOC100146348,LOC100146664,LOC100146965,LOC100070547,LOC100070531,LOC100146857,LOC100146861,LOC100147159,JAK3,INSL3,LOC100146466,LOC100146770,LOC100070399,LOC100146356,GLT25D1,LOC100147252,LOC100070210,LOC100070188,LOC100070181,LOC100630452,LOC100070171,LOC100146856,LOC100147156,GTPBP3,LOC100146761,LOC100630400,LOC100630373,ABHD8,ANKLE1,LOC100069531,USHBP1,LOC100070036,LOC100070019,LOC100070008,MYO9B
21	2348717	4836867	2488151	3	1	CDK7,CCDC125,LOC100055662,LOC100055620,LOC100055538,LOC100055246,TRNAE-CUC,TRNAL-CAA,LOC100147062,LOC100055150,SH3BP5L,LOC100054975,LOC100054929,LOC100054881,ATP13A1,GMIP,LOC100071198,LOC100629525,CILP2,LOC100071173,LOC100146253,LOC100146574,GATAD2A,MAU2,LOC100071143,TM6SF2,LOC100146860,LOC100147158,LOC100069669,LOC100069652,LOC100071132,LOC100071129,TMEM161A,LOC100069638,ARMC6,SUGP2,LOC100146769,DDX49,COPE,LOC100069594,LOC100071079,UPF1,COMP,LOC100071052,KLHL26,LOC100146655,LOC100146959,LOC100071014,UBA52,LOC100070992,FKBP8,ELL,LOC100147538,LOC100070963,LOC100070946,LOC100070928,LOC100070916,LOC100070899,LOC100069542,LOC100070886,LOC100070878,LOC100146348,LOC100146664,LOC100146965,LOC100070547,LOC100070531,LOC100146857,LOC100146861,LOC100147159,JAK3,INSL3,LOC100146466,LOC100146770,LOC100070399,LOC100146356,GLT25D1,LOC100147252,LOC100070210,LOC100070188,LOC100070181,LOC100630452,LOC100070171,LOC100146856
21	2851979	2880928	28950	1	1	LOC100070878,LOC100146348,LOC100146664

Chrom	Start	End	Size	Cn	Samples (n)	Genes
21	2851979	3320212	468234	3	1	LOC100071052,KLHL26,LOC100146655,LOC100146959,LOC100071014,UBA52,LOC100070992,FKBP8,ELL,LOC100147538,LOC100070963,LOC100070946,LOC100070928,LOC100070916,LOC100070899,LOC100069542,LOC100070886,LOC100070878,LOC100146348,LOC100146664
21	2851979	4011553	1159575	3	1	GMIP,LOC100071198,LOC100629525,CILP2,LOC100071173,LOC100146253,LOC100146574,GATAD2A,MAU2,LOC100071143,TM6SF2,LOC100146860,LOC100147158,LOC100069669,LOC100069652,LOC100071132,LOC100071129,TMEM161A,LOC100069638,ARMC6,SUGP2,LOC100146769,DDX49,COPE,LOC100069594,LOC100071079,UPF1,COMP,LOC100071052,KLHL26,LOC100146655,LOC100146959,LOC100071014,UBA52,LOC100070992,FKBP8,ELL,LOC100147538,LOC100070963,LOC100070946,LOC100070928,LOC100070916,LOC100070899,LOC100069542,LOC100070886,LOC100070878,LOC100146348,LOC100146664
21	3650019	4079493	429475	3	1	ATP13A1,GMIP,LOC100071198,LOC100629525,CILP2,LOC100071173,LOC100146253,LOC100146574,GATAD2A,MAU2,LOC100071143,TM6SF2,LOC100146860,LOC100147158,LOC100069669,LOC100069652,LOC100071132,LOC100071129
21	3872145	5030403	1158259	3	1	SLC30A5,LOC100050093,LOC100055887,LOC100050028,CDK7,CCDC125,LOC100055662,LOC100055620,LOC100055538,LOC100055246,TRNAE-CUC,TRNAL-CAA,LOC100147062,LOC100055150,SH3BP5L,LOC100054975,LOC100054929,LOC100054881,ATP13A1,GMIP,LOC100071198,LOC100629525,CILP2,LOC100071173,LOC100146253,LOC100146574,GATAD2A
21	8879458	9232014	352557	1	1	RNF180,LOC100057970
21	15306038	15309477	3440	1	1	
21	15306119	15309477	3359	1	1	
21	18811395	18814925	3531	3	1	
21	27888900	28335214	446315	1	1	NIPBL,LOC100053501,LOC100053450,NUP155,WDR70
21	34276138	34691653	415516	1	1	
21	34531228	34691653	160426	1	1	
21	34531228	34926026	394799	1	1	LOC100069125
21	34531228	35344088	812861	1	1	LOC100069241,LOC100069226,LOC100629987,LOC100069125
21	34892949	35809631	916683	1	1	LOC100069309,LOC100069241,LOC100069226,LOC100629987
21	35167053	36758243	1591191	1	1	LOC100054286,LOC100069309,LOC100069241,LOC100069226
21	35205768	35230144	24377	1	1	LOC100069241
21	39789736	41466762	1677027	1	1	UBXD4,CDH12
21	56095987	56476421	380435	3	1	LOC100071885,LOC100071879,IRX4
21	56113660	56476421	362762	3	1	LOC100071885,LOC100071879,IRX4
21	56204564	57016411	811848	3	1	LOC100057994,LOC100058040,LOC100630695,CLPTM1L,SLC6A3,LOC100071898,LOC100071885,LOC100071879,IRX4
21	56242599	56246603	4005	1	1	
21	56242599	57654847	1412249	3	1	LOC100057474,LRRC14B,LOC100057272,SDHA,LOC100057312,LOC100057633,EXOC3,SLC9A3,CEP72,LOC100146662,LOC100057809,BRD9,TRIP13,LOC100057994,LOC100058040,LOC100630695,CLPTM1L,SLC6A3,LOC100071898,LOC100071885,LOC100071879,IRX4
22	13811557	14025949	214393	1	1	PAK7,ANKRD5
22	20037733	23910138	3872406	3	1	BPIFA2,LOC100068814,LOC100068796,BPIL3,BPIL1,LOC100068745,LOC100053947,DNMT3B,LOC100068709,LOC100068694,ASXL1,KIF3B,LOC100630708,LOC100068638,PLAGL2,LOC100068606,LOC100068573,LOC100068557,LOC100147204,LOC100068534,LOC100068515,TTLL9,LOC100146918,LOC100147208,LOC100068461,MYLK2,TPX2,LOC100053597,LOC100053548,LOC100068419,HM13,LOC100053451,LOC100629950,LOC100630303,LOC100629933,LOC100068353,LOC100629884,LOC100629858,LOC100068223,LOC100068192,LOC100629800,LOC100068135,LOC100630230,LOC100068113,LOC100068090,LOC100053406,LOC100053353,LOC100067987,TBC1D20,LOC100067919,LOC100067865,LOC100067845,LOC100053310,LOC100067818,RSPO4,LOC100053261,LOC100067734,LOC100629580,RAD21L,SNPH,LOC100053209,LOC100629541,LOC100067632,LOC100146829,LOC100067609,LOC100067561,LOC100067519,LOC100067431,LOC100053159,LOC100067361,SIRPA,LOC100053108,LOC100067316,TGM3,TGM6,LOC100053021,LOC100629339,TMC2,NOP56,LOC100052969,LOC100067031
22	20704407	22122752	1418346	3	1	LOC100068113,LOC100068090,LOC100053406,LOC100053353,LOC100067987,TBC1D20,LOC100067919,LOC100067865,LOC100067845,LOC100053310,LOC100067818,RSPO4,LOC100053261,LOC100067734,LOC100629580,RAD21L,SNPH,LOC100053209,LOC100629541,LOC100067632,LOC100146829,LOC100067609,LOC100067561,LOC100067519,LOC100067431,LOC100053159,LOC100067361,SIRPA
22	22713848	24107087	1393240	4	1	LOC100068919,LATH,BPIFA2,LOC100068814,LOC100068796,BPIL3,BPIL1,LOC100068745,LOC100053947,DNMT3B,LOC100068709,LOC100068694,ASXL1,KIF3B,LOC100630708,LOC100068638,PLAGL2,LOC100068606,LOC100068573,LOC100068557,LOC100147204,LOC100068534,LOC100068515,TTLL9,LOC100146918,LOC100147208,LOC100068461,MYLK2,TPX2,LOC100053597
22	28194027	28451955	257929	3	1	LOC100070061,LOC100070046,RPRD1B,TTI1,LOC100069991
22	28439582	28451955	12374	3,4	1,1	LOC100070061,LOC100070046
22	28439582	28652598	213017	4	1	LOC100070140,LBP,BPI,LOC100070061,LOC100070046
22	28439582	28931459	491878	3	1	LOC100070271,LOC100146532,LOC100070248,LOC100630181,RALGAPB,LOC100070140,LBP,BPI,LOC100070061,LOC100070046

Chrom	Start	End	Size	Cn	Samples (n)	Genes
22	33098209	37835325	4737117	3	1	PTGIS,KCNB1,LOC100630752,LOC100630714,LOC100071401,ZNFX1,DDX27,STAU1,CSE1L,ARFGEF2,LOC100071328,SULF2,NCOA3,LOC100056807,EYA2,SLC2A10,LOC100071235,SLC13A3,LOC100630513,LOC100071217,LOC100630472,ELMO2,SLC35C2,LOC100146724,CD40,NCOA5,SLC12A5,MMP9,ZNF335,PCIF1,PLTP,CTSA,LOC100071119,LOC100056463,ZSWIM1,ZSWIM3,ACOT8,SNX21,LOC100056292,UBE2C,DNTTIP1,LOC100071084,LOC100071077,LOC100630249,LOC100630229,LOC100629250,LOC100630200,LOC100071021,LOC100630144,LOC100056252,LOC100056211,PIGT,LOC100070968,LOC100070960,LOC100070943,LOC100070934,RBPJL,MATN4,LOC100070893,LOC100056125,LOC100070884,LOC100070875,STK4,LOC100070847,PABPC1L,LOC100056084,LOC100070800,LOC100070779,ADA,LOC100070766,LOC100070755,LOC100070746,TTPAL,HNF4A,LOC100147026,LOC100147308,LOC100055926,LOC100070723,LOC100146318,TOX2,LOC100070703,MYBL2
22	35996923	39710346	3713424	3	1	SALL4,ATP9A,NFATC2,KCNG1,LOC100052426,LOC100052367,ADNP,LOC100052252,PARD6B,LOC100049830,PTPN1,LOC100071474,TMEM189,LOC100071451,LOC100071447,SPATA2,LOC100071430,SLC9A8,LOC100071419,PTGIS,KCNB1,LOC100630752,LOC100630714,LOC100071401,ZNFX1,DDX27,STAU1,CSE1L,ARFGEF2,LOC100071328,SULF2,NCOA3,LOC100056807
22	36384241	36545238	160998	3	1	
22	36384241	37791711	1407471	3,4	1,1	PTGIS,KCNB1,LOC100630752,LOC100630714,LOC100071401,ZNFX1,DDX27,STAU1,CSE1L,ARFGEF2,LOC100071328
22	36384241	38313191	1928951	3	1	LOC100071474,TMEM189,LOC100071451,LOC100071447,SPATA2,LOC100071430,SLC9A8,LOC100071419,PTGIS,KCNB1,LOC100630752,LOC100630714,LOC100071401,ZNFX1,DDX27,STAU1,CSE1L,ARFGEF2,LOC100071328
22	37095542	39038452	1942911	3	1	KCNG1,LOC100052426,LOC100052367,ADNP,LOC100052252,PARD6B,LOC100049830,PTPN1,LOC100071474,TMEM189,LOC100071451,LOC100071447,SPATA2,LOC100071430,SLC9A8,LOC100071419,PTGIS,KCNB1,LOC100630752,LOC100630714,LOC100071401,ZNFX1,DDX27,STAU1,CSE1L,ARFGEF2,LOC100071328
22	45694662	45749595	54934	1	2	EDN3
22	47060122	49647501	2587380	3	1	LOC100051629,TPD52L2,LOC100060886,LOC100051559,LOC100060846,LOC100060807,LOC100060772,LOC100629202,LOC100146495,LOC100060731,LOC100060664,GMEB2,LOC100060598,LOC100146686,SRMS,PTK6,LOC100630715,EEF1A2,KCNQ2,LOC100147373,YTHDF1,LOC100058964,LOC100051346,DIDO1,LOC100058698,LOC100058660,LOC100146483,LOC100058495,NTSR1,SLCO4A1,MIR133A-2,MIR1-2,LOC100058278,LOC100058190,LOC100058145,LOC100630744,LAMA5,LOC100051201,OSBPL2,LOC100058016,LOC100057922,LOC100057871,LOC100057828,LOC100057778,TAF4,LOC100057648
22	47374927	49015861	1640935	3	1	YTHDF1,LOC100058964,LOC100051346,DIDO1,LOC100058698,LOC100058660,LOC100146483,LOC100058495,NTSR1,SLCO4A1,MIR133A-2,MIR1-2,LOC100058278,LOC100058190,LOC100058145,LOC100630744,LAMA5,LOC100051201,OSBPL2,LOC100058016,LOC100057922,LOC100057871,LOC100057828,LOC100057778,TAF4,LOC100057648
22	47580770	48819937	1239168	3	1	LOC100058964,LOC100051346,DIDO1,LOC100058698,LOC100058660,LOC100146483,LOC100058495,NTSR1,SLCO4A1,MIR133A-2,MIR1-2,LOC100058278,LOC100058190,LOC100058145,LOC100630744,LAMA5,LOC100051201,OSBPL2,LOC100058016,LOC100057922,LOC100057871,LOC100057828,LOC100057778,TAF4,LOC100057648
22	47580770	49358382	1777613	3	1	GMEB2,LOC100060598,LOC100146686,SRMS,PTK6,LOC100630715,EEF1A2,KCNQ2,LOC100147373,YTHDF1,LOC100058964,LOC100051346,DIDO1,LOC100058698,LOC100058660,LOC100146483,LOC100058495,NTSR1,SLCO4A1,MIR133A-2,MIR1-2,LOC100058278,LOC100058190,LOC100058145,LOC100630744,LAMA5,LOC100051201,OSBPL2,LOC100058016,LOC100057922,LOC100057871,LOC100057828,LOC100057778,TAF4,LOC100057648
22	47580770	49761753	2180984	3	1	LOC100146980,LOC100061164,LOC100147562,LOC100146261,LOC100051629,TPD52L2,LOC100060886,LOC100051559,LOC100060846,LOC100060807,LOC100060772,LOC100629202,LOC100146495,LOC100060731,LOC100060664,GMEB2,LOC100060598,LOC100146686,SRMS,PTK6,LOC100630715,EEF1A2,KCNQ2,LOC100147373,YTHDF1,LOC100058964,LOC100051346,DIDO1,LOC100058698,LOC100058660,LOC100146483,LOC100058495,NTSR1,SLCO4A1,MIR133A-2,MIR1-2,LOC100058278,LOC100058190,LOC100058145,LOC100630744,LAMA5,LOC100051201,OSBPL2,LOC100058016,LOC100057922,LOC100057871,LOC100057828,LOC100057778,TAF4,LOC100057648
22	48037042	49944830	1907789	4	1	LOC100051947,MYT1,LOC100051823,LOC100051762,LOC100146679,LOC100051702,LOC100146980,LOC100061164,LOC100147562,LOC100146261,LOC100051629,TPD52L2,LOC100060886,LOC100051559,LOC100060846,LOC100060807,LOC100060772,LOC100629202,LOC100146495,LOC100060731,LOC100060664,GMEB2,LOC100060598,LOC100146686,SRMS,PTK6,LOC100630715,EEF1A2,KCNQ2,LOC100147373,YTHDF1,LOC100058964,LOC100051346,DIDO1,LOC100058698,LOC100058660,LOC100146483,LOC100058495,NTSR1,SLCO4A1,MIR133A-2,MIR1-2,LOC100058278,LOC100058190,LOC100058145,LOC100630744,LAMA5,LOC100051201,OSBPL2,LOC100058016,LOC100057922,LOC100057871,LOC100057828,LOC100057778
22	48819937	49761753	941817	1	1	LOC100146980,LOC100061164,LOC100147562,LOC100146261,LOC100051629,TPD52L2,LOC100060886,LOC100051559,LOC100060846,LOC100060807,LOC100060772,LOC100629202,LOC100146495,LOC100060731,LOC100060664,GMEB2,LOC100060598,LOC100146686,SRMS,PTK6,LOC100630715,EEF1A2,KCNQ2,LOC100147373,YTHDF1
23	41804	264863	223060	3,4	1,1	LOC100146373,LOC100049855,LOC100629283
23	7421748	8512483	1090736	3	1	LOC100054022,LOC100055310,LOC100055131,LOC100055085,LOC100054860,LOC100054543,LOC100054500,LOC100054455,LOC100054357,LOC100054171,LOC100054122,SLC28A3,LOC100055132,SLC28A3
23	16945569	17031995	86427	3	1	
23	17021853	17100602	78750	1	3	
23	29970005	30230205	260201	1	1	PTPRD
23	29970005	30719694	749690	3	1	PTPRD

Chrom	Start	End	Size	Cn	Samples (n)	Genes
23	31505334	32671156	1165823	1	1	LOC100629545,LOC100629526
23	31505334	32741939	1236606	1	1	LOC100629545,LOC100629526
23	31628999	31843421	214423	1	1	LOC100629526
23	31628999	32741939	1112941	1	1	LOC100629545,LOC100629526
23	32216945	32671156	454212	1	1	LOC100629545
23	35814091	35987901	173811	4	1	
23	42919317	43408006	488690	1	2	LOC100630134
23	47780596	48104343	323748	1	1	LOC100067680
23	52460740	53311605	850866	3	1	LOC100061893,SHC3,LOC100630890,SECISBP2,LOC100062234,LOC100054996
23	53853729	54980902	1127174	1	1	LOC100056145,LOC100146174,LOC100056530,LOC100146796,LOC100147073,LOC100057755,LOC100058211,LOC100629266,PTPDC1,MIRLET7F,MIRLET7D,LOC100059859,ZNF169,LOC100060076,LOC100060234,LOC100060268
23	53980697	54021152	40456	1	1	LOC100060076
23	54295300	55235809	940510	3	1	ECM2,LOC100054857,IPPK,BICD2,LOC100056145,LOC100146174,LOC100056530,LOC100146796,LOC100147073,LOC100057755,LOC100058211,LOC100629266
23	54295300	55698274	1402975	3	1	LOC100055223,ZNF484,IARS,LOC100055560,LOC100054772,ASPN,ECM2,LOC100054857,IPPK,BICD2,LOC100056145,LOC100146174,LOC100056530,LOC100146796,LOC100147073,LOC100057755,LOC100058211,LOC100629266
23	54359138	55194983	835846	3,4	1,1	IPPK,BICD2,LOC100056145,LOC100146174,LOC100056530,LOC100146796,LOC100147073,LOC100057755,LOC100058211
23	54359138	55235809	876672	3	1	ECM2,LOC100054857,IPPK,BICD2,LOC100056145,LOC100146174,LOC100056530,LOC100146796,LOC100147073,LOC100057755,LOC100058211
23	54466079	55235809	769731	3	1	ECM2,LOC100054857,IPPK,BICD2,LOC100056145,LOC100146174,LOC100056530,LOC100146796,LOC100147073,LOC100057755,LOC100058211
23	54575178	55009991	434814	1	1	LOC100056145,LOC100146174,LOC100056530,LOC100146796,LOC100147073,LOC100057755
23	54759475	55194983	435509	4	1	IPPK,BICD2,LOC100056145,LOC100146174,LOC100056530,LOC100146796,LOC100147073
23	54759475	55232008	472534	4	1	LOC100054857,IPPK,BICD2,LOC100056145,LOC100146174,LOC100056530,LOC100146796,LOC100147073
23	54865842	55194983	329142	4	1	IPPK,BICD2,LOC100056145,LOC100146174,LOC100056530,LOC100146796,LOC100147073
23	54962623	55698274	735652	3	1	LOC100055223,ZNF484,IARS,LOC100055560,LOC100054772,ASPN,ECM2,LOC100054857,IPPK,BICD2,LOC100056145
23	55094544	55232008	137465	4	1	LOC100054857,IPPK,BICD2
24	16894423	16990120	95698	4	1	MAP3K9
24	32416012	32508738	92727	3	3	TTC8,EML5
24	32416012	32628728	212717	3	2	TTC8,EML5
24	38319415	38375555	56141	3	1	
24	38370785	38375555	4771	3	1	
24	42379627	42672141	292515	3	1	DLK1,LOC100146339,WDR25
24	44688141	44978135	289995	1	1	MARK3,EIF5,LOC100059242,LOC100146644
24	45592333	45831667	239335	1	1	
24	46407316	46707155	299840	1	1	LOC100147439,LOC100064522,ZFP36L1,LOC100063626
25	8051850	9362252	1310403	1	1	LOC100062681,LOC100062753,GRIN3A
25	26318531	26560311	241781	1	1	LOC100071413,LOC100071420,LOC100146823,LOC100071438,LOC100071445,LOC100071448,LOC100071452,LOC100071460,LOC100071469,LOC100071479,LOC100071486,LOC100071488,LOC100071492,LOC100071496,LOC100071502
25	26318531	26599435	280905	1	1	LOC100071402,LOC100071407,LOC100071413,LOC100071420,LOC100146823,LOC100071438,LOC100071445,LOC100071448,LOC100071452,LOC100071460,LOC100071469,LOC100071479,LOC100071486,LOC100071488,LOC100071492,LOC100071496,LOC100071502
25	26318531	26647861	329331	1	1	LOC100071352,LOC100071358,LOC100071365,LOC100071376,LOC100071382,LOC100071392,LOC100071402,LOC100071407,LOC100071413,LOC100071420,LOC100146823,LOC100071438,LOC100071445,LOC100071448,LOC100071452,LOC100071460,LOC100071469,LOC100071479,LOC100071486,LOC100071488,LOC100071492,LOC100071496,LOC100071502
25	26318531	26918263	599733	1	3	LOC100071251,LOC100067520,LOC100071258,LOC100071264,LOC100071270,LOC100071275,LOC100071278,LOC100071283,LOC100071287,LOC100071297,LOC100146817,LOC100071311,LOC100071317,LOC100147676,LOC100071322,LOC100071329,LOC100071332,LOC100071338,LOC100071346,LOC100071352,LOC100071358,LOC100071365,LOC100071376,LOC100071382,LOC100071392,LOC100071402,LOC100071407,LOC100071413,LOC100071420,LOC100146823,LOC100071438,LOC100071445,LOC100071448,LOC100071452,LOC100071460,LOC100071469,LOC100071479,LOC100071486,LOC100071488,LOC100071492,LOC100071496,LOC100071502
25	26318531	26942120	623590	1	5	LOC100071244,LOC100071251,LOC100067520,LOC100071258,LOC100071264,LOC100071270,LOC100071275,LOC100071278,LOC100071283,LOC100071287,LOC100071297,LOC100146817,LOC100071311,LOC100071317,LOC100147676,LOC100071322,LOC100071329,LOC100071332,LOC100071338,LOC100071346,LOC100071352,LOC100071358,LOC100071365,LOC100071376,LOC100071382,LOC100071392,LOC100071402,LOC100071407,LOC100071413,LOC100071420,LOC100146823,LOC100071438,LOC100071445,LOC100071448,LOC100071452,LOC100071460,LOC100071469,LOC100071479,LOC100071486,LOC100071488,LOC100071492,LOC100071496,LOC100071502
25	26361000	26512888	151889	1	6	LOC100146823,LOC100071438,LOC100071445,LOC100071448,LOC100071452,LOC100071460,LOC100071469,LOC100071479,LOC100071486
25	26361000	26560311	199312	1	10	LOC100071413,LOC100071420,LOC100146823,LOC100071438,LOC100071445,LOC100071448,LOC100071452,LOC100071460,LOC100071469,LOC100071479,LOC100071486

Chrom	Start	End	Size	Cn	Samples (n)	Genes
25	26361000	26599435	238436	1	1	LOC100071402,LOC100071407,LOC100071413,LOC100071420,LOC100146823,LOC100071438,LOC100071445,LOC100071448,LOC100071452,LOC100071460,LOC100071469,LOC100071479,LOC100071486
25	26361000	26647861	286862	1	3	LOC100071352,LOC100071358,LOC100071365,LOC100071376,LOC100071382,LOC100071392,LOC100071402,LOC100071407,LOC100071413,LOC100071420,LOC100146823,LOC100071438,LOC100071445,LOC100071448,LOC100071452,LOC100071460,LOC100071469,LOC100071479,LOC100071486
25	26361000	26918263	557264	1	7	LOC100071251,LOC100067520,LOC100071258,LOC100071264,LOC100071270,LOC100071275,LOC100071278,LOC100071283,LOC100071287,LOC100071297,LOC100146817,LOC100071311,LOC100071317,LOC100147676,LOC100071322,LOC100071329,LOC100071332,LOC100071338,LOC100071346,LOC100071352,LOC100071358,LOC100071365,LOC100071376,LOC100071382,LOC100071392,LOC100071402,LOC100071407,LOC100071413,LOC100071420,LOC100146823,LOC100071438,LOC100071445,LOC100071448,LOC100071452,LOC100071460,LOC100071469,LOC100071479,LOC100071486
25	26361000	26942120	581121	0,1	14,53	LOC100071244,LOC100071251,LOC100067520,LOC100071258,LOC100071264,LOC100071270,LOC100071275,LOC100071278,LOC100071283,LOC100071287,LOC100071297,LOC100146817,LOC100071311,LOC100071317,LOC100147676,LOC100071322,LOC100071329,LOC100071332,LOC100071338,LOC100071346,LOC100071352,LOC100071358,LOC100071365,LOC100071376,LOC100071382,LOC100071392,LOC100071402,LOC100071407,LOC100071413,LOC100071420,LOC100146823,LOC100071438,LOC100071445,LOC100071448,LOC100071452,LOC100071460,LOC100071469,LOC100071479,LOC100071486
25	26361000	27125754	764755	1	3	RC3H2,LOC100071170,LOC100071180,LOC100067499,LOC100071189,LOC100630709,LOC100071212,LOC100071218,LOC100071227,LOC100071236,LOC100071244,LOC100071251,LOC100067520,LOC100071258,LOC100071264,LOC100071270,LOC100071275,LOC100071278,LOC100071283,LOC100071287,LOC100071297,LOC100146817,LOC100071311,LOC100071317,LOC100147676,LOC100071322,LOC100071329,LOC100071332,LOC100071338,LOC100071346,LOC100071352,LOC100071358,LOC100071365,LOC100071376,LOC100071382,LOC100071392,LOC100071402,LOC100071407,LOC100071413,LOC100071420,LOC100146823,LOC100071438,LOC100071445,LOC100071448,LOC100071452,LOC100071460,LOC100071469,LOC100071479,LOC100071486
25	26393590	26560311	166722	1	2	LOC100071413,LOC100071420,LOC100146823,LOC100071438,LOC100071445,LOC100071448,LOC100071452,LOC100071460,LOC100071469
25	26393590	26647861	254272	1	1	LOC100071352,LOC100071358,LOC100071365,LOC100071376,LOC100071382,LOC100071392,LOC100071402,LOC100071407,LOC100071413,LOC100071420,LOC100146823,LOC100071438,LOC100071445,LOC100071448,LOC100071452,LOC100071460,LOC100071469
25	26393590	26918263	524674	1	2	LOC100071251,LOC100067520,LOC100071258,LOC100071264,LOC100071270,LOC100071275,LOC100071278,LOC100071283,LOC100071287,LOC100071297,LOC100146817,LOC100071311,LOC100071317,LOC100147676,LOC100071322,LOC100071329,LOC100071332,LOC100071338,LOC100071346,LOC100071352,LOC100071358,LOC100071365,LOC100071376,LOC100071382,LOC100071392,LOC100071402,LOC100071407,LOC100071413,LOC100071420,LOC100146823,LOC100071438,LOC100071445,LOC100071448,LOC100071452,LOC100071460,LOC100071469
25	26393590	26942120	548531	1	1	LOC100071244,LOC100071251,LOC100067520,LOC100071258,LOC100071264,LOC100071270,LOC100071275,LOC100071278,LOC100071283,LOC100071287,LOC100071297,LOC100146817,LOC100071311,LOC100071317,LOC100147676,LOC100071322,LOC100071329,LOC100071332,LOC100071338,LOC100071346,LOC100071352,LOC100071358,LOC100071365,LOC100071376,LOC100071382,LOC100071392,LOC100071402,LOC100071407,LOC100071413,LOC100071420,LOC100146823,LOC100071438,LOC100071445,LOC100071448,LOC100071452,LOC100071460,LOC100071469
25	26509315	26599435	90121	1	1	LOC100071402,LOC100071407,LOC100071413,LOC100071420,LOC100146823
25	26509315	26647861	138547	1	3	LOC100071352,LOC100071358,LOC100071365,LOC100071376,LOC100071382,LOC100071392,LOC100071402,LOC100071407,LOC100071413,LOC100071420,LOC100146823
25	26509315	26918263	408949	1	1	LOC100071251,LOC100067520,LOC100071258,LOC100071264,LOC100071270,LOC100071275,LOC100071278,LOC100071283,LOC100071287,LOC100071297,LOC100146817,LOC100071311,LOC100071317,LOC100147676,LOC100071322,LOC100071329,LOC100071332,LOC100071338,LOC100071346,LOC100071352,LOC100071358,LOC100071365,LOC100071376,LOC100071382,LOC100071392,LOC100071402,LOC100071407,LOC100071413,LOC100071420,LOC100146823
25	26509315	26942120	432806	1	10	LOC100071244,LOC100071251,LOC100067520,LOC100071258,LOC100071264,LOC100071270,LOC100071275,LOC100071278,LOC100071283,LOC100071287,LOC100071297,LOC100146817,LOC100071311,LOC100071317,LOC100147676,LOC100071322,LOC100071329,LOC100071332,LOC100071338,LOC100071346,LOC100071352,LOC100071358,LOC100071365,LOC100071376,LOC100071382,LOC100071392,LOC100071402,LOC100071407,LOC100071413,LOC100071420,LOC100146823
25	26560311	26647861	87551	1,3	2,1	LOC100071352,LOC100071358,LOC100071365,LOC100071376,LOC100071382,LOC100071392,LOC100071402,LOC100071407
25	26560311	26866414	306104	0,1	4,3	LOC100071270,LOC100071275,LOC100071278,LOC100071283,LOC100071287,LOC100071297,LOC100146817,LOC100071311,LOC100071317,LOC100147676,LOC100071322,LOC100071329,LOC100071332,LOC100071338,LOC100071346,LOC100071352,LOC100071358,LOC100071365,LOC100071376,LOC100071382,LOC100071392,LOC100071402,LOC100071407
25	26560311	26942120	381810	1	2	LOC100071244,LOC100071251,LOC100067520,LOC100071258,LOC100071264,LOC100071270,LOC100071275,LOC100071278,LOC100071283,LOC100071287,LOC100071297,LOC100146817,LOC100071311,LOC100071317,LOC100147676,LOC100071322,LOC100071329,LOC100071332,LOC100071338,LOC100071346,LOC100071352,LOC100071358,LOC100071365,LOC100071376,LOC100071382,LOC100071392,LOC100071402,LOC100071407
25	26560311	27125754	565444	1	1	RC3H2,LOC100071170,LOC100071180,LOC100067499,LOC100071189,LOC100630709,LOC100071212,LOC100071218,LOC100071227,LOC100071236,LOC100071244,LOC100071251,LOC100067520,LOC100071258,LOC100071264,LOC100071270,LOC100071275,LOC100071278,LOC100071283,LOC100071287,LOC100071297,LOC100146817,LOC100071311,LOC100071317,LOC100147676,LOC100071322,LOC100071329,LOC100071332,LOC100071338,LOC100071346,LOC100071352,LOC100071358,LOC100071365,LOC100071376,LOC100071382,LOC100071392,LOC100071402,LOC100071407
25	26599255	26647861	48607	3	1	LOC100071352,LOC100071358,LOC100071365,LOC100071376,LOC100071382,LOC100071392
25	26599255	26751364	152110	1	1	LOC100146817,LOC100071311,LOC100071317,LOC100147676,LOC100071322,LOC100071329,LOC100071332,LOC100071338,LOC100071346,LOC100071352,LOC100071358,LOC100071365,LOC100071376,LOC100071382,LOC100071392

Chrom	Start	End	Size	Cn	Samples (n)	Genes
25	26599255	26866414	267160	1	1	LOC100071270,LOC100071275,LOC100071278,LOC100071283,LOC100071287,LOC100071297,LOC100146817,LOC100071311,LOC100071317,LOC100147676,LOC100071322,LOC100071329,LOC100071332,LOC100071338,LOC100071346,LOC100071352,LOC100071358,LOC100071365,LOC100071376,LOC100071382,LOC100071392
25	26647861	26751364	103504	3	2	LOC100146817,LOC100071311,LOC100071317,LOC100147676,LOC100071322,LOC100071329,LOC100071332,LOC100071338,LOC100071346
25	26647861	26918263	270403	1	4	LOC100071251,LOC100067520,LOC100071258,LOC100071264,LOC100071270,LOC100071275,LOC100071278,LOC100071283,LOC100071287,LOC100071297,LOC100146817,LOC100071311,LOC100071317,LOC100147676,LOC100071322,LOC100071329,LOC100071332,LOC100071338,LOC100071346
25	26647861	26942120	294260	1	1	LOC100071244,LOC100071251,LOC100067520,LOC100071258,LOC100071264,LOC100071270,LOC100071275,LOC100071278,LOC100071283,LOC100071287,LOC100071297,LOC100146817,LOC100071311,LOC100071317,LOC100147676,LOC100071322,LOC100071329,LOC100071332,LOC100071338,LOC100071346
25	26750874	26751364	491	3	4	
25	26750874	26942120	191247	1	2	LOC100071244,LOC100071251,LOC100067520,LOC100071258,LOC100071264,LOC100071270,LOC100071275,LOC100071278,LOC100071283,LOC100071287,LOC100071297
25	26866414	26918263	51850	1	3	LOC100071251,LOC100067520,LOC100071258,LOC100071264
25	26866414	26942120	75707	1	2	LOC100071244,LOC100071251,LOC100067520,LOC100071258,LOC100071264
25	29618659	29621832	3174	3	6	LOC100070756
25	30948674	31493936	545263	1	1	LOC100067056,LOC100070310,LOC100070332,SLC25A25,LOC100067073,LOC100067099,LOC100070352,PIP5KL1,LOC100070379,LOC100070387,LOC100067121,ST6GALNAC6,LOC100070413,ENG,FPGS,LOC100070437,SH2D3C,LOC100070462,TTC16,LOC100070475,LOC100070483,STXBP1,LOC100070508,LRSAM1
25	33241747	38860003	5618257	4	1	EXD3,LOC100058595,LOC100059522,LOC100059560,LOC100058638,TUBB2C,LOC100147578,LOC100629886,LOC100058677,LOC100058717,LOC100146716,LOC100147202,LOC100059767,LOC100059826,GRIN1,MAN1A2,LOC100059924,MAN1B1,UAP1L1,LOC100067771,LOC100066152,LOC100067846,ABCA2,LOC100066181,LOC100146303,LOC100146610,PTGDS,LOC100147492,FBXW5,TANK,LOC100068036,MAMDC4,LOC100068092,LOC100068114,LOC100629484,LOC100068153,LOC100147579,LOC100068169,LOC100068193,LOC100068210,LGB2,LGB1,LOC100068354,LOC100068369,P19,LOC100068400,LOC100068421,CAMSAP1,LOC100068463,NACC2,LHX3,LOC100068607,LOC100068625,LOC100066325,LOC100629160,CARD9,LOC100066352,SDCCAG3,PMPCA,LOC100068731,SEC16A,NOTCH1,MIR126,LOC100066378,LOC100068797,LOC100068815,LOC100068864,LOC100146707,OLFM1,LOC100069029,LOC100069057,LOC100069096,WDR5,BRD3,LOC100069175,SARDH,DBH,LOC100147292,ADAMTSL2,LOC100147681,ADAMTS13,LOC100066519,LOC100147300,LOC100069310,LOC100146305,LOC100069334,RPL7A,LOC100066547,LOC100066565,LOC100147494,LOC100069353,LOC100069388,LOC100069402,CEL,GTF3C5,LOC100069424,TSC1,LOC100069435,AK8,GTF3C4,DDX31,BARHL1,LOC100069473,LOC100069488,SETX,LOC100069501,LOC100069513,RAPGEF1,LOC100066704,LOC100069565,POMT1,PRRC2B,LOC100069601,LOC100066755,NUP214,LOC100069645,LAMC3,LOC100069687,LOC100069698,ABL1,LOC100069734,LOC100146306,FUBP3,ASS1
25	35440408	37675442	2235035	3	1	LOC100068625,LOC100066325,LOC100629160,CARD9,LOC100066352,SDCCAG3,PMPCA,LOC100068731,SEC16A,NOTCH1,MIR126,LOC100066378,LOC100068797,LOC100068815,LOC100068864,LOC100146707,OLFM1,LOC100069029,LOC100069057,LOC100069096,WDR5,BRD3,LOC100069175,SARDH,DBH,LOC100147292,ADAMTSL2,LOC100147681,ADAMTS13,LOC100066519,LOC100147300,LOC100069310,LOC100146305,LOC100069334,RPL7A,LOC100066547,LOC100066565,LOC100147494,LOC100069353
25	35440408	37929574	2489167	3	1	NACC2,LHX3,LOC100068607,LOC100068625,LOC100066325,LOC100629160,CARD9,LOC100066352,SDCCAG3,PMPCA,LOC100068731,SEC16A,NOTCH1,MIR126,LOC100066378,LOC100068797,LOC100068815,LOC100068864,LOC100146707,OLFM1,LOC100069029,LOC100069057,LOC100069096,WDR5,BRD3,LOC100069175,SARDH,DBH,LOC100147292,ADAMTSL2,LOC100147681,ADAMTS13,LOC100066519,LOC100147300,LOC100069310,LOC100146305,LOC100069334,RPL7A,LOC100066547,LOC100066565,LOC100147494,LOC100069353
25	35440408	38860003	3419596	3	1	EXD3,LOC100058595,LOC100059522,LOC100059560,LOC100058638,TUBB2C,LOC100147578,LOC100629886,LOC100058677,LOC100058717,LOC100146716,LOC100147202,LOC100059767,LOC100059826,GRIN1,MAN1A2,LOC100059924,MAN1B1,UAP1L1,LOC100067771,LOC100066152,LOC100067846,ABCA2,LOC100066181,LOC100146303,LOC100146610,PTGDS,LOC100147492,FBXW5,TANK,LOC100068036,MAMDC4,LOC100068092,LOC100068114,LOC100629484,LOC100068153,LOC100147579,LOC100068169,LOC100068193,LOC100068210,LGB2,LGB1,LOC100068354,LOC100068369,P19,LOC100068400,LOC100068421,CAMSAP1,LOC100068463,NACC2,LHX3,LOC100068607,LOC100068625,LOC100066325,LOC100629160,CARD9,LOC100066352,SDCCAG3,PMPCA,LOC100068731,SEC16A,NOTCH1,MIR126,LOC100066378,LOC100068797,LOC100068815,LOC100068864,LOC100146707,OLFM1,LOC100069029,LOC100069057,LOC100069096,WDR5,BRD3,LOC100069175,SARDH,DBH,LOC100147292,ADAMTSL2,LOC100147681,ADAMTS13,LOC100066519,LOC100147300,LOC100069310,LOC100146305,LOC100069334,RPL7A,LOC100066547,LOC100066565,LOC100147494,LOC100069353
25	35440408	39003009	3562602	3	1	PNPLA7,LOC100147094,ENTPD8,LOC100059408,EXD3,LOC100058595,LOC100059522,LOC100059560,LOC100058638,TUBB2C,LOC100147578,LOC100629886,LOC100058677,LOC100058717,LOC100146716,LOC100147202,LOC100059767,LOC100059826,GRIN1,MAN1A2,LOC100059924,MAN1B1,UAP1L1,LOC100067771,LOC100066152,LOC100067846,ABCA2,LOC100066181,LOC100146303,LOC100146610,PTGDS,LOC100147492,FBXW5,TANK,LOC100068036,MAMDC4,LOC100068092,LOC100068114,LOC100629484,LOC100068153,LOC100147579,LOC100068169,LOC100068193,LOC100068210,LGB2,LGB1,LOC100068354,LOC100068369,P19,LOC100068400,LOC100068421,CAMSAP1,LOC100068463,NACC2,LHX3,LOC100068607,LOC100068625,LOC100066325,LOC100629160,CARD9,LOC100066352,SDCCAG3,PMPCA,LOC100068731,SEC16A,NOTCH1,MIR126,LOC100066378,LOC100068797,LOC100068815,LOC100068864,LOC100146707,OLFM1,LOC100069029,LOC100069057,LOC100069096,WDR5,BRD3,LOC100069175,SARDH,DBH,LOC100147292,ADAMTSL2,LOC100147681,ADAMTS13,LOC100066519,LOC100147300,LOC100069310,LOC100146305,LOC100069334,RPL7A,LOC100066547,LOC100066565,LOC100147494,LOC100069353

Chrom	Start	End	Size	Cn	Samples (n)	Genes
25	35440408	39026923	3586516	3	1	PNPLA7,LOC100147094,ENTPD8,LOC100059408,EXD3,LOC100058595,LOC100059522,LOC100059560,LOC100058638,TUBB2C,LOC100147578,LOC100629886,LOC100058677,LOC100058717,LOC100146716,LOC100147202,LOC100059767,LOC100059826,GRIN1,MAN1A2,LOC100059924,MAN1B1,UAP1L1,LOC100067771,LOC100066152,LOC100067846,ABCA2,LOC100066181,LOC100146303,LOC100146610,PTGDS,LOC100147492,FBXW5,TANK,LOC100068036,MAMDC4,LOC100068092,LOC100068114,LOC100629484,LOC100068153,LOC100147579,LOC100068169,LOC100068193,LOC100068210,LGB2,LGB1,LOC100068354,LOC100068369,P19,LOC100068400,LOC100068421,CAMSAP1,LOC100068463,NACC2,LHX3,LOC100068607,LOC100068625,LOC100066325,LOC100629160,CARD9,LOC100066352,SDCCAG3,PMPCA,LOC100068731,SEC16A,NOTCH1,MIR126,LOC100066378,LOC100068797,LOC100068815,LOC100068864,LOC100146707,OLFM1,LOC100069029,LOC100069057,LOC100069096,WDR5,BRD3,LOC100069175,SARDH,DBH,LOC100147292,ADAMTSL2,LOC100147681,ADAMTS13,LOC100066519,LOC100147300,LOC100069310,LOC100146305,LOC100069334,RPL7A,LOC100066547,LOC100066565,LOC100147494,LOC100069353
25	35440408	39090329	3649922	3	1	LOC100059140,LOC100147491,PNPLA7,LOC100147094,ENTPD8,LOC100059408,EXD3,LOC100058595,LOC100059522,LOC100059560,LOC100058638,TUBB2C,LOC100147578,LOC100629886,LOC100058677,LOC100058717,LOC100146716,LOC100147202,LOC100059767,LOC100059826,GRIN1,MAN1A2,LOC100059924,MAN1B1,UAP1L1,LOC100067771,LOC100066152,LOC100067846,ABCA2,LOC100066181,LOC100146303,LOC100146610,PTGDS,LOC100147492,FBXW5,TANK,LOC100068036,MAMDC4,LOC100068092,LOC100068114,LOC100629484,LOC100068153,LOC100147579,LOC100068169,LOC100068193,LOC100068210,LGB2,LGB1,LOC100068354,LOC100068369,P19,LOC100068400,LOC100068421,CAMSAP1,LOC100068463,NACC2,LHX3,LOC100068607,LOC100068625,LOC100066325,LOC100629160,CARD9,LOC100066352,SDCCAG3,PMPCA,LOC100068731,SEC16A,NOTCH1,MIR126,LOC100066378,LOC100068797,LOC100068815,LOC100068864,LOC100146707,OLFM1,LOC100069029,LOC100069057,LOC100069096,WDR5,BRD3,LOC100069175,SARDH,DBH,LOC100147292,ADAMTSL2,LOC100147681,ADAMTS13,LOC100066519,LOC100147300,LOC100069310,LOC100146305,LOC100069334,RPL7A,LOC100066547,LOC100066565,LOC100147494,LOC100069353
25	36110418	36869793	759376	3	2	LOC100069029,LOC100069057,LOC100069096,WDR5,BRD3
25	36110418	37067512	957095	3	1	OLFM1,LOC100069029,LOC100069057,LOC100069096,WDR5,BRD3
25	36110418	37207857	1097440	3	1	OLFM1,LOC100069029,LOC100069057,LOC100069096,WDR5,BRD3
25	36110418	37675442	1565025	3	1	LOC100068625,LOC100066325,LOC100629160,CARD9,LOC100066352,SDCCAG3,PMPCA,LOC100068731,SEC16A,NOTCH1,MIR126,LOC100066378,LOC100068797,LOC100068815,LOC100068864,LOC100146707,OLFM1,LOC100069029,LOC100069057,LOC100069096,WDR5,BRD3
25	36110418	37929574	1819157	4	1	NACC2,LHX3,LOC100068607,LOC100068625,LOC100066325,LOC100629160,CARD9,LOC100066352,SDCCAG3,PMPCA,LOC100068731,SEC16A,NOTCH1,MIR126,LOC100066378,LOC100068797,LOC100068815,LOC100068864,LOC100146707,OLFM1,LOC100069029,LOC100069057,LOC100069096,WDR5,BRD3
25	36110418	38643208	2532791	4	1	MAN1B1,UAP1L1,LOC100067771,LOC100066152,LOC100067846,ABCA2,LOC100066181,LOC100146303,LOC100146610,PTGDS,LOC100147492,FBXW5,TANK,LOC100068036,MAMDC4,LOC100068092,LOC100068114,LOC100629484,LOC100068153,LOC100147579,LOC100068169,LOC100068193,LOC100068210,LGB2,LGB1,LOC100068354,LOC100068369,P19,LOC100068400,LOC100068421,CAMSAP1,LOC100068463,NACC2,LHX3,LOC100068607,LOC100068625,LOC100066325,LOC100629160,CARD9,LOC100066352,SDCCAG3,PMPCA,LOC100068731,SEC16A,NOTCH1,MIR126,LOC100066378,LOC100068797,LOC100068815,LOC100068864,LOC100146707,OLFM1,LOC100069029,LOC100069057,LOC100069096,WDR5,BRD3
25	36110418	39026923	2916506	4	1	PNPLA7,LOC100147094,ENTPD8,LOC100059408,EXD3,LOC100058595,LOC100059522,LOC100059560,LOC100058638,TUBB2C,LOC100147578,LOC100629886,LOC100058677,LOC100058717,LOC100146716,LOC100147202,LOC100059767,LOC100059826,GRIN1,MAN1A2,LOC100059924,MAN1B1,UAP1L1,LOC100067771,LOC100066152,LOC100067846,ABCA2,LOC100066181,LOC100146303,LOC100146610,PTGDS,LOC100147492,FBXW5,TANK,LOC100068036,MAMDC4,LOC100068092,LOC100068114,LOC100629484,LOC100068153,LOC100147579,LOC100068169,LOC100068193,LOC100068210,LGB2,LGB1,LOC100068354,LOC100068369,P19,LOC100068400,LOC100068421,CAMSAP1,LOC100068463,NACC2,LHX3,LOC100068607,LOC100068625,LOC100066325,LOC100629160,CARD9,LOC100066352,SDCCAG3,PMPCA,LOC100068731,SEC16A,NOTCH1,MIR126,LOC100066378,LOC100068797,LOC100068815,LOC100068864,LOC100146707,OLFM1,LOC100069029,LOC100069057,LOC100069096,WDR5,BRD3
25	36110418	39090329	2979912	3	2	LOC100059140,LOC100147491,PNPLA7,LOC100147094,ENTPD8,LOC100059408,EXD3,LOC100058595,LOC100059522,LOC100059560,LOC100058638,TUBB2C,LOC100147578,LOC100629886,LOC100058677,LOC100058717,LOC100146716,LOC100147202,LOC100059767,LOC100059826,GRIN1,MAN1A2,LOC100059924,MAN1B1,UAP1L1,LOC100067771,LOC100066152,LOC100067846,ABCA2,LOC100066181,LOC100146303,LOC100146610,PTGDS,LOC100147492,FBXW5,TANK,LOC100068036,MAMDC4,LOC100068092,LOC100068114,LOC100629484,LOC100068153,LOC100147579,LOC100068169,LOC100068193,LOC100068210,LGB2,LGB1,LOC100068354,LOC100068369,P19,LOC100068400,LOC100068421,CAMSAP1,LOC100068463,NACC2,LHX3,LOC100068607,LOC100068625,LOC100066325,LOC100629160,CARD9,LOC100066352,SDCCAG3,PMPCA,LOC100068731,SEC16A,NOTCH1,MIR126,LOC100066378,LOC100068797,LOC100068815,LOC100068864,LOC100146707,OLFM1,LOC100069029,LOC100069057,LOC100069096,WDR5,BRD3
25	37004731	39090329	2085599	3	1	LOC100059140,LOC100147491,PNPLA7,LOC100147094,ENTPD8,LOC100059408,EXD3,LOC100058595,LOC100059522,LOC100059560,LOC100058638,TUBB2C,LOC100147578,LOC100629886,LOC100058677,LOC100058717,LOC100146716,LOC100147202,LOC100059767,LOC100059826,GRIN1,MAN1A2,LOC100059924,MAN1B1,UAP1L1,LOC100067771,LOC100066152,LOC100067846,ABCA2,LOC100066181,LOC100146303,LOC100146610,PTGDS,LOC100147492,FBXW5,TANK,LOC100068036,MAMDC4,LOC100068092,LOC100068114,LOC100629484,LOC100068153,LOC100147579,LOC100068169,LOC100068193,LOC100068210,LGB2,LGB1,LOC100068354,LOC100068369,P19,LOC100068400,LOC100068421,CAMSAP1,LOC100068463,NACC2,LHX3,LOC100068607,LOC100068625,LOC100066325,LOC100629160,CARD9,LOC100066352,SDCCAG3,PMPCA,LOC100068731,SEC16A,NOTCH1,MIR126,LOC100066378,LOC100068797,LOC100068815,LOC100068864,LOC100146707
25	37207857	38176057	968201	1	1	LOC100068400,LOC100068421,CAMSAP1,LOC100068463,NACC2,LHX3,LOC100068607,LOC100068625,LOC100066325,LOC100629160,CARD9,LOC100066352,SDCCAG3,PMPCA,LOC100068731,SEC16A,NOTCH1,MIR126,LOC100066378,LOC100068797,LOC100068815,LOC100068864,LOC100146707

Chrom	Start	End	Size	Cn	Samples (n)	Genes
25	37543445	38460481	917037	1	2	TANK,LOC100068036,MAMDC4,LOC100068092,LOC100068114,LOC100629484,LOC100068153,LOC100147579,LOC100068169,LOC100068193,LOC100068210,LGB2,LGB1,LOC100068354,LOC100068369,P19,LOC100068400,LOC100068421,CAMSAP1,LOC100068463,NACC2,LHX3,LOC100068607,LOC100068625,LOC100066325,LOC100629160,CARD9,LOC100066352,SDCCAG3,PMPCA
25	38084663	38460481	375819	1	1	TANK,LOC100068036,MAMDC4,LOC100068092,LOC100068114,LOC100629484,LOC100068153,LOC100147579,LOC100068169,LOC100068193,LOC100068210,LGB2,LGB1,LOC100068354,LOC100068369,P19,LOC100068400,LOC100068421
25	38216374	39003009	786636	4	2	PNPLA7,LOC100147094,ENTPD8,LOC100059408,EXD3,LOC100058595,LOC100059522,LOC100059560,LOC100058638,TUBB2C,LOC100147578,LOC100629886,LOC100058677,LOC100058717,LOC100146716,LOC100147202,LOC100059767,LOC100059826,GRIN1,MAN1A2,LOC100059924,MAN1B1,UAP1L1,LOC100067771,LOC100066152,LOC100067846,ABCA2,LOC100066181,LOC100146303,LOC100146610,PTGDS,LOC100147492,FBXW5,TANK,LOC100068036,MAMDC4,LOC100068092,LOC100068114,LOC100629484,LOC100068153,LOC100147579,LOC100068169,LOC100068193,LOC100068210,LGB2,LGB1,LOC100068354
25	38240551	39003009	762459	3,4	1,8	PNPLA7,LOC100147094,ENTPD8,LOC100059408,EXD3,LOC100058595,LOC100059522,LOC100059560,LOC100058638,TUBB2C,LOC100147578,LOC100629886,LOC100058677,LOC100058717,LOC100146716,LOC100147202,LOC100059767,LOC100059826,GRIN1,MAN1A2,LOC100059924,MAN1B1,UAP1L1,LOC100067771,LOC100066152,LOC100067846,ABCA2,LOC100066181,LOC100146303,LOC100146610,PTGDS,LOC100147492,FBXW5,TANK,LOC100068036,MAMDC4,LOC100068092,LOC100068114,LOC100629484,LOC100068153,LOC100147579,LOC100068169,LOC100068193,LOC100068210,LGB2,LGB1
25	38643208	38912345	269138	3	1	EXD3,LOC100058595,LOC100059522,LOC100059560,LOC100058638,TUBB2C,LOC100147578,LOC100629886,LOC100058677,LOC100058717,LOC100146716,LOC100147202,LOC100059767,LOC100059826,GRIN1,MAN1A2,LOC100059924,MAN1B1
26	6538231	6636890	98660	1	1	
26	17942353	18207145	264793	1	1	UBE2J2,LOC100067458
26	18158669	18612443	453775	1	1	UBE2J2
26	22126353	22672137	545785	1	2	
26	39081069	40729847	1648779	3	1	SLC19A1,LOC100055712,LOC100055890,LOC100055928,LOC100050166,LOC100056049,ITGB2,LOC100630038,LOC100630015,LOC100056215,LOC100630163,LOC100630145,LOC100056557,LOC100146588,LOC100056649,LOC100630073,LOC100630231,LOC100630027,TSPEAR,LOC100050523,TRPM2,LOC100050601,LOC100056927,LOC100056971,DNMT3L,LOC100057048,LOC100057089,LOC100057131,AGPAT3,LOC100057293
26	39081069	41229124	2148056	3	1	LOC100049972,COL6A2,COL6A1,PCBP3,SLC19A1,LOC100055712,LOC100055890,LOC100055928,LOC100050166,LOC100056049,ITGB2,LOC100630038,LOC100630015,LOC100056215, LOC100630163,LOC100630145,LOC100056557,LOC100146588,LOC100056649,LOC100630073,LOC100630231,LOC100630027,TSPEAR,LOC100050523,TRPM2,LOC100050601,LOC100056927,LOC100056971,DNMT3L,LOC100057048,LOC100057089,LOC100057131,AGPAT3,LOC100057293
26	40138871	40252326	113456	1	1	
27	685561	14319372	13633812	3	1	LOC100062785,LOC100062721,MBOAT4,LOC100058409,LOC100058367,LOC100058323,LOC100062365,LOC100058282,PPP2CB,TEX15,LOC100058235,PURG,LOC100062021,NRG1,LOC100061264,LOC100061170,LOC100058148,LOC100061066,LOC100060999,LOC100058104,LOC100060811,UNC5D,KCNU1,LOC100058019,LOC100146543,LOC100057973,LOC100059780,GPR124,ZFP36L2,RAB11FIP1,LOC100059580,LOC100147322,LOC100057875,ASH2L,STAR,LOC100057784,BAG4,DDHD2,LOC100059204,WHSC1L1,LETM2,FGFR1,TACC1,LOC100057530,LOC100058701,LOC100058661,ADAM9,ADAM2,LOC100147614,LOC100630731,ADAM32,LOC100057011,LOC100630663,LOC100056890,LOC100056776,LOC100050681,IDO2,LOC100050602,LOC100056469,LOC100055845,LOC100050524,LOC100055757,AGPAT6,LOC100055502,MIR486,ANK1,LOC100630444,MYST3,LOC100054750,AP3M2,PLAT,LOC100630402, LOC100050167,LOC100050102,LOC100054568,LOC100050036,LOC100054432,LOC100049973,CHRNB3,CHRNA6,LOC100054001,LOC100053953,HOOK3,LOC100053751,LOC100053704,LOC100053457,LOC100053359,LOC100053115,CSGALNACT1,SH2D4A,TRNAA-UGC,PSD3,LOC100049837
27	3510418	4145140	634723	3	1	LOC100055845,LOC100050524,LOC100055757,AGPAT6,LOC100055502,MIR486,ANK1,LOC100630444
27	4959976	5928605	968630	1	1	ADAM32,LOC100057011,LOC100630663,LOC100056890,LOC100056776,LOC100050681,IDO2,LOC100050602
27	4959976	6539738	1579763	1	1	ADAM2,LOC100147614,LOC100630731,ADAM32,LOC100057011,LOC100630663,LOC100056890,LOC100056776,LOC100050681,IDO2,LOC100050602
27	12001424	12220092	218669	1	1	NRG1
27	17178274	18241350	1063077	1	1	LOC100049909
27	17367313	18241350	874038	1	1	LOC100049909
27	17367313	23545062	6177750	3	1	LOC100055580,LOC100630297,LOC100055254,LOC100055154,TRIML1,LOC100054569,LOC100054433,PCM1,LOC100054096,MTUS1,PDGFRL,LOC100050240,MTMR7,VPS37A,LOC100050103,LOC100053506,LOC100053360,LOC100050037,MSR1,TUSC3,MIR383,LOC100049909
27	17540371	17660789	120419	1	1	LOC100049909
27	17540371	18067175	526805	1	2	LOC100049909
27	17601828	17660789	58962	3	3	LOC100049909

Chrom	Start	End	Size	Cn	Samples (n)	Genes
27	24287352	39932455	15645104	3	1	LOC100629636,EQUCABV1R-PS932,EQUCABV1R-PS931,FBXO25,LOC100066006,LOC100065976,LOC100065419,LOC100065394,LOC100065372,LOC100065281,MYOM2,CSMD1,ANGPT2,LOC100064063,LOC100063996,XKR5,LOC100629676,LOC100629656,LOC100629644,LOC100629622,LOC100629602,DEFA17,LOC100629584,DEFA26,LOC100629563,DEFA35L,DEFA5,DEFA12,DEFA1,DEFA11,DEFA31L,LOC100629514,BD-1,LOC100629494,LOC100629479,LOC100629456,LOC100629426,LOC100629355,LOC100629408,LOC100629331,LOC100063044,LOC100146734,LOC100062056,LOC100629371,LOC100051492,LOC100061593,WDR17,LOC100051422,LOC100061344,LOC100629176,LOC100060890,NEIL3,LOC100060636,ODZ1,DCTD,WWC2,LOC100058927,LOC100058890,LOC100058784,LOC100051208,LOC100058620,LOC100051135,STOX2,ENPP6,IRF2,LOC100630717,CASP3,CCDC111,LOC100058020,ACSL1,LOC100057785,LOC100050837,LOC100050762,LOC100057615,SNX25,LRP2BP,LOC100630630,UFSP2,LOC100057211,LOC100057171,LOC100057090,SORBS2
27	29682432	29833466	151035	1	1	
27	30149065	30473284	324220	1	1	
27	36789720	36917670	127951	1	1	CSMD1
28	159333	1850650	1691318	3	1	TRHDE,TPH2,TBC1D15,LOC100056811,LOC100049910,LOC100049839
28	905851	1087329	181479	1	1	TRHDE
28	1694001	3555389	1861389	1	1	LOC100058150,LOC100058105,LOC100058062,KCNC2,LOC100050038
28	1794412	2147442	353031	1	1	
28	2497524	5151208	2653685	3	1	E2F7,LOC100050307,ZDHHC17,LOC100059205,OSBPL8,LOC100058970,NAP1L1,LOC100058739,KRR1,LOC100058283,LOC100058192,LOC100058150,LOC100058105,LOC100058062,KCNC2,LOC100050038
28	2806694	3090447	283754	1	1	
28	5942749	13916114	7973366	3	1	MGAT4C,LOC100630896,LOC100062225,RASSF9,ALX1,LRRIQ1,LOC100146893,LOC100050913,TMTC2,LOC100050763,LOC100061266,PPFIA2,ACSS3,LOC100050683,LOC100060703,LOC100050603,PTPRQ,OTOGL,LOC100630781,PPP1R12A,LOC100060291,LOC100050526,NAV3
28	8450753	8834693	383941	1	1	PPFIA2,ACSS3
28	10947499	11047216	99718	1	1	
28	14108681	14398793	290113	1	1	CEP290,LOC100051280,LOC100051136
28	14108681	14681697	573017	1	1	TMTC3,CEP290,LOC100051280,LOC100051136
28	28107669	28367173	259505	1	1	LOC100068154,ALDH1L2,LOC100053656,SLC41A2
28	30220749	43990487	13769739	3	1	TBC1D22A,CERK,GRAMD4,LOC100053508,LOC100053410,GTSE1,TTC38,LOC100053216,LOC100053165,PPARA,LOC100053118,MIRLET7A-2,LOC100053027,LOC100052926,LOC100052767,LOC100052553,SMC1B,LOC100052373,LOC100052016,LOC100051710,NUP50,LOC100629899,PHF21B,LOC100629845,LOC100051354,LOC100051281,LOC100146571,LOC100050914,LOC100050839,SAMM50,LOC100050604,LOC100050449,LOC100050374,EFCAB6,LOC100050170,SCUBE1,LOC100071120,LOC100071110,LOC100071100,LOC100056216,PACSIN2,LOC100071070,LOC100071058,LOC100071050,POLDIP3,LOC100071022,LOC100071013,LOC100071003,TCF20,LOC100070990,LOC100146596,LOC100147480,LOC100070962,LOC100056087,LOC100070954,CYP2D50,LOC100146391,LOC100070905,LOC100070895,LOC100147382,LOC100147660,LOC100629651,LOC100070817,LOC100056050,LOC100056008,NAGA,LOC100070785,SEPT3,CENPM,TNFRSF13C,LOC100070715,MIR33A,SREBF2,LOC100055970,MEI1,LOC100629531,LOC100070695,XRCC6,LOC100070677,LOC100055891,LOC100070658,LOC100055847,LOC100055803,LOC100055758,LOC100055713,LOC100055668,ZC3H7B,RANGAP1,CHADL,LOC100070590,EP300,LOC100055581,LOC100629166,XPNPEP3,LOC100055503,LOC100070515,LOC100070500,MCHR1,LOC100070476,LOC100070463,LOC100070427,TNRC6B,LOC100070395,LOC100055418,ENTHD1,LOC100070365,LOC100070342,LOC100070334,LOC100055376,MGAT3,LOC100070300,RPL3,LOC100070283,LOC100070273,APOBEC3Z3,APOBEC3Z2E,APOBEC3Z2A-Z2,APOBEC3Z1B,LOC100146802,LOC100055293,LOC100070185,LOC100070178,GTPBP1,LOC100055255,LOC100070131,LOC100070115,LOC100629284,DMC1,DDX17,LOC100070048,LOC100070031,LOC100055155,TMEM184B,PLA2G6,LOC100069993,LOC100069977,PICK1,SOX10,LOC100054935,LOC100069945,MICALL1,EIF3L,LOC100054842,LOC100630844,GCAT,LOC100069872,TRIOBP,LOC100054799,LOC100069841,LOC100630816,SH3BP1,LOC100147078,LOC100069787,LOC100054751,CARD10,LOC100069766,ELFN2,CYTH4,LOC100054701,LOC100069706,LOC100069688,LOC100069676,LOC100069647,LOC100630683,LOC100069619,LOC100054655,LOC100069602,LOC100069576,CSF2RB,NCF4,PVALB,LOC100069514,LOC100054570,LOC100054528,FOXRED2,LOC100069490,MYH9,LOC100069464,LOC100069448,LOC100069374,LOC100069362,L
28	32996239	34266193	1269955	3	1	NCF4,PVALB,LOC100069514,LOC100054570,LOC100054528,FOXRED2,LOC100069490,MYH9,LOC100069464,LOC100069448,LOC100069374,LOC100069362,LOC100069341,LOC100069335,MB,LOC100054386,MCM5
28	34266193	35068111	801919	3	1	EIF3L,LOC100054842,LOC100630844,GCAT,LOC100069872,TRIOBP,LOC100054799,LOC100069841,LOC100630816,SH3BP1,LOC100147078,LOC100069787,LOC100054751,CARD10,LOC100069766,ELFN2,CYTH4,LOC100054701,LOC100069706,LOC100069688,LOC100069676,LOC100069647,LOC100630683,LOC100069619,LOC100054655,LOC100069602,LOC100069576,CSF2RB
28	34455398	34874132	418735	1	1	LOC100147078,LOC100069787,LOC100054751,CARD10,LOC100069766,ELFN2,CYTH4,LOC100054701,LOC100069706,LOC100069688,LOC100069676,LOC100069647
28	34827251	35398805	571555	4	1	TMEM184B,PLA2G6,LOC100069993,LOC100069977,PICK1,SOX10,LOC100054935,LOC100069945,MICALL1,EIF3L,LOC100054842,LOC100630844,GCAT,LOC100069872,TRIOBP,LOC100054799,LOC100069841,LOC100630816,SH3BP1,LOC100147078,LOC100069787,LOC100054751

Chrom	Start	End	Size	Cn	Samples (n)	Genes
28	36188836	43384544	7195709	3	1	TBC1D22A,CERK,GRAMD4,LOC100053508,LOC100053410,GTSE1,TTC38,LOC100053216,LOC100053165,PPARA,LOC100053118,MIRLET7A-2,LOC100053027,LOC100052926,LOC100052767,LOC100052553,SMC1B,LOC100052373,LOC100052016,LOC100051710,NUP50,LOC100629899,PHF21B,LOC100629845,LOC100051354,LOC100051281,LOC100146571,LOC100050914,LOC100050839,SAMM50,LOC100050604,LOC100050449,LOC100050374,EFCAB6,LOC100050170,SCUBE1,LOC100071120,LOC100071110,LOC100071100,LOC100056216,PACSIN2,LOC100071070,LOC100071058,LOC100071050,POLDIP3,LOC100071022,LOC100071013,LOC100071003,TCF20,LOC100070990,LOC100146596,LOC100147480,LOC100070962,LOC100056087,LOC100070954,CYP2D50,LOC100146391,LOC100070905,LOC100070895,LOC100147382,LOC100147660,LOC100629651,LOC100070817,LOC100056050,LOC100056008,NAGA,LOC100070785,SEPT3,CENPM,TNFRSF13C,LOC100070715,MIR33A,SREBF2,LOC100055970,MEI1,LOC100629531,LOC100070695,XRCC6,LOC100070677,LOC100055891,LOC100070658,LOC100055847,LOC100055803,LOC100055758,LOC100055713,LOC100055668,ZC3H7B,RANGAP1,CHADL,LOC100070590,EP300,LOC100055581,LOC100629166,XPNPEP3,LOC100055503,LOC100070515,LOC100070500,MCHR1,LOC100070476,LOC100070463,LOC100070427,TNRC6B,LOC100070395,LOC100055418,ENTHD1,LOC100070365,LOC100070342,LOC100070334,LOC100055376,MGAT3,LOC100070300
28	36248740	36281744	33005	1	1	
28	37742058	40119761	2377704	3	1	LOC100050374,EFCAB6,LOC100050170,SCUBE1,LOC100071120,LOC100071110,LOC100071100,LOC100056216,PACSIN2,LOC100071070,LOC100071058,LOC100071050,POLDIP3,LOC100071022,LOC100071013,LOC100071003,TCF20,LOC100070990,LOC100146596,LOC100147480,LOC100070962,LOC100056087,LOC100070954,CYP2D50,LOC100146391,LOC100070905,LOC100070895,LOC100147382,LOC100147660,LOC100629651,LOC100070817,LOC100056050,LOC100056008,NAGA,LOC100070785,SEPT3,CENPM,TNFRSF13C,LOC100070715,MIR33A,SREBF2,LOC100055970,MEI1,LOC100629531,LOC100070695,XRCC6,LOC100070677,LOC100055891,LOC100070658,LOC100055847,LOC100055803,LOC100055758,LOC100055713,LOC100055668,ZC3H7B,RANGAP1,CHADL,LOC100070590
28	37927102	40749346	2822245	3	1	LOC100051281,LOC100146571,LOC100050914,LOC100050839,SAMM50,LOC100050604,LOC100050449,LOC100050374,EFCAB6,LOC100050170,SCUBE1,LOC100071120,LOC100071110,LOC100071100,LOC100056216,PACSIN2,LOC100071070,LOC100071058,LOC100071050,POLDIP3,LOC100071022,LOC100071013,LOC100071003,TCF20,LOC100070990,LOC100146596,LOC100147480,LOC100070962,LOC100056087,LOC100070954,CYP2D50,LOC100146391,LOC100070905,LOC100070895,LOC100147382,LOC100147660,LOC100629651,LOC100070817,LOC100056050,LOC100056008,NAGA,LOC100070785,SEPT3,CENPM,TNFRSF13C,LOC100070715,MIR33A,SREBF2,LOC100055970,MEI1,LOC100629531,LOC100070695,XRCC6,LOC100070677,LOC100055891,LOC100070658,LOC100055847,LOC100055803,LOC100055758
28	38798397	40311897	1513501	4	1	SAMM50,LOC100050604,LOC100050449,LOC100050374,EFCAB6,LOC100050170,SCUBE1,LOC100071120,LOC100071110,LOC100071100,LOC100056216,PACSIN2,LOC100071070,LOC100071058,LOC100071050,POLDIP3,LOC100071022,LOC100071013,LOC100071003
28	40288467	46147177	5858711	3	1	SHANK3,LOC100630687,LOC100056911,LOC100055523,LOC100055480,CPT1B,LOC100630606,LOC100056829,LOC100055399,LOC100146854,LOC100056712,NCAPH2,LMF2,LOC100056580,LOC100629546,SBF1,PPP6R2,LOC100056442,PLXNB2,LOC100056278,LOC100056239,LOC100146361,LOC100056192,SELO,TRABD,LOC100056108,MOV10L1,LOC100055870,LOC100146652,ZBED4,BRD1,LOC100630223,TBC1D22A,CERK,GRAMD4,LOC100053508,LOC100053410,GTSE1,TTC38,LOC100053216,LOC100053165,PPARA,LOC100053118,MIRLET7A-2,LOC100053027,LOC100052926,LOC100052767,LOC100052553,SMC1B,LOC100052373,LOC100052016,LOC100051710,NUP50,LOC100629899,PHF21B,LOC100629845,LOC100051354,LOC100051281,LOC100146571,LOC100050914,LOC100050839
28	40926909	43990487	3063579	3	1	TBC1D22A,CERK,GRAMD4,LOC100053508,LOC100053410,GTSE1,TTC38,LOC100053216,LOC100053165,PPARA,LOC100053118,MIRLET7A-2,LOC100053027,LOC100052926,LOC100052767,LOC100052553,SMC1B,LOC100052373,LOC100052016,LOC100051710,NUP50,LOC100629899,PHF21B
28	43785150	45398011	1612862	3	1	LOC100146652,ZBED4,BRD1,LOC100630223
28	45033500	46147177	1113678	1	1	SHANK3,LOC100630687,LOC100056911,LOC100055523,LOC100055480,CPT1B,LOC100630606,LOC100056829,LOC100055399,LOC100146854,LOC100056712,NCAPH2,LMF2,LOC100056580,LOC100629546,SBF1,PPP6R2,LOC100056442,PLXNB2,LOC100056278,LOC100056239,LOC100146361,LOC100056192,SELO,TRABD,LOC100056108,MOV10L1,LOC100055870,LOC100146652,ZBED4,BRD1
28	45186697	45935120	748424	1	1	SBF1,PPP6R2,LOC100056442,PLXNB2,LOC100056278,LOC100056239,LOC100146361,LOC100056192,SELO,TRABD,LOC100056108,MOV10L1,LOC100055870,LOC100146652,ZBED4,BRD1
29	282613	631698	349086	3	1	LOC100070840,LOC100070867,LOC100070887,LOC100058171,LOC100054174
29	477117	631698	154582	0,3	1,6	LOC100070840,LOC100070867,LOC100070887
29	889904	1113030	223127	4	1	LOC100146859
29	18222052	18335246	113195	1	1	LOC100068484,LOC100068465
29	24016990	25355633	1338644	1	1	LOC100069800
29	24976830	25205754	228925	1	1	LOC100069800
29	28244988	28590765	345778	3	1	LOC100057172,TUBAL3,NET1,LOC100057133,LOC100070422,LOC100070388
30	28004	750427	722424	1	1	LOC100630333,LOC100062135,LOC100062207,LOC100062241,LOC100062283,LOC100630213,HNRNPU,LOC100630169
30	1889883	2496898	607016	3	1	LOC100060872,LOC100630473
30	6640213	7347808	707596	3	1	LOC100058218,LOC100058310,LOC100058392,PARP1,LOC100058563,LOC100058602,LOC100054506,LOC100058643,CDC42BPA
30	6640213	7430735	790523	3,4	5,2	LOC100058044,LOC100058218,LOC100058310,LOC100058392,PARP1,LOC100058563,LOC100058602,LOC100054506,LOC100058643,CDC42BPA
30	8196894	8374816	177923	1	1	DNAH14
30	8472946	12352660	3879715	3	1	HHIPL2,TRNAT-UGU,LOC100055068,DUSP10,LOC100054295,LOC100054203,LOC100054157,LOC100054102,MARK1,LOC100050043,MIR664,RAB3GAP2,MIR194-2,MIR215,IARS2,LOC100053608,LOC100053511,LOC100629977,LOC100053461,LOC100053366,LOC100053271,LOC100053220,DISP1,LOC100070601,LOC100070594,CAPN8,CAPN2,TP53BP2,LOC100054130,LOC100055442,LOC100054179,NVL,LOC100629313,WDR26,LOC100055915

Chrom	Start	End	Size	Cn	Samples (n)	Genes
30	18941430	19963284	1021855	1	1	LOC100058741,LOC100050767,LOC100050688
30	20412866	20413139	274	1	1	
30	27410619	30032225	2621607	3	1	CHIT1,CHI3L1,MYBPH,LOC100052496,LOC100065194,LOC100146744,PPFIA4,TMEM183A,LOC100065011,LOC100064979,LOC100064917,ADIPOR1,KLHL12,LOC100064841,LOC100064760,KDM5B,LOC100146749,PPP1R12B,LOC100063899,LGR6,LOC100146338,PTPN7,LOC100052198,GPR37L1,LOC100146645,LOC100063775,LOC100052076,LMOD1,LOC100063707,IPO9,LOC100063631,LOC100051956,LOC100063505,LOC100063471,LOC100063440,LOC100146343,PKP1,LOC100063337,LOC100051835,CACNA1S,KIF21B,LOC100063112,LOC100062957,DDX59,LOC100146944,LOC100062616,NR5A2
30	28502215	28857432	355218	1	2	IPO9,LOC100063631,LOC100051956,LOC100063505
30	28502215	29117580	615366	1	1	LGR6,LOC100146338,PTPN7,LOC100052198,GPR37L1,LOC100146645,LOC100063775,LOC100052076,LMOD1,LOC100063707,IPO9,LOC100063631,LOC100051956,LOC100063505
31	4829031	4884348	55318	1	1	QKI
31	23869434	24094788	225355	1	1	LOC100067848,LOC100630671
31	23869434	24662405	792972	1	1	LOC100630719,LOC100067848,LOC100630671
