# Supplementary material for: Analysis of copy number variants by three detection algorithms and their association with body size in horses
Source: BMC Genomics. 2013 Jul 18;14:487. doi: 10.1186/1471-2164-14-487 (PMC3720552; doi:10.1186/1471-2164-14-487)
Supplement: Additional file 4 — Comparative analysis of three CNV detection algorithms. The table shows 50 CNVs derived from three detection algorithms, their position, size, copy number per sample and breed (HAN: Hanoverian; LUS: Lusitano; MAR: Maremanno; OLD: Oldenburg; RDK: Rhenish-German Cold-Blood; WES: Westphalian; HOL: Holsteiner; TB-H: Thoroughbred; AV: Arabian; RHD: Rhinelander horse; PRZ: Przewalski; BRAN: Brandenburger; TRAK: Trakehner; RPON: German Riding Pony). Text in PDF format. [file 1471-2164-14-487-S4.pdf]

Chromosome	Start	End	Size	Loss (per sample and breed)	Gain (per sample and breed)	Genes	Human orthologes
1	155487276	155593582	106307	HAN 3,LUS 2,MAR 2	HAN 1	LOC100072126,LOC100072129,LOC100072120,LOC100072117,LOC100072112,LOC100058312	OR4L1,OR4F6,GPCRLTM7,OR4F6,OR4F21,OR4K3
1	155487276	155652475	165200	HAN 23,LUS 5,MAR 3		LOC100072126,LOC100072136,LOC100072146,LOC100072141,LOC100072149,LOC100072129,LOC100072120,LOC100072117,LOC100072112,LOC100058312	OR4L1,OR4K3,OR4F21,OR4F15,OR4F15,OR4F6,GPCRLTM7,OR4F6,OR4F21,OR4K3
1	155487276	155656642	169367	HAN 75,LUS 13,MAR 10,OLD 1,RDK 1,WES 1		LOC100072126,LOC100072136,LOC100072146,LOC100072141,LOC100072149,LOC100072129,LOC100072120,LOC100072117,LOC100072112,LOC100058312	OR4L1,OR4K3,OR4F21,OR4F15,OR4F15,OR4F6,GPCRLTM7,OR4F6,OR4F21,OR4K3
1	155795029	156012981	217953	HAN 2,MAR 1		LOC100072179,LOC100072215,LOC100058354,LOC100072192,LOC100072202,LOC100072184,LOC100072227,LOC100072195,LOC100072221,LOC100072206,LOC100072211,LOC100058393	Olfr1318,OR11G2,OR4F21,Olfr1318,OR4F6,Olfr1318,OR4K3,OR4K3,OR4L1,OR4K3,OR4K3,OR4K17
1	155795029	156657881	862853	HAN 17,HOL 1,MAR 2,TB-H 1		LOC100072296,LOC100072258,LOC100072414,LOC100072179,LOC100072447,LOC100072426,LOC100072215,LOC100072251,LOC100058354,LOC100072237,LOC100072429,LOC100072368,LOC100072386,LOC100072192,LOC100072351,LOC100072271,LOC100072432,LOC100072275,LOC100072202,LOC100072265,LOC100072279,LOC100072306,LOC100072261,LOC100072184,LOC100072398,LOC100072342,LOC100072288,LOC100072317,LOC100072300,LOC100072380,LOC100072227,LOC100072195,LOC100072365,LOC100072443,LOC100058435,LOC100072402,LOC100072282,LOC100072290,LOC100072423,LOC100072320,LOC100072338,LOC100072221,LOC100072392,LOC100072354,LOC100072404,LOC100146987,LOC100072206,LOC100072211,LOC100072325,LOC100058393,LOC100072375,LOC100072345,LOC100072408,LOC100072254,LOC100072330,LOC100072335,LOC100072245	OR4F21,OR4N2,OR11G2,Olfr1318,OR4K1,OR11G2,OR11G2,OR4K15,OR4F21,OR4Q2,Olfr1318,Olfr1317,OR11H6,Olfr1318,OR4F15,OR11G2,Olfr1318,OR11H7,OR4F6,OR11H6,OR11H7,OR4F15,OR11H12,Olfr1318,OR11H4,OR4K3,OR11G2,Olfr1318,OR4F17,OR11G2,OR4K3,OR4K3,OR4F15,OR4K15,OR4K5,OR11G2,OR11G2,Olfr1318,OR11G2,Olfr1318,OR4L1,OR4L1,OR11G2,OR4F6,OR11H6,OR4F21,OR4K3,OR4K3,OR4F17,OR4K17,OR11G2,OR4F6,OR11G2,OR4N2,OR4K3,OR4K17,OR11G2
1	156125915	156491374	365460	AV 1,HAN 7,LUS 6,WES 1		LOC100072296,LOC100072368,LOC100072386,LOC100072351,LOC100072306,LOC100072398,LOC100072342,LOC100072288,LOC100072317,LOC100072300,LOC100072380,LOC100072365,LOC100072290,LOC100072320,LOC100072338,LOC100072392,LOC100072354,LOC100146987,LOC100072325,LOC100072375,LOC100072345,LOC100072330,LOC100072335	OR4F21,Olfr1317,OR11H6,OR4F15,OR4F15,OR11H4,OR4K3,OR11G2,Olfr1318,OR4F17,OR11G2,OR4F15,Olfr1318,Olfr1318,OR4L1,OR11G2,OR4F6,OR4F21,OR4F17,OR11G2,OR4F6,OR4K3,OR4K17
1	156449080	156657881	208802	HAN 1		LOC100072414,LOC100072447,LOC100072426,LOC100072429,LOC100072386,LOC100072432,LOC100072398,LOC100072443,LOC100058435,LOC100072402,LOC100072423,LOC100072392,LOC100072404,LOC100072408	OR11G2,OR4K1,OR11G2,Olfr1318,OR11H6,Olfr1318,OR11H4,OR4K15,OR4K5,OR11G2,OR11G2,OR11G2,OR11H6,OR11G2
1	156449080	156870455	421376	HAN 12		LOC100072414,LOC100072447,LOC100072426,LOC100072429,LOC100072386,LOC100072432,LOC100072464,LOC100072493,LOC100072499,LOC100072398,LOC100072469,LOC100629829,LOC100072443,LOC100058435,LOC100072495,LOC100072402,LOC100072423,LOC100072392,LOC100072404,LOC100072457,LOC100072479,LOC100072473,LOC100072408,LOC100072489,LOC100072477,LOC100072452	OR11G2,OR4K1,OR11G2,Olfr1318,OR11H6,Olfr1318,OR4K3,Tlr11,OR11G2,OR11H4,OR4K2,OR11H1,OR4K15,OR4K5,OR11G2,OR11G2,OR11G2,OR11G2,OR11H6,OR4K2,OR4S2,OR4N2,OR11G2,OR4Q3,OR4M1,OR4K15
1	156462763	156818876	356114	HAN 1		LOC100072414,LOC100072447,LOC100072426,LOC100072429,LOC100072386,LOC100072432,LOC100072464,LOC100072398,LOC100072469,LOC100629829,LOC100072443,LOC100058435,LOC100072402,LOC100072423,LOC100072392,LOC100072404,LOC100072457,LOC100072479,LOC100072473,LOC100072408,LOC100072489,LOC100072477,LOC100072452	OR11G2,OR4K1,OR11G2,Olfr1318,OR11H6,Olfr1318,OR4K3,OR11H4,OR4K2,OR11H1,OR4K15,OR4K5,OR11G2,OR11G2,OR11G2,OR11H6,OR4K2,OR4S2,OR4N2,OR11G2,OR4Q3,OR4M1,OR4K15
1	156529837	156870455	340619	HAN 4		LOC100072414,LOC100072447,LOC100072426,LOC100072429,LOC100072432,LOC100072464,LOC100072493,LOC100072499,LOC100072469,LOC100629829,LOC100072443,LOC100058435,LOC100072495,LOC100072423,LOC100072457,LOC100072479,LOC100072473,LOC100072489,LOC100072477,LOC100072452	OR11G2,OR4K1,OR11G2,Olfr1318,Olfr1318,OR4K3,Tlr11,OR11G2,OR4K2,OR11H1,OR4K15,OR4K5,OR11G2,OR11G2,OR4K2,OR4S2,OR4N2,OR4Q3,OR4M1,OR4K15
1	178798269	179550475	752207		HAN 3	LOC100062807,LOC100062668,LOC100062944	ZNF257,uncharacterized,YWHAZ
2	106062109	106063373	1265	HAN 14,RHD 1,TB-H 1,WES 1			
3	41567820	41635136	67317		HAN 3,HOL 1		
3	65705932	65951800	245869	HAN 1	HAN 2	LOC100067903,LOC100066472,LOC100629168,LOC100067952,LOC100067974,LOC100067994,LOC100629252,LOC100629210,LOC100066501,LOC100068012	UGT2B17,UGT2B17,UGT2B17,UGT2B17,UGT2B17,MGC152010,MOCS3,TIGD1,UGT2B17,UGT2B10
4	52424614	52612016	187403		HAN 1	LOC100067077	uncharacterized
5	37840041	37916448	76408	HAN 7,TB-H 2,WES 1		LOC100053475,LOC100057667	APCS,OR10J6
5	88243192	88258862	15671		HAN 25,TB-H 2,TRAK 1		
6	26086675	26126581	39907		AV 1,HAN 9,OLD 1,RHD 1,WES 1	LOC100067436,LOC100147336	AQP12B,GPR35

Chromosome	Start	End	Size	Loss (per sample and breed)	Gain (per sample and breed)	Genes	Human orthologes
6	72032729	72485833	453105	AV 1,HAN 5,HOL 1		LOC100630091,LOC100050632,LOC100055222,LOC100055352,LOC100055645,LOC100055600,LOC100055946,LOC100055778,LOC100146480,LOC100054585,LOC100050555,LOC100054671,LOC100147637,LOC100054771,LOC100055272,LOC100146474,LOC100055559,LOC100055434,LOC100055688,LOC100055866,LOC100055818,LOC100055734,LOC100050712,LOC100055520,LOC100147260,LOC100055477,LOC100055308,LOC100055176,LOC100055082,LOC100055039,LOC100054995,LOC100054952,LOC100054904,LOC100054856,LOC100054816,LOC100146580,LOC100054626	OR6C3,OR6C2,OR6C2,OR6C2,OR6C1,OR6C3,OR6C6,OR6C74,OR6C3,OR10A7,OR9K2,OR6C1,OR6C75,OR6C74,OR6C2,OR6C2,OR6C75,OR6C2,OR6C3,OR6C3,OR6C4,OR6C1,OR6C75,OR6C76,OR6C75,OR6C76,OR6C2,OR6C2,OR6C2,OR6C68,OR6C2,OR6C65,OPN4,OR6C1,OR6C1,OR6C3,OR6C4
6	72032729	72493903	461175	AV 10,HAN 44,LUS 16,OLD 1	AV 1	LOC100630091,LOC100050632,LOC100055222,LOC100055352,LOC100055645,LOC100055600,LOC100055986,LOC100055946,LOC100055778,LOC100146480,LOC100054585,LOC100050555,LOC100054671,LOC100147637,LOC100054771,LOC100055272,LOC100146474,LOC100055559,LOC100055434,LOC100055688,LOC100055866,LOC100055818,LOC100055734,LOC100050712,LOC100055520,LOC100147260,LOC100055477,LOC100055308,LOC100055176,LOC100055082,LOC100055039,LOC100054995,LOC100054952,LOC100054904,LOC100054856,LOC100054816,LOC100146580,LOC100054626	OR6C3,OR6C2,OR6C2,OR6C2,OR6C1,OR6C3,OR6C4,OR6C6,OR6C74,OR6C3,OR10A7,OR9K2,OR6C1,OR6C75,OR6C74,OR6C2,OR6C2,OR6C75,OR6C2,OR6C3,OR6C3,OR6C4,OR6C1,OR6C75,OR6C76,OR6C75,OR6C76,OR6C2,OR6C2,OR6C2,OR6C68,OR6C2,OR6C65,OPN4,OR6C1,OR6C1,OR6C3,OR6C4
6	72032729	72607543	574815	AV 1,HAN 2,LUS 1,OLD 1		LOC100630091,LOC100050632,LOC100055222,LOC100055352,LOC100055645,LOC100055600,LOC100055986,LOC100055946,LOC100055778,LOC100146480,LOC100054585,LOC100050555,LOC100054671,LOC100147637,LOC100054771,LOC100055272,LOC100146474,LOC100055559,LOC100055434,LOC100055688,LOC100056234,LOC100056025,LOC100056186,LOC100630654,LOC100056100,LOC100055866,LOC100055818,LOC100055734,LOC100050712,LOC100055520,LOC100147260,LOC100055477,LOC100055308,LOC100055176,LOC100055082,LOC100055039,LOC100054995,LOC100054952,LOC100054904,LOC100054856,LOC100054816,LOC100146580,LOC100054626	OR6C3,OR6C2,OR6C2,OR6C2,OR6C1,OR6C3,OR6C4,OR6C6,OR6C74,OR6C3,OR10A7,OR9K2,OR6C1,OR6C75,OR6C74,OR6C2,OR6C2,OR6C75,OR6C2,OR6C3,OR6C76,OR10A7,OR9K2,OR2T33,OR6C75,OR6C3,OR6C4,OR6C1,OR6C75,OR6C76,OR6C75,OR6C76,OR6C2,OR6C2,OR6C2,OR6C68,OR6C2,OR6C65,OPN4,OR6C1,OR6C1,OR6C3,OR6C4
7	31406445	31520977	114533	HAN 8		LOC100071915,LOC100071904,LOC100071894,LOC100071910,LOC100630881	OR8D4,OR6T1,OR10G6,OR4D5,OR8B3
7	52610482	52677786	67305		HAN 5	LOC100146282,LOC100064357,LOC100064296	Olfr18,OR7D4,ZDHHC19
7	73083306	73197149	113844	AV 4,MAR 1,PRZ 2		LOC100146503,LOC100067901,LOC100067972,LOC100067873,LOC100068010,LOC100067992,LOC100067950,LOC100067928,LOC100147186,LOC100146803	OR51L1,OR52E1,OR52D1,OR51G1,OR52J3,OR52E2,OR52E2,OR52E1,OR52D1,OR52E2
8	4280605	4430473	149869	HAN 6,HOL 1,RDK 1		LOC100062472	Ig lambda chain V-I region BL2-like
8	4430473	4621044	190572	HAN 14		LOC100062472	Ig lambda chain V-I region BL2-like
8	4430473	4646812	216340	HAN 2		LOC100062472	Ig lambda chain V-I region BL2-like
8	4537919	4621044	83126	HAN 27,HOL 1,OLD 1,PRZ 1		LOC100062472	Ig lambda chain V-I region BL2-like
9	31574454	31574969	516	AV 3,MAR 1		PXDNL	PXDNL
10	674485	1271225	596741		HAN 4	LOC100053533,LOC100053634	GAPDH,UQCRFS1
11	54645681	54812394	166714	AV 2,HAN 2			
12	12524489	13401991	877503		AV 1,LUS 3	LOC100146383,LOC100054355,LOC100054307,LOC100054258,LOC100054215,LOC100054169,LOC100054120,LOC100054065,LOC100054021,LOC100050556,LOC100053872,LOC100050476,LOC100053820,LOC100053768,LOC100629822,LOC100053672,LOC100053625,LOC100053568,LOC100053527,LOC100147669,LOC100053479,LOC100053427,LOC100053382,LOC100053328,LOC100053284,LOC100053236,LOC100053185,LOC100053134,LOC100053085,LOC100053045,LOC100052992,LOC100146502,LOC100052946,LOC100146388,LOC100052842,LOC100147383,LOC100052788,LOC100052732,LOC100050399,LOC100052683,LOC100052629,LOC100052515,LOC100052454,LOC100052398,LOC100052341,LOC100052217,LOC100052157,LOC100052096,LOC100052037,LOC100146189,LOC100051977,LOC100051914,LOC100146810,LOC100050328,LOC100051731,LOC100051664,LOC100051592,LOC100051520,LOC100051452,LOC100051381,LOC100050265,LOC100630831	OR5M11,Olfr1030,OR5AP2,OR5AR1,OR10A7,Olfr1009,OR5G3,OR5D13,OR5T2,OR8H1,OR5I1,Olfr1131,OR5AP2,OR5W2,OR5I1,OR5L1,OR5D18,OR5L1,OR5D18,OR5D14,OR5D13,OR5D13,OR5D13,OR5D13,OR4P4,OR4P4,OR4P4,OR4P4,Olfr998,OR5G3,OR5G3,Olfr1002,OR6C70,Olfr1009,Olfr1009,OR9G4,Olfr1013,Olfr1013,Olfr1013,Olfr1013,OR6A2,OR5AR1,OR5AP2,OR5M3,OR5M9,OR5M3,OR5M9,OR5AL1,OR8U1,OR8J3,OR8J3,OR8K5,OR8K3,OR8J2,OR5T1,OR5T2,OR8H3,OR8I2,OR5I1,OR5I1,OR5I1,OR10AG1

Chromosome	Start	End	Size	Loss (per sample and breed)	Gain (per sample and breed)	Genes	Human orthologes
12	12829176	13401991	572816		HAN 2	LOC100146383,LOC100054355,LOC100054307,LOC100054258,LOC100054215,LOC100054169,LOC100054120,LOC100054065,LOC100054021,LOC100050556,LOC100053872,LOC100050476,LOC100053820,LOC100053768,LOC100629822,LOC100053672,LOC100053625,LOC100053568,LOC100053527,LOC100147669,LOC100053479,LOC100053427,LOC100053382,LOC100053328,LOC100053284,LOC100053236,LOC100053185,LOC100053134,LOC100053085,LOC100053045,LOC100052992,LOC100146502,LOC100052946,LOC100146388,LOC100052842,LOC100147383,LOC100052788,LOC100052732,LOC100050399,LOC100052683	OR5M11,Olfr1030,OR5AP2,OR5AR1,OR10A7,Olfr1009,OR5G3,OR5D13,OR5T2,OR8H1,OR5I1,Olfr1131,OR5AP2,OR5W2,OR5I1,OR5L1,OR5D18,OR5L1,OR5D18,OR5D14,OR5D13,OR5D13,OR5D13,OR5D13,OR4P4,OR4P4,OR4P4,OR4P4,Olfr998,OR5G3,OR5G3,Olfr1002,OR6C70,Olfr1009,Olfr1009,OR9G4,Olfr1013,Olfr1013,Olfr1013,Olfr1013
12	12829176	13422256	593081		HAN 1,HOL 1	LOC100054453,LOC100146383,LOC100054355,LOC100054307,LOC100054258,LOC100054215,LOC100054169,LOC100054120,LOC100054065,LOC100054021,LOC100050556,LOC100053872,LOC100050476,LOC100053820,LOC100053768,LOC100629822,LOC100053672,LOC100053625,LOC100053568,LOC100053527,LOC100147669,LOC100053479,LOC100053427,LOC100053382,LOC100053328,LOC100053284,LOC100053236,LOC100053185,LOC100053134,LOC100053085,LOC100053045,LOC100052992,LOC100146502,LOC100052946,LOC100146388,LOC100052842,LOC100147383,LOC100052788,LOC100052732,LOC100050399,LOC100052683	OR5M10,OR5M11,Olfr1030,OR5AP2,OR5AR1,OR10A7,Olfr1009,OR5G3,OR5D13,OR5T2,OR8H1,OR5I1,Olfr1131,OR5AP2,OR5W2,OR5I1,OR5L1,OR5D18,OR5L1,OR5D18,OR5D14,OR5D13,OR5D13,OR5D13,OR5D13,OR4P4,OR4P4,OR4P4,OR4P4,Olfr998,OR5G3,OR5G3,Olfr1002,OR6C70,Olfr1009,Olfr1009,OR9G4,Olfr1013,Olfr1013,Olfr1013,Olfr1013
12	13149957	14128309	978353		HAN 4,MAR 1	LOC100056626,LOC100056578,LOC100056531,LOC100056488,LOC100629158,LOC100050633,LOC100056395,LOC100056356,LOC100056314,LOC100056273,LOC100056235,LOC100056187,LOC100056146,LOC100630788,LOC100629923,LOC100056026,LOC100055988,LOC100146992,LOC100055947,LOC100147569,LOC100146290,LOC100055908,LOC100055867,LOC100055780,LOC100630577,LOC100055646,LOC100055601,LOC100055561,LOC100055521,LOC100055478,LOC100055435,LOC100055397,LOC100055353,LOC100055309,LOC100055273,LOC100055224,LOC100055177,LOC100055129,LOC100055083,LOC100052898,LOC100147187,LOC100054954,LOC100054906,LOC100054858,LOC100054773,LOC100054722,LOC100054673,LOC100054627,LOC100054586,LOC100054541,LOC100054498,LOC100054453,LOC100146383,LOC100054355,LOC100054307,LOC100054258,LOC100054215,LOC100054169,LOC100054120,LOC100054065,LOC100054021,LOC100050556,LOC100053872,LOC100050476,LOC100053820,LOC100053768,LOC100629822	OR4A47,OR4A47,OR4A15,OR4A16,OR4C15,OR4C11,OR4A47,OR4A47,OR4A47,OR4A47,OR4A15,OR4A16,OR4P4,OR4S2,OR4C46,OR5D13,OR5D13,OR5D13,OR5D13,OR5D13,OR5D14,OR5D16,OR5AP2,OR5W2,OR10AG1,OR5T1,OR10AG1,OR10AG1,OR5T2,OR5T2,OR8K3,OR8K3,OR8K3,OR8K3,OR8K3,OR5I1,OR8K5,OR8K1,OR8J3,OR8J3,OR8U1,OR5AL1,OR5AL1,TBCK,OR5R1,OR5M9,OR5M3,OR5M3,Olfr1030,Olfr1030,Olfr1030,OR5M10,OR5M11,Olfr1030,OR5AP2,OR5AR1,OR10A7,Olfr1009,OR5G3,OR5D13,OR5T2,OR8H1,OR5I1,Olfr1131,OR5AP2,OR5W2,OR5I1
12	13439146	14391372	952227	HAN 2	HAN 2	LOC100057026,LOC100050714,LOC100056989,LOC100056947,LOC100056906,LOC100056869,LOC100146809,LOC100067978,LOC100067957,LOC100056826,LOC100056793,LOC100056755,LOC100147000,LOC100056708,LOC100056626,LOC100056578,LOC100056531,LOC100056488,LOC100629158,LOC100050633,LOC100056395,LOC100056356,LOC100056314,LOC100056273,LOC100056235,LOC100056187,LOC100056146,LOC100630788,LOC100629923,LOC100056026,LOC100055988,LOC100146992,LOC100055947,LOC100147569,LOC100146290,LOC100055908,LOC100055867,LOC100055780,LOC100630577,LOC100055646,LOC100055601,LOC100055561,LOC100055521,LOC100055478,LOC100055435,LOC100055397,LOC100055353,LOC100055309,LOC100055273,LOC100055224,LOC100055177,LOC100055129,LOC100055083,LOC100052898,LOC100147187,LOC100054954,LOC100054906,LOC100054858,LOC100054773,LOC100054722,LOC100054673,LOC100054627,LOC100054586,LOC100054541	OR4B1,OR4C3,OR4C5,OR4A16,OR4A15,OR2F1,OR4P4,OR4P4,OR4P4,OR5D14,OR5D13,OR5D14,OR5D18,OR4A47,OR4A47,OR4A47,OR4A15,OR4A16,OR4C15,OR4C11,OR4A47,OR4A47,OR4A47,OR4A47,OR4A15,OR4A16,OR4P4,OR4S2,OR4C46,OR5D13,OR5D13,OR5D13,OR5D13,OR5D13,OR5D14,OR5D16,OR5AP2,OR5W2,OR10AG1,OR5T1,OR10AG1,OR10AG1,OR5T2,OR5T2,OR8K3,OR8K3,OR8K3,OR8K3,OR8K3,OR5I1,OR8K5,OR8K1,OR8J3,OR8J3,OR8U1,OR5AL1,OR5AL1,TBCK,OR5R1,OR5M9,OR5M3,OR5M3,Olfr1030,Olfr1030

Chromosome	Start	End	Size	Loss (per sample and breed)	Gain (per sample and breed)	Genes	Human orthologes
12	13488187	14391372	903186		AV 1,HAN 5	LOC100057026,LOC100050714,LOC100056989,LOC100056947,LOC100056906,LOC100056869,LOC100146809,LOC100067978,LOC100067957,LOC100056826,LOC100056793,LOC100056755,LOC100147000,LOC100056708,LOC100056626,LOC100056578,LOC100056531,LOC100056488,LOC100629158,LOC100050633,LOC100056395,LOC100056356,LOC100056314,LOC100056273,LOC100056235,LOC100056187,LOC100056146,LOC100630788,LOC100629923,LOC100056026,LOC100055988,LOC100146992,LOC100055947,LOC100147569,LOC100146290,LOC100055908,LOC100055867,LOC100055780,LOC100630577,LOC100055646,LOC100055601,LOC100055561,LOC100055521,LOC100055478,LOC100055435,LOC100055397,LOC100055353,LOC100055309,LOC100055273,LOC100055224,LOC100055177,LOC100055129,LOC100055083,LOC100052898,LOC100147187,LOC100054954,LOC100054906,LOC100054858,LOC100054773,LOC100054722	OR4B1,OR4C3,OR4C5,OR4A16,OR4A15,OR2F1,OR4P4,OR4P4,OR4P4,OR5D14,OR5D13,OR5D14,OR5D18,OR4A47,OR4A47,OR4A47,OR4A15,OR4A16,OR4C15,OR4C11,OR4A47,OR4A47,OR4A47,OR4A47,OR4A15,OR4A16,OR4P4,OR4S2,OR4C46,OR5D13,OR5D13,OR5D13,OR5D13,OR5D13,OR5D14,OR5D16,OR5AP2,OR5W2,OR10AG1,OR5T1,OR10AG1,OR10AG1,OR5T2,OR5T2,OR8K3,OR8K3,OR8K3,OR8K3,OR8K3,OR5I1,OR8K5,OR8K1,OR8J3,OR8J3,OR8U1,OR5AL1,OR5AL1,TBCK,OR5R1,OR5M9
12	13573356	14354314	780959		HAN 3	LOC100056947,LOC100056906,LOC100056869,LOC100146809,LOC100067978,LOC100067957,LOC100056826,LOC100056793,LOC100056755,LOC100147000,LOC100056708,LOC100056626,LOC100056578,LOC100056531,LOC100056488,LOC100629158,LOC100050633,LOC100056395,LOC100056356,LOC100056314,LOC100056273,LOC100056235,LOC100056187,LOC100056146,LOC100630788,LOC100629923,LOC100056026,LOC100055988,LOC100146992,LOC100055947,LOC100147569,LOC100146290,LOC100055908,LOC100055867,LOC100055780,LOC100630577,LOC100055646,LOC100055601,LOC100055561,LOC100055521,LOC100055478,LOC100055435,LOC100055397,LOC100055353,LOC100055309,LOC100055273,LOC100055224,LOC100055177,LOC100055129,LOC100055083,LOC100052898	OR4A16,OR4A15,OR2F1,OR4P4,OR4P4,OR4P4,OR5D14,OR5D13,OR5D14,OR5D18,OR4A47,OR4A47,OR4A47,OR4A15,OR4A16,OR4C15,OR4C11,OR4A47,OR4A47,OR4A47,OR4A47,OR4A15,OR4A16,OR4P4,OR4S2,OR4C46,OR5D13,OR5D13,OR5D13,OR5D13,OR5D13,OR5D14,OR5D16,OR5AP2,OR5W2,OR10AG1,OR5T1,OR10AG1,OR10AG1,OR5T2,OR5T2,OR8K3,OR8K3,OR8K3,OR8K3,OR8K3,OR5I1,OR8K5,OR8K1,OR8J3,OR8J3
12	13945011	14128309	183299	MAR 1	HAN 11,LUS 1,MAR 1	LOC100056626,LOC100056578,LOC100056531,LOC100056488,LOC100629158,LOC100050633,LOC100056395,LOC100056356,LOC100056314,LOC100056273,LOC100056235,LOC100056187,LOC100056146	OR4A47,OR4A47,OR4A15,OR4A16,OR4C15,OR4C11,OR4A47,OR4A47,OR4A47,OR4A47,OR4A15,OR4A16,OR4P4
12	13945011	14777981	832971		AV 1,BRAN 1,HAN 8,LUS 2,MAR 2,OLD 1	LOC100058077,LOC100147282,LOC100058036,LOC100057989,LOC100057945,LOC100057894,LOC100057845,LOC100057804,LOC100057756,LOC100057709,LOC100057670,LOC100057589,LOC100057547,LOC100057513,LOC100057472,LOC100057433,LOC100057393,LOC100057352,LOC100057310,LOC100147286,LOC100146990,LOC100057269,LOC100057228,LOC100057191,LOC100057110,LOC100146600,LOC100057026,LOC100050714,LOC100056989,LOC100056947,LOC100056906,LOC100056869,LOC100146809,LOC100067978,LOC100067957,LOC100056826,LOC100056793,LOC100056755,LOC100147000,LOC100056708,LOC100056626,LOC100056578,LOC100056531,LOC100056488,LOC100629158,LOC100050633,LOC100056395,LOC100056356,LOC100056314,LOC100056273,LOC100056235,LOC100056187,LOC100056146	OR10AG1,OR10AG1,OR10AG1,OR10AG1,OR10AG1,OR10AG1,OR5F1,OR5AS1,OR5I1,OR5T1,OR5T2,OR8K3,OR5I1,OR8K1,OR8J3,OR8K3,OR8K3,OR8K3,OR8K3,OR8K3,OR5I1,OR8K1,OR8J3,PTPRH,OR4B1,OR4B1,OR4B1,OR4C3,OR4C5,OR4A16,OR4A15,OR2F1,OR4P4,OR4P4,OR4P4,OR5D14,OR5D13,OR5D14,OR5D18,OR4A47,OR4A47,OR4A47,OR4A15,OR4A16,OR4C15,OR4C11,OR4A47,OR4A47,OR4A47,OR4A47,OR4A15,OR4A16,OR4P4
12	14108229	14777981	669753		HAN 1,LUS 3,TRAK 1	LOC100058077,LOC100147282,LOC100058036,LOC100057989,LOC100057945,LOC100057894,LOC100057845,LOC100057804,LOC100057756,LOC100057709,LOC100057670,LOC100057589,LOC100057547,LOC100057513,LOC100057472,LOC100057433,LOC100057393,LOC100057352,LOC100057310,LOC100147286,LOC100146990,LOC100057269,LOC100057228,LOC100057191,LOC100057110,LOC100146600,LOC100057026,LOC100050714,LOC100056989,LOC100056947,LOC100056906,LOC100056869,LOC100146809,LOC100067978,LOC100067957,LOC100056826,LOC100056793,LOC100056755,LOC100147000,LOC100056708,LOC100056626,LOC100056578	OR10AG1,OR10AG1,OR10AG1,OR10AG1,OR10AG1,OR10AG1,OR5F1,OR5AS1,OR5I1,OR5T1,OR5T2,OR8K3,OR5I1,OR8K1,OR8J3,OR8K3,OR8K3,OR8K3,OR8K3,OR8K3,OR5I1,OR8K1,OR8J3,PTPRH,OR4B1,OR4B1,OR4B1,OR4C3,OR4C5,OR4A16,OR4A15,OR2F1,OR4P4,OR4P4,OR4P4,OR5D14,OR5D13,OR5D14,OR5D18,OR4A47,OR4A47,OR4A47
18	11660478	12399073	738596		LUS 2	LOC100052304,LOC100052243,LOC100049893,STEAP3,LOC100629302,DBI	uncharacterized,MARCO,C1QL2,STEAP3,C2orf76,DBI
20	32059082	32210308	151227	HAN 10		LOC100059844,LOC100059681	TSPAN17,C6orf10
20	32127612	32210308	82697	AV 1,HAN 15,MAR 3,OLD 1,RHD 2,WES 1			
24	32416012	32628728	212717		HAN 3	TTC8,EML5	TTC8,EML5

Chromosome	Start	End	Size	Loss (per sample and breed)	Gain (per sample and breed)	Genes	Human orthologes
25	26318531	26918263	599733	AV 1,HAN 4		LOC100071251,LOC100067520,LOC100071258,LOC100071264,LOC100071270,LOC100071275,LOC100071278,LOC100071283,LOC100071287,LOC100071297,LOC100146817,LOC100071311,LOC100071317,LOC100147676,LOC100071322,LOC100071329,LOC100071332,LOC100071338,LOC100071346,LOC100071352,LOC100071358,LOC100071365,LOC100071376,LOC100071382,LOC100071392,LOC100071402,LOC100071407,LOC100071413,LOC100071420,LOC100146823,LOC100071438,LOC100071445,LOC100071448,LOC100071452,LOC100071460,LOC100071469,LOC100071479,LOC100071486,LOC100071488,LOC100071492,LOC100071496,LOC100071502	OR1L4,OR1L4,OR1L6,OR1L3,OR1L1,OR1B1,OR1Q1,OR1N1,OR1J2,OR1N2,OR1L8,OR1L8,OR1J2,OR1J4,OR1J4,OR1J4,OR1J4,OR1L8,OR1L8,OR1N1,OR1J1,OR1J2,OR1J4,OR1J4,OR1J2,OR1J2,OR1L6,OR1B1,Olfr859,OR1Q1,OR1N2,OR1G1,OR1N2,OR1J1,OR1J2,OR1L6,OR1L3,OR1L1,OR1J2,OR1J4,OR1J2,OR1J2
25	26318531	26942120	623590	AV 1,HAN 7		LOC100071244,LOC100071251,LOC100067520,LOC100071258,LOC100071264,LOC100071270,LOC100071275,LOC100071278,LOC100071283,LOC100071287,LOC100071297,LOC100146817,LOC100071311,LOC100071317,LOC100147676,LOC100071322,LOC100071329,LOC100071332,LOC100071338,LOC100071346,LOC100071352,LOC100071358,LOC100071365,LOC100071376,LOC100071382,LOC100071392,LOC100071402,LOC100071407,LOC100071413,LOC100071420,LOC100146823,LOC100071438,LOC100071445,LOC100071448,LOC100071452,LOC100071460,LOC100071469,LOC100071479,LOC100071486,LOC100071488,LOC100071492,LOC100071496,LOC100071502	OR1L4,OR1L4,OR1L4,OR1L6,OR1L3,OR1L1,OR1B1,OR1Q1,OR1N1,OR1J2,OR1N2,OR1L8,OR1L8,OR1J2,OR1J4,OR1J4,OR1J4,OR1J4,OR1L8,OR1L8,OR1N1,OR1J1,OR1J2,OR1J4,OR1J4,OR1J2,OR1J2,OR1L6,OR1B1,Olfr859,OR1Q1,OR1N2,OR1G1,OR1N2,OR1J1,OR1J2,OR1L6,OR1L3,OR1L1,OR1J2,OR1J4,OR1J2,OR1J2
25	26361000	26918263	557264	HAN 10		LOC100071251,LOC100067520,LOC100071258,LOC100071264,LOC100071270,LOC100071275,LOC100071278,LOC100071283,LOC100071287,LOC100071297,LOC100146817,LOC100071311,LOC100071317,LOC100147676,LOC100071322,LOC100071329,LOC100071332,LOC100071338,LOC100071346,LOC100071352,LOC100071358,LOC100071365,LOC100071376,LOC100071382,LOC100071392,LOC100071402,LOC100071407,LOC100071413,LOC100071420,LOC100146823,LOC100071438,LOC100071445,LOC100071448,LOC100071452,LOC100071460,LOC100071469,LOC100071479,LOC100071486	OR1L4,OR1L4,OR1L6,OR1L3,OR1L1,OR1B1,OR1Q1,OR1N1,OR1J2,OR1N2,OR1L8,OR1L8,OR1J2,OR1J4,OR1J4,OR1J4,OR1J4,OR1L8,OR1L8,OR1N1,OR1J1,OR1J2,OR1J4,OR1J4,OR1J2,OR1J2,OR1L6,OR1B1,Olfr859,OR1Q1,OR1N2,OR1G1,OR1N2,OR1J1,OR1J2,OR1L6,OR1L3,OR1L1
25	26361000	26942120	581121	AV 4,HAN 44,HOL 3,LUS 1,MAR 2,OLD 4,RDK 1,RHD 3,RPON 1,TRAK 3,WES 12		LOC100071244,LOC100071251,LOC100067520,LOC100071258,LOC100071264,LOC100071270,LOC100071275,LOC100071278,LOC100071283,LOC100071287,LOC100071297,LOC100146817,LOC100071311,LOC100071317,LOC100147676,LOC100071322,LOC100071329,LOC100071332,LOC100071338,LOC100071346,LOC100071352,LOC100071358,LOC100071365,LOC100071376,LOC100071382,LOC100071392,LOC100071402,LOC100071407,LOC100071413,LOC100071420,LOC100146823,LOC100071438,LOC100071445,LOC100071448,LOC100071452,LOC100071460,LOC100071469,LOC100071479,LOC100071486	OR1L4,OR1L4,OR1L4,OR1L6,OR1L3,OR1L1,OR1B1,OR1Q1,OR1N1,OR1J2,OR1N2,OR1L8,OR1L8,OR1J2,OR1J4,OR1J4,OR1J4,OR1J4,OR1L8,OR1L8,OR1N1,OR1J1,OR1J2,OR1J4,OR1J4,OR1J2,OR1J2,OR1L6,OR1B1,Olfr859,OR1Q1,OR1N2,OR1G1,OR1N2,OR1J1,OR1J2,OR1L6,OR1L3,OR1L1
25	26361000	27125754	764755	HAN 1,HOL 1,LUS 1,MAR 2		RC3H2,LOC100071170,LOC100071180,LOC100067499,LOC100071189,LOC100630709,LOC100071212,LOC100071218,LOC100071227,LOC100071236,LOC100071244,LOC100071251,LOC100067520,LOC100071258,LOC100071264,LOC100071270,LOC100071275,LOC100071278,LOC100071283,LOC100071287,LOC100071297,LOC100146817,LOC100071311,LOC100071317,LOC100147676,LOC100071322,LOC100071329,LOC100071332,LOC100071338,LOC100071346,LOC100071352,LOC100071358,LOC100071365,LOC100071376,LOC100071382,LOC100071392,LOC100071402,LOC100071407,LOC100071413,LOC100071420,LOC100146823,LOC100071438,LOC100071445,LOC100071448,LOC100071452,LOC100071460,LOC100071469,LOC100071479,LOC100071486	RC3H2,DPRX,PDCL,OR1K1,OR5C1,OR1G1,OR1L6,OR1L6,OR1L6,OR1L4,OR1L4,OR1L4,OR1L4,OR1L6,OR1L3,OR1L1,OR1B1,OR1Q1,OR1N1,OR1J2,OR1N2,OR1L8,OR1L8,OR1J2,OR1J4,OR1J4,OR1J4,OR1J4,OR1L8,OR1L8,OR1N1,OR1J1,OR1J2,OR1J4,OR1J4,OR1J2,OR1J2,OR1L6,OR1B1,Olfr859,OR1Q1,OR1N2,OR1G1,OR1N2,OR1J1,OR1J2,OR1L6,OR1L3,OR1L1
