# Supplementary figures and images for: Analysis of copy number variants by three detection algorithms and their association with body size in horses
Source: BMC Genomics. 2013 Jul 18;14:487. doi: 10.1186/1471-2164-14-487 (PMC3720552; doi:10.1186/1471-2164-14-487)

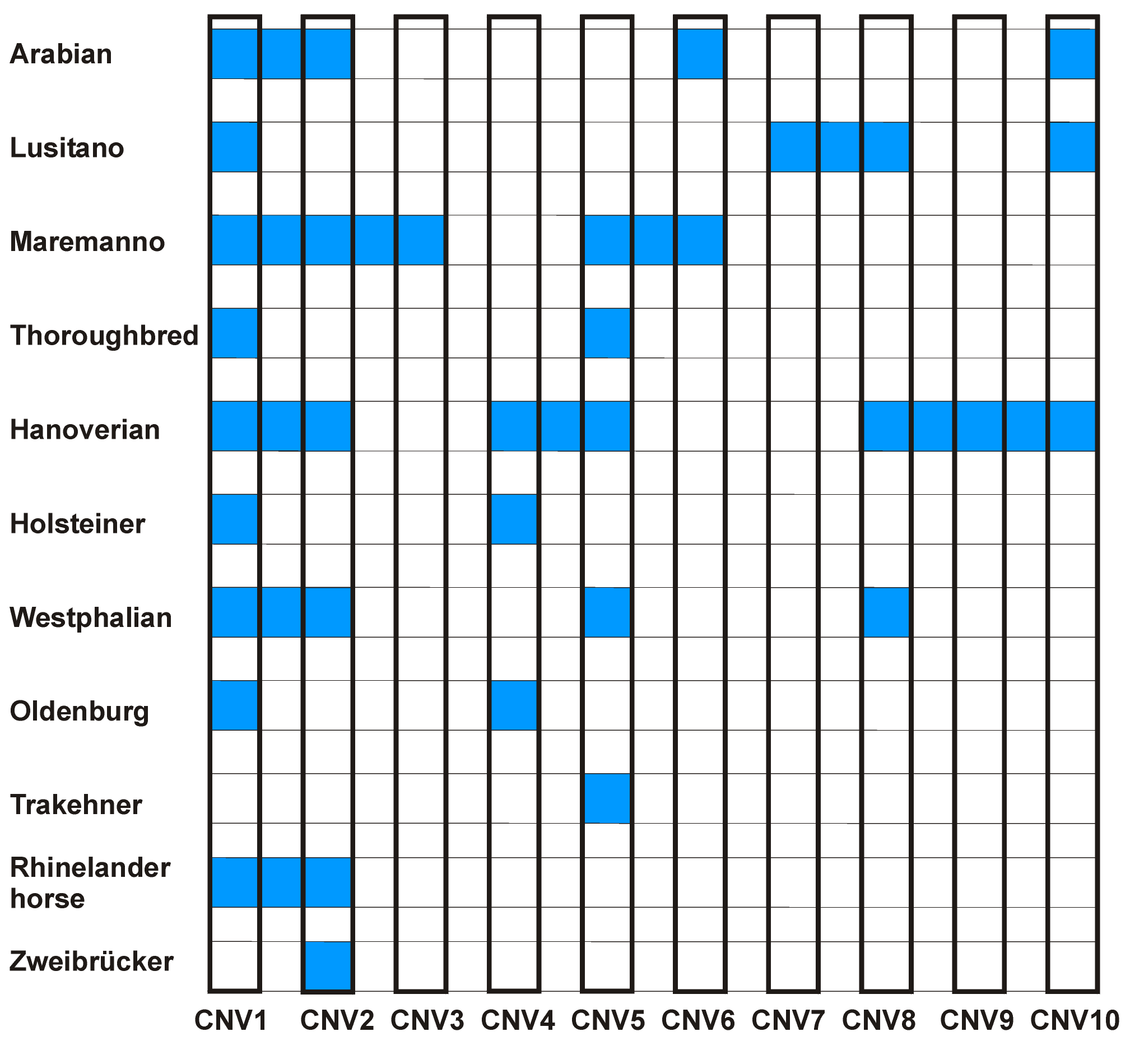

Supplement: Additional file 5 — Display of CNVs detected in two Przewalski horses by comparative analysis of three algorithms. The overlap of CNVs with different breeds is shown. [file 1471-2164-14-487-S5.tiff]
