# Supplementary material for: Analysis of copy number variants by three detection algorithms and their association with body size in horses
Source: BMC Genomics. 2013 Jul 18;14:487. doi: 10.1186/1471-2164-14-487 (PMC3720552; doi:10.1186/1471-2164-14-487)
Supplement: Additional file 6 — Functional annotation analysis of enriched genes derived from 45 CNV regions using DAVID 6.7. In five CNV regions we could not detect any genes for evaluation. Text in DOC format. [file 1471-2164-14-487-S6.docx]

**Additional file 6. Functional annotation analysis of enriched genes derived from 45 CNV regions using DAVID 6.7.**

| **Enriched terms** | **Involved genes (n)** | **Involved genes (%)** | **P-value (EASE score)** | **Bonferroni** |
| --- | --- | --- | --- | --- |
| **Sensory perception** |  |  |  |  |
| olfaction | 100 | 76.3 | 4.20E-142 | 4.90E-140 |
| Olfactory receptor | 100 | 76.3 | 2.40E-134 | 2.30E-132 |
| sensory perception of smell | 100 | 76.3 | 6.90E-132 | 1.30E-129 |
| sensory transduction | 103 | 78.6 | 1.50E-130 | 8.50E-129 |
| olfactory receptor activity | 101 | 77.1 | 3.80E-130 | 3.60E-128 |
| sensory perception of chemical stimulus | 100 | 76.3 | 6.60E-127 | 6.40E-125 |
| 7TM GPCR rhodopsin-like | 103 | 78.6 | 2.50E-115 | 7.90E-114 |
| GPCR rhodopsin-like superfamily | 103 | 78.6 | 2.10E-115 | 1.00E-113 |
| g-protein coupled receptor | 103 | 78.6 | 1.40E-115 | 5.40E-114 |
| sensory perception | 103 | 78.6 | 2.40E-108 | 1.50E-106 |
| G protein-coupled olfactory receptor | 95 | 72.5 | 8.40E-107 | 1.90E-105 |
| cognition | 103 | 78.6 | 5.60E-103 | 2.70E-101 |
| Olfactory transduction | 91 | 69.5 | 7.00E-96 | 1.70E-94 |
| rhodopsin-like G protein-coupled receptors | 103 | 78.6 | 3.10E-91 | 3.60E-90 |
| neurological system process | 103 | 78.6 | 7.90E-90 | 2.50E-88 |
| Olfactory receptor MOR106 | 4 | 3.1 | 8.90E-06 | 2.10E-04 |
| G protein-coupled olfactory receptor class I | 6 | 4.6 | 2.10E-03 | 1.60E-02 |

**Additional file 6** continued.

| **Enriched terms** | **Involved genes (n)** | **Involved genes (%)** | **PValue (EASE score)** | **Bonferroni** |
| --- | --- | --- | --- | --- |
| **Signal transduction** |  |  |  |  |
| transducer | 103 | 78.6 | 2.20E-112 | 6.40E-111 |
| G-protein coupled receptor protein signaling pathway | 103 | 78.6 | 3.10E-93 | 1.20E-91 |
| receptor | 106 | 80.9 | 2.60E-90 | 6.00E-89 |
| cell surface receptor linked signal transduction | 104 | 79.4 | 3.30E-72 | 9.10E-71 |
| **Cellular component** |  |  |  |  |
| cell membrane | 103 | 78.6 | 7.00E-71 | 1.30E-69 |
| glycosylation site:N-linked (GlcNAc...) | 109 | 83.2 | 1.00E-50 | 7.20E-49 |
| transmembrane | 113 | 86.3 | 1.60E-47 | 2.10E-46 |
| transmembrane region | 112 | 85.5 | 1.50E-46 | 8.10E-45 |
| membrane | 117 | 89.3 | 1.20E-41 | 1.40E-40 |
| plasma membrane | 106 | 80.9 | 3.40E-38 | 2.40E-36 |
| integral to membrane | 114 | 87 | 2.30E-32 | 7.90E-31 |
| intrinsic to membrane | 114 | 87 | 9.40E-31 | 2.20E-29 |
| **Miscellaneous** |  |  |  |  |
| disulfide bond | 100 | 76.3 | 1.40E-54 | 2.40E-53 |
| glycoprotein | 109 | 83.2 | 5.40E-49 | 7.80E-48 |
