# Supplementary material for: Analysis of copy number variants by three detection algorithms and their association with body size in horses
Source: BMC Genomics. 2013 Jul 18;14:487. doi: 10.1186/1471-2164-14-487 (PMC3720552; doi:10.1186/1471-2164-14-487)
Supplement: Additional file 7 — Gene ontology analysis of significantly over- and underrepresented genes involved in molecular functions and protein classes. The software PANTHER was used for the evaluation of 45 CNV regions detected by all three algorithms. [file 1471-2164-14-487-S7.docx]

**Additional file 7. Gene ontology analysis of significantly over- and underrepresented genes involved in molecular functions and protein classes.** The software PANTHER was used for the evaluation of 45 CNV regions detected by all three algorithms.

|  | **Number of reference genes**  **(Homo sapiens)** | **Number of genes in CNV regions (Horse)** | **Expected** | **Raw P-value** | **Bonferroni corrected P-value for multiple testing** |
| --- | --- | --- | --- | --- | --- |
| **Molecular Function-**  **overrepresented genes** | | | | |  |
| G-protein coupled receptor activity | 504 | 21 | 3.10 | 5.58E-12 | 8.32E-10 |
| receptor activity | 1917 | 24 | 11.79 | 5.95E-04 | 8.87E-02 |
| **Molecular Function-**  **underrepresented genes** |  |  |  |  |  |
| structural constituent of cytoskeleton | 1028 | 0 | 6.32 | 1.52E-03 | 2.26E-01 |
| transcription factor activity | 2179 | 1 | 13.40 | 1.10E-05 | 1.65E-03 |
| transferase activity | 1625 | 2 | 9.99 | 2.10E-03 | 3.13E-01 |
| binding | 6916 | 7 | 42.53 | 1.98E-14 | 2.95E-12 |
| catalytic activity | 5410 | 8 | 33.27 | 6.38E-09 | 9.50E-07 |
| transcription regulator activity | 2179 | 1 | 13.40 | 1.10E-05 | 1.65E-03 |
| protein binding | 3248 | 5 | 19.98 | 2.55E-05 | 3.79E-03 |
| hydrolase activity | 2256 | 2 | 13.87 | 5.58E-05 | 8.31E-03 |
| structural molecule activity | 1578 | 1 | 9.70 | 4.70E-04 | 7.00E-02 |
| DNA binding | 2455 | 2 | 15.10 | 1.67E-05 | 2.48E-03 |
| nucleic acid binding | 3885 | 2 | 23.89 | 1.35E-09 | 2.02E-07 |
| **Protein Class-**  **overrepresented genes** | | | | |  |
| receptor | 1904 | 24 | 11.71 | 5.40E-04 | 9.72E-02 |
| G-protein coupled receptor | 504 | 21 | 3.10 | 5.58E-12 | 1.01E-09 |
| **Protein Class -**  **underrepresented genes** | | | | |  |
| hydrolase | 1884 | 2 | 11.59 | 4.93E-04 | 8.87E-02 |
| cytoskeletal protein | 1028 | 0 | 6.32 | 1.52E-03 | 2.73E-01 |
| RNA binding protein | 1139 | 0 | 7.00 | 7.38E-04 | 1.33E-01 |
| transferase | 1571 | 2 | 9.66 | 2.82E-03 | 5.07E-01 |
| transcription factor | 2179 | 1 | 13.40 | 1.10E-05 | 1.99E-03 |
| nucleic acid binding | 2806 | 2 | 17.26 | 1.86E-06 | 3.34E-04 |
