# Supplementary material for: Analysis of copy number variants by three detection algorithms and their association with body size in horses
Source: BMC Genomics. 2013 Jul 18;14:487. doi: 10.1186/1471-2164-14-487 (PMC3720552; doi:10.1186/1471-2164-14-487)
Supplement: Additional file 8 — Genomic regions analysed for copy number variants (CNVs) by real-time quantitative RT-qPCR. Primer sequences, their position, product size, annealing temperature and TaqMan probes are shown. Text in DOC format. [file 1471-2164-14-487-S8.docx]

**Additional file 8.** **Genomic regions analysed for copy number variants (CNVs) by real-time quantitative RT-qPCR.** Primer sequences, their position, product size, annealing temperature and TaqMan probes are shown.

| ECA | Gene name | Primers forward (F) and reverse (R) | Start | End | Length (bp) | AT^a^ (°C) | TaqMan probe |
| --- | --- | --- | --- | --- | --- | --- | --- |
| 1 | Olfr1284 (ENSECAG00000006791) | F-GCCATGGCATTTGACAGGTA  R-ACCCAGCCACTGATAACAAGTGA | 155,625,509 | 155,625,609 | 101 | 59.5 | CTTGAACATCATGAACCGTA |
| 1 | OR4K2 (ENSECAG00000006318) | F-CTCCCATTCTGAACCCAGTAATCT  R-AATGTGAAGGAGTTGCCTTGTTG | 156,690,447 | 156,690,557 | 111 | 59.5 | TGAGGAAACTGAAGAAAA |
| 8 | IGLV3-32 (ENSECAG00000005113) | F-TGTGGCCTCTTCTGAGCTGAA  R-TACCAGCTAGCATAACTGTTTCTTAAGC | 4,502,751 | 4,502,861 | 111 | 59.5 | ATCACCTGCCAGGGAG |
| 6 | GAPDH (ENSECAG00000003944) | F-CGATGCTGGTGCTGAATATGTT  R-GGTCAACTCCCCTCATCTTTAGC | 34,076,957 | 34,077,058 | 102 | 59.5 | TCTTCACTACCTTGGAGAAG |

AT ^a^: Annealing temperature
