# Supplementary material for: Analysis of copy number variants by three detection algorithms and their association with body size in horses
Source: BMC Genomics. 2013 Jul 18;14:487. doi: 10.1186/1471-2164-14-487 (PMC3720552; doi:10.1186/1471-2164-14-487)
Supplement: Additional file 9 — Copy number detection accuracy of three SNP array algorithms. The deleted (0, 1) or duplicated (3) copy numbers validated by qPCR are compared with the detection results of CNVPartition, PennCNV and QuantiSNP. Reference samples with the copy number 2 are not displayed as they were selected specifically for two copies in all three programs. [file 1471-2164-14-487-S9.docx]

**Additional file 9. Copy number detection accuracy of three SNP array algorithms.** The deleted (0, 1) or duplicated (3) copy numbers validated by qPCR are compared with the detection results of CNVPartition, PennCNV and QuantiSNP. Reference samples with the copy number 2 are not displayed as they were selected specifically for two copies in all three programs.

| **Sample** | **Copy number validated by qPCR** | **Copy number detected by**  **CNVPartition** | **Copy number detected by**  **PennCNV** | **Copy number detected by**  **QuantiSNP** |
| --- | --- | --- | --- | --- |
| **ECA1: ENSECAG00000006791** | |  |  |  |
| Horse 1 | 0 | 0 | 0 | 0 |
| Horse 2 | 0 | 0 | 0 | 0 |
| Horse 3 | 1 | 1 | 2 | 2 |
| Horse 4 | 1 | 2 | 1 | 1 |
| Horse 5 | 1 | 2 | 1 | 1 |
| Horse 6 | 1 | 2 | 1 | 1 |
| Horse 7 | 1 | 1 | 1 | 1 |
| Horse 8 | 1 | 1 | 1 | 1 |
| Horse 9 | 1 | 1 | 1 | 1 |
| Horse 10 | 1 | 1 | 1 | 1 |
| False discovery rate |  | 30% | 10% | 10% |
| **ECA1: ENSECAG00000006318 (OR4K2)** | |  |  |  |
| Horse 1 | 1 | 2 | 1 | 1 |
| Horse 2 | 1 | 2 | 2 | 1 |
| Horse 3 | 1 | 1 | 1 | 1 |
| Horse 4 | 1 | 2 | 2 | 1 |
| Horse 5 | 1 | 2 | 1 | 1 |
| Horse 6 | 1 | 1 | 1 | 1 |
| Horse 7 | 1 | 2 | 1 | 1 |
| Horse 8 | 1 | 2 | 1 | 1 |
| Horse 9 | 1 | 1 | 1 | 1 |
| Horse 10 | 1 | 2 | 1 | 2 |
| False discovery rate |  | 70% | 20% | 10% |

**Additional file 9 continued.**

| **Sample** | **Copy number validated by qPCR** | **Copy number detected by**  **CNVPartition** | **Copy number detected by**  **PennCNV** | **Copy number detected by**  **QuantiSNP** |
| --- | --- | --- | --- | --- |
| **ECA8: ENSECAG00000005113** | |  |  |  |
| Horse 1 | 3 | 2 | 2 | 3 |
| Horse 2 | 3 | 2 | 2 | 3 |
| Horse 3 | 3 | 2 | 2 | 3 |
| Horse 4 | 3 | 2 | 2 | 3 |
| Horse 5 | 3 | 2 | 2 | 3 |
| Horse 6 | 3 | 2 | 2 | 3 |
| Horse 7 | 0 | 0 | 2 | 2 |
| Horse 8 | 1 | 2 | 1 | 1 |
| Horse 9 | 1 | 2 | 1 | 1 |
| Horse 10 | 1 | 2 | 1 | 1 |
| False discovery rate |  | 90% | 70% | 10% |
