# Supplementary material for: Analysis of copy number variants by three detection algorithms and their association with body size in horses
Source: BMC Genomics. 2013 Jul 18;14:487. doi: 10.1186/1471-2164-14-487 (PMC3720552; doi:10.1186/1471-2164-14-487)
Supplement: Additional file 10 — Comparison of CNVs validated by qPCR in CGH analysis (Doan et al.) with CNVs detected by CNVPartition, PennCNV and QuantiSNP. The number of samples detected by QuantiSNP is considerably high. [file 1471-2164-14-487-S10.docx]

**Additional file 10. Comparison of CNVs validated by qPCR in CGH analysis (Doan *et al.*) with CNVs detected by CNVPartition, PennCNV and QuantiSNP.** The number of samples detected by QuantiSNP is considerably high.

| **Chrom** | **Product position in bp**  **(validated by qPCR;**  **Doan *et al.*)** | **Number of samples with a duplication or deletion detected by**  **CNVPartition (n)** | **Number of samples with a duplication or deletion detected by**  **PennCNV (n)** | **Number of samples with a duplication or deletion detected by**  **QuantiSNP (n)** |
| --- | --- | --- | --- | --- |
| 1 | 155.376.465-155.376.598 | 4 | 0 | 1 |
| 1 | 155.625.474-155.625.615 | 35 | 41 | 115 |
| 12 | 14.666.629-14.666.771 | 3 | 9 | 8 |
| 6 | 72.246.758-72.246.881 | 36 | 61 | 68 |
